# Manganese-Pincer-Catalyzed Nitrile Hydration, α-Deuteration, and α-Deuterated Amide Formation via Metal Ligand Cooperation

**DOI:** 10.1021/acscatal.1c01748

**Published:** 2021-08-03

**Authors:** Quan-Quan Zhou, You-Quan Zou, Sayan Kar, Yael Diskin-Posner, Yehoshoa Ben-David, David Milstein

**Affiliations:** ^†^Department of Molecular Chemistry and Materials Science and ^‡^Chemical Research Support, Weizmann Institute of Science, Rehovot 76100, Israel

**Keywords:** hydration, α-deuteration, manganese pincer complex, metal−ligand cooperation, nitriles

## Abstract

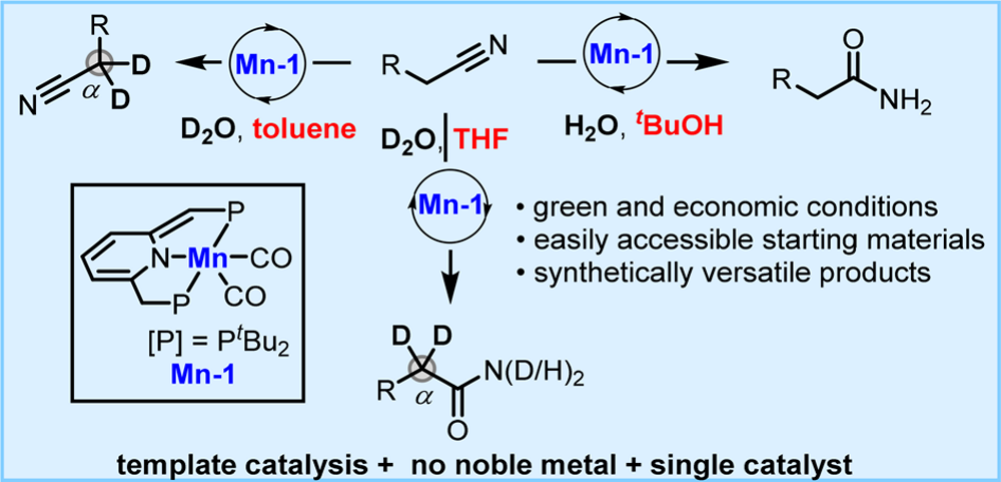

A simple and efficient system for the hydration and α-deuteration of nitriles to form amides, α-deuterated nitriles, and α-deuterated amides catalyzed by a single pincer complex of the earth-abundant manganese capable of metal–ligand cooperation is reported. The reaction is selective and tolerates a wide range of functional groups, giving the corresponding amides in moderate to good yields. Changing the solvent from *tert*-butanol to toluene and using D_2_O results in formation of α-deuterated nitriles in high selectivity. Moreover, α-deuterated amides can be obtained in one step directly from nitriles and D_2_O in THF. Preliminary mechanistic studies suggest the transformations contributing toward activation of the nitriles via a metal–ligand cooperative pathway, generating the manganese ketimido and enamido pincer complexes as the key intermediates for further transformations.

Amides are ubiquitous in biological macromolecules, polymers, and pharmaceuticals, as well as approved drugs,^1^ and great efforts have been devoted to the synthesis of functionalized amides.^2^ Nitriles constitute important building blocks which can be easily elaborated into various useful and fine chemicals.^3^ An atom-economical and environmentally benign method to access primary amides is the direct hydration of nitriles.^4^ Traditional hydration procedures require strong and stoichiometric amounts of bases or acids often under relatively harsh conditions that can cause overhydrolysis (to carboxylic acids) and are poorly tolerated by other sensitive functional groups.^1c,5,7^ During the past few years, homogeneous transition metal catalyzed hydration of nitriles have been reported using complexes of Ru,^6a−6e,13^ Rh,^6f^ Au,^6g^ Pt,^6h−6k^ Pd,^6l^ Co,^6m^ and Ni,^6n,6o^ where the metal center acts as a Lewis acid to activate the nitrile group and facilitate the nucleophilic attack by water.^7^ Nevertheless, some of these catalytic systems still need additives to promote a high conversion.^8^ Moreover, the synthesis of α-deuterated amides, which are of interest in the pharmaceutical sector, is still unexplored under similar conditions.

Metal–ligand cooperation (MLC) using pincer complexes has attracted much interest toward the deveopment of green and sustainable synthetic transformations.^9^ Recent investigations have shown that MLC of dearomatized lutidine-based pincer complexes can provide a new approach to the activation of double and triple bonds via simultaneos substrate binding to the ligand and metal center.^10^ In this regard, we have observed that rhenium and manganese pincer complexes catalyze the addition of nitriles to Michael acceptors, as well as *oxa*- and *aza*-Michael addition reactions, in which the catalytic activity is based on the nitrile bound to the complex via C–C and M–N bond formation of the C≡N group, which we termed “Template Catalysis”, (Scheme 1a).^11^ The Otten group also developed *oxa*-Michael addition of alcohols to unsaturated nitriles, and hydration of nitriles, via template activation of the C≡N bond catalyzed by a ruthenium pincer complex (Scheme 1a).^12,13^

**Scheme 1:**
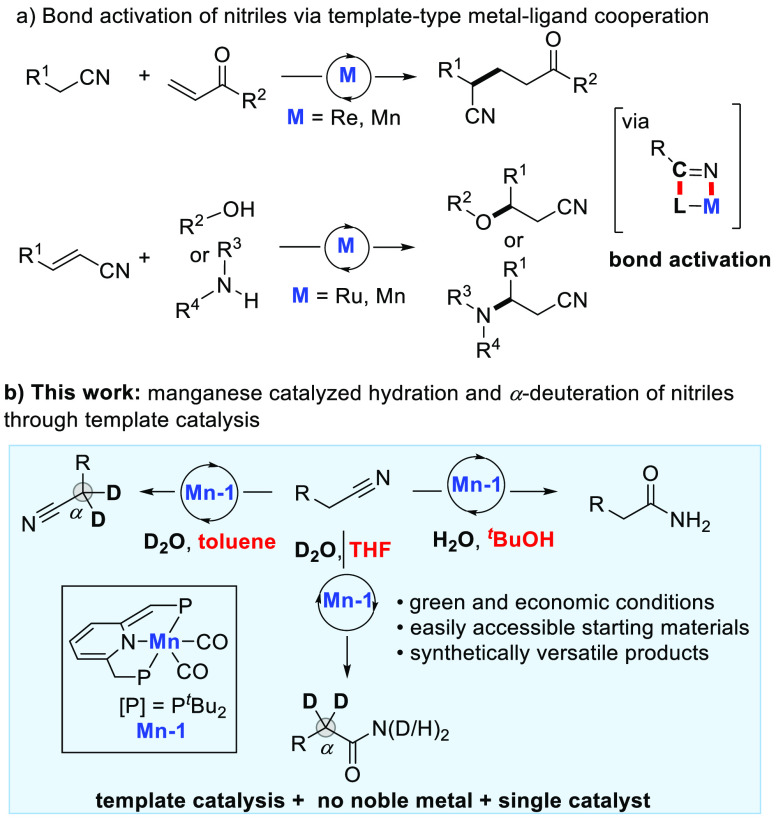
Activation of Nitriles through Template Catalysis by Transition Metal Complexes

Reactions catalyzed by earth-abundant metals (e.g., Fe, Co, Ni, Mn) have received considerable attention in recent years.^14^ Compared with noble-metals, these base-metals are inexpensive and generally display lower toxicity, making their application preferable in organic synthesis. In 2016, our group reported the first example of a reaction catalyzed by a manganese pincer complex, namely dehydrogenative coupling of alcohols and amines to form aldimines.^15^ As part of our ongoing research on sustainable catalysis using earth-abundant metals, we herein disclose a catalytic nitrile hydration route based on our previously reported template catalysis,^11^ catalyzed by a pincer complex of the earth abundant manganese (Scheme 1b). α-Deuterated nitriles and amides were also easily accessed using D_2_O as the deuterium source.

We started our investigations with a model reaction of benzyl nitrile **1a** (1.0 mmol) and H_2_O (5.0 mmol) (Table 1). Encouragingly, the desired amide **2a** was afforded in 99% GC yield using 1 mol % of dearomatized manganese PNP complex **Mn-1** as the catalyst in *tert*-butanol as solvent (97% isolated yield, entry 1). By changing the catalyst to the dearomatized PNN complex (**Mn-2**) or the bipyridine-based PNN complex (**Mn-3**), lower yields were recorded (entries 2–3, 20% and 65% yield, respectively). Screening solvents revealed that THF, 2-propanol, and *tert*-amyl alcohol were also adequate, whereas the reaction did not take place in toluene (entries 4–7). In addition, a higher loading of water led to reduced reaction efficiency (entry 8). Moreover, the reaction can be performed smoothly at room temperature to give the amide product in 55% yield (entry 9). A control experiment indicated that the manganese pincer catalyst was essential for this transformation (entry 10).

**Table 1:**
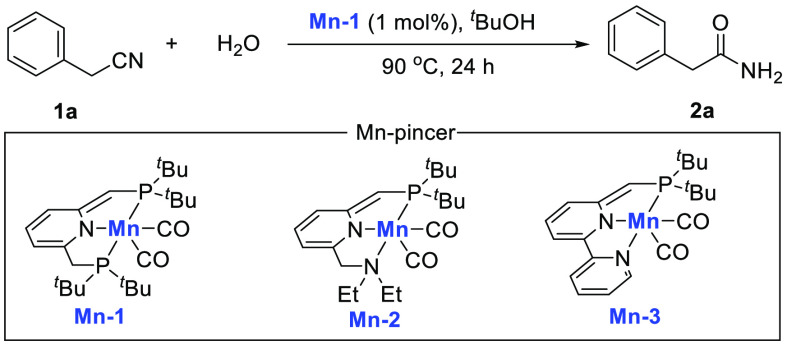
Optimizing of the Reaction Conditions^a^

entry	variation from the standard conditions	yield (%)^b^
1	none	99 (97)^c^
2	**Mn-2** instead of **Mn-1**	20
3	**Mn-3** instead of **Mn-1**	65
4	**THF** instead of ^***t***^**BuOH**	96
5	**Toluene** instead of ^***t***^**BuOH**	0
6	**2-propanol** instead of ^***t***^**BuOH**	90
7	**tert-Amyl alcohol** instead of ^***t***^**BuOH**	95
8	1 mL **H**_**2**_**O** instead of 5 mmol **H**_**2**_**O**	17
9	rt for 24 h	55
10	no **Mn-1**	0

a Reaction conditions: **1a** (1.0 mmol), **H**_**2**_**O** (5.0 mmol, 5.0 equiv), **Mn-1** (0.01 mmol, 1 mol %), and ^*t*^BuOH (1.0 mL) at 90 °C for 24 h.

b Yields were determined by GC with biphenyl as an internal standard.

c Isolated yield in parentheses.

With the optimal reaction conditions in hand, the scope of the hydration reaction was studied. As shown in Table 2, various benzyl nitriles **1a**–**e** bearing electron-withdrawing (-F, -Br) and electron-donating (-Me, -OMe) groups on the benzene ring exhibited high hydration efficiency and delivered the desired products in excellent yields (**2a**–**e**, 92–99%). A gram-scale hydration reaction of **1a** using only 0.5 mol % catalyst was also carried out to further demonstrate the synthetic utility (10 mmol scale, 1.21 g **2a**, 90% yield). Benzyl nitrile containing a heteroaromatic group also worked well to give the corresponding amide in a moderate yield (**2f**, 55%). Next, a variety of nonactivated aliphatic nitriles (**1g**–**l**) were examined. Significantly, aliphatic nitriles also reacted well, forming the corresponding amides in 55–95% yields (**2g**–**l**). In addition to alkyl nitriles, benzonitrile and its derivatives were also compatible with the optimal conditions. Reactions of benzonitriles bearing electron-neutral (-H), electron-withdrawing (-F, -Cl, -Br -NO_2_, and -COCH_3_) and electron-donating (-Me, -OMe) substituents took place smoothly to afford the primary amide products in good to excellent yields (**2m**–**v**, 53–97% yields). Moreover, furan-2-carbonitrile and 2-naphthonitrile also readily hydrolyzed to the corresponding amides **2w**, and **2x** in 72% and 94% yields, respectively. It should be noted that due to the competing N-H or -OH activation by the catalyst, nitriles bearing aldehyde, alcohol, or amine substituents did not react under the standard conditions.

**Table 2:**
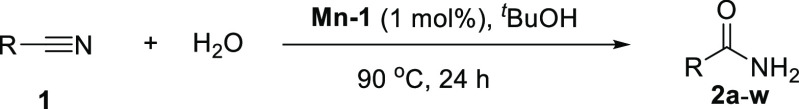

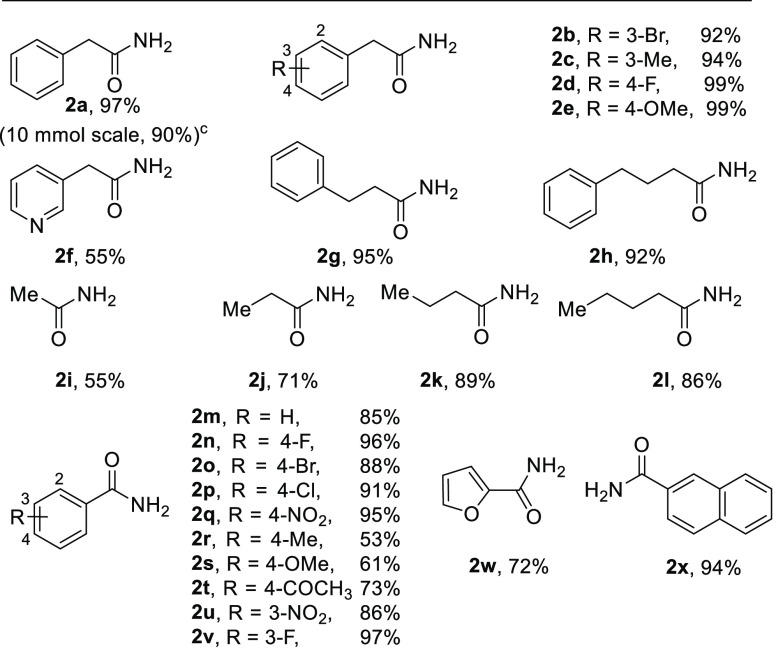
Substrate Scope of the Hydration Reaction^a^^,^^b^

a Reaction conditions: nitrile (1.0 mmol), H_2_O (5.0 mmol, 5.0 equiv), **Mn-1** (0.01 mmol, 1 mol %), and ^*t*^BuOH (1.0 mL) at 90 °C for 24 h.

b Isolated yield.

c **1a** (10.0 mmol), **Mn-1** (0.05 mmol, 0.5 mol %).

The selective ruthenium pincer complex catalyzed *α-* and β-deuteration of alcohols was previously reported by us.^16^ Keeping in mind the diverse transformation of nitriles, it is desirable to synthesize α-deuterated nitriles, as key intermediates toward the synthesis of various α-deuterated carbonyl compounds. Hence we became interested in the α-deuteration of nitriles using our manganese pincer complexes. Noteworthy, ruthenium-catalyzed α-deuteration of aliphatic nitriles using D_2_O was reported by Gunanathan.^17^ We observed that although hydration of nitriles did not proceed in toluene, nitriles could still bind very well to the dearomatized manganese pincer complex **Mn-1** (see Supporting Information Figure S3 for details). Significantly, reaction of 4-phenylbutanenitrile (**1h**) with D_2_O at 70 °C in the presence of 1 mol % of **Mn-1**, the α-deuterated product **3h** was obtained with 98% deuteration (against a theoretical maximum 98% deuteration), and no hydration product was observed (Table 3, entry 1). Although the deuteration is conducted with a higher water (D_2_O) amount, the reaction is likely biphasic with low effective water concentration in the organic layer where catalysis is likely to happen. Thus, it is possible to selectively obtain α-deuterated nitriles using D_2_O in toluene.

**Table 3:**
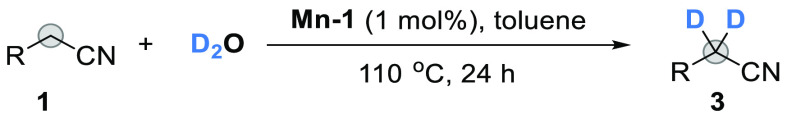

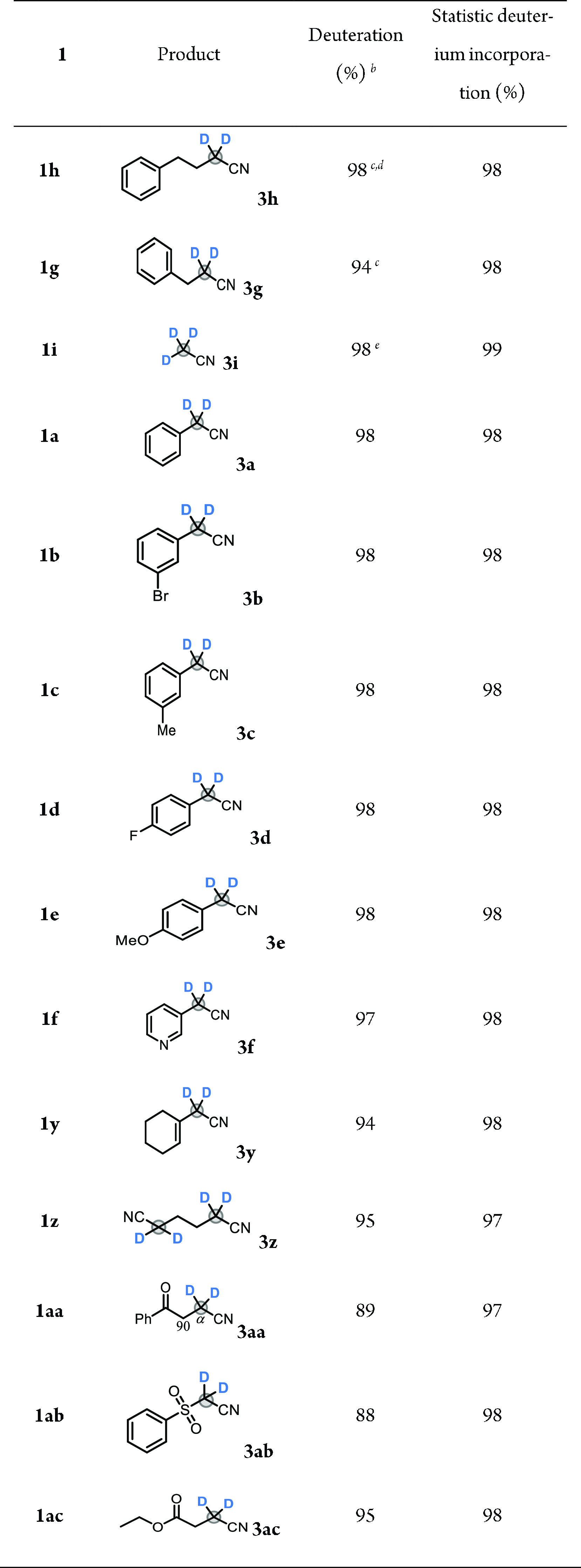
Manganese-Catalyzed α-Deuteration of Aliphatic Nitriles^a^

a Reaction conditions: nitrile (0.50 mmol), **Mn-1** (1 mol %), D_2_O (0.5 mL) and toluene (0.5 mL) at 110 °C (bath temperature) for 24 h.

b After the reaction was complete, ethyl acetate was extracted, and the solvent was removed, the degree of α-deuteration was calculated from ^**1**^**H NMR**.

c Reaction performed at 70 °C.

d The reaction could be performed smoothly at room temperature with 78% α-deuteration after 36 h.

e 1.0 mL D_2_O as solvent, methanol (0.50 mmol) was added as internal standard.

Table 3 shows the substrate scope of the **Mn-1** catalyzed α-deuteration of nitriles. Aliphatic primary nitriles such as 4-phenylbutanenitrile **1h** and 3-phenylpropanenitrile **1g** were fully converted into the corresponding α-deuterated derivatives with excellent selectivities (98% and 94% α-deuteration, respectively). Deuterated acetonitrile **3i** was formed with 98% deuteration when acetonitrile was used. Benzyl and heteroaromatic nitriles were also selectively converted into α-deuterated nitriles with excellent selectivities (**3a**–**f**, 97–98% α-deuteration). 1-Cyclohexeneacetonitrile **1y** was also selectively converted into the α-deuterated product **3y** with 94% α-deuteration. Notably, the reaction could be extended to aliphatic dinitriles, giving the α-deuterated product, at both the α-positions of the two CN groups with 95% deuteration (**3z**). The reaction proceeded well for the ketone substituted nitrile 4-oxo-4-phenylbutanenitrile **1aa** with 89% α-deuteration, together with the deuteration of the carbonyl α-position with a 90% deuteration, further showing the utility of our methodology. Sulfonyl- and ester-substituted nitriles displayed efficient deuteration at α-position (**3ab**, 88% α-deuteration, **3ac**, 95% α-deuteration). The good tolerance of this α-deuteration process using readily available nitriles and D_2_O further demonstrates the utility of our earth-abundant manganese catalytic MLC system.

The hydration and α-deuteration reactions inspired us to explore the synthesis of α-deuterated amides using the same catalyst in a one step process. As exemplified in Scheme 2, α-deuterated amide **4** did indeed form in excellent 94% yield with 92% α-deuteration by simply switching the solvent to THF. To the best of our knowledge, this is the first example of transtion-metal-catalyzed one-step construction of α-deuterated amides from nitriles. An attempt for direct deuteration of the amide 3-phenylpropanamide **2g** under the reaction conditions resulted in no α-deuterated product. This implies that in the one-pot reaction, α-deuteration precedes the hydration reaction.

**Scheme 2:**
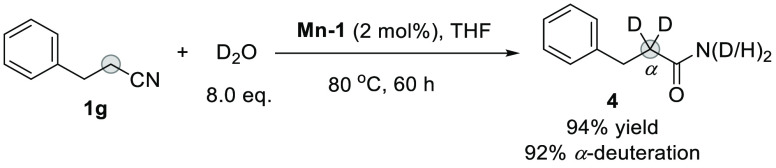
α-Deuterated Amide Formed by a One-Step Nitrile Deuteration Hydration Reaction Using D_2_O

Mechanistic studies indicate that these transformations are initiated by bond activation via metal–ligand cooperation rather than simple coordination. As shown in Scheme 3, reaction of **Mn-1** with benzonitrile **1m**, lacking an α-methylene group, generates the [1,3]-addition product of ketimido manganese complex **Mn-5** (Scheme 3,a). **Mn-5** was detected as an intermediate in the corresponding hydration system, and it can catalyze the hydration reaction (see Supporting Information for more details about monitoring the ^31^P NMR spectra of the reaction conversion process). The observed ability of **Mn-1** to activate benzonitrile differs from the reactivity of dearomatized Ru PNP complexes with nitrile substrates under ambient temperature.^13^ Catalyst **Mn-1** readily reacts with benzyl nitrile **1a**, bearing an α-methylene group, to form the manganese enamido complex **Mn-6** (Scheme 3, b).^11b^ This intermediate was also detected as the major metal species at the beginning of hydration and α-deuteration reactions at room temperature. **Mn-6** is presumably in equilibrium with the tautometic imine ketimido manganese complex. Treating the enamido **Mn-6** with methyl triflate in benzene, the cationic imine complex **Mn-7** was afforded in 99% yield. Single-crystal X-ray diffraction analysis further confirmed the structure of complexes **Mn-5** and **Mn-7** (Figure 1).^18^ We also explored the catalytic performance of the isolated enamido complex **Mn-6** in the hydration and α-deuteration processes (Scheme 3, c). Complex **Mn-6** effectively catalyzes both reactions, suggesting that the catalytic pathways likely involve activation of nitriles by the **Mn-1** catalyst via MLC. These results also clearly show that the enamido complex **Mn-6** is a catalytic species in the hydration and α-deuteration of benzyl nitrile.

**Scheme 3:**
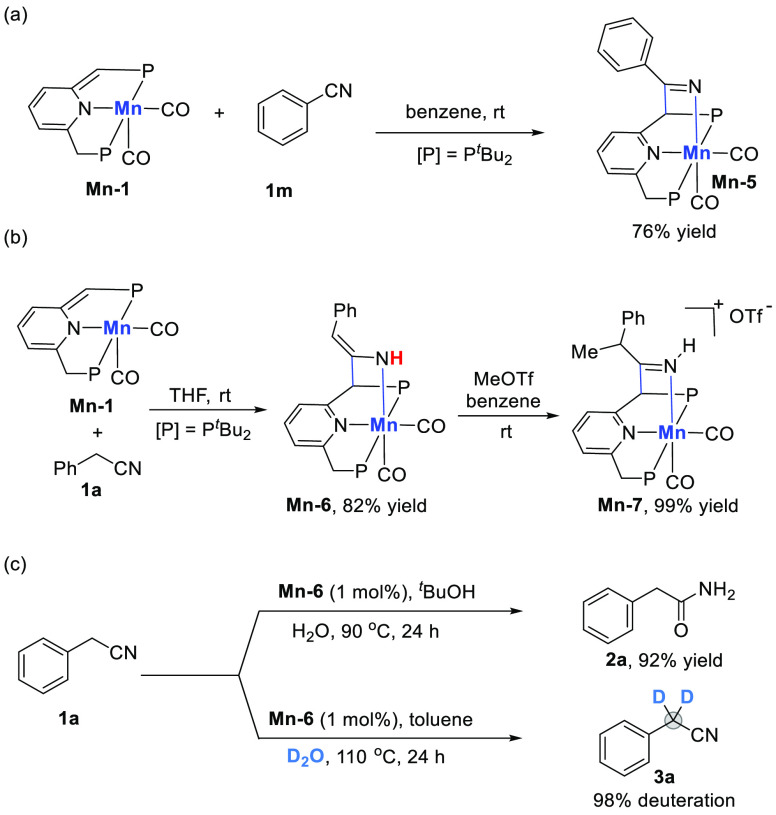
Mechanistic Studies

**Figure 1:**
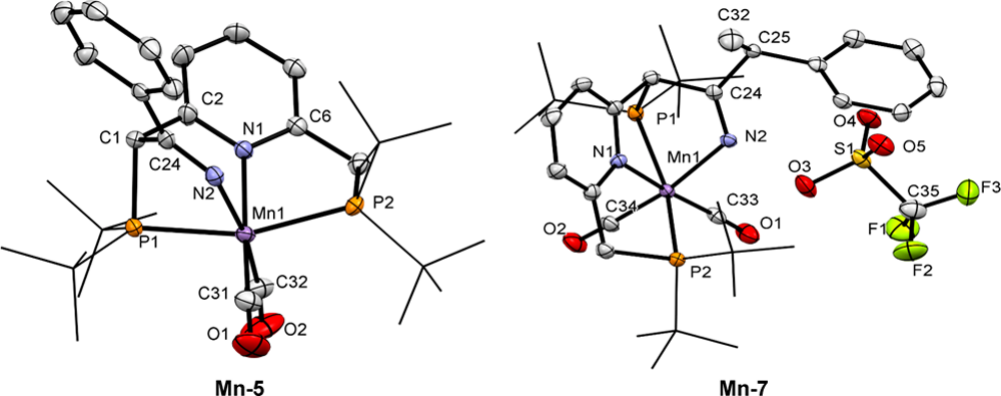
X-ray crystal structures of complexed **Mn-5** and **Mn-7**. Atoms are presented as thermal ellipsoids at 50% probability level. The P(*tert*-butyl)_2_ groups are drawn as wire frames, and hydrogen atoms are omitted for clarity. Selected bond lengths (Å) and angles (deg) of **Mn-5**: Mn(1)–N(1), 2.0530(13); Mn(1)–N(2), 2.0495(13); Mn(1)–P(1), 2.3239(9); Mn(1)–P(2), 2.3163(4); Mn(1)–C(31), 1.7812(17); Mn(1)–C(32), 1.7764(18); C(24)–C(1), 1.503(3); N(2)–C(24), 1.271(2); C(2)–C(1), 1.511(3); N(1)–C(2), 1.362(2); P(2)–Mn(1)–P(1), 159.65(3); C(2)–N(1)–Mn(1), 117.89(10), C(24)–N(2)–Mn(1), 116.38(12); C(24)–C(1)–C(2),105.34(17). Selected bond lengths (Å) and angles (deg) of **Mn-7**: Mn(1)–P(1), 2.3123(11); Mn(1)–P(2), 2.3307(11); Mn(1)–N(1), 2.058(3); Mn(1)–N(2), 2.053(3); Mn(1)–C(33),1.784(4); Mn(1)–C(34), 1.782(4); N(2)–C(24), 1.280(5); C(24)–C(25), 1.515(5); C(25)–C(32), 1.531(6); N(1)–Mn(1)–P(1), 80.19(9); N(1)–Mn(1)–P(2), 79.94(9); C(24)–N(2)–Mn(1), 119.1(3); C(24)–C(25)–C(32), 109.1(3).

Based on the previous reports and current observations, we propose possible catalytic pathways for the hydration and α-deuteration of nitriles. As depicted in Scheme 4, using benzyl nitrile **1a** as the model substrate, initially, the dearomatized manganese complex **Mn-1** reversibly binds benzyl nitrile **1a** in a cooperative^1,3^ addition manner via MLC to generate the ketimido manganese complex **B**. A side equilibrium of water addition to **Mn-1** to generate off-cycle intermediate **A** is also likely. Subsequently, nucleophilic attack on complex **B** by water gives intermediate **D**, which presumably produces **E** by C–C and Mn–N bond cleavage regenerating the dearomatized catalyst **Mn-1**, while **E** isomerizes to the final product amide **2a**. In addition, since benzyl nitrile bears an α-methylene group, ketimido complex **B** can undergo a reversible tautomeric [1,3]-proton shift to generate the enamido complex **C**. D_2_O enables a rapid equilibrium H/D substitution to produce the mono α-deuterated nitrile **H**, releasing the catalyst **Mn-1** and re-entering the next α-deuteration cycle (Scheme 4, b). The final di-α-deuterated nitrile **3a** is generated following a similar catalytic cycle.

**Scheme 4:**
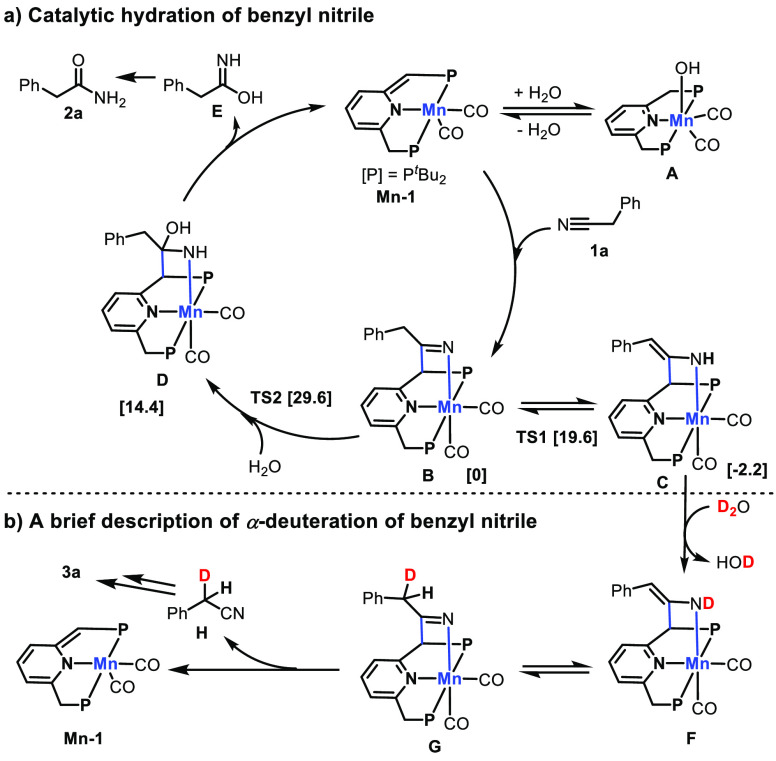
Proposed Reaction Pathways Numbers inside brackets correspond to the gas phase Gibbs free energies (kcal/mol) with respect to intermediate **B** + 2 H_2_O at 298 K and atmospheric pressure. Mass balance ensured throughout (see Supporting Information, section 6 for details).

We also conducted preliminary density functional theory (DFT) studies to verify that α-deuteration mechanism involving the formation of intermediate **C** from **B** is more favored than the hydration mechanism involving the generation of intermediate **D** from **B**. According to our calculations, the enamine complex **C** is thermodynamically more stable than the imine complex **B** by 2.2 kcal/mol in gas phase. However, the hydration intermediate **D** was found significantly higher in energy (14.4 kcal/mol). On a similar note, the relevant transition state leading to α-deuteration (TS1) was found less energetically demanding (19.6 kcal/mol) than the corresponding hydration transition state (TS2, 29.6 kcal/mol) (Figure S10). These results, along with the experimental observations, suggest that the deuteration pathway is more readily accessible than the hydration pathway. As a result, when toluene is used as solvent, only deuteration is observed because of limited solubility of water in toluene. Furthermore, when ^*t*^BuOH is used as solvent, both deuteration and hydration are observed.

In summary, we have developed the selective hydration of nitriles to amides using *t*-butanol as solvent. Switching the solvent to toluene and using D_2_O, α-deuterated nitriles are formed, with no hydration, being catalyzed for the first time by a manganese pincer complex. Both hydration and deuteration processes are atom economical and environmentally friendly using the same earth-abundant manganese-based catalyst, without producing waste. By switching the solvent to THF, we also realized the sequential α-deuteration and hydration processes to synthesize α-deuterated amides in a one-step process for the first time. Mechanistically these processes involve the reversible Mn–N and C–C bond formation between the substrate and the dearomatized manganese pincer complex through MLC. The postulated ketimido- and enamido-manganese intermediates were separately prepared and shown to catalyze the hydration and α-deuteration processes. We anticipate that this strategy can be further extended to other compounds with C≡C or C≡N moieties toward the assembly of structurally more complex bioactive molecules.

## Supporting Information Available

Experimental and spectroscopic details of the catalytic reactions (PDF). Crystallographic information file for **Mn-5** and **Mn-7**. (CIF) The Supporting Information is available free of charge at https://pubs.acs.org/doi/10.1021/acscatal.1c01748.

(PDF)
Crystallographic information file for **Mn-5** (CIF)
Crystallographic information file for **Mn-7** (CIF)
Crystallographic information file for **Mn-8** (CIF)

## Supplementary Material

cs1c01748_si_001.pdf

cs1c01748_si_002.cif

cs1c01748_si_003.cif

cs1c01748_si_004.cif
