# Supplementary material for: Manganese-Pincer-Catalyzed Nitrile Hydration, α-Deuteration, and α-Deuterated Amide Formation via Metal Ligand Cooperation
Source: ACS Catal. 2021 Aug 3;11(16):10239–45. doi: 10.1021/acscatal.1c01748 (PMC8383274; doi:10.1021/acscatal.1c01748)
Supplement: Supplementary file 1 — cs1c01748_si_001.pdf [file cs1c01748_si_001.pdf]

Supporting Information for

Manganese Pincer Catalyzed Nitrile Hydration, α-Deuteration, and α-Deuterated Amide Formation via Metal Ligand Cooperation

Quan-Quan Zhou,[†] You-Quan Zou,[†] Sayan Kar,[†] Yael Diskin-Posner,[‡] Yehoshoa Ben-David,[†] and David Milstein*,[†]

[†] Department of Molecular Chemistry and Materials Science and [‡]Chemical Research Support, Weizmann Institute of Science, Rehovot 76100, Israel

Correspondence to: david.milstein@weizmann.ac.il (D.M.).

Table of Contents

1. General Information S3

2. General Procedure for Manganese Pincer Catalyzed Hydration and *α*-Deuteration of Organonitriles S3

2.1 Description of the reaction setup S3

2.2 General procedure for hydration reaction of organonitriles S4

2.3 General procedure for *α*-deuteration of aliphatic nitriles S4

2.4 General procedure for one pot *α*-deuteration and hydration of nitrile S5

3. Synthetic Applications and Mechanistic Studies S6

3.1 Procedure for gram-scale reaction of **1a** S6

3.2 Procedure for transformation of primary amide products S6

3.3 Synthesis of complexes **Mn-5** and **Mn-7** S7

3.4 NMR studies of benzyl nitrile **1a** binding to the dearomatized manganese pincer complex **Mn-1** in toluene S8

3.5 The interconversion between the hydroxomanganase **A** and the enamido intermediate **B** S10

3.6 Catalytic performance of the isolated ketimido manganese complex **Mn-5** and enamido complex **Mn-6** in the hydration and *α*-deuteration reactions S11

3.7 ^{31}P NMR spectra of complexes for monitoring the reaction conversion process S12

3.8 Test base catalytic α-deuteration reactions. S15

4. Characterization Data of the Synthesized Compounds S16

4.1 Characterization data of amides and synthesized mangenese complex S16

4.2 Characterization data of *α*-deuterated nitriles S29

5. X-ray Crystal Structure Data of the Mnagenese Complex S39

6. Computational Details S45

7. NMR Spectra of the Synthesized Compounds S57

7.1 ^{1}H NMR and ^{13}C NMR spectra of the amide products and synthesized manganese complex S57

7.2 ^{1}H NMR, ^{2}H NMR and ^{13}C NMR spectra of the nitriles and *α*-deuterated nitriles S83

8. References S119

1. General Information

All experiments with metal complexes and phosphine ligands were carried out under an atmosphere of purified nitrogen in a Vacuum Atmospheres glove box equipped with a MO 40-2 inert gas purifier or using standard Schlenk techniques. All solvents were reagent grade or better. All non-deuterated solvents were purified according to standard procedures under argon atmosphere. All solvents were degassed with N_2 and kept in the glove box. Most of the chemicals used in the catalytic reactions were purified according to standard procedures (vacuum distillation) and stored over 3 Å molecular sieves.[1] Analytical TLC was performed on Merck silica gel 60 F_{254} plates. Purification of products was accomplished by flash column chromatography on silica gel 60 (Merck, particle diameter 40-63 μm). Flash column chromatography was performed using 200-300 mesh silica gel.[2] NMR spectra were recorded at room temperature either on a Bruker AMX-400 or an AMX-500. Chemical shifts of the NMR spectra are reported relative to $CDCl_3$ (^1H NMR: δ = 7.26 ppm, ^{13}C NMR: δ = 77.0 ppm), d_6-DMSO (^1H NMR: δ = 2.50 ppm, ^{13}C NMR: δ = 39.52 ppm), CD_3CN (^1H NMR: δ = 1.94 ppm, ^{13}C NMR: δ = 1.32, 118.26 ppm) or D_2O (^1H NMR: δ = 4.79 ppm).[3] Data for ^1H NMR spectra were reported as follows: chemical shift (ppm), peak shape (s = singlet, d = doublet, t = triplet, q = quartet, m = multiplet, dd = doublet of doublets, vt = virtual triplet), coupling constant (Hz), and integration. Data for ^{13}C NMR were reported in terms of chemical shift (ppm). Manganese complexes **Mn-1**,[4] **Mn-2**,[5] **Mn-3**,[6] **Mn-4**,[7] **Mn-6**,[4] were prepared by our reported methods.

2. General Procedure for Manganese Pincer Catalyzed Hydration and *α*-Deuteration of Organonitriles

2.1 Description of the reaction setup

Hydration and *α*-deuteration of reactions were performed in a 100 mL Schlenk tube equipped with a magnetic stirring bar in a closed system. After adding all the reactants into the Schlenk tube in a glovebox, the reaction mixture was stirred at the specified temperatures (oil bath temperature).

The sideview of the reaction tube is shown in Figure S1.

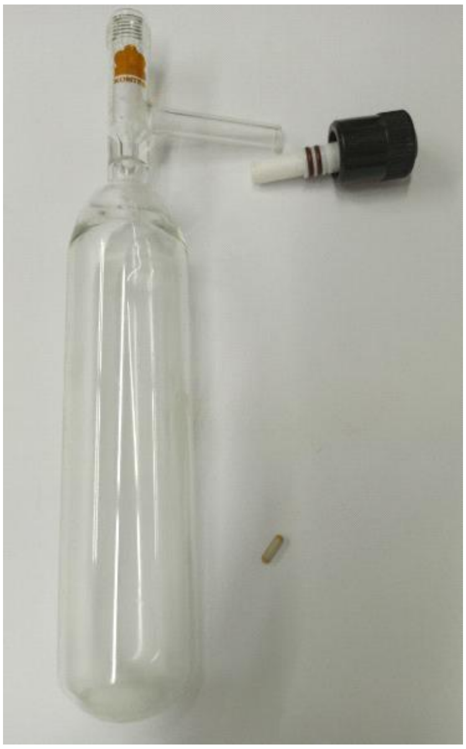

Figure S1. Sideview of the reaction tubes.

2.2 General procedure for hydration reaction of organonitriles

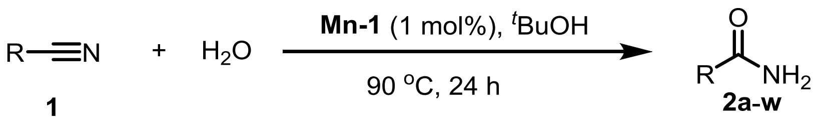

In a glovebox, to a 100 mL Schlenk tube charged with a stirring bar the manganese pincer complex **Mn-1** (0.01 mmol, 1 mol%) was added. Liquid nitriles **1** (1.0 mmol, 1.0 eq.) were added with a glass pipette in to the tube (solid nitriles **1** were added together with the catalyst at the first step). Degassed ^{t}BuOH (2 × 0.5 mL) was transferred into the above Schlenk tube using the same glass pipette and H_2O (5.0 mmol, 90.0 mg, 5.0 eq.) was added. The Schlenk tube was taken out of the glovebox and stirred at 90 ˚C (oil bath) for 24 hours. The reacton mixture was then transferred into a 20 mL vial, the solvent was removed under vacuum and the residue was washed with pentane and dried under vacuum to give the amide product **2**. If the amide product was not pure at that stage, it was purified through a short column (silica gel) to give the pure amide product.

2.3 General procedure for *α*-deuteration of aliphatic nitriles

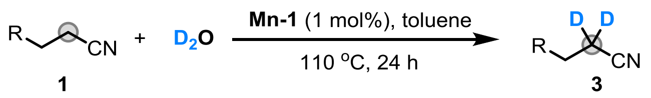

In a glovebox, to a 100 mL Schlenk tube charged with a stirring bar the manganese pincer complex **Mn-1** (0.005 mmol, 1 mol%) was added. Aliphatic nitriles **1** (0.5 mmol, 1.0 eq.) were added with a glass pipette into the tube and degassed toluene (0.5 mL) was added using the same glass pipette. The solution was stirred at room temperature for 10 min, then degassed D_2O (0.5 mL) was added. The Schlenk tube was taken out of the glovebox and stirred at 110 ˚C (oil bath) for 24 hours (substrate **1g** and **1h** reacted at 70 ˚C). The reacton mixture was then extracted with ethyl acetate and the ethyl acetate solution was dried with anhydrous sodium sulfate. Then, the solvent was removed by vacuum to afford pure *α*-deuterated aliphatic nitrile products **3**. The α-deuteration level was calculated from ^{1}H NMR, and the structure was further confirmed by ^{2}H NMR and ^{13}C{^{1}H} NMR.

2.4 General procedure for one pot *α*-deuteration and hydration of nitrile

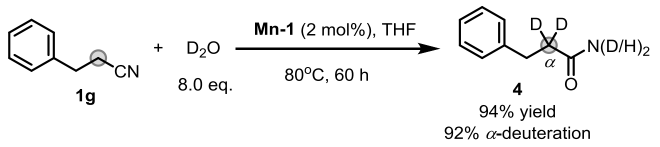

In a glovebox, to a 100 mL Schlenk tube charged with a stirring bar was added the manganese pincer complex **Mn-1** (0.01 mmol, 2 mol%). 3-phenylpropanenitrile **1g** (0.5 mmol, 65.5 mg, 1.0 eq.) was added with a glass pipette into the tube, followed by degassed THF (0.5 mL) using the same glass pipette and D_2O (4.0 mmol, 8.0 eq.). The Schlenk tube was taken out of the glovebox and stirred at 90 ˚C (oil bath) for 60 hours. The reacton mixture was then transferred into a 20 mL vial, the solvent was removed under vacuum and the residue was purified using a short column (silica gel) to give the amide product **4** as a white solid (94% yield, 92% *α*-deuteration). Under this same condition, deuteration of the 3-phenylpropanamide was tested, no α-deuterated product was detected after the reaction. This implies that in the one pot reaction α-deuteration precedes the hydration reaction.

3. Synthetic Applications and Mechanistic Studies

3.1 Procedure for gram-scale reaction of 1a

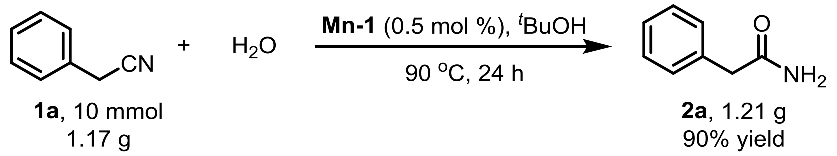

In a glovebox, to a 100 mL Schlenk tube charged with a stirring bar was added the manganese pincer complex **Mn-1** (0.05 mmol, 0.5 mol%). Benzyl nitrile **1a** (10.0 mmol, 1.17g, 1.0 eq.) was added with a glass pipette in to the tube, followed by degassed ^{t}BuOH (2 × 2.5 mL) using the same glass pipette and H_2O (50.0 mmol, 900.0 mg, 5.0 eq.). The Schlenk tube was taken out of the glovebox and stirred at 90 ˚C (oil bath) for 24 hours. The reacton mixture was transferred into a 20 mL vial, and the solvent was removed under vacuum. The precipitated amide was washed with pentane and dried under vacuum to give 1.21 g of the amide product **2a** (90% yield, white solid).

3.2 Procedure for transformation of primary amide products

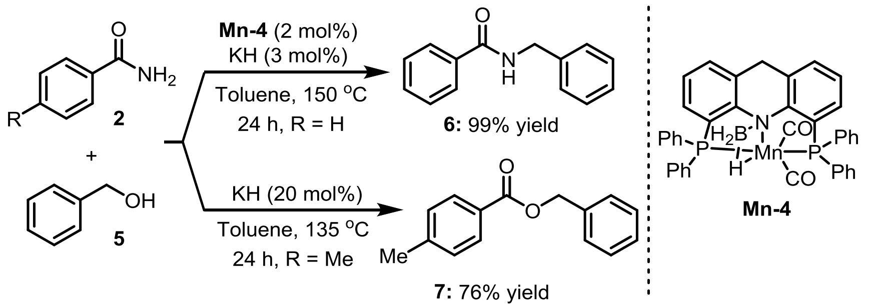

The procedure of transformation of primary amide to secondary amide using **Mn-4** was performed according to our previous reported procedure.[7]

General procedure for the alcoholysis of primary amide to ester: In a glovebox, to a 100 mL Schlenk tube charged with a stirring bar was added 4-methylbenzamide (1.0 mmol, 135.1 mg, 1.0 eq.) and a catalytic amount of KH (0.2 mmol, 8.0 mg, 20 mol%). Degassed toluene (0.5 mL) was then transferred into the same Schlenk tube, and phenylmethanol **5** (2.0 mmol, 216.1 mg) was

added with a glass pipette in to the tube, followed by degassed toluene (2 × 0.5 mL) using the same glass pipette. The Schlenk tube was taken out of the glovebox and stirred at 135 ˚C (oil bath) for 24 hours. Upon the completion of the reaction (24 h), the reacton mixture was analyzed by GC using biphenyl as an internal standard forming benzyl 4-methylbenzoate **7** in 76% yield.

3.3 Synthesis of complexes Mn-5 and Mn-7

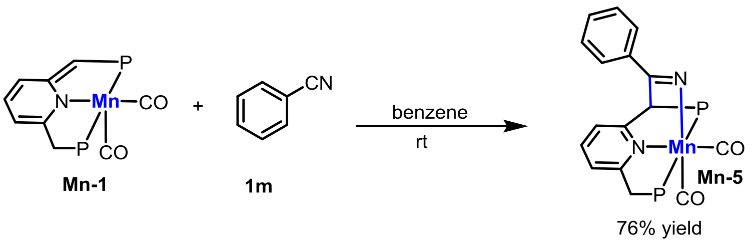

General procedure: In a glovebox, **Mn-1** (20.2 mg, 0.04 mmol) was suspended in benzene (0.5 mL) in a 20.0 mL vial charged with a stirring bar. Benzonitrile **1m** (20.6 mg, 0.20 mmol) was added to the stirred solution. After stirring at room temperature for 1 h, the system color turned from dark blue to brownish red. Half of the solvent was removed under vacuum, n-pentane (6 mL) was added, the solution was kept at −38 °C in a freezer for 48 h to form brownish red crystals. The crystals were decanted and dried under vacuum with a 76% yield.

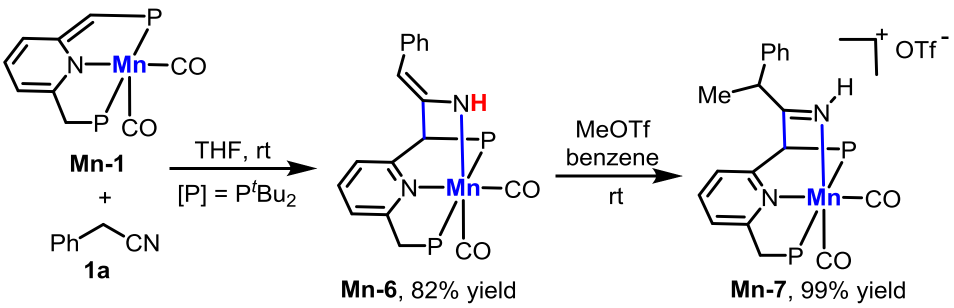

The reaction of complex **Mn-1** with benzyl nitrile **1a** to form complex **Mn-6** was performed according to our previous reported procedure.[4]

General procedure for the reaction of the enamido complex **Mn-6** with methyl triflate: in a glovebox, **Mn-6** (24.9 mg, 0.04 mmol) was suspended in benzene (1.0 mL) in a 20 mL vial charged with a stirring bar. Methyl triflate (52.5 mg, 0.32 mmol) was added to the stirred solution. After stirring at room temperature for 20 min, the system color turns from brownish red to yellow. n-Pentane was directly added to the mixture to precipitate a yellow solid, which was separated by

decantation and dried under reduced pressure to yield cationic imine complex **Mn-7** in 99% yield.

3.4 NMR Studies of benzyl nitrile 1a binding to the dearomatized manganese pincer complex Mn-1 in toluene

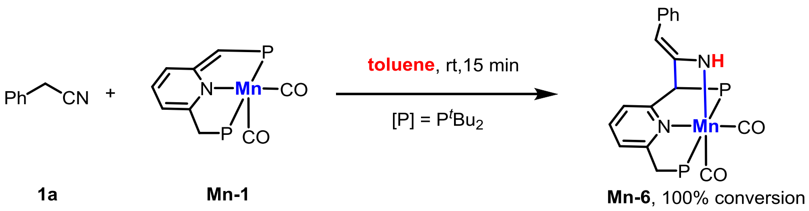

General procedure: In a glovebox, **Mn-1** (20.2 mg, 0.04 mmol) was suspended in toluene (1.0 mL) in a 20.0 mL vial charged with a stirring bar. Benzyl nitrile **1a** (0.2 mmol, 23.4 mg, 5.0 eq.) was added to the stirred solution. After stirring at room temperature for 15 min, the system color turned from dark blue to brownish red. The resulting solution was transferred into a J. Young valve NMR tube and the $^{31}P\{^1H\}$ NMR measured directly, indicating that the product is **Mn-6** which consistent with our previous literature report.[4]

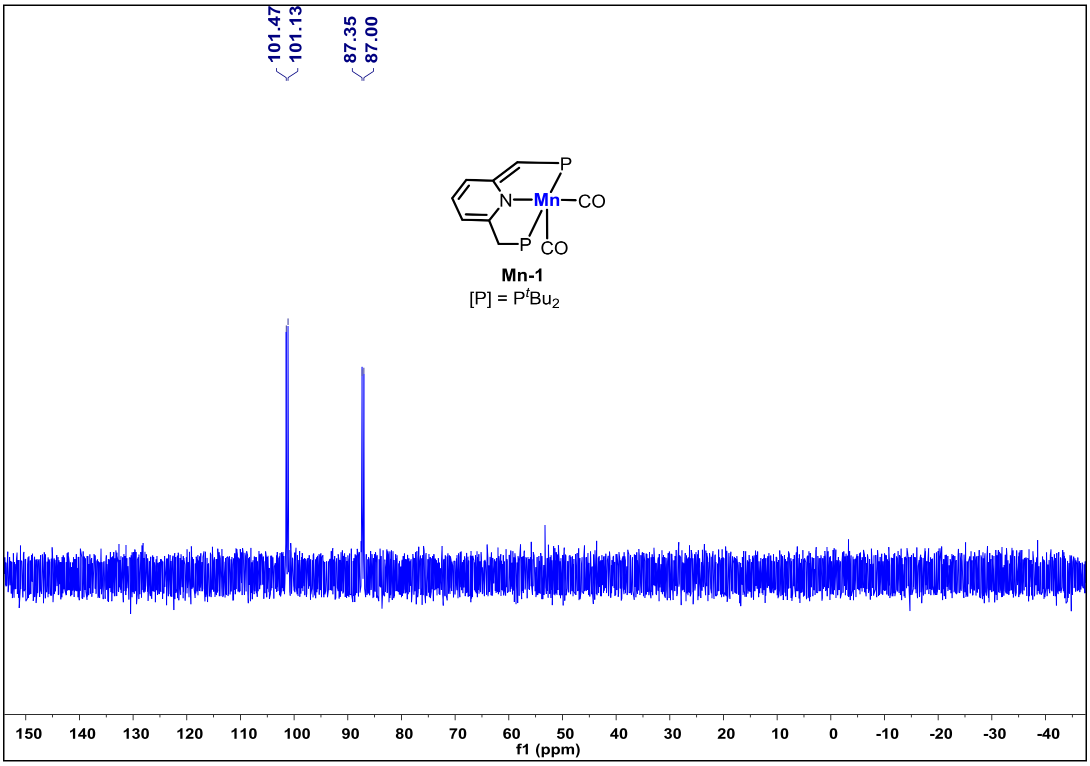

Figure S2. $^{31}P\{^1H\}$NMR spectrum (121 MHz, THF, 298 K, **Mn-1** in toluene)

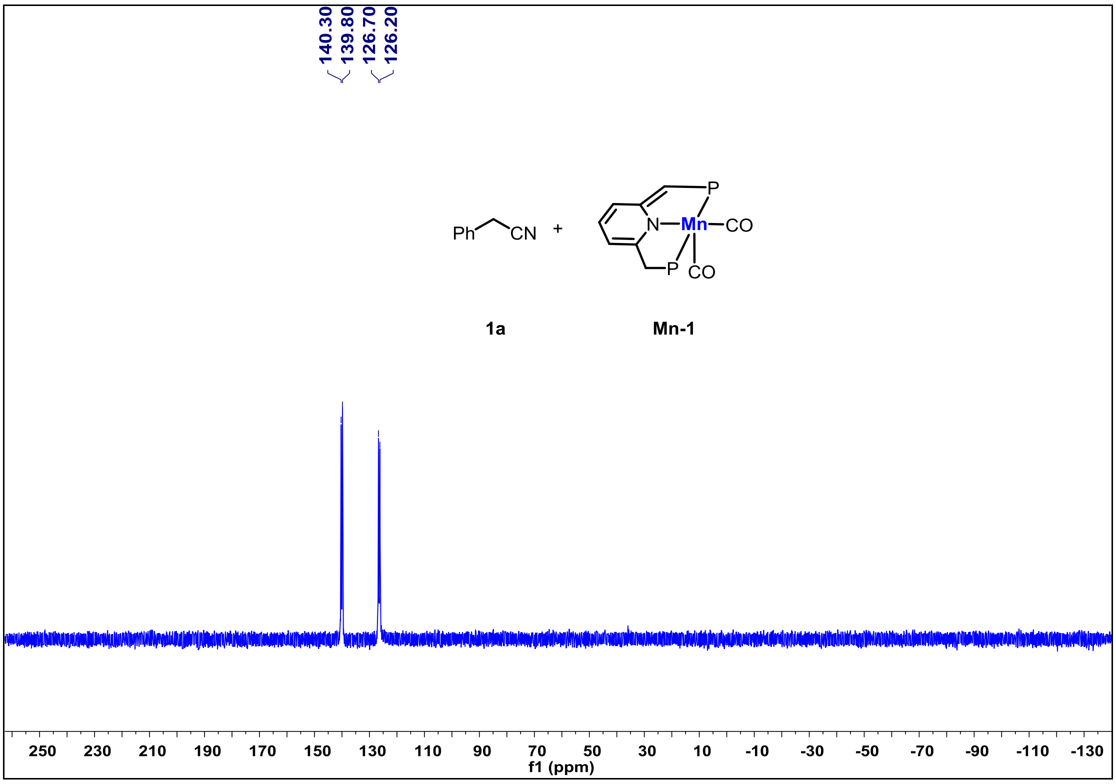

Figure S3. $^{31}P\{^{1}H\}$NMR spectrum (121 MHz, THF, 298 K, **Mn-1** in toluene, added 5.0 eq. benzyl nitrile **1a**)

3.5 The interconversion between the hydroxomanganase A and the enamido intermediate B

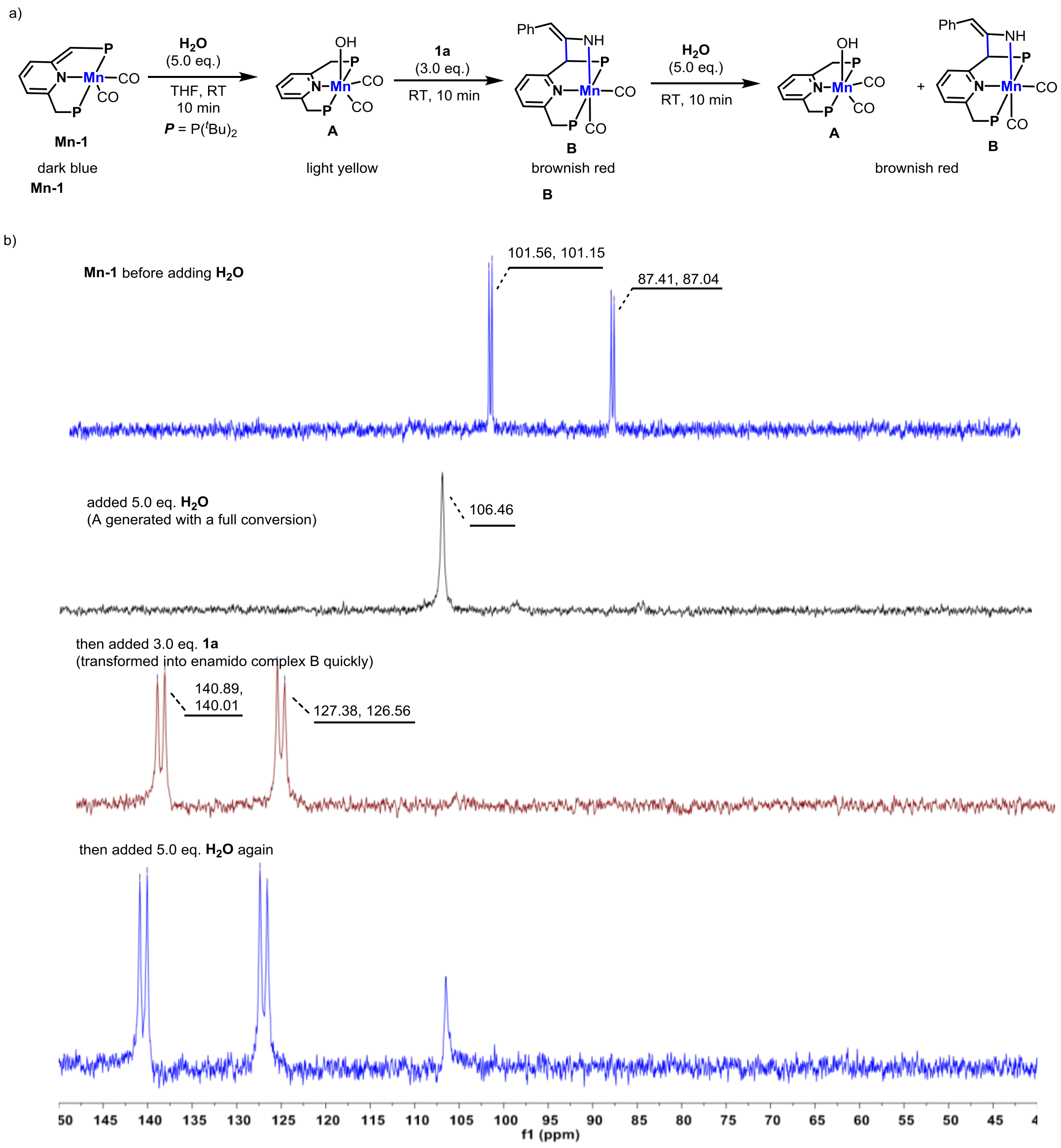

Figure S4. Dynamic equilibria of dearomatized manganese **Mn-1**, nitrile **1a** and water ($^{31}P\{^1H\}$NMR spectrum, 121 MHz, THF, 298 K)

General procedure: 1) in a glovebox, **Mn-1** (10.1 mg, 0.02 mmol) were suspended in THF (0.5 mL) in a 5.0 mL vial. H_2O (1.8 mg, 0.1 mmol, 5.0 eq.) was added with a glass pipette in to vial. After stirring at room temperature for 10 min, the color changes from dark blue to light yellow, the resulting solution was transferred into a J. Young valve NMR tube and the $^{31}P\{^1H\}$ NMR measured directly (Fig S4). 2) Then take the J. Young valve NMR tube back to glovebox, benzyl

nitrile **1a** (7.0 mg, 0.06 mmol, 3.0 eq.) was added, and stirring at room temperature for 10 min, and measure the $^{31}P\{^1H\}$ NMR directly. 3) Again, take the J. Young valve NMR tube back to glovebox, H_2O (1.8 mg, 0.1 mmol, 5.0 eq.) was added, stirring at room temperature for 10 min and measure the $^{31}P\{^1H\}$ NMR directly.

Analyze from $^{31}P\{^1H\}$ NMR data, the interconversion between the hydroxomanganase **A** and the enamido intermediate **B** can be clearly observed (see Fig S4). Addition of 5.0 eq. water to a THF solution of dearomatized complex **Mn-1** resulted in the formation of the hydroxomanganase complex A with a singlet appearing at δ = 106.46 ppm in the $^{31}P\{^1H\}$ NMR spectrum. Upon treatment with 3.0 eq. benzyl nitrile **1a**, hydroxomanganase **A** completely transformed into enamido complex **B** quickly. Addition of 5 more equivalents of water to the solution regenerates the hydroxo complex signifying the competitive binding of water and nitriles to **Mn-1** via MLC.

3.6 Catalytic performance of the isolated ketimido manganese complex Mn-5 and enamido complex Mn-6 in the hydration and α-deuteration reactions

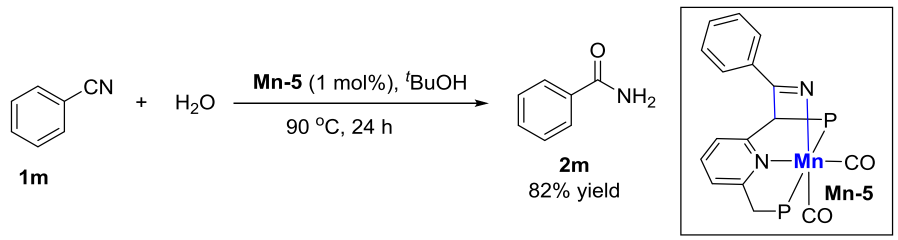

General procedure: in a glovebox, to a 100 mL Schlenk tube charged with a stirring bar was added the manganese pincer complex **Mn-5** (0.005 mmol, 1 mol%). Benzonitrile **1m** (51.5 mg, 0.50 mmol) was added with a glass pipette in to the tube, followed by degassed *ᵗ*BuOH (2 × 0.3 mL) using the same glass pipette, and H_2O (2.5 mmol, 45.0 mg, 5.0 eq.) was added. The Schlenk tube was taken out of the glovebox and stirred at 90 ˚C (oil bath) for 24 hours. The reacton mixture was then transferred to a 20 mL vial and the solvent was removed under vacuum and the precipitated amide was washed with pentane and dried under vacuum to give the amide product **2m** in 82% yield.

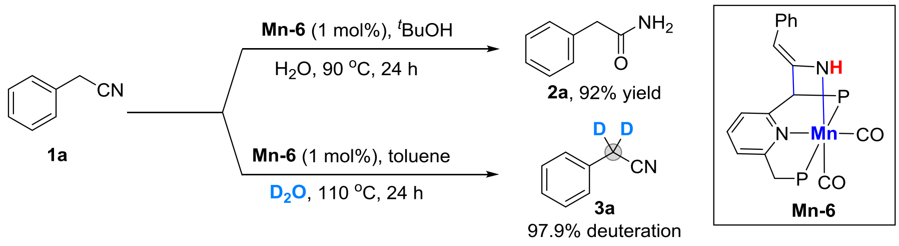

General procedure for hydration and α-deuteration: the procedure is same as with the hydration and α-deuteration procedure using **Mn-1** as catalyst.

3.7 ^{31}P NMR spectra of complexes for monitoring the reaction conversion process

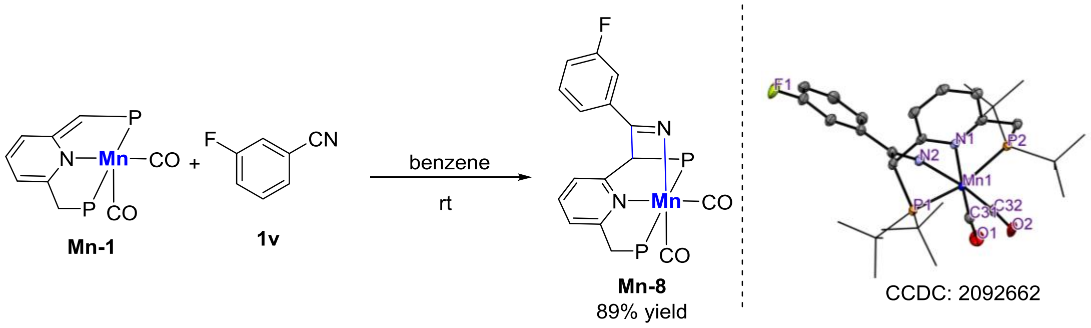

General procedure for stoichiometric reaction between **Mn-1** and 3-fluorobenzonitrile: In a glovebox, **Mn-1** (20.2 mg, 0.04 mmol) was suspended in benzene (0.5 mL) in a 20.0 mL vial charged with a stirring bar. 3-F-Benzonitrile **1v** (0.16 mmol) was added to the stirred solution. After stirring at room temperature for 1 h, the system color turned from dark blue to brownish red. Half of the solvent was removed under vacuum, n-pentane (6 mL) was added, and the solution was kept at −38 ° C in a freezer for 48 h to form brownish red crystals. The crystals were decanted and dried under vacuum to give of complex Mn-8 in 89% yield.

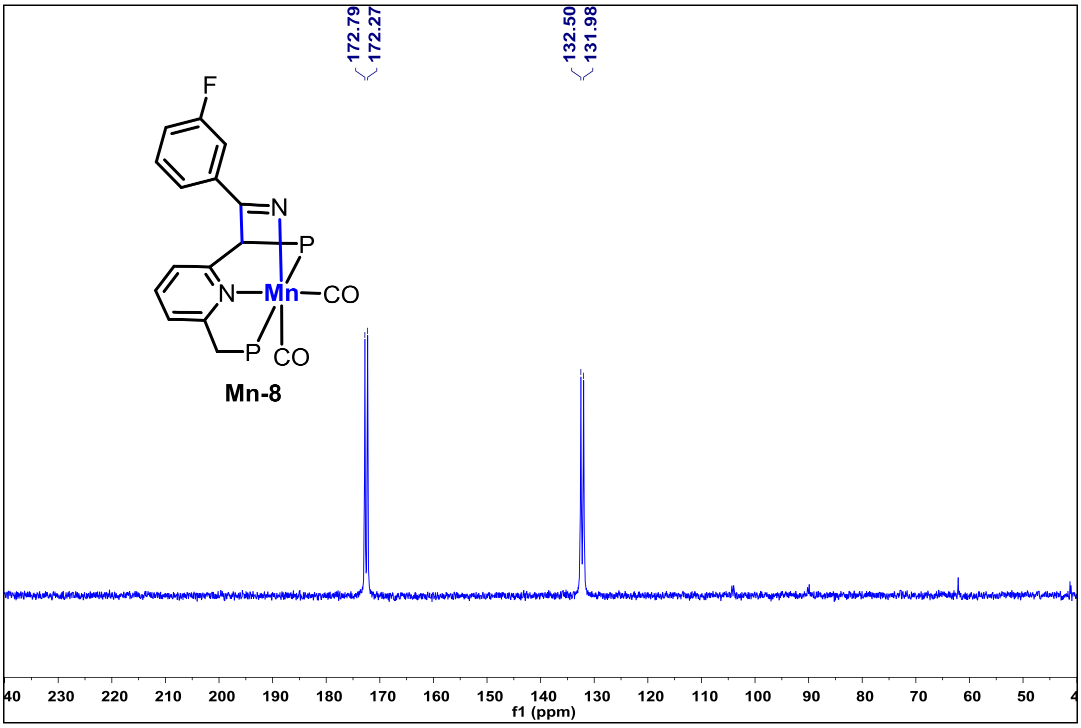

Figure S5. ^{31}P NMR spectrum of **Mn-8** (202 MHz, CD_3CN, 298 K).

Based on the structurecharacterization of **Mn-8**, we also do performed some experiments to monitor the reaction conversion process using 3-fluorobenzonitrile as substrate (see Figure S6 below).

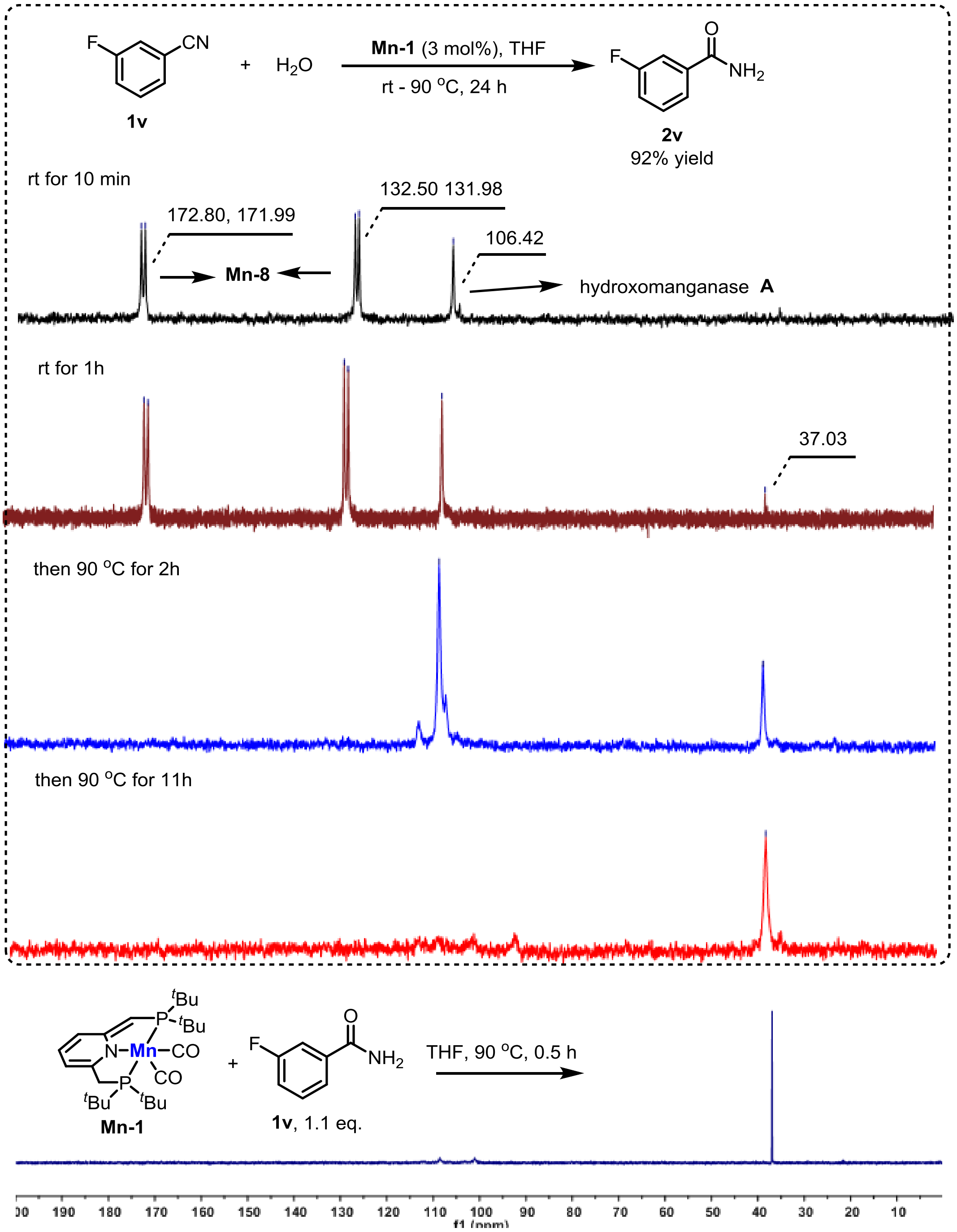

Figure S6. ^{31}P NMR spectra of complexes for monitoring the reaction conversion process.

When the reaction was just established and stirred at room temperature for 10 minutes, the ketimido manganese complex was detected in the reaction system as the main intermediate, the hydroxomanganase complex A was also detected exhibiting a singlet appearing at δ = 106.46 ppm in the ^{31}P NMR spectrum. After stirring at room temperature for 1 hour, hydroxomanganase complex A gradually increased and manganese-amide addition product with a singlet at δ = 37.03

ppm appears. Then upon heating the reaction at 90 oC for 2 hours, the ketimido manganese complex slowly disappeared, and the hydroxomanganase complex **A** and the manganese-amide addition intermediate (δ = 37.03 ppm in the ^{31}P NMR spectrum) gradually become the main products. Then heating at 90 oC for additional 11 hours, hydroxomanganase complex **A** also disappeared, and only the manganese-amide addition intermediate was detected, after 24 hours, the amide product **2v** was isolated in 92% yield.

3.8 Test base catalytic α-deuteration reactions

Table S1. Base catalyzed α-deuteration reaction of nitrile **1g**

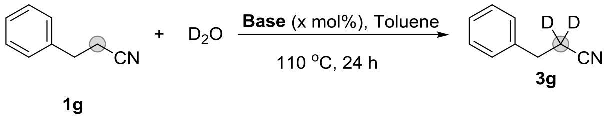

entry[a]	Base	x mol%	Deuteration (%) [b]
1	NaOH	10 mol%	4
2	K_2CO_3	10 mol%	0
3	^{t}BuOK	10 mol%	8
4	CsF	10 mol%	0
5	--	--	0

[a]Reaction conditions: nitrile (0.50 mmol), **Base** (x mol%), D_2O (0.5 mL) and toluene (0.5 mL) at 110 °C (bath temperature) for 24 hours. [b]After the reaction was complete, extraction with ethyl acetate and removing the solvent, the degree of *α*-deuteration was calculated from **^{1}H NMR**.

Both our hydration and deuteration reactions proceeded under neutral conditions without the addition of any catalytic base. In addition, we have tried a variety of bases under the similar conditions of α-deuteration reaction of nitrile **1g** without Mn catalyst and only minimal

deuteration was observed (please note that the deuteration reaction is likely biphasic and the effective base concentration in toluene is significantly lower than otherwise would have been obtained by the added base).

4. Characterization Data of the Synthesized Compounds

4.1 Characterization data of amides and synthesized manganese complex

2-Phenylacetamide (2a)

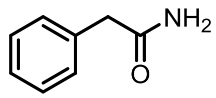

Yield: 97% (white solid, 131.0 mg).

^{1}H NMR (400 MHz, DMSO-d6, 298 K) δ (ppm) = 7.47 (s, 1H), 7.33 – 7.10 (m, 5H), 6.86 (s, 1H), 3.36 (s, 2H).

^{13}C{^{1}H} NMR (100 MHz, DMSO-d6, 298 K) δ (ppm) = 172.41, 136.51, 129.10, 128.22, 126.35, 42.31.

Know compound, see: Li, Z.; Wang, L.; Zhou, X. *Adv. Synth. Catal.* **2012**, *354*, 584-588.

2-(3-Bromophenyl)acetamide (2b)

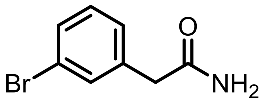

Yield: 92% (white solid, 195.1 mg).

^{1}H NMR (400 MHz, DMSO-d6, 298 K) δ (ppm) = 7.47 (s, 1H), 7.44– 7.42 (m, 1H),

7.39-7.36 (m, 1H), 7.24 – 7.21 (m, 2H), 6.89 (s, 1H), 3.34 (s, 2H).

^{13}C{^{1}H} NMR (100 MHz, DMSO-d6, 298 K) δ (ppm) = 171.63, 139.19, 131.77, 130.25, 129.13, 128.20, 121.34, 41.57.

Know compound, see: Chen, H.; Dai, W.; Chen, Y.; Xu, Q.; Chen, J.; Yu, L.; Zhao, Y.; Ye, M.; Pan, Y. *Green Chem.* **2014**, *16*, 2136−2141.

2-(m-Tolyl)acetamide (2c)

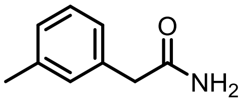

Yield: 94% (white solid, 140.3 mg).

^{1}H NMR (500 MHz, DMSO-d6, 298 K) δ (ppm) = 7.42 (s, 1H), 7.19-7.15 (m, 1H), 7.07-7.01 (m, 3H), 6.84 (s, 1H), 3.31 (s, 2H), 2.27 (s, 3H).

^{13}C{^{1}H} NMR (125 MHz, DMSO-d6, 298 K) δ (ppm) = 172.23, 137.09, 136.34, 129.66, 128.02, 126.86, 126.09, 42.22, 20.97.

Know compound, see: Martina, F; Manuel, A; Francesco, A; Andrea, M; Giorgio, S; Andrea,C; Roccaldo,S; Antonio, M; Emidio, C; Paride, L. *Bioorg Med Chem.,* **2015** *23*, 4860-4865.

2-(4-Fluorophenyl)acetamide (2d)

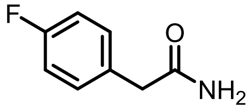

Yield: 99% (white solid, 150.7 mg).

¹H NMR (500 MHz, $CDCl_3$, 298 K) δ (ppm) = 7.07 (s, 2H), 6.87 (d, J = 8.6 Hz, 2H), 5.34 (d, *J* = 101.7 Hz, 2H), 3.38 (s, 2H).

¹³C{¹H} NMR (125 MHz, DMSO-d6, 298 K) δ (ppm) = 172.14, 160.96 (*J* = 203.6 Hz), 132.63, 130.85 (*J* = 8.1 Hz), 114.80 (*J* = 21.1 Hz), 41.21.

Know compound, see: Szostak, M.; Spain, M.; Eberhart, A. J.; Procter, D. J. *J. Am. Chem. Soc.* **2014**, *136*, 2268-2271.

2-(4-Methoxyphenyl)acetamide (2e)

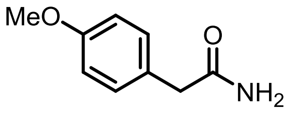

Yield: 99% (white solid, 163.9 mg).

¹H NMR (500 MHz, DMSO-d6, 298 K) δ (ppm) = 7.39 (s, 1H), 7.17 (d, J = 7.9 Hz, 2H), 6.85 (d, J = 8.7 Hz, 3H), 3.72 (s, 3H), 3.29 (s, 2H).

¹³C{¹H} NMR (125 MHz, DMSO-d6, 298 K) δ (ppm) = 172.63, 157.83, 130.01, 128.43, 113.58, 55.00, 41.36.

Know compound, see: Chen, H.; Dai, W.; Chen, Y.; Xu, Q.; Chen, J.; Yu, L.; Zhao, Y.; Ye, M.; Pan, Y. *Green Chem.* **2014**, *16*, 2136−2141.

2-(Pyridin-3-yl)acetamide (2f)

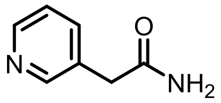

Yield: 55% (white solid, 74.1 mg).

^{1}H NMR (400 MHz, $CDCl_3$, 298 K) δ (ppm) = 8.66 (s, 2H), 7.92 – 7.65 (m, 1H), 7.42 (s, 1H), 5.98 (d, *J* = 64.0 Hz, 2H), 3.69 (s, 2H).

^{13}C{^{1}H} NMR (100 MHz, $CDCl_3$, 298 K) δ (ppm) = 172.47, 150.37, 148.77, 137.04, 130.75, 123.87, 40.04.

3-Phenylpropanamide (2g)

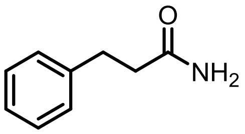

Yield: 95% (white solid, 141.3 mg).

^{1}H NMR (400 MHz, DMSO-d6, 298 K) δ (ppm) =7.30 – 6.75 (m, 6H), 6.75 (s, 1H), 2.79 (t, *J* = 7.8 Hz, 2H), 2.35 (t, *J* = 7.8 Hz, 2H).

^{13}C{^{1}H} NMR (100 MHz, DMSO-d6, 298 K) δ (ppm) = 173.54, 141.49, 128.28, 128.23, 125.86, 36.72, 30.92.

4-Phenylbutanamide (2h)

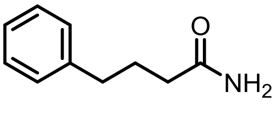

Yield: 92% (white solid, 149.6 mg).

^{1}H NMR (400 MHz, DMSO-d6, 298 K) δ (ppm) = 7.37 (dd, *J* = 8.2, 6.8 Hz, 3H), 7.28 (d, *J* = 7.5 Hz, 3H), 6.82 (s, 1H), 2.65 (t, *J* = 7.7 Hz, 2H), 2.16 (t, *J* = 7.5 Hz, 2H), 1.91 – 1.84 (m, 2H).

^{13}C{^{1}H} NMR (100 MHz, DMSO-d6, 298 K) δ (ppm) = 174.03, 141.81, 128.29,

128.25, 125.71, 34.69, 34.53, 26.88.

Acetamide (2i)

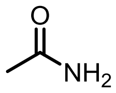

Yield: 55% (white solid, 32.6 mg).

^{1}H NMR (400 MHz, DMSO-d6, 298 K) δ (ppm) = 7.28 (s, 1H), 6.68 (s, 1H), 1.75 (s, 3H).

^{13}C{^{1}H} NMR (100 MHz, DMSO-d6, 298 K) δ (ppm) = 171.58, 22.52.

Know compound, see: Li, Z.; Wang, L.; Zhou, X. *Adv. Synth. Catal.* **2012**, *354*, 584-588.

Propionamide (2j)

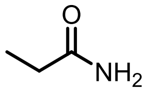

Yield: 71% (white solid, 51.5 mg).

^{1}H NMR (400 MHz, DMSO-d6, 298 K) δ (ppm) = 7.23 (s, 1H), 6.68 (s, 1H), 2.04 (q, J = 7.6 Hz, 2H), 0.97 (t, J = 7.5 Hz, 3H).

^{13}C{^{1}H} NMR (125 MHz, DMSO-d6, 298 K) δ (ppm) = 175.15, 28.22, 9.79.

Know compound, see: Coeck, R; Berden, S; De Vos, D. *Green Chem.* **2014**, *16*, 2136−2141.

Butyramide (2k)

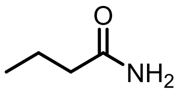

Yield: 89% (white solid, 77.4 mg).

^{1}H NMR (400 MHz, DMSO-d6, 298 K) δ (ppm) = 7.23 (s, 1H), 6.68 (s, 1H), 2.01 (t, *J* = 7.3 Hz, 2H), 1.49 (q, *J* = 7.3 Hz, 2H), 0.85 (t, *J* = 7.3 Hz, 3H).

^{13}C{^{1}H} NMR (125 MHz, DMSO-d6, 298 K) δ (ppm) = 174.13, 37.07, 18.47, 13.67.

Know compound, see: Singh, K.; Sarbajna, A.; Dutta, I.; Pandey, P.; Bera, J. K. *Chem. - Eur. J.* **2017**, *23*, 7761-7771.

Pentanamide (2l)

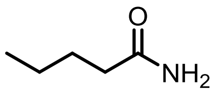

Yield: 86% (white solid, 86.7 mg).

^{1}H NMR (400 MHz, DMSO-d6, 298 K) δ (ppm) = 6.95 (s, 1H), 6.66 (s, 1H), 2.02 (t, *J* = 7.4 Hz, 2H), 1.48 – 1.40 (m, 2H), 1.29 – 1.20 (m, 2H), 0.85 (t, *J* = 7.3 Hz, 3H).

^{13}C{^{1}H} NMR (100 MHz, DMSO-d6, 298 K) δ (ppm) = 174.60, 34.89, 27.33, 21.90, 13.80.

Know compound, see: Das, R.; Chakraborty, D. *Catal. Commun.* **2012**, *26*, 48-53.

Benzamide (2m)

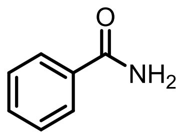

Yield: 85% (white solid, 102.9 mg).

^{1}**H NMR** (400 MHz, DMSO-d6, 298 K) δ (ppm) = 7.98 (s, 1H), 7.90 – 7.83 (m, 2H), 7.56 – 7.48 (m, 1H), 7.47 – 7.44 (m, 2H), 7.35 (s, 1H).

^{13}**C{^{1}H} NMR** (100 MHz, DMSO-d6, 298 K) δ (ppm) = 168.09, 134.27, 131.35, 128.31, 127.52.

Know compound, see: Li, Z.; Wang, L.; Zhou, X. *Adv. Synth. Catal.* **2012**, *354*, 584-588.

4-Fluorobenzamide (2n)

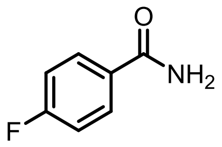

Yield: 96% (white solid, 133.7 mg).

^{1}**H NMR** (400 MHz, DMSO-d6, 298 K) δ (ppm) = 7.99 (s, 1H), 7.96 – 7.90 (m, 2H), 7.39 (s, 1H), 7.33 – 7.18 (m, 2H).

^{13}**C{^{1}H} NMR** (100 MHz, DMSO-d6, 298 K) δ (ppm) = 166.78, 163.90 (d, *J* = 246.6 Hz), 130.73 (d, *J* = 2.9 Hz), 130.09 (d, *J* = 8.9 Hz), 115.07 (d, *J* =21.6 Hz).

Know compound, see: Tao, T.; Wang, Z.; Liu, Z.; Feng, X.; Wang, Q. *Green. Chem.* **2012**, *14*, 921-924.

4-bromobenzamide (2o)

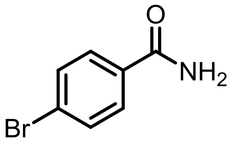

Yield: 88% (white solid, 175.1 mg).

^{1}H NMR (500 MHz, DMSO-d6, 298 K) δ (ppm) = 8.05 (s, 1H), 7.82 (d, *J* = 8.0 Hz, 2H), 7.67 (d, *J* = 8.0 Hz, 2H), 7.46 (s, 1H).

^{13}C{^{1}H} NMR (125 MHz, DMSO-d6, 298 K) δ (ppm) = 167.00, 133.41, 131.27, 129.63, 125.05.

Know compound: Tao, T.; Wang, Z.; Liu, Z.; Feng, X.; Wang, Q. *Green. Chem.* **2012**, *14*, 921-924.

4-Chlorobenzamide (2p)

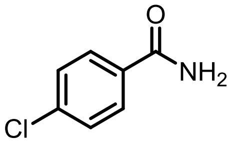

Yield: 91% (white solid, 141.1 mg).

^{1}H NMR (400 MHz, DMSO-d6, 298 K) δ (ppm) = 8.13 (s, 1H), 7.97 (d, *J* = 8.2 Hz, 2H), 7.60 (d, *J* = 8.0 Hz, 2H), 7.54 (s, 1H).

^{13}C{^{1}H} NMR (100 MHz, DMSO-d6, 298 K) δ (ppm) = 166.90, 136.13, 133.05, 129.44, 128.34.

Know compound: Li, Z.; Wang, L.; Zhou, X. *Adv. Synth. Catal.* **2012**, *354*, 584-588.

4-Nitrobenzamide (2q)

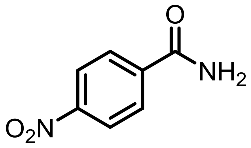

Yield: 95% (white solid, 157.7 mg).

^{1}H NMR (500 MHz, DMSO-d6, 298 K) δ (ppm) = 8.30 – 8.27 (m, 3H), 8.09 (d, J = 8.4 Hz, 2H), 7.72 (s, 1H).

^{13}C{^{1}H} NMR (125 MHz, DMSO-d6, 298 K) δ (ppm) = 165.70, 147.81, 135.78, 133.80, 130.07, 125.89, 122.22.

Know compound, see: Singh, K.; Sarbajna, A.; Dutta, I.; Pandey, P.; Bera, J. K. *Chem. - Eur. J.* **2017**, *23*, 7761-7771.

4-Methylbenzamide (2r)

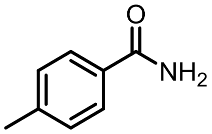

Yield: 53% (white solid, 71.5 mg).

^{1}H NMR (400 MHz, DMSO-d6, 298 K) δ (ppm) = 7.88 (s, 1H), 7.80 – 7.73 (m, 2H), 7.24 (d, J = 7.8 Hz, 3H), 2.34 (s, 3H).

^{13}C{^{1}H} NMR (100 MHz, DMSO-d6, 298 K) δ (ppm) = 167.76, 141.03, 131.47, 128.71, 127.48, 20.94.

Know compound: Li, Z.; Wang, L.; Zhou, X. *Adv. Synth. Catal.* **2012**, *354*, 584-588.

4-Methoxybenzamide (2s)

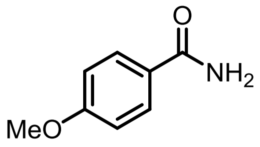

Yield: 61% (white solid, 91.9 mg).

^{1}**H NMR** (500 MHz, DMSO-d6, 298 K) δ (ppm) = 7.85-7.81 (m, 3H), 7.17 (s, 1H), 6.97 (d, *J* = 8.4 Hz, 2H), 3.80 (s, 3H).

^{13}**C{^{1}H} NMR** (125 MHz, DMSO-d6, 298 K) δ (ppm) = 167.46, 161.58, 129.36, 126.51, 113.39, 55.31.

Know compound, see: Zhang, S.; Xu, H.; Lou, C.; Senan, A. M.; Chen, Z.; Yin, G. *Eur. J. Org. Chem.* **2017**, *2017*, 1870−1875.

4-Acetylbenzamide (2t)

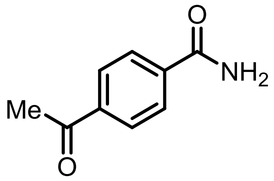

Yield: 73% (white solid, 119.1 mg).

^{1}**H NMR** (400 MHz, DMSO-d6, 298 K) δ (ppm) = 8.10 (s, 1H), 7.99-7.95 (m, 4H), 7.47 (s, 1H), 2.59 (s, 3H).

^{13}**C{^{1}H} NMR** (100 MHz, DMSO-d6, 298 K) δ (ppm) = 197.29, 167.28, 138.55, 138.00, 127.89, 127.71, 26.78.

3-Nitrobenzamide (2u)

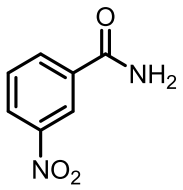

Yield: 86% (white solid, 142.9 mg).

^{1}H NMR (400 MHz, DMSO-d6, 298 K) δ (ppm) = 8.69 (s, 1H), 8.42 – 8.27 (m, 3H), 7.79 – 7.75 (m, 1H), 7.71 (s, 1H).

^{13}C{^{1}H} NMR (100 MHz, DMSO-d6, 298 K) δ (ppm) = 165.70, 147.81, 135.78, 133.80, 130.07, 125.89, 122.22.

Know compound, see: Li, Z.; Wang, L.; Zhou, X. *Adv. Synth. Catal.* **2012**, *354*, 584-588.

3-Fluorobenzamide (2v)

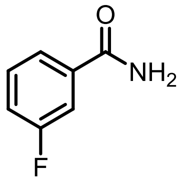

Yield: 97% (white solid, 134.6 mg).

^{1}H NMR (500 MHz, DMSO-d6, 298 K) δ (ppm) = 8.06 (s, 1H), 7.76 – 7.70 (m, 1H), 7.66 (d, *J* = 10.3 Hz, 1H), 7.58 – 7.46 (m, 2H), 7.37 (d, *J* = 9.3 Hz, 1H).

^{13}C{^{1}H} NMR (125 MHz, DMSO-d6, 298 K) δ (ppm) = 161.96 (*J* = 242.5 Hz), 136.70 (*J* = 6.6 Hz), 130.38 (*J* = 8.0 Hz), 123.60 (*J* = 2.7 Hz), 118.11 (*J* = 21.0 Hz), 114.16 (*J* = 22.2 Hz).

Know compound, see: Chen, H.; Dai, W.; Chen, Y.; Xu, Q.; Chen, J.; Yu, L.; Zhao, Y.; Ye, M.; Pan, Y. *Green Chem.* **2014**, *16*, 2136−2141

Furan-2-carboxamide (2w)

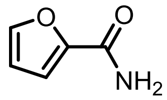

Yield: 72% (white solid, 79.8 mg).

^{1}H NMR (400 MHz, DMSO-d6, 298 K) δ (ppm) = 7.79 (dd, *J* = 1.8, 0.8 Hz, 1H), 7.75 (s, 1H), 7.35 (s, 1H), 7.09 (dd, *J* = 3.4, 0.8 Hz, 1H), 6.59 (dd, *J* = 3.5, 1.8 Hz, 1H).

^{13}C{^{1}H} NMR (100 MHz, DMSO-d6, 298 K) δ (ppm) = 159.38, 148.04, 145.00, 113.59, 111.76.

Know compound, see: Guo, B.; de Vries, J. G.; Otten, E. *Chem. Sci.* **2019**, *10*, 10647−10652.

2-Naphthamide (2x)

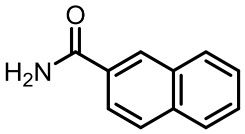

Yield: 94% (white solid, 160.8 mg).

^{1}H NMR (400 MHz, DMSO-d6, 298 K) δ (ppm) = 8.49 (d, *J* = 1.5 Hz, 1H), 8.14 (s, 1H), 8.03 – 7.94 (m, 4H), 7.63 – 7.56 (m, 2H), 7.47 (s, 1H).

^{13}C{^{1}H} NMR (100 MHz, DMSO-d6, 298 K) δ (ppm) = 167.95, 134.16, 132.14,

131.63, 131.60, 128.85, 127.79, 127.75, 127.58, 127.56, 126.64, 124.38.

Know compound, see: Chen, H.; Dai, W.; Chen, Y.; Xu, Q.; Chen, J.; Yu, L.; Zhao, Y.; Ye, M.; Pan, Y. *Green Chem.* **2014**, *16*, 2136−2141.

[Mn(PNPtBu–HN=CCH(Me)Ph)(CO)2]OTf (Mn-7)

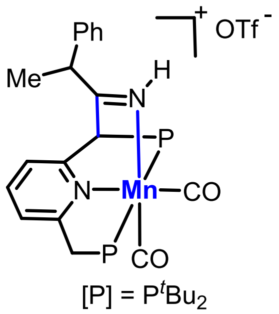

^{1}H NMR (500 MHz, CD_3CN, 298 K) δ (ppm) = 10.04 (s, 1H), 7.85 (dt, J = 14.2, 7.6 Hz, 2H), 7.52 (d, J = 7.4 Hz, 1H), 7.48 – 7.36 (m, 3H), 7.18 – 7.12 (m, 2H), 5.64 (dt, J = 8.5, 2.0 Hz, 1H), 5.47 (s, 1H), 4.45 (q, J = 7.4 Hz, 1H), 3.86 – 3.53 (m, 3H), 1.53 (d, J = 13.0 Hz, 10H), 1.43 (d, J = 12.5 Hz, 9H), 1.11 (d, J = 11.6 Hz, 9H), 0.67 (d, J = 11.7 Hz, 9H).

^{13}C{^{1}H} NMR (125 MHz, CD_3CN, 298 K) δ (ppm) = 188.75 (dd, *J* = 10.0, 3.7 Hz), 165.23 (dd, *J* = 5.5, 2.7 Hz), 158.49 (dd, *J* = 7.8, 5.8 Hz), 139.65 , 137.64 , 130.47 (d, J = 120.2 Hz), 130.27 , 129.43 , 129.37 , 123.39 (d, J = 5.4 Hz), 122.07 (d, J = 6.9 Hz), 110.68 , 58.10 , 55.01 , 50.47 (d, J = 2.4 Hz), 39.60 (d, J = 2.0 Hz), 39.14 – 38.57 (m), 37.87 (d, J = 4.0 Hz), 37.68 (d, J = 15.1 Hz), 31.42 (m), 17.94 .

^{31}P NMR (202 MHz, CD_3CN, 298 K) δ (ppm) = 185.26, 126.20.

^{19}F NMR (470 MHz, CD_3CN, 298 K) δ (ppm) = -80.22.

Similar compound of our previous report, see: Vogt, M.; Nerush, A.; Iron, M. A.;

Leitus, G.; Diskin-Posner, Y.; Shimon, L. J. W.; Ben-David, Y.; Milstein, D. *J. Am. Chem. Soc.* **2013**, *135*, 17004−17018.

4.2 Characterization Data of α- deuterated nitriles

4-Phenylbutanenitrile (1h)

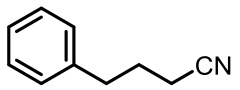

^{1}H NMR (500 MHz, $CDCl_3$, 298 K) δ (ppm) = 7.36 (t, *J* = 7.5 Hz, 2H), 7.28 (t, *J* = 7.3 Hz, 1H), 7.23 (d, *J* = 7.0 Hz, 2H), 2.81 (t, *J* = 7.5 Hz, 2H), 2.34 (t, *J* = 7.1 Hz, 2H), 2.02 (q, *J* = 7.3 Hz, 2H).

^{13}C{^{1}H} NMR (125 MHz, $CDCl_3$, 298 K) δ (ppm) = 139.57, 128.46, 128.26, 126.29, 119.35, 34.17, 26.72, 16.16.

4-Phenylbutanenitrile-2,2-d2 (3h)

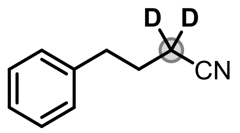

^{1}H NMR (500 MHz, $CDCl_3$, 298 K) δ (ppm) = δ 7.35 (t, J = 7.4 Hz, 2H), 7.26 (d, J = 7.3 Hz, 1H), 7.23 (d, J = 7.4 Hz, 2H), 2.81 (t, J = 7.4 Hz, 2H), 2.34-2.30 (m, 0.0424H), 1.99 (t, J = 7.4 Hz, 2H).

^{2}H NMR (77 MHz, $CDCl_3$, 298 K) δ (ppm) = 2.30

^{13}C{^{1}H} NMR (125 MHz, $CDCl_3$, 298 K) δ (ppm) = 139.53, 128.42, 128.22, 126.25, 119.31, 77.25, 77.00, 76.75, 34.08, 26.50, 15.71.

3-Phenylpropanenitrile (1g)

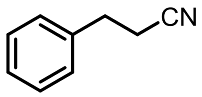

^{1}H NMR (500 MHz, $CDCl_3$, 298 K) δ (ppm) = δ 7.37 (t, *J* = 7.4 Hz, 2H), 7.31 (t, *J* = 7.5 Hz, 1H), 7.29 – 7.21 (m, 2H), 2.98 (t, *J* = 7.4 Hz, 2H), 2.64-2.61 (m, 2H).

^{13}C{^{1}H} NMR (125 MHz, $CDCl_3$, 298 K) δ (ppm) = 137.93, 128.64, 128.09, 127.00, 119.02, 31.31, 19.08.

3-Phenylpropanenitrile-2,2-d2 (3g)

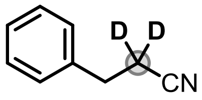

^{1}H NMR (500 MHz, $CDCl_3$, 298 K) δ (ppm) = 7.37 (t, *J* = 7.4 Hz, 2H), 7.28 (dd, *J* = 21.4, 11.1 Hz, 3H), 2.97 (s, 2H), 2.64 (d, *J* = 7.4 Hz, 0.1197H).

^{2}H NMR (77 MHz, $CDCl_3$, 298 K) δ (ppm) = 2.61.

^{13}C{^{1}H} NMR (125 MHz, $CDCl_3$, 298 K) δ (ppm) = 137.95, 128.77, 128.17, 127.13, 119.06, 31.30, 18.87.

Acetonitrile (1i)

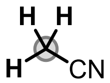

^{1}H NMR (500 MHz, D_2O, 298 K) δ (ppm) = 2.02 – 1.97 (m, 3H).

^{13}C{^{1}H} NMR (125 MHz, $CDCl_3$, 298 K) δ (ppm) = 119.02, 0.92.

Acetonitrile-d3 (3i)

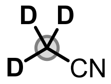

Deuteration: 97.9%, added 0.5 mmol CH_3OH as internal standard.

^{1}H NMR (500 MHz, D_2O, 298 K) δ (ppm) = 2.08-1.98 (m, 0.0661H).

^{2}H NMR (77 MHz, $CDCl_3$, 298 K) δ (ppm) = 1.95.

^{13}C{^{1}H} NMR (125 MHz, D_2O, 298 K) δ (ppm) = 119.05, 0.47.

2-Phenylacetonitrile (1a)

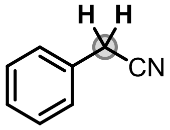

^{1}H NMR (500 MHz, $CDCl_3$, 298 K) δ (ppm) = 7.44 – 7.32 (m, 5H), 3.73 (s, 2H).

^{13}C{^{1}H} NMR (125 MHz, $CDCl_3$, 298 K) δ (ppm) = 129.79, 128.83, 127.71, 127.65, 117.76, 23.21.

2-Phenylacetonitrile-d2 (3a)

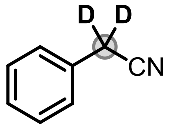

^{1}H NMR (500 MHz, $CDCl_3$, 298 K) δ (ppm) = 7.38 (dt, J = 24.3, 7.6 Hz, 5H), 3.78 – 3.73 (m, 0.0376H).

^{2}H NMR (77 MHz, $CDCl_3$, 298 K) δ (ppm) = 3.72

^{13}C{^{1}H} NMR (125 MHz, $CDCl_3$, 298 K) δ (ppm) = 129.25, 129.04, 127.96, 127.84,

117.81, 23.25.

2-(3-Bromophenyl)acetonitrile (1b)

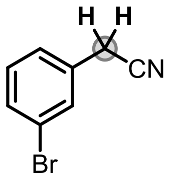

^{1}H NMR (500 MHz, $CDCl_3$, 298 K) δ (ppm) = 7.50 – 7.43 (m, 2H), 7.30 – 7.21 (m, 2H), 3.73 (s, 2H).

^{13}C{^{1}H} NMR (125 MHz, $CDCl_3$, 298 K) δ (ppm) = 131.90, 130.99, 130.73, 130.43, 126.40, 122.74, 117.10, 22.93.

2-(3-Bromophenyl)acetonitrile-d2 (3b)

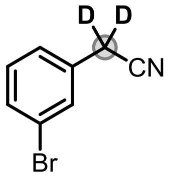

^{1}H NMR (500 MHz, $CDCl_3$, 298 K) δ (ppm) = 7.53 – 7.45 (m, 2H), 7.33 – 7.24 (m, 2H), 3.75-3.73 (m, 0.0493H).

^{2}H NMR (77 MHz, $CDCl_3$, 298 K) δ (ppm) = 3.71.

^{13}C{^{1}H} NMR (125 MHz, $CDCl_3$, 298 K) δ (ppm) = 131.84, 131.25, 130.95, 130.58, 126.52, 122.99, 117.10, 77.25, 77.00, 76.75, 22.87.

2-(m-Tolyl)acetonitrile (1c)

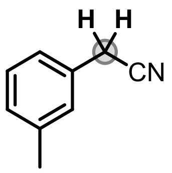

^{1}H NMR (500 MHz, $CDCl_3$, 298 K) δ (ppm) = 7.30 (t, J = 7.7 Hz, 1H), 7.18 (s, 1H), 7.18 – 7.12 (m, 2H), 3.72 (s, 2H), 2.40 (s, 3H).

^{13}C{^{1}H} NMR (125 MHz, $CDCl_3$, 298 K) δ (ppm) = 138.77, 129.69, 128.79, 128.53, 128.42, 124.76, 117.89, 23.23, 21.10.

2-(m-Tolyl)acetonitrile-d2 (3c)

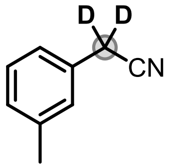

^{1}H NMR (500 MHz, $CDCl_3$, 298 K) δ (ppm) = 7.34 – 7.25 (m, 1H), 7.21 – 7.09 (m, 3H), 3.73-3.72 (m, 0.0372H), 2.39 (s, 3H).

^{2}H NMR (77 MHz, $CDCl_3$, 298 K) δ (ppm) = 3.68.

^{13}C{^{1}H} NMR (125 MHz, $CDCl_3$, 298 K) δ (ppm) = 138.94, 129.63, 128.93, 128.70, 128.56, 124.89, 117.95, 23.26, 21.24.

2-(4-Fluorophenyl)acetonitrile (1d)

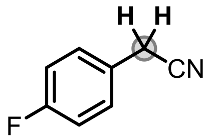

^{1}H NMR (500 MHz, $CDCl_3$, 298 K) δ (ppm) = 7.34-7.31 (m, 2H), 7.011-7.07 (m, 2H),

3.75 (s, 2H).

^{13}C{^{1}H} NMR (125 MHz, $CDCl_3$, 298 K) δ (ppm) = 163.35 (*J* = 245.6 Hz), 129.59 (*J* = 8.1 Hz), 125.62 (*J* = 3.3 Hz), 117.67, 116.06 (*J* = 21.8 Hz), 22.87.

2-(4-Fluorophenyl)acetonitrile-d2 (3d)

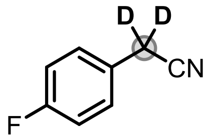

^{1}H NMR (500 MHz, $CDCl_3$, 298 K) δ (ppm) = 7.37 – 7.29 (m, 2H), 7.13 – 7.05 (m, 2H), 3.76 – 3.72 (m, 0.0481H).

^{2}H NMR (77 MHz, $CDCl_3$, 298 K) δ (ppm) = 3.69.

^{13}C{^{1}H} NMR (125 MHz, $CDCl_3$, 298 K) δ (ppm) = 162.41 (*J* = 245.8 Hz), 129.61 (*J* = 8.4 Hz), 125.52 (*J* = 3.2 Hz), 117.65, 116.11 (*J* = 21.8 Hz), 22.50.

2-(4-Methoxyphenyl)acetonitrile (1e)

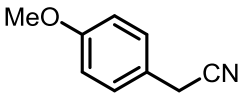

^{1}H NMR (500 MHz, $CDCl_3$, 298 K) δ (ppm) = 7.24 (d, *J* = 8.4 Hz, 2H), 6.91 (d, *J* = 8.4 Hz, 2H), 3.81 (s, 3H), 3.67 (s, 2H).

^{13}C{^{1}H} NMR (125 MHz, $CDCl_3$, 298 K) δ (ppm) = 159.10, 128.88, 121.68, 118.14, 114.28, 55.11, 22.50.

2-(4-Methoxyphenyl)acetonitrile-d2 (3e)

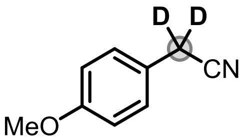

^{1}H NMR (500 MHz, $CDCl_3$, 298 K) δ (ppm) = 7.23 (d, *J* = 8.1 Hz, 2H), 6.90 (d, *J* = 8.1 Hz, 2H), 3.80 (s, 3H), 3.65 (s, 0.0502H).

^{2}H NMR (77 MHz, $CDCl_3$, 298 K) δ (ppm) = 3.64.

^{13}C{^{1}H} NMR (125 MHz, $CDCl_3$, 298 K) δ (ppm) = 159.09, 128.88, 121.57, 118.14, 114.27, 55.10, 22.30.

2-(Pyridin-3-yl)acetonitrile (1f)

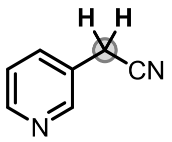

^{1}H NMR (500 MHz, $CDCl_3$, 298 K) δ (ppm) = 8.48-8.47 (m, 2H), 7.60 (d, J = 8.0 Hz, 1H), 7.23 (dd, J = 7.9, 4.9 Hz, 1H), 3.70 (s, 2H).

^{13}C{^{1}H} NMR (125 MHz, $CDCl_3$, 298 K) δ (ppm) = 149.04, 148.68, 135.13, 125.84, 123.46, 116.72, 20.74.

2-(Pyridin-3-yl)acetonitrile-d2 (3f)

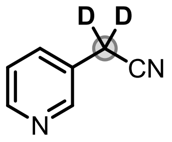

^{1}H NMR (500 MHz, $CDCl_3$, 298 K) δ (ppm) = 8.57 (s, 2H), 7.67 (d, J = 7.7 Hz, 1H), 7.32 (d, J = 7.8 Hz, 1H), 3.74 (d, J = 4.1 Hz, 0.0687H).

^{2}H NMR (77 MHz, $CDCl_3$, 298 K) δ (ppm) = 3.72.

^{13}C{^{1}H} NMR (125 MHz, $CDCl_3$, 298 K) δ (ppm) = 149.37, 148.94, 135.38, 125.94, 123.76, 116.74, 20.76.

2-(Cyclohex-1-en-1-yl)acetonitrile (1y)

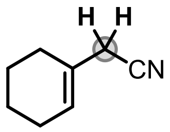

^{1}H NMR (400 MHz, $CDCl_3$, 298 K) δ (ppm) = 5.75 (d, J = 2.9 Hz, 1H), 2.96 (s, 2H), 2.04-1.96 (m, 4H), 1.67-1.62 (m, 2H), 1.58 – 1.54 (m, 2H).

^{13}C{^{1}H} NMR (125 MHz, $CDCl_3$, 298 K) δ (ppm) = 126.90, 126.17, 117.67, 27.89, 25.66, 24.97, 22.30, 21.61.

2-(Cyclohex-1-en-1-yl)acetonitrile-d2 (3y)

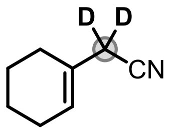

^{1}H NMR (400 MHz, $CDCl_3$, 298 K) δ (ppm) = 5.78 – 5.67 (m, 1H), 2.95 (s, 0.1327H), 2.04-1.95 (m, 4H), 1.69 – 1.62 (m, 2H), 1.57 – 1.52 (m, 2H).

^{13}C{^{1}H} NMR (100 MHz, $CDCl_3$, 298 K) δ (ppm) = 126.76, 125.98, 117.55, 27.70, 25.47, 25.05, 22.17, 21.50.

Adiponitrile (z)

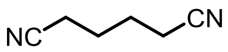

^{1}H NMR (500 MHz, $CDCl_3$, 298 K) δ (ppm) = 2.49 – 2.35 (m, 4H), 1.90 – 1.73 (m, 4H).

^{13}C{^{1}H} NMR (125 MHz, $CDCl_3$, 298 K) δ (ppm) = 118.78, 24.11, 16.45.

Hexanedinitrile-2,2,5,5-d4 (3z)

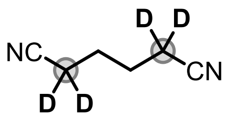

^{1}H NMR (500 MHz, $CDCl_3$, 298 K) δ (ppm) = 2.44 – 2.40 (m, 0.1973H), 1.81 (s, 4H).

^{2}H NMR (77 MHz, $CDCl_3$, 298 K) δ (ppm) =2.42.

^{13}C{^{1}H} NMR (125 MHz, $CDCl_3$, 298 K) δ (ppm) = 118.67, 23.91, 16.39.

4-Oxo-4-phenylbutanenitrile (1aa)

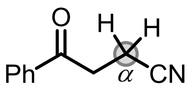

^{1}H NMR (400 MHz, $CDCl_3$, 298 K) δ (ppm) = 7.98 – 7.91 (m, 2H), 7.64 – 7.57 (m, 1H), 7.50-7.47 (m, 2H), 3.37 (t, J = 7.2 Hz, 2H), 2.76 (t, J = 7.2 Hz, 2H).

^{13}C{^{1}H} NMR (100 MHz, $CDCl_3$, 298 K) δ (ppm) = 195.29, 135.51, 133.81, 128.78, 127.93, 119.18, 34.17, 11.71.

4-Oxo-4-phenylbutanenitrile-2,2-d2 (3aa)

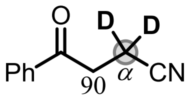

^{1}H NMR (400 MHz, $CDCl_3$, 298 K) δ (ppm) = 7.95-7.93 (m, 2H), 7.61 (t, J = 7.4 Hz, 1H), 7.49 (t, J = 7.7 Hz, 2H), 3.39-3.33 (m, 0.2165H), 2.78-2.74 (m, 0.2321H).

^{13}C{^{1}H} NMR (100 MHz, $CDCl_3$, 298 K) δ (ppm) = 195.38, 135.53, 133.83, 128.80, 127.94, 119.17, 77.32, 77.00, 76.68, 33.90, 11.70.

2-(Phenylsulfonyl)acetonitrile (1ab)

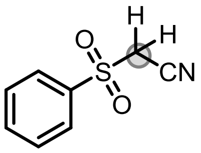

^{1}H NMR (400 MHz, $CDCl_3$, 298 K) δ (ppm) = 8.06 (dd, J = 8.1, 1.8 Hz, 2H), 7.84 – 7.77 (m, 1H), 7.68 (t, J = 7.8 Hz, 2H), 4.11 (s, 2H).

^{13}C{^{1}H} NMR (100 MHz, $CDCl_3$, 298 K) δ (ppm) = 136.63, 135.41, 129.79, 128.82, 110.41, 45.73.

2-(Phenylsulfonyl)acetonitrile-d2 (3ab)

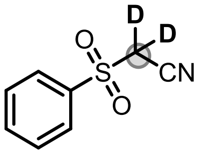

^{1}H NMR (400 MHz, $CDCl_3$, 298 K) δ (ppm) = 8.12 – 8.02 (m, 2H), 7.86 – 7.76 (m, 1H), 7.69 (t, J = 7.7 Hz, 2H), 4.10 – 4.08 (m, 0.2435H).

^{2}H NMR (61 MHz, $CDCl_3$, 298 K) δ (ppm) =4.11.

^{13}C{^{1}H} NMR (100 MHz, $CDCl_3$, 298 K) δ (ppm) = 136.62, 135.44, 129.81, 128.86, 110.31, 45.50.

Ethyl 3-cyanopropanoate (1ac)

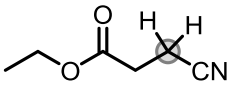

^{1}H NMR (400 MHz, $CDCl_3$, 298 K) δ (ppm) = 4.22 (q, J = 7.2 Hz, 2H), 2.76 – 2.61

(m, 4H), 1.31 (t, J = 7.1 Hz, 3H).

$^{13}C\{^{1}H\}$ NMR (100 MHz, $CDCl_3$, 298 K) δ (ppm) = 169.99, 118.44, 61.45, 29.98, 14.12, 12.95.

Ethyl 3-cyanopropanoate-3,3-d2 (3ac)

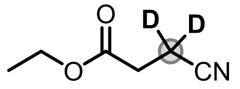

^{1}H NMR (400 MHz, $CDCl_3$, 298 K) δ (ppm) = 4.17 (q, J = 7.0 Hz, 2H), 2.64 (s, 2.0996H), 1.26 (t, J = 7.2 Hz, 3H).

^{2}H NMR (61 MHz, $CDCl_3$, 298 K) δ (ppm) =2.62.

$^{13}C\{^{1}H\}$ NMR (100 MHz, $CDCl_3$, 298 K) δ (ppm) =169.90, 118.41, 61.22, 29.62, 13.94, 12.80.

3-Phenylpropanamide-N,N,2,2-d4 (4)

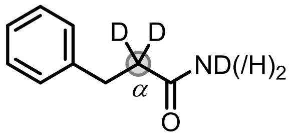

^{1}H NMR (500 MHz, DMSO-d6, 298 K) δ (ppm) = 7.27 (t, J = 7.5 Hz, 3H), 7.23 – 7.14 (m, 3H), 6.76 (s, 0.67H), 2.78 (s, 2H), 2.37-2.31 (m, 0.1514 H).

$^{13}C\{^{1}H\}$ NMR (125 MHz, $CDCl_3$, 298 K) δ (ppm) = 173.58, 141.45, 128.27, 128.21, 125.84, 36.54, 30.78.

5. X-ray Crystal Structure Data of the Manganese Complex

Single crystal XRD analysis was performed by fine-focus sealed tube dual source Rigaku

XtaLAB PRO dual source equipped with Pilatus 200K CuKα (λ=1.54184 Å) for complex **Mn-5** and **Mn-8**, and Rigaku Sygergy-S diffractometer dual source equipped with Dectris Pilatus3 R CdTe 300K detector MoKα (λ=0.71073 Å) for complex **Mn-7**. Data collection was performed in low temperature under LN. Data were processed with CrysAlis^{PRO} (Rigaku). Structure was solved using SHELXT[8]. Refinement performed based on F^2 with SHELXL[9] with full matrix least-squares. All non-hydrogen atoms were treated anisotropically, hydrogens were calculated and refined in riding mode. Full details can be found in the CIF file and Tables S2-S7.

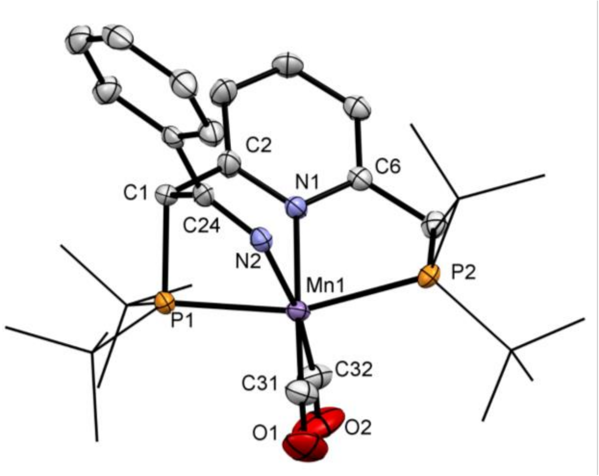

Figure S7. X-ray crystal structure of complex **Mn-5**. Atoms are presented as thermal ellipsoids at 50% probability level. The P(tert-butyl)$_2$ groups are drawn as wire frames, and hydrogen atoms are omitted for clarity.

Table S2. Selected bond lengths [Å] and bond angles [˚] of complex **Mn-5**.

Bond Lengths [Å]		Bond Angles [˚]	
Mn(1)-N(1)	2.0530(13)	N(1)-Mn(1)-P(2)	79.78(4)
Mn(1)-N(2)	2.0495(13)	N(1)-Mn(1)-P(1)	80.02(4)
Mn(1)-C(31)	1.7812(17)	N(2)-Mn(1)-P(2)	95.45(4)
Mn(1)-C(32)	1.7764(18)	N(2)-Mn(1)-N(1)	82.45(5)
Mn(1)-P(1)	2.3239(9)	N(2)-Mn(1)-P(1)	79.49(5)
Mn(1)-P(2)	2.3163(4)	C(31)-Mn(1)-P(2)	97.46(6)
P(2)-C(7)	1.8569(16)	C(31)-Mn(1)-N(1)	171.84(7)
P(2)-C(16)	1.8952(16)	C(31)-Mn(1)-N(2)	90.21(7)
P(1)-C(1)	1.869(3)	C(31)-Mn(1)-P(1)	102.23(6)
P(1)-C(8)	1.912(2)	C(32)-Mn(1)-P(2)	93.25(6)
O(1)-C(31)	1.165(2)	C(32)-Mn(1)-N(1)	99.02(7)

C(1)-C(2)	1.511(3)	C(32)-Mn(1)-N(2)	171.30(7)
O(2)-C(32)	1.168(2)	C(32)-Mn(1)-C(31)	88.77(8)
N(1)-C(2)	1.362(2)	C(32)-Mn(1)-P(1)	92.28(7)
N(2)-C(24)	1.271(2)	C(7)-P(2)-Mn(1)	96.60(5)

Table S3. Crystal data, data collection and structure refinement of complex **Mn-5**.

	**Complex Mn-5**
CCDC No.	2070221
Diffractometer	Rigaku XtaLAB PRO
Empirical formula	$C_{32}H_{47}MnN_2O_2P_2$
Crystal description	red plate
Formula weight (g/mol)	608.59
Temperature (K)	100(2)
Wavelength (Å)	1.54184
Crystal system	Monoclinic
Space group	*P21/n*
a (Å)	12.9252(2)
b (Å)	16.7518(2)
c (Å)	16.3352(2)
α (°)	90
β (°)	101.960(1)
γ (°)	90
Volume (Å^3)	3460.2(1)
Z	4
Density calculated (Mg/m^3)	1.168
Absorption coefficient (mm^{-1})	4.187
F(000)	1296
Crystal size (mm^3)	0.283 x 0.150 x 0.070
Reflection collected (Unique)	29932 / 7042
R int	0.0367
Completeness	99.6%
Data\restraints\parameters	7042 / 0 / 426
Goodness-of-fit on F^2	1.055
Final R [I>2σ(I)]	R1 = 0.0332, wR2 = 0.0860
R (all data)	R1 = 0.0350, wR2 = 0.0870
Largest diff. peak and hole (e$^{\cdot}$ Å^{-3})	0.451 and –0.430

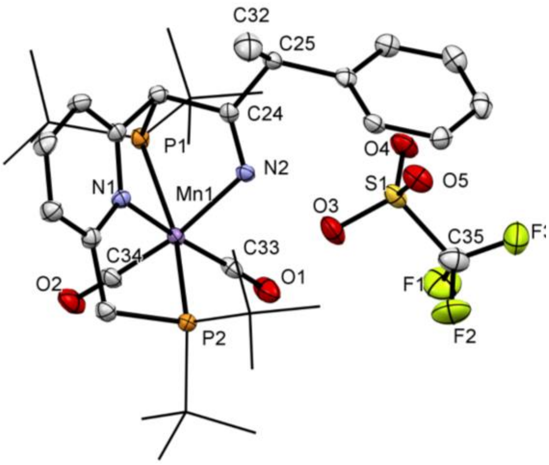

Figure S8. X-ray crystal structure of complex **Mn-7**. Atoms are presented as thermal ellipsoids at 50% probability level. The P(tert-butyl)$_2$ groups are drawn as wire frames, and hydrogen atoms are omitted for clarity.

Table S4. Selected bond lengths [Å] and bond angles [˚] of complex **Mn-7**.

Bond Lengths [Å]		Bond Angles [˚]	
Mn(1)-P(1)	2.3123(11)	P(1)-Mn(1)-P(2)	160.02(4)
Mn(1)-P(2)	2.3307(11)	N(1)-Mn(1)-P(1)	80.19(9)
Mn(1)-N(1)	2.058(3)	N(1)-Mn(1)-P(2)	79.94(9)
Mn(1)-N(2)	2.053(3)	N(2)-Mn(1)-P(1)	80.39(9)
Mn(1)-C(33)	1.784(4)	N(2)-Mn(1)-P(2)	98.63(9)
Mn(1)-C(34)	1.782(4)	N(2)-Mn(1)-N(1)	81.48(12)
N(2)-C(24)	1.280(5)	C(33)-Mn(1)-P(1)	102.43(13)
C(24)-C(25)	1.515(5)	C(33)-Mn(1)-P(2)	97.54(13)
C(25)-C(32)	1.531(6)	C(34)-Mn(1)-C(33)	87.34(18)
P(1)-C(1)	1.893(4)	C(1)-P(1)-Mn(1)	89.08(12)
P(1)-C(8)	1.891(4)	C(1)-P(1)-C(12)	103.89(19)
P(1)-C(12)	1.901(4)	C(8)-P(1)-Mn(1)	120.09(14)
P(2)-C(7)	1.851(4)	C(8)-P(1)-C(1)	108.99(17)
O(1)-C(33)	1.155(5)	C(8)-P(1)-C(12)	109.46(19)
O(2)-C(34)	1.157(5)	C(12)-P(1)-Mn(1)	120.96(14)

Table S5. Crystal data, data collection and structure refinement of complex **Mn-7**.

	**Complex 7**
CCDC No.	2070222
Diffractometer	Rigaku Synergy-S
Empirical formula	$C_{36}H_{53}Cl_2F_3MnN_2O_5P_2S$
Crystal description	yellow prism

Formula weight (g/mol)	870.64
Temperature (K)	100(2)
Wavelength (Å)	0.71073
Crystal system	Orthorhombic
Space group	$P2_12_12_1$
a (Å)	12.1759(3)
b (Å)	13.3287(3)
c (Å)	25.6006(6)
α (°)	90
β (°)	90
γ (°)	90
Volume (Å^3)	4154.69(17)
Z	4
Density calculated (Mg/m^3)	1.392
Absorption coefficient (mm^{-1})	0.629
Crystal size (mm^3)	0.182 x 0.174 x 0.053
R int	0.0707
Reflection collected (Unique)	51677 / 10302
Completeness	100.0%
Data\restraints\parameters	10302 / 0 / 4916
Goodness-of-fit on F^2	1.052
Goodness-of-fit on F^2	1.052
Final R [I>2σ(I)]	R1 = 0.0465, wR2=0.1097
R (all data)	R1 = 0.0536, wR2=0.1135
Largest diff. peak and hole (e$\cdot$ Å^{-3})	1.133 and − 0.756

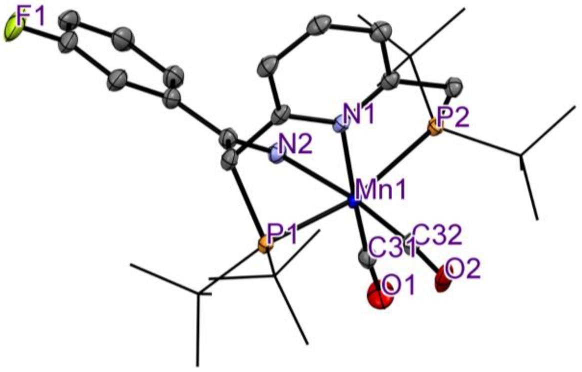

Figure S9. X-ray crystal structure of complex **Mn-8**. Atoms are presented as thermal ellipsoids at 50% probability level. The P(tert-butyl)$_2$ groups are drawn as wire frames, and hydrogen atoms are omitted for clarity.

Table S6. Selected bond lengths [Å] and bond angles [˚] of complex **Mn-8**.

Bond Lengths [Å]		Bond Angles [˚]	
Mn(1)-P(1)	2.3035(5)	P(1)-Mn(1)-P(2)	162.48(2)
Mn(1)-P(2)	2.3161(5)	N(1)-Mn(1)-P(1)	82.71(4)
Mn(1)-N(1)	2.0596(15)	N(1)-Mn(1)-P(2)	79.93(4)
Mn(1)-N(2)	2.0470(16)	N(2)-Mn(1)-P(1)	78.84(5)
Mn(1)-C(31)	1.780(2)	N(2)-Mn(1)-P(2)	96.45(5)
Mn(1)-C(32)	1.777(2)	N(2)-Mn(1)-N(1)	82.50(6)
P(1)-C(1)	1.8637(19)	C(31)-Mn(1)-P(1)	102.38(6)
P(1)-C(8)	1.918(2)	C(31)-Mn(1)-P(2)	94.75(6)
P(1)-C(12)	1.8966(19)	C(31)-Mn(1)-N(1)	173.32(8)
P(2)-C(7)	1.8531(19)	C(31)-Mn(1)-N(2)	94.17(8)
P(2)-C(16)	1.8973(19)	C(32)-Mn(1)-P(1)	91.48(6)
P(2)-C(20)	1.896(2)	C(32)-Mn(1)-P(2)	92.57(6)
O(1)-C(31)	1.165(3)	C(32)-Mn(1)-N(1)	95.58(8)
O(2)-C(32)	1.164(3)	C(32)-Mn(1)-N(2)	170.29(7)
N(1)-C(2)	1.355(2)	C(32)-Mn(1)-C(31)	88.67(9)
N(1)-C(6)	1.354(2)	C(1)-P(1)-Mn(1)	89.49(6)
N(2)-C(24)	1.267(3)	C(1)-P(1)-C(8)	104.06(9)

Table S7. Crystal data, data collection and structure refinement of complex **Mn-8**.

	**Complex Mn-8**
CCDC No.	2092662
Diffractometer	Rigaku Xtalab PRO
Empirical formula	$C_{32}H_{46}FMnN_2O_2P_2$
Crystal description	orange prism
Formula weight (g/mol)	626.59
Temperature (K)	100(2)
Crystal system	Monoclinic
Wavelength (Å)	1.54184
Space group	$P2_1/c$
a (Å)	12.6518(2)
b (Å)	15.3285(3)
c (Å)	16.7761(4)
α (°)	90
β (°)	104.703(2)
γ (°)	90
Volume ($Å^3$)	3146.90(12)
Z	4
Density calculated (Mg/m^3)	1.323
Absorption coefficient (mm^{-1})	4.667
Crystal size (mm^3)	0.157 x 0.057 x 0.036

Reflection collected (Unique)	27152 / 6430
R int	0.0643
Completeness	99.9%
Data\restraints\parameters	6430 / 0 / 373
Goodness-of-fit on F^2	1.048
Final R [I>2σ(I)]	R1 = 0.0388, wR2 = 0.1030
R (all data)	R1 = 0.0421, wR2 = 0.1051
Largest diff. peak and hole (e^{-} Å^{-3})	0.879 and –0.640

6. Computational Details

All geometries were optimized using Truhlar's M06-L functional, [10] the triple-ξ def2-TZVP basis set[11] and W06 density fitting to increase computational efficiency[12] as well as Grimme's D3(0) empirical dispersion correction. [13] Frequency calculations at this level of theory were run in order to confirm stationary points and transition states, as well as to compute thermodynamic properties. Single point energies of the optimized structures were computed using the range-separated meta-GGA hybrid functional ωB97M-V of the Head-Gordon group[14] including dispersion correction, [15] together with the triple-ξ def2-TZVPP basis set[11] and the corresponding auxiliary basis sets, def2/J[12] and def2-TZVPP/C, [16] for RIJCOSX density fitting. Gibbs free energies were computed by adding the free energy correction terms from the frequency calculations to the single point energies according to

$$G^{\omega B97M\text{-}V}_{gas} = E^{\omega B97M\text{-}V}_{el/gas} + corr^{M06\text{-}L}_{freq/gas}$$

where $E^{\omega B97M\text{-}V}_{el/gas}$ is the single point electronic energy and $corr^{M06\text{-}L}_{freq/gas}$ is a thermal correction term to the Gibbs free energy from the frequency calculation.
Optimizations and frequency calculations were done using the Gaussian 16 software suite in the C.01 revision. [17]Single point calculations were performed using Gaussian 16[17]or the ORCA Software in the 4.2.1 release. [18]

Summary of the calculated energies of intermediates and transition states

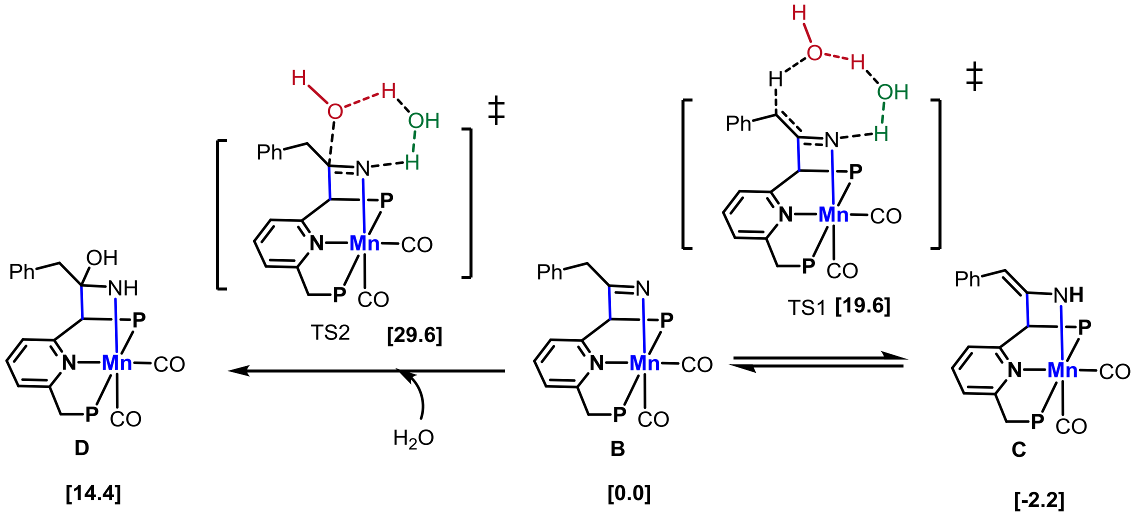

Figure S10. Calculated gas phase Gibbs free energies of intermediate and transition states. Energy values provided inside brackets correspond to Gibbs free energies in kcal/mol with respect to **B**+2H_2O at 298 K and 1 atmosphere pressure. Mass balance is ensured throughout.

We conducted a preliminary density functional theory (DFT) study to verify that α-deuteration mechanism, involving the formation of intermediate **C** from **B** is more favored than the hydration mechanism, involving the generation of intermediate **D** from **B**. According to our calculations, the enamine complex **C** is thermodynamically more stable than the imine complex **B** by 2.2 kcal/mol in the gas phase. On the other hand, the hydration intermediate **D** was found to be significantly higher in energy (14.4 kcal/mol). On a similar note, the relevant transition state leading to α-deuteration (TS1) was found less energetically demanding (19.6 kcal/mol) than the corresponding hydration transition state (TS2, 29.6 kcal/mol) (Figure S10). These results, along with the experimental observations, suggest that the deuteration pathway is more readily accessible than the hydration pathway. As a result, when toluene is used as solvent, only deuteration is observed due to the limited solubility of water in toluene. On the other hand, when t-BuOH is used as solvent, both deuteration and hydration are observed.

Coordinates of the computed structures:

Water

8	-4.486032000	1.955746000	-0.004827000
1	-3.526882000	1.912621000	0.008262000
1	-4.763253000	1.076053000	0.262160000

B

15	-2.450935000	-0.308387000	0.031896000
7	-0.174931000	1.021439000	1.051861000

6	0.892462000	1.841653000	0.960642000
6	0.954924000	3.025267000	1.687468000
1	1.812112000	3.676508000	1.575110000
6	-0.094078000	3.351725000	2.522714000
1	-0.063832000	4.264355000	3.103920000
6	-1.203802000	2.517606000	2.584937000
1	-2.057935000	2.769312000	3.198676000
6	-1.228334000	1.364604000	1.819256000
6	-2.428555000	0.478374000	1.714819000
1	-3.338046000	1.023817000	1.975393000
1	-2.345909000	-0.357513000	2.417324000
6	1.883399000	1.434845000	-0.065184000
1	2.785988000	2.048455000	-0.017080000
6	3.106169000	-0.794604000	1.541411000
6	3.240197000	-0.771554000	-1.544365000
6	3.114306000	-2.303130000	1.809329000
1	2.113892000	-2.727395000	1.795314000
1	3.525674000	-2.478229000	2.805765000
1	3.727901000	-2.860038000	1.108789000
6	4.325332000	0.287307000	-1.737405000
1	3.898751000	1.268780000	-1.940985000
1	4.930412000	0.018877000	-2.606529000
1	4.999790000	0.383997000	-0.891575000
6	-3.692025000	-1.727505000	0.244002000
6	-4.146052000	-2.223560000	-1.128412000
1	-3.299375000	-2.463791000	-1.771896000
1	-4.780326000	-1.511544000	-1.651268000
1	-4.726852000	-3.139146000	-0.999812000
6	-4.902346000	-1.353941000	1.101497000
1	-5.499234000	-0.544962000	0.693948000
1	-4.610298000	-1.082005000	2.115692000
1	-5.554595000	-2.226057000	1.185475000
6	-2.998358000	-2.895476000	0.942466000
1	-3.717327000	-3.711000000	1.046706000
1	-2.652336000	-2.640430000	1.942163000
1	-2.149313000	-3.272229000	0.376583000
6	-3.239059000	0.999716000	-1.093548000
6	-2.952281000	0.605597000	-2.545005000
1	-3.449675000	-0.316188000	-2.838028000
1	-1.879723000	0.497379000	-2.707435000
1	-3.319931000	1.395097000	-3.205282000
6	-2.532651000	2.328776000	-0.817347000
1	-2.860927000	3.055490000	-1.564748000
1	-1.452264000	2.237124000	-0.895369000

1	-2.784746000	2.739331000	0.161459000
6	-4.733008000	1.240373000	-0.899968000
1	-4.982194000	1.512065000	0.126773000
1	-5.348046000	0.391605000	-1.189756000
1	-5.033130000	2.079541000	-1.532420000
15	2.117476000	-0.408932000	-0.038744000
6	3.884360000	-2.146045000	-1.356349000
1	4.395206000	-2.422469000	-2.280254000
1	4.627065000	-2.166125000	-0.563269000
1	3.140683000	-2.918478000	-1.154841000
6	2.433532000	-0.849598000	-2.845303000
1	3.088755000	-1.257726000	-3.618324000
1	1.569302000	-1.502293000	-2.763371000
1	2.079936000	0.116095000	-3.189679000
6	2.391998000	-0.126032000	2.721888000
1	2.505625000	0.956245000	2.709959000
1	1.331341000	-0.361639000	2.770010000
1	2.846965000	-0.487938000	3.645997000
6	4.539393000	-0.267563000	1.539465000
1	4.970333000	-0.412398000	2.533049000
1	5.185363000	-0.787699000	0.836040000
1	4.585313000	0.800534000	1.322474000
6	1.068589000	1.547306000	-1.400590000
6	-0.293362000	-2.094812000	-1.237471000
8	-0.413944000	-3.019422000	-1.929517000
25	-0.172433000	-0.699119000	-0.122769000
6	-0.233101000	-1.751136000	1.309327000
8	-0.211308000	-2.391019000	2.284803000
7	0.160101000	0.713714000	-1.623397000
6	1.416826000	2.721788000	-2.306956000
1	0.667718000	2.736337000	-3.099383000
1	2.384408000	2.532513000	-2.783957000
6	1.468794000	4.035720000	-1.584036000
6	0.297757000	4.739454000	-1.306671000
6	2.674261000	4.558127000	-1.118734000
6	0.327827000	5.914160000	-0.573001000
6	2.710425000	5.734218000	-0.384418000
6	1.534163000	6.413340000	-0.102254000
1	-0.646375000	4.354885000	-1.672880000
1	3.597885000	4.033427000	-1.341262000
1	-0.594276000	6.444091000	-0.369393000
1	3.659194000	6.124081000	-0.037553000
1	1.559004000	7.332693000	0.468336000

C

15	-2.465540000	-0.116839000	-0.045101000
7	-0.151435000	0.949695000	1.181226000
6	0.973171000	1.686998000	1.214349000
6	1.095899000	2.760924000	2.086169000
1	2.015114000	3.329177000	2.112252000
6	0.039168000	3.074589000	2.919904000
1	0.120295000	3.898477000	3.616823000
6	-1.134068000	2.342240000	2.836503000
1	-1.992955000	2.590667000	3.444905000
6	-1.210079000	1.287667000	1.940941000
6	-2.466956000	0.515195000	1.700089000
1	-3.343487000	1.110449000	1.964987000
1	-2.490582000	-0.380072000	2.329707000
6	1.999185000	1.307803000	0.195827000
1	2.951237000	1.804295000	0.375386000
6	2.960485000	-1.123843000	1.697459000
6	3.288137000	-0.897843000	-1.366535000
6	2.844708000	-2.644955000	1.844584000
1	1.817797000	-2.989128000	1.752851000
1	3.191819000	-2.925124000	2.841393000
1	3.450048000	-3.192546000	1.130210000
6	4.450789000	0.095158000	-1.419589000
1	4.105538000	1.115151000	-1.581350000
1	5.086687000	-0.162358000	-2.269706000
1	5.079296000	0.079783000	-0.534092000
6	-3.824705000	-1.438225000	-0.012111000
6	-4.269291000	-1.751505000	-1.440158000
1	-3.422078000	-1.964812000	-2.092922000
1	-4.853638000	-0.950454000	-1.887031000
1	-4.901748000	-2.641120000	-1.428427000
6	-5.030451000	-1.050043000	0.845515000
1	-5.534879000	-0.147806000	0.515625000
1	-4.756609000	-0.921240000	1.892409000
1	-5.761488000	-1.860649000	0.809619000
6	-3.252223000	-2.723657000	0.582220000
1	-4.035847000	-3.484145000	0.578146000
1	-2.928633000	-2.599948000	1.613622000
1	-2.412999000	-3.109862000	0.007968000
6	-3.096101000	1.354847000	-1.068948000
6	-2.862240000	1.043779000	-2.550812000
1	-3.605540000	0.360412000	-2.953818000

1	-1.884436000	0.600440000	-2.727476000
1	-2.921974000	1.966447000	-3.131103000
6	-2.261924000	2.582027000	-0.684641000
1	-2.521738000	3.401365000	-1.357972000
1	-1.192736000	2.404497000	-0.772122000
1	-2.482504000	2.922266000	0.327382000
6	-4.560372000	1.730484000	-0.861246000
1	-4.791370000	1.938193000	0.184185000
1	-5.252952000	0.973687000	-1.222627000
1	-4.769304000	2.645890000	-1.420066000
15	2.103276000	-0.563054000	0.093793000
6	3.825022000	-2.325481000	-1.248042000
1	4.375017000	-2.564425000	-2.159691000
1	4.514528000	-2.456613000	-0.418433000
1	3.022455000	-3.058731000	-1.153025000
6	2.556349000	-0.823121000	-2.711494000
1	3.232554000	-1.215030000	-3.474736000
1	1.653011000	-1.426472000	-2.731202000
1	2.306898000	0.195671000	-2.990550000
6	2.224160000	-0.498864000	2.887371000
1	2.417434000	0.568292000	2.975465000
1	1.148120000	-0.656392000	2.857456000
1	2.594124000	-0.967023000	3.801474000
6	4.424686000	-0.707005000	1.816024000
1	4.780935000	-0.953865000	2.818976000
1	5.073686000	-1.223593000	1.113285000
1	4.561923000	0.366447000	1.681021000
6	1.404968000	1.653940000	-1.164654000
6	-0.363291000	-1.982092000	-1.347624000
8	-0.507299000	-2.841243000	-2.114484000
25	-0.213064000	-0.678519000	-0.133511000
6	-0.433952000	-1.824640000	1.199524000
8	-0.520187000	-2.545085000	2.112601000
7	0.390373000	0.819559000	-1.444337000
1	0.023622000	0.968755000	-2.375415000
6	1.904215000	2.622819000	-2.002628000
1	1.476086000	2.609004000	-3.003103000
6	2.993598000	3.549144000	-1.829071000
6	3.396598000	4.108960000	-0.602015000
6	3.715926000	3.967342000	-2.965444000
6	4.476292000	4.971040000	-0.512481000
6	4.784369000	4.837075000	-2.876231000
6	5.188746000	5.338991000	-1.644445000
1	2.813696000	3.909505000	0.286009000

1	3.422860000	3.575941000	-3.933326000
1	4.747917000	5.381448000	0.453036000
1	5.313853000	5.123864000	-3.776835000
1	6.028097000	6.017548000	-1.572440000

D

15	-2.501469000	-0.138340000	-0.142421000
7	-0.176737000	0.974211000	1.113651000
6	0.954314000	1.697477000	1.218929000
6	1.066454000	2.714950000	2.157373000
1	1.987857000	3.277845000	2.227577000
6	-0.015579000	2.999359000	2.969083000
1	0.047601000	3.792348000	3.703092000
6	-1.190175000	2.276957000	2.819826000
1	-2.061108000	2.497814000	3.422193000
6	-1.246889000	1.259895000	1.879216000
6	-2.484342000	0.456058000	1.619567000
1	-3.377120000	1.009559000	1.919155000
1	-2.464009000	-0.457209000	2.223960000
6	1.995364000	1.397803000	0.185615000
1	2.939961000	1.890021000	0.428339000
6	2.806557000	-1.061364000	1.725788000
6	3.353319000	-0.972525000	-1.287989000
6	2.742454000	-2.600178000	1.805052000
1	1.913703000	-3.025696000	1.244961000
1	2.604242000	-2.902641000	2.843333000
1	3.656668000	-3.068827000	1.455762000
6	4.544999000	-0.014791000	-1.369733000
1	4.244816000	0.994144000	-1.633019000
1	5.225904000	-0.378140000	-2.143461000
1	5.110280000	0.022561000	-0.441781000
6	-3.777011000	-1.544918000	-0.120976000
6	-4.227411000	-1.834966000	-1.553095000
1	-3.379429000	-1.994921000	-2.219827000
1	-4.849812000	-1.049021000	-1.973143000
1	-4.821267000	-2.750917000	-1.558610000
6	-4.985172000	-1.246535000	0.768707000
1	-5.546264000	-0.366569000	0.473021000
1	-4.696579000	-1.128554000	1.813105000
1	-5.670494000	-2.095930000	0.726278000
6	-3.140078000	-2.823826000	0.415375000
1	-3.888688000	-3.618306000	0.383743000
1	-2.813441000	-2.729019000	1.448208000
1	-2.290178000	-3.147040000	-0.181792000

6	-3.285197000	1.299018000	-1.109882000
6	-2.999379000	1.073648000	-2.598128000
1	-3.649023000	0.319841000	-3.036274000
1	-1.972422000	0.756187000	-2.774750000
1	-3.165889000	2.002847000	-3.147222000
6	-2.546534000	2.555063000	-0.652736000
1	-2.781248000	3.383049000	-1.325335000
1	-1.472105000	2.400605000	-0.667959000
1	-2.840315000	2.860610000	0.352274000
6	-4.774362000	1.560741000	-0.911135000
1	-5.028510000	1.725563000	0.136431000
1	-5.411964000	0.769901000	-1.298934000
1	-5.040099000	2.474811000	-1.448349000
15	2.084600000	-0.473972000	0.051710000
6	3.902866000	-2.378271000	-1.032886000
1	4.443447000	-2.687213000	-1.929819000
1	4.608728000	-2.423281000	-0.207966000
1	3.114918000	-3.111773000	-0.864797000
6	2.622920000	-1.070929000	-2.637855000
1	3.346080000	-0.955327000	-3.447457000
1	2.163058000	-2.051499000	-2.748890000
1	1.818948000	-0.349451000	-2.759343000
6	1.985437000	-0.510704000	2.898630000
1	2.149351000	0.551623000	3.055550000
1	0.917651000	-0.689812000	2.806777000
1	2.320446000	-1.018163000	3.804880000
6	4.243777000	-0.595280000	1.958648000
1	4.545223000	-0.866185000	2.973326000
1	4.956282000	-1.059184000	1.281894000
1	4.345247000	0.487556000	1.871333000
6	1.402156000	1.957156000	-1.158897000
6	-0.346075000	-1.753772000	-1.620821000
8	-0.485585000	-2.537347000	-2.464868000
25	-0.221385000	-0.555541000	-0.295659000
6	-0.395368000	-1.800252000	0.947949000
8	-0.437165000	-2.580549000	1.815392000
7	0.388462000	1.064937000	-1.552323000
1	-0.264341000	1.529054000	-2.171398000
6	0.953454000	3.430006000	-1.007490000
1	0.047689000	3.469760000	-0.402525000
6	1.911597000	4.438930000	-0.432061000
6	3.295946000	4.389115000	-0.598960000
6	1.387971000	5.484398000	0.331858000
6	4.117390000	5.339312000	-0.008003000

6	2.204316000	6.438717000	0.915294000
6	3.580169000	6.366057000	0.752580000
1	3.727372000	3.602514000	-1.202549000
1	0.313792000	5.535924000	0.478763000
1	5.189357000	5.275711000	-0.148702000
1	1.766600000	7.236750000	1.502132000
1	4.225530000	7.104001000	1.211399000
8	2.472601000	2.048659000	-2.153202000
1	2.207713000	1.412409000	-2.825827000
1	0.653994000	3.735429000	-2.015432000

TS1

15	-2.553009000	-0.491031000	-0.067579000
7	-0.312472000	-0.126427000	1.603753000
6	0.714313000	0.579837000	2.106255000
6	0.765187000	0.917853000	3.452408000
1	1.604416000	1.487877000	3.827477000
6	-0.260775000	0.506630000	4.281492000
1	-0.234578000	0.738768000	5.338103000
6	-1.337984000	-0.182290000	3.744297000
1	-2.175536000	-0.477612000	4.361311000
6	-1.352360000	-0.467841000	2.389077000
6	-2.522574000	-1.082521000	1.692863000
1	-3.444530000	-0.894477000	2.247101000
1	-2.405504000	-2.169136000	1.632036000
6	1.725492000	1.006427000	1.095533000
1	2.604476000	1.443529000	1.569977000
6	3.019244000	-1.726473000	0.942480000
6	3.213249000	0.275734000	-1.411046000
6	3.113465000	-3.041875000	0.160654000
1	2.139747000	-3.388561000	-0.176905000
1	3.516025000	-3.809370000	0.824680000
1	3.771290000	-2.987590000	-0.699517000
6	4.206270000	1.280527000	-0.828339000
1	3.704638000	2.154047000	-0.414714000
1	4.854990000	1.637250000	-1.631445000
1	4.847841000	0.863543000	-0.056648000
6	-3.751082000	-1.703050000	-0.898103000
6	-4.233780000	-1.114725000	-2.223138000
1	-3.404991000	-0.774053000	-2.844618000
1	-4.924051000	-0.284944000	-2.089937000
1	-4.763780000	-1.887659000	-2.782676000
6	-4.944602000	-2.079348000	-0.018030000

1	-5.569643000	-1.238614000	0.262903000
1	-4.630939000	-2.588450000	0.893054000
1	-5.575230000	-2.781001000	-0.568045000
6	-3.008278000	-3.000850000	-1.210337000
1	-3.700359000	-3.681239000	-1.710554000
1	-2.654555000	-3.505111000	-0.313322000
1	-2.159207000	-2.848464000	-1.872489000
6	-3.393663000	1.208880000	0.040644000
6	-3.225946000	1.934965000	-1.295451000
1	-3.867783000	1.527316000	-2.072727000
1	-2.202168000	1.916294000	-1.662231000
1	-3.491446000	2.986702000	-1.169595000
6	-2.678019000	2.020925000	1.124533000
1	-3.099120000	3.028400000	1.127979000
1	-1.609073000	2.113494000	0.942076000
1	-2.836996000	1.612847000	2.122522000
6	-4.872725000	1.175755000	0.415187000
1	-5.057394000	0.647212000	1.351423000
1	-5.498048000	0.736220000	-0.358440000
1	-5.217396000	2.202618000	0.555702000
15	2.051830000	-0.441367000	-0.070836000
6	3.966987000	-0.872002000	-2.085622000
1	4.511851000	-0.467712000	-2.939926000
1	4.698754000	-1.347870000	-1.438854000
1	3.289757000	-1.636530000	-2.468900000
6	2.427402000	0.976415000	-2.525804000
1	3.130681000	1.199737000	-3.330634000
1	1.646050000	0.348447000	-2.944571000
1	1.978492000	1.917638000	-2.229386000
6	2.241603000	-2.028571000	2.228533000
1	2.289362000	-1.209060000	2.941778000
1	1.197278000	-2.275956000	2.050676000
1	2.701780000	-2.895073000	2.706673000
6	4.413889000	-1.268949000	1.364359000
1	4.838900000	-2.013500000	2.041303000
1	5.103036000	-1.168579000	0.529816000
1	4.391496000	-0.321047000	1.903480000
6	1.048828000	1.975377000	0.131542000
6	-0.327487000	-0.948928000	-2.181356000
8	-0.433055000	-1.184259000	-3.310571000
25	-0.246694000	-0.633286000	-0.417967000
6	-0.283113000	-2.371630000	-0.057244000
8	-0.243294000	-3.501527000	0.221696000
7	0.151524000	1.395374000	-0.601726000

6	1.392896000	3.389497000	0.034722000
1	0.288143000	3.982218000	-0.024092000
1	1.710247000	3.621819000	-0.985846000
8	-0.896369000	4.592666000	-0.440228000
1	-0.731558000	3.931685000	-1.891139000
1	-1.619725000	4.308155000	0.124677000
8	-0.416606000	3.361378000	-2.666504000
1	-0.247968000	2.011525000	-1.315422000
1	-0.995255000	3.565325000	-3.403090000
6	2.274453000	3.979072000	1.060262000
6	1.884442000	3.973882000	2.404290000
6	3.481116000	4.607596000	0.743075000
6	2.682760000	4.526536000	3.391272000
6	4.276683000	5.174686000	1.726113000
6	3.889301000	5.126967000	3.057662000
1	0.916788000	3.554538000	2.661015000
1	3.792921000	4.651052000	-0.294764000
1	2.352142000	4.511165000	4.423155000
1	5.207251000	5.655501000	1.451047000
1	4.511485000	5.568778000	3.824899000

TS2

15	-2.648580000	-0.687686000	-0.111338000
7	-0.446040000	0.829789000	0.900551000
6	0.558334000	1.724439000	0.832030000
6	0.546280000	2.878817000	1.602450000
1	1.360311000	3.587256000	1.519680000
6	-0.522364000	3.099678000	2.452356000
1	-0.554226000	3.990201000	3.066593000
6	-1.565444000	2.186905000	2.492514000
1	-2.429913000	2.355618000	3.120160000
6	-1.510550000	1.055946000	1.690993000
6	-2.633058000	0.070954000	1.587843000
1	-3.578385000	0.527852000	1.888037000
1	-2.458397000	-0.768404000	2.269225000
6	1.576054000	1.436693000	-0.237737000
1	2.441806000	2.089351000	-0.143062000
6	2.819215000	-0.675293000	1.514032000
6	3.189989000	-0.838798000	-1.532560000
6	2.994788000	-2.183188000	1.770979000
1	2.157235000	-2.778441000	1.417081000
1	3.069857000	-2.351913000	2.846027000
1	3.899917000	-2.578496000	1.323984000
6	4.201125000	0.282381000	-1.773613000
1	3.705052000	1.143355000	-2.219475000

1	4.943770000	-0.077209000	-2.490108000
1	4.739095000	0.577084000	-0.875580000
6	-3.733649000	-2.227792000	0.108012000
6	-4.200506000	-2.711601000	-1.265032000
1	-3.368045000	-2.827249000	-1.959623000
1	-4.936836000	-2.054449000	-1.720426000
1	-4.669427000	-3.691026000	-1.154303000
6	-4.933439000	-1.989954000	1.027064000
1	-5.609087000	-1.214974000	0.681022000
1	-4.621100000	-1.739126000	2.040723000
1	-5.510468000	-2.914599000	1.096063000
6	-2.916229000	-3.356245000	0.731098000
1	-3.557248000	-4.235941000	0.817888000
1	-2.562570000	-3.115703000	1.731087000
1	-2.057607000	-3.633203000	0.123256000
6	-3.626879000	0.545360000	-1.175045000
6	-3.392746000	0.194617000	-2.649180000
1	-4.011516000	-0.637339000	-2.976473000
1	-2.357917000	-0.068652000	-2.860152000
1	-3.643144000	1.053101000	-3.274901000
6	-3.026350000	1.921772000	-0.890292000
1	-3.397685000	2.637198000	-1.626650000
1	-1.943470000	1.905218000	-0.958703000
1	-3.306271000	2.294203000	0.096047000
6	-5.125327000	0.654712000	-0.911328000
1	-5.347091000	0.897496000	0.128532000
1	-5.679140000	-0.241216000	-1.181245000
1	-5.524937000	1.469897000	-1.519509000
15	1.942212000	-0.407701000	-0.165747000
6	3.938740000	-2.128168000	-1.190451000
1	4.458803000	-2.452948000	-2.093426000
1	4.695289000	-1.996792000	-0.420687000
1	3.271453000	-2.938658000	-0.895826000
6	2.420027000	-1.134738000	-2.823696000
1	1.894551000	-2.084723000	-2.752035000
1	1.719911000	-0.355718000	-3.096950000
1	3.147178000	-1.220692000	-3.633920000
6	1.978963000	-0.118333000	2.669500000
1	1.969958000	0.967997000	2.692095000
1	0.953152000	-0.476809000	2.673483000
1	2.438482000	-0.448147000	3.602827000
6	4.181279000	0.012520000	1.598838000
1	4.573663000	-0.104048000	2.611887000
1	4.914206000	-0.415982000	0.920641000

1	4.117514000	1.083287000	1.399519000
6	0.750796000	1.713203000	-1.505847000
6	-0.375917000	-2.193836000	-1.522528000
8	-0.468055000	-3.086375000	-2.255261000
25	-0.337135000	-0.842242000	-0.345374000
6	-0.292485000	-1.954312000	1.031520000
8	-0.199085000	-2.639911000	1.969302000
7	-0.067380000	0.718884000	-1.753392000
6	0.313471000	3.150915000	-1.729083000
1	-0.605119000	3.290577000	-1.147385000
1	-0.003386000	3.236545000	-2.771366000
6	1.221839000	4.288784000	-1.336816000
6	0.621654000	5.454221000	-0.854373000
6	2.615569000	4.261878000	-1.403607000
6	1.370974000	6.547396000	-0.454540000
6	3.370459000	5.353071000	-0.994314000
6	2.756008000	6.499821000	-0.518470000
1	-0.460380000	5.492892000	-0.784582000
1	3.115632000	3.376585000	-1.775672000
1	0.872850000	7.435635000	-0.086762000
1	4.450485000	5.301625000	-1.052610000
1	3.348625000	7.348253000	-0.201745000
1	0.647754000	1.549401000	-4.148259000
8	-0.262655000	1.508547000	-4.556126000
1	-0.664447000	0.902863000	-2.554866000
1	-0.155919000	0.995298000	-5.359306000
8	2.006408000	1.804907000	-3.135033000
1	2.139970000	2.732257000	-3.361910000

7. NMR Spectra of the Synthesized Compounds

7.1 ^{1}H NMR and ^{13}C NMR spectra of the amide products and synthesized manganese complex

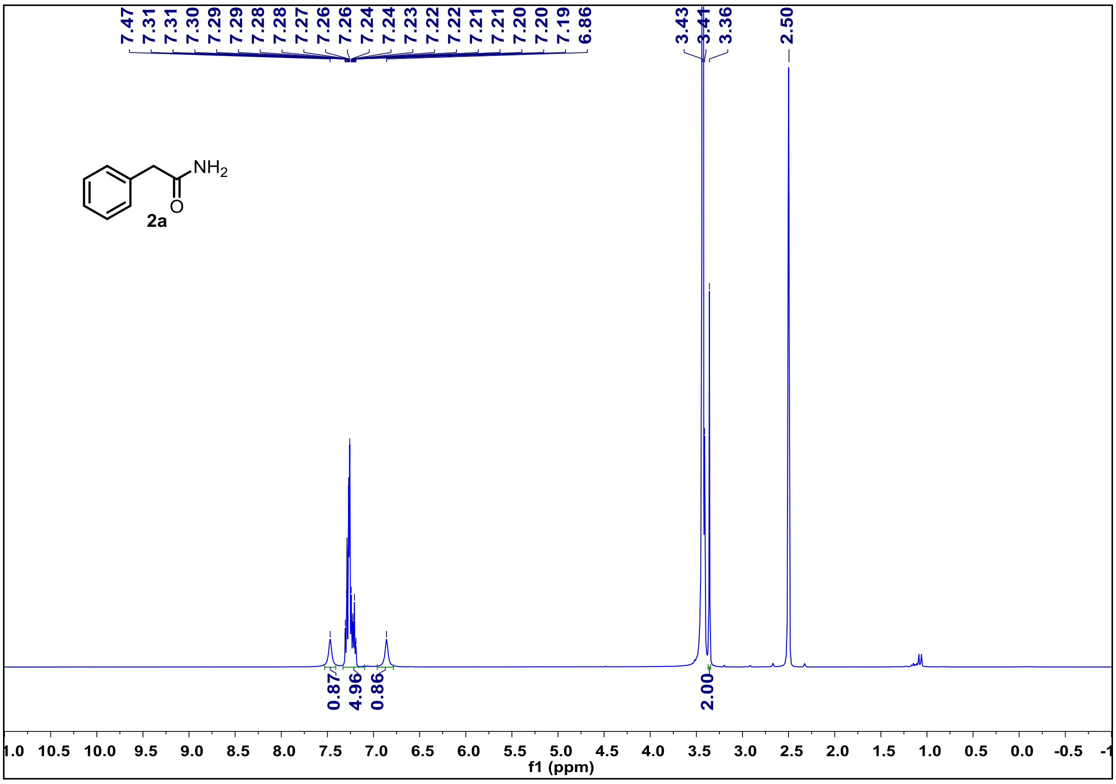

Figure S11. ^{1}H NMR spectrum of **2a** (400 MHz, DMSO-d6, 298 K).

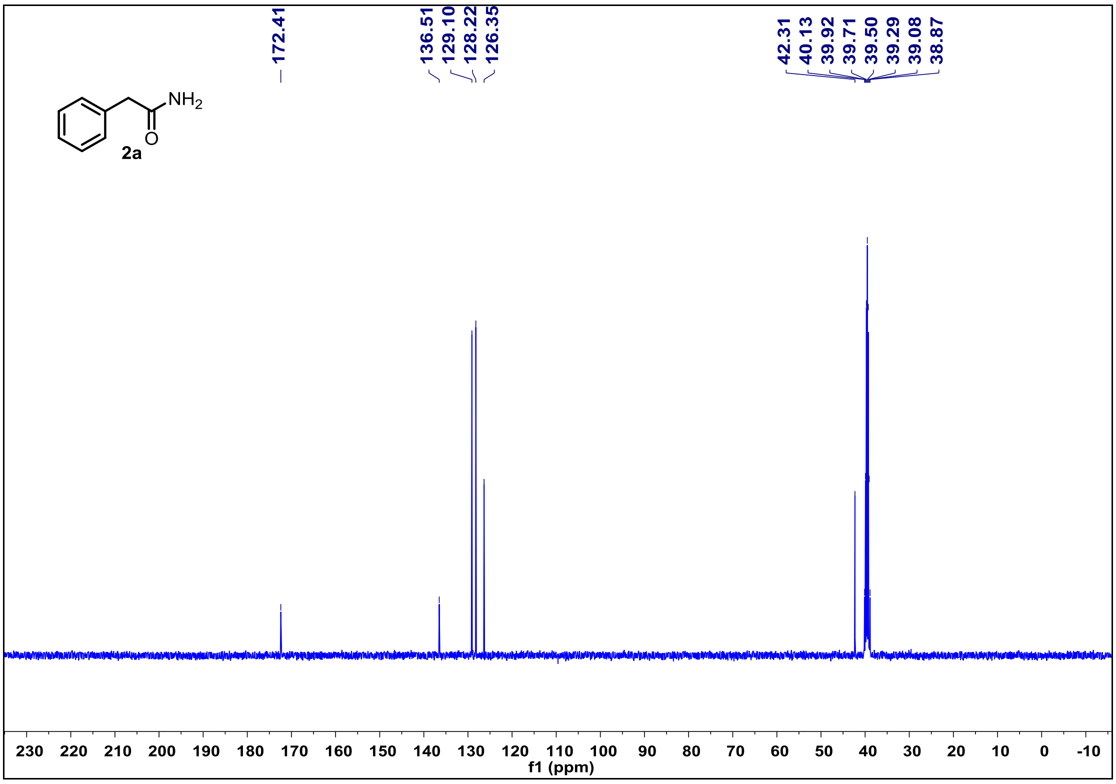

Figure S12. $^{13}C\{^{1}H\}$ NMR spectrum of of **2a** (100 MHz, DMSO-d6, 298 K).

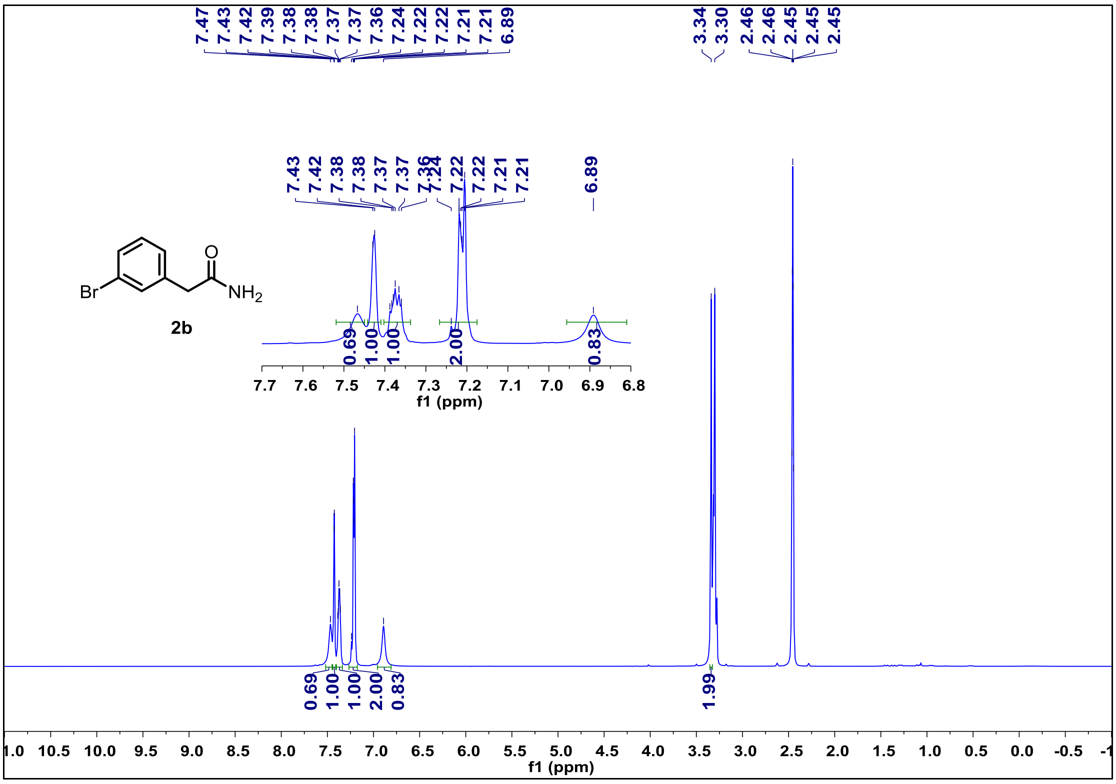

Figure S13. ^{1}H NMR spectrum of **2b** (400 MHz, DMSO-d6, 298 K).

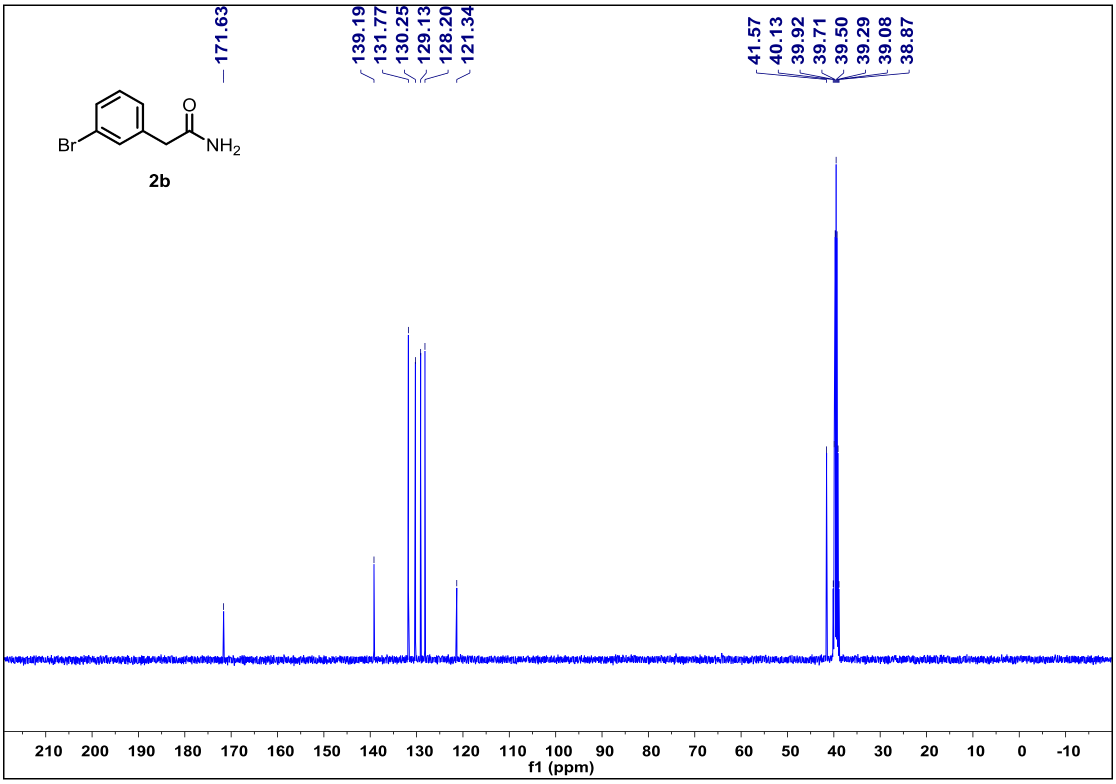

Figure S14. ^{13}C{^{1}H} NMR spectrum of 2**b** (100 MHz, DMSO-d6, 298 K).

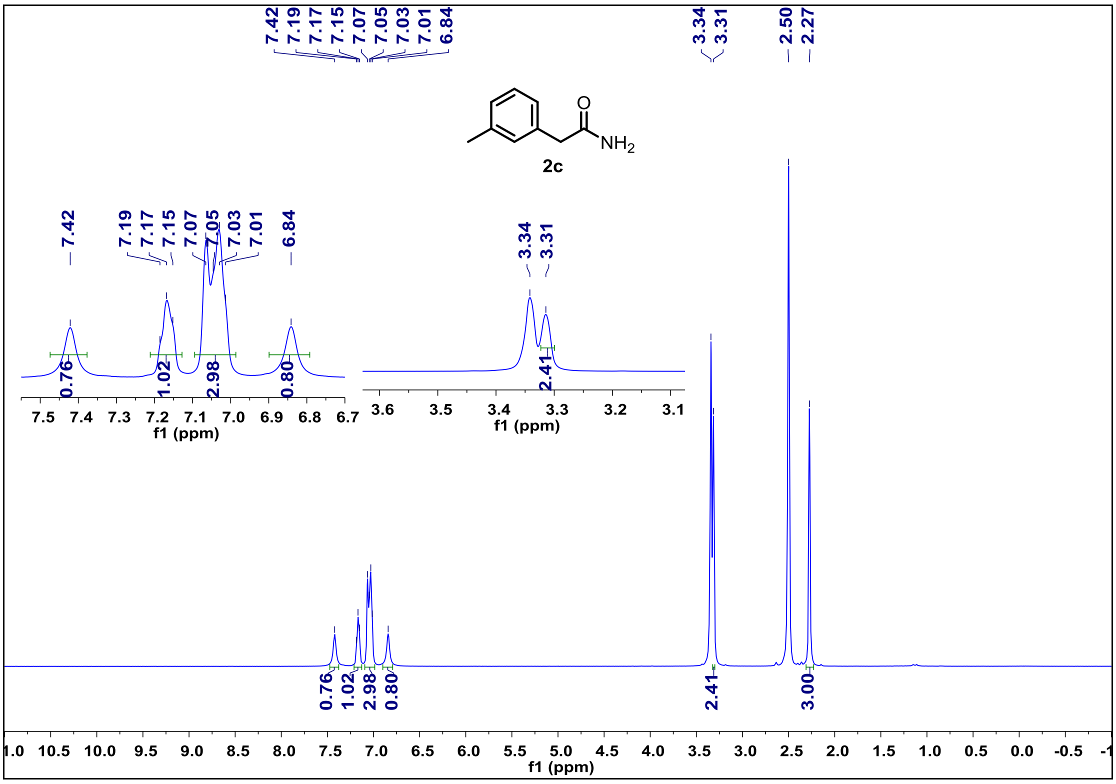

Figure S15. ^{1}H NMR spectrum of **2c** (500 MHz, DMSO-d6, 298 K).

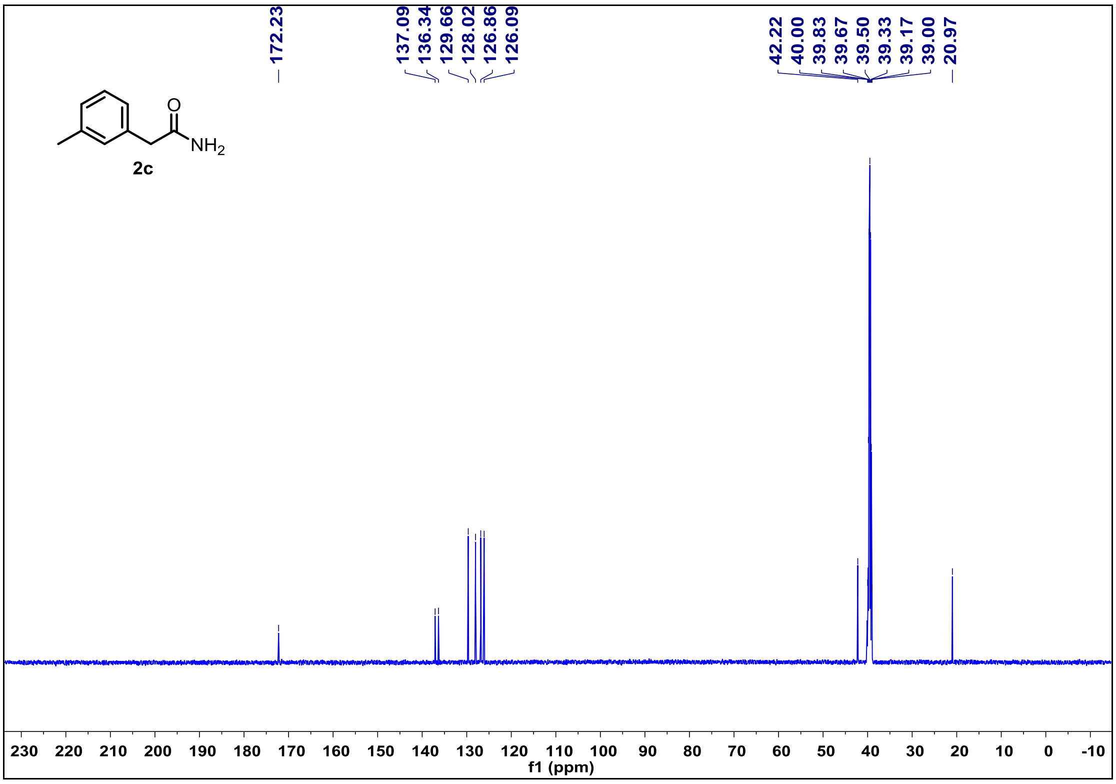

Figure S16. ^{13}C{^{1}H} NMR spectrum of **2c** (125 MHz, DMSO-d6, 298 K).

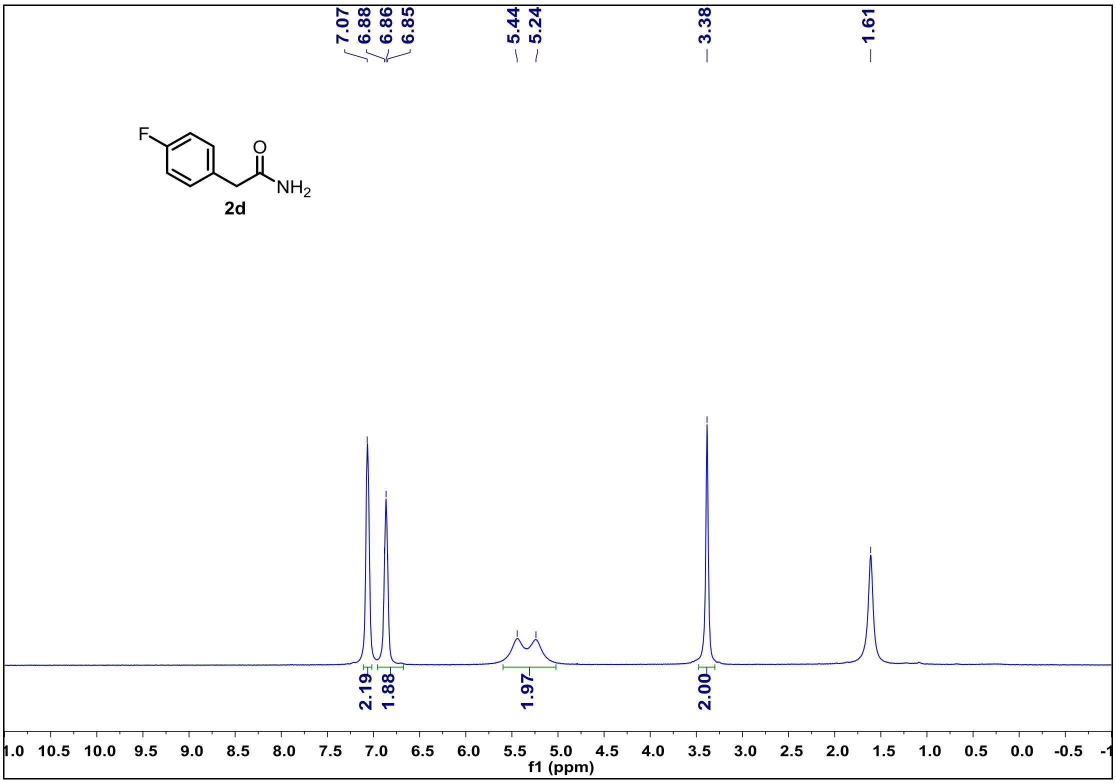

Figure S17. ^{1}H NMR spectrum of **2d** (500 MHz, $CDCl_3$, 298 K).

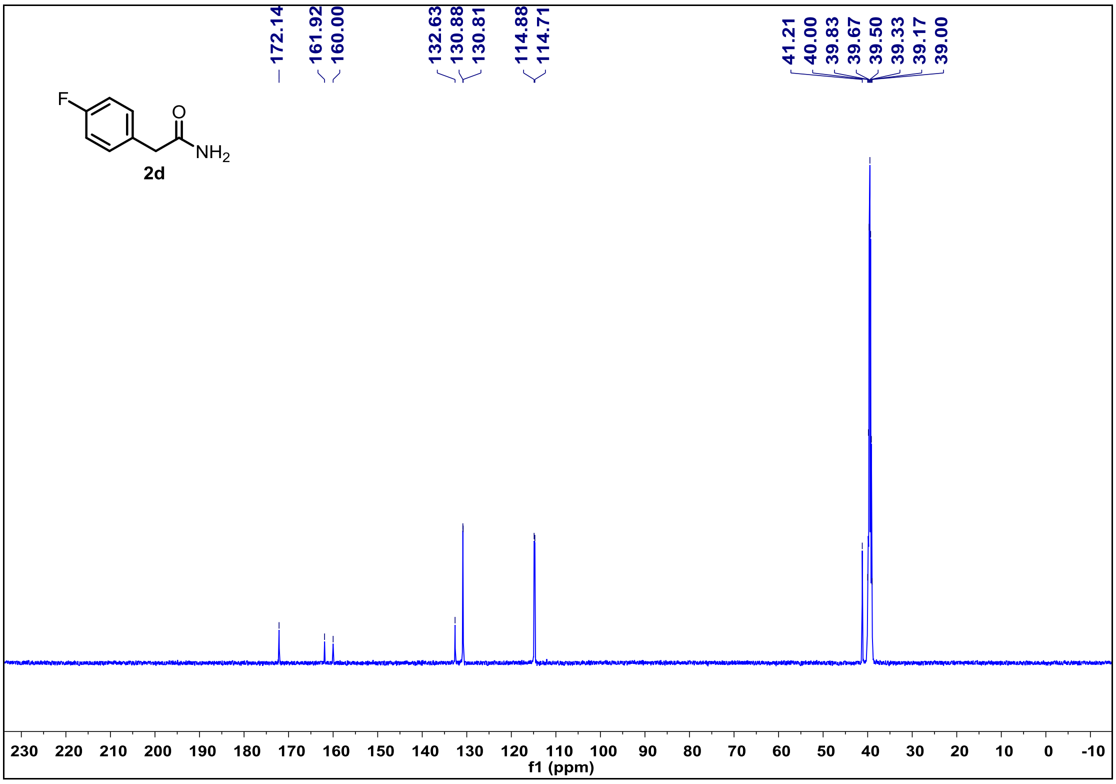

Figure S18. $^{13}C\{^1H\}$ NMR spectrum of **2d** (125 MHz, DMSO-d6, 298 K).

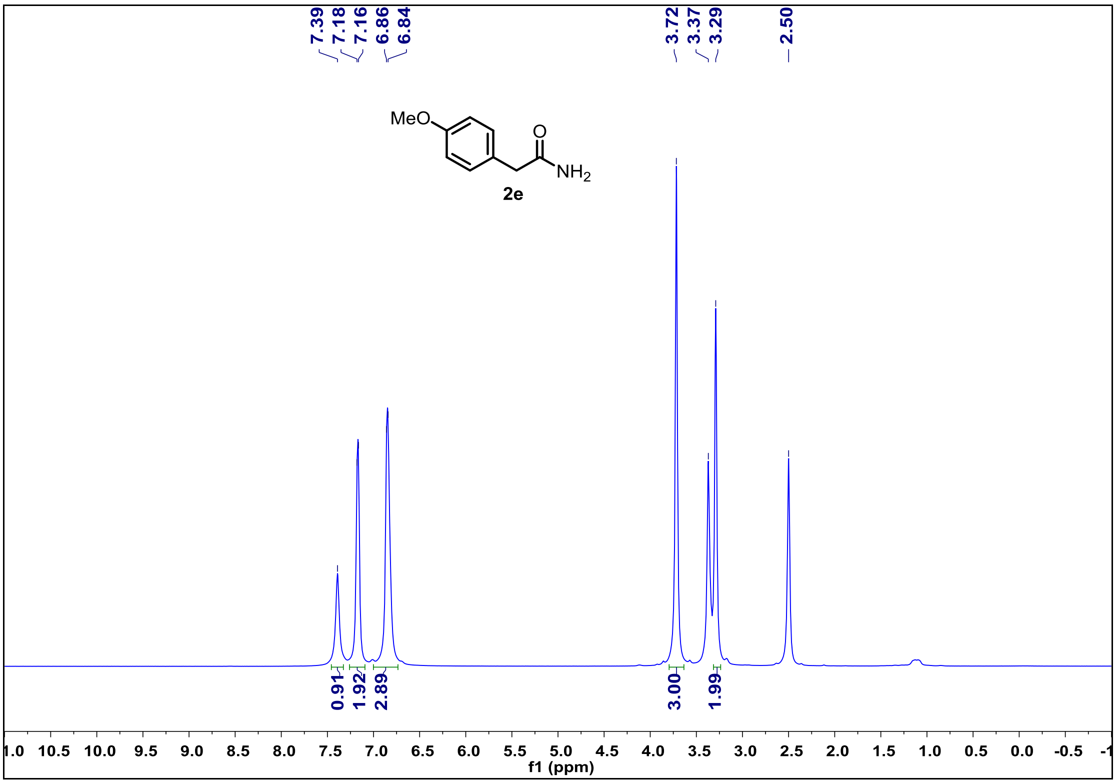

Figure S19. ^{1}H NMR spectrum of **2e** (500 MHz, DMSO-d6, 298 K).

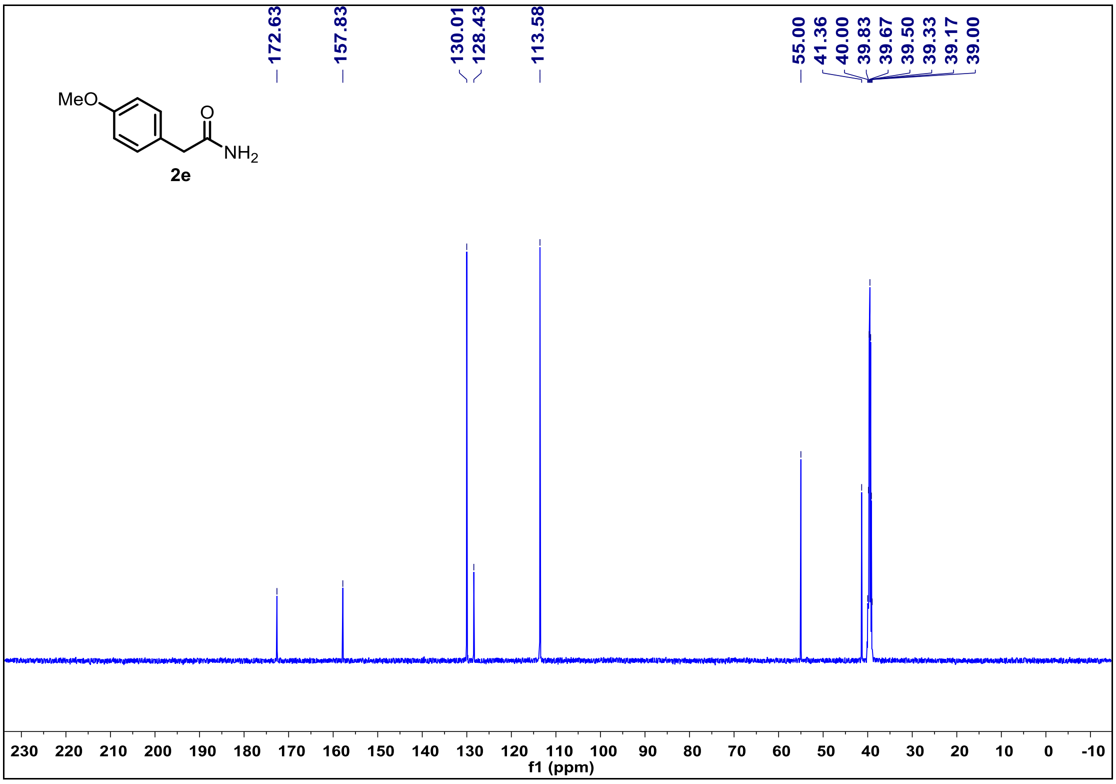

Figure S20. ^{13}C{^{1}H} NMR spectrum of **2e** (125 MHz, DMSO-d6, 298 K).

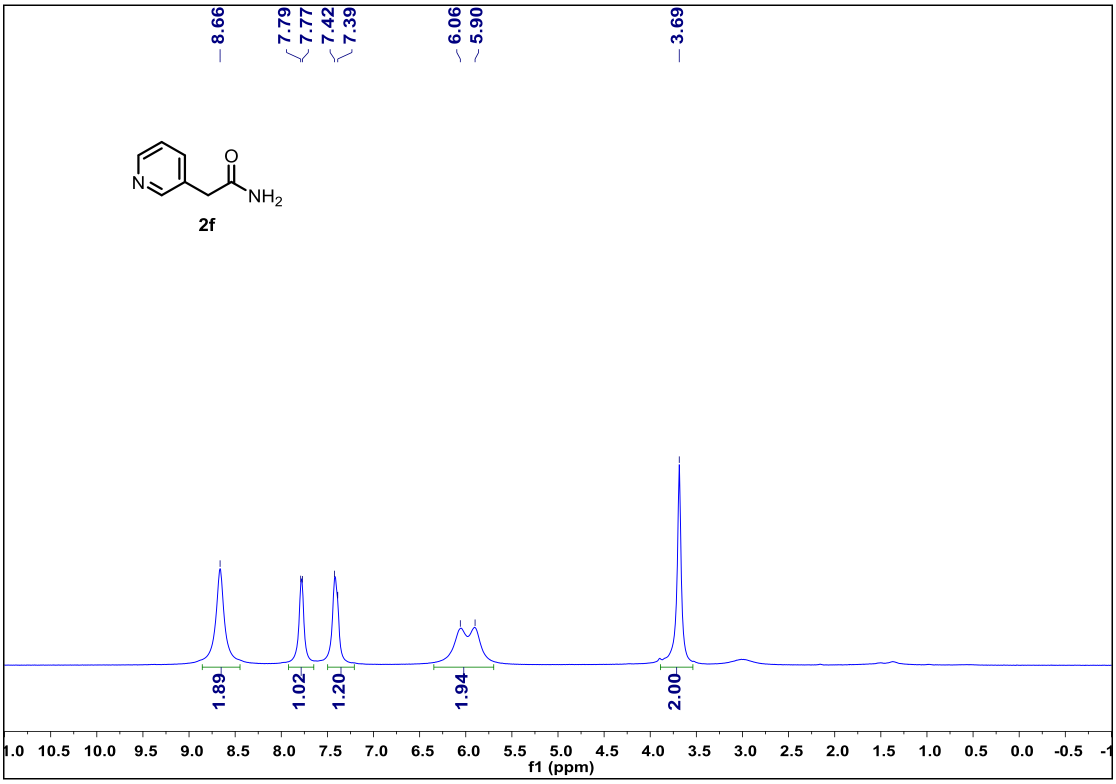

Figure S21. ^{1}H NMR spectrum of **2f** (400 MHz, $CDCl_3$, 298 K).

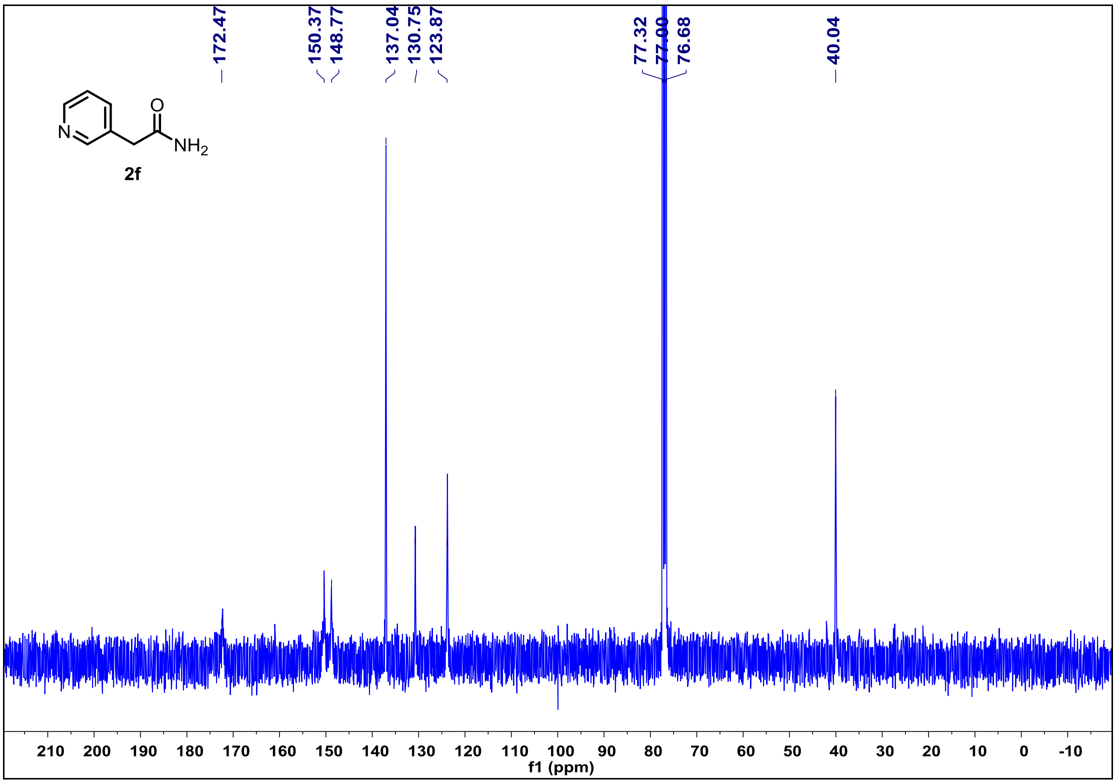

Figure S22. $^{13}C\{^{1}H\}$ NMR spectrum of **2f** (100 MHz, $CDCl_3$, 298 K).

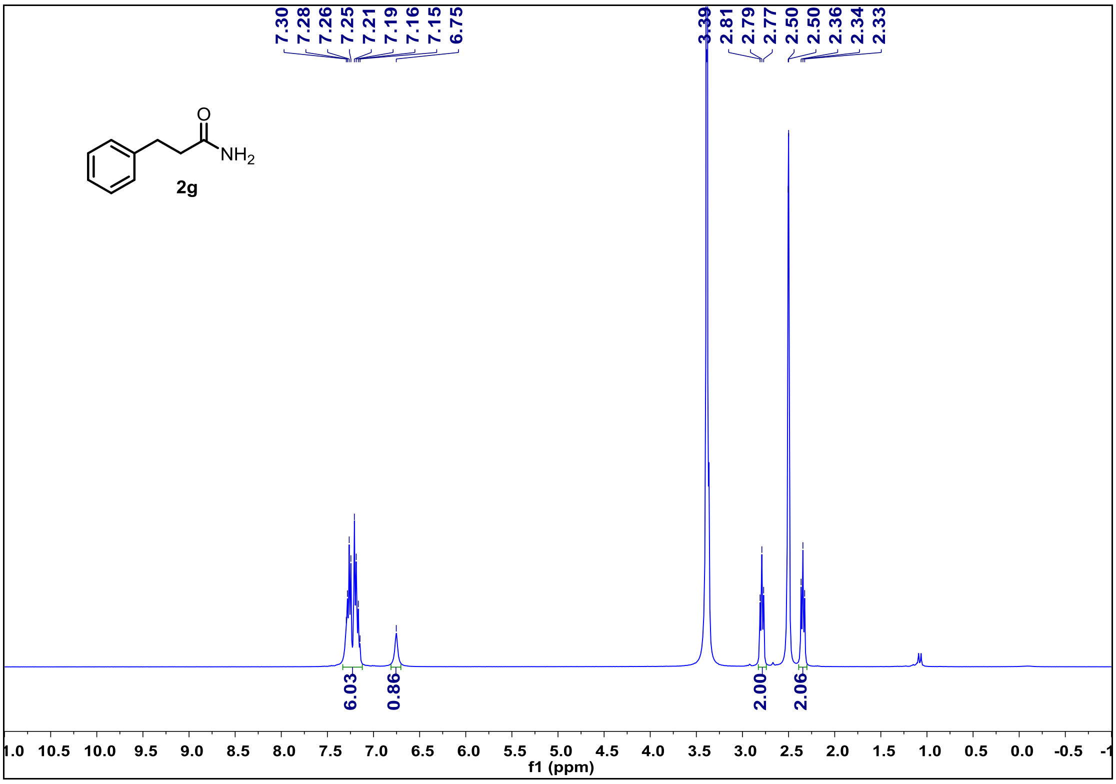

Figure S23. ^{1}H NMR spectrum of **2g** (400 MHz, DMSO-d6, 298 K).

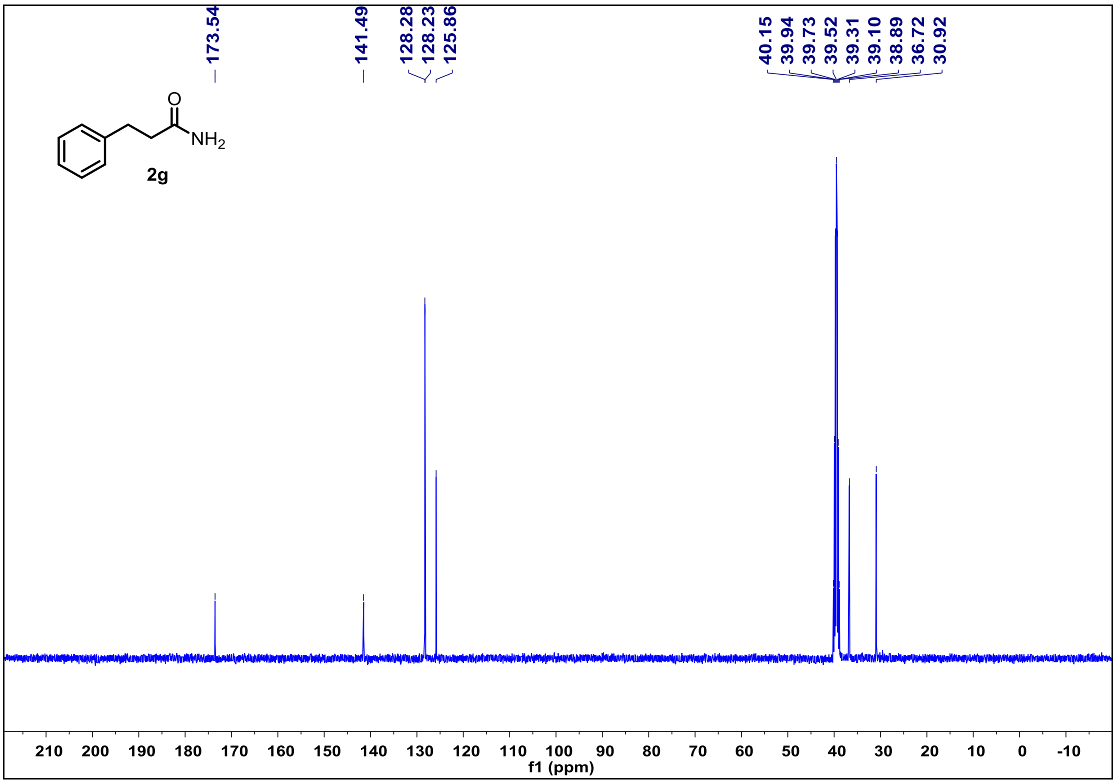

Figure S24. ^{13}C{^{1}H} NMR spectrum of **2g** (100 MHz, DMSO-d6, 298 K).

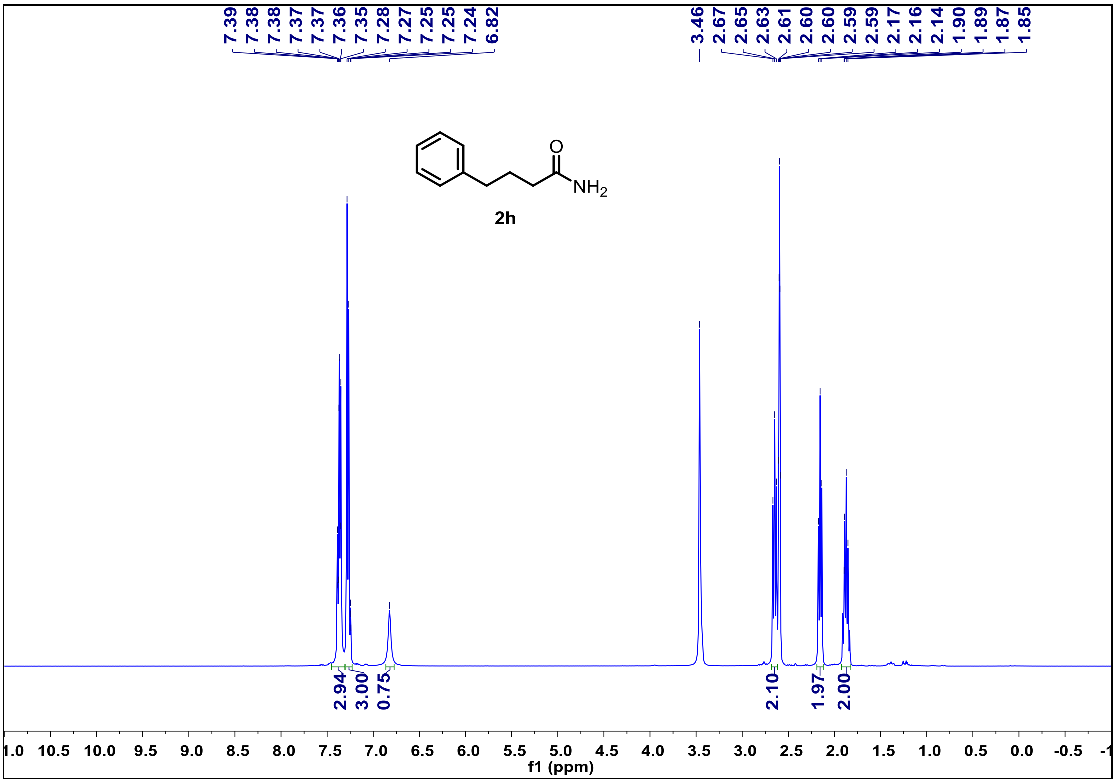

Figure S25. ^{1}H NMR spectrum of **2h** (400 MHz, DMSO-d6, 298 K).

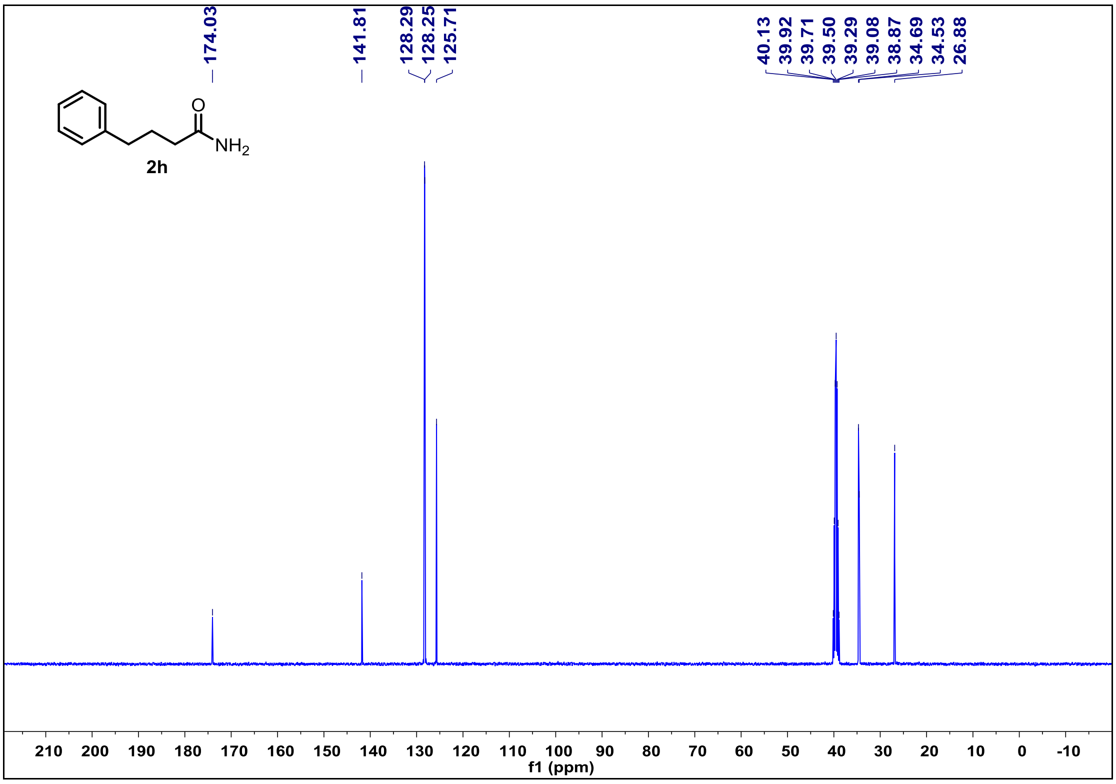

Figure S26. ^{13}C{^{1}H} NMR spectrum of **2h** (100 MHz, DMSO-d6, 298 K).

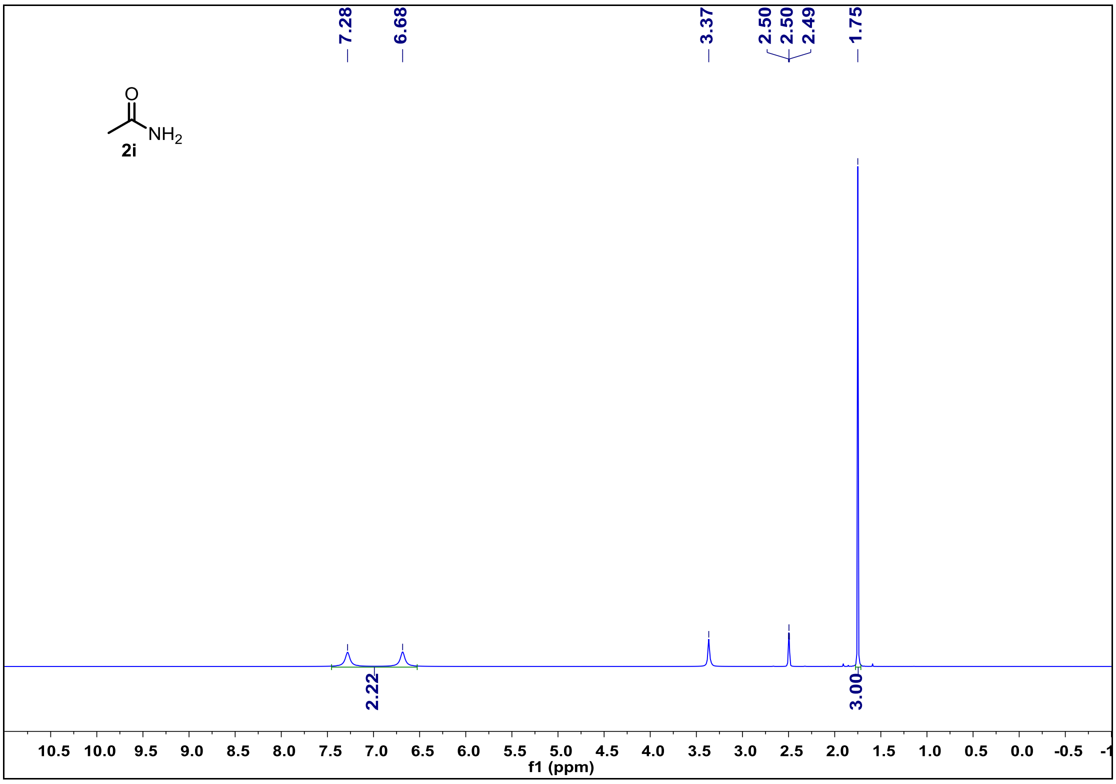

Figure S27. ^{1}H NMR spectrum of **2i** (400 MHz, DMSO-d6, 298 K).

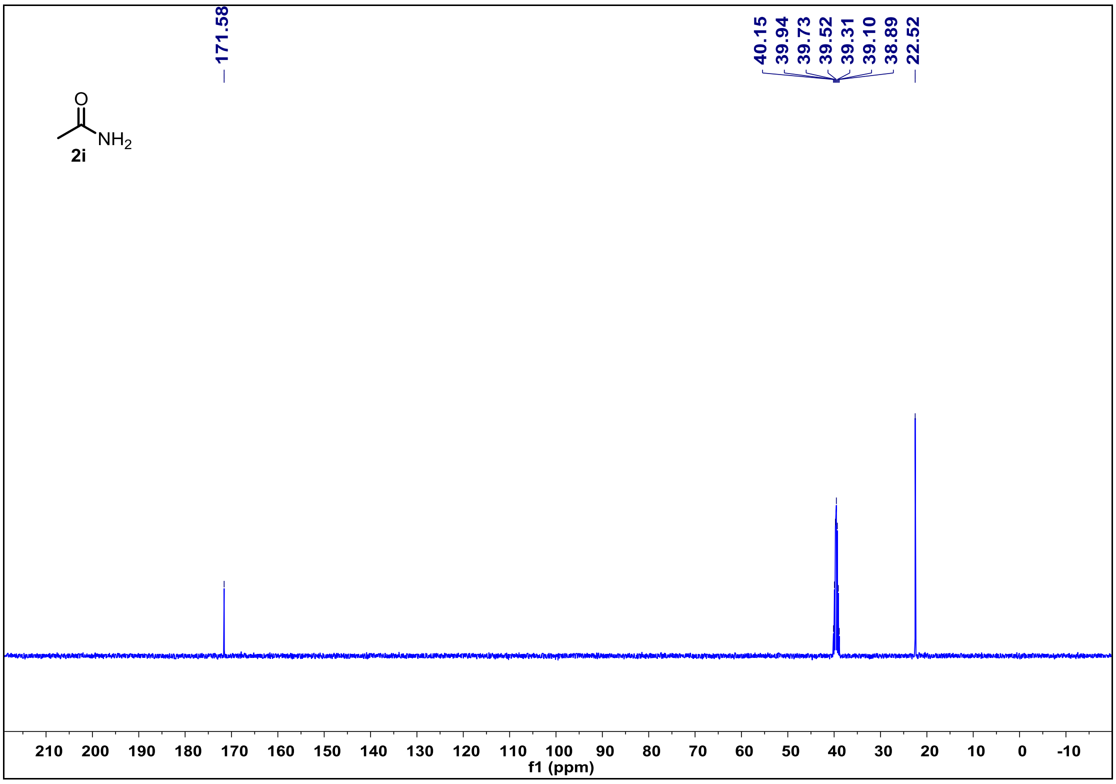

Figure S28. $^{13}C\{^{1}H\}$ NMR spectrum of **2i** (100 MHz, DMSO-d6, 298 K).

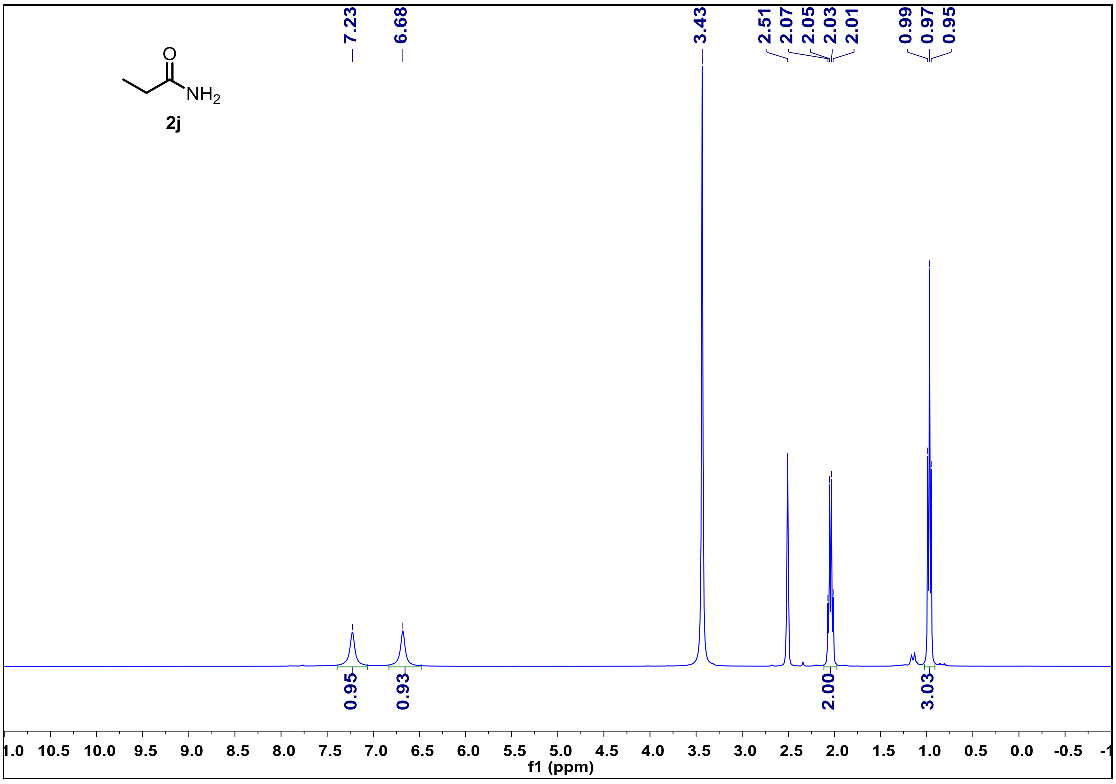

Figure S29. ^{1}H NMR spectrum of **2j** (400 MHz, DMSO-d6, 298 K).

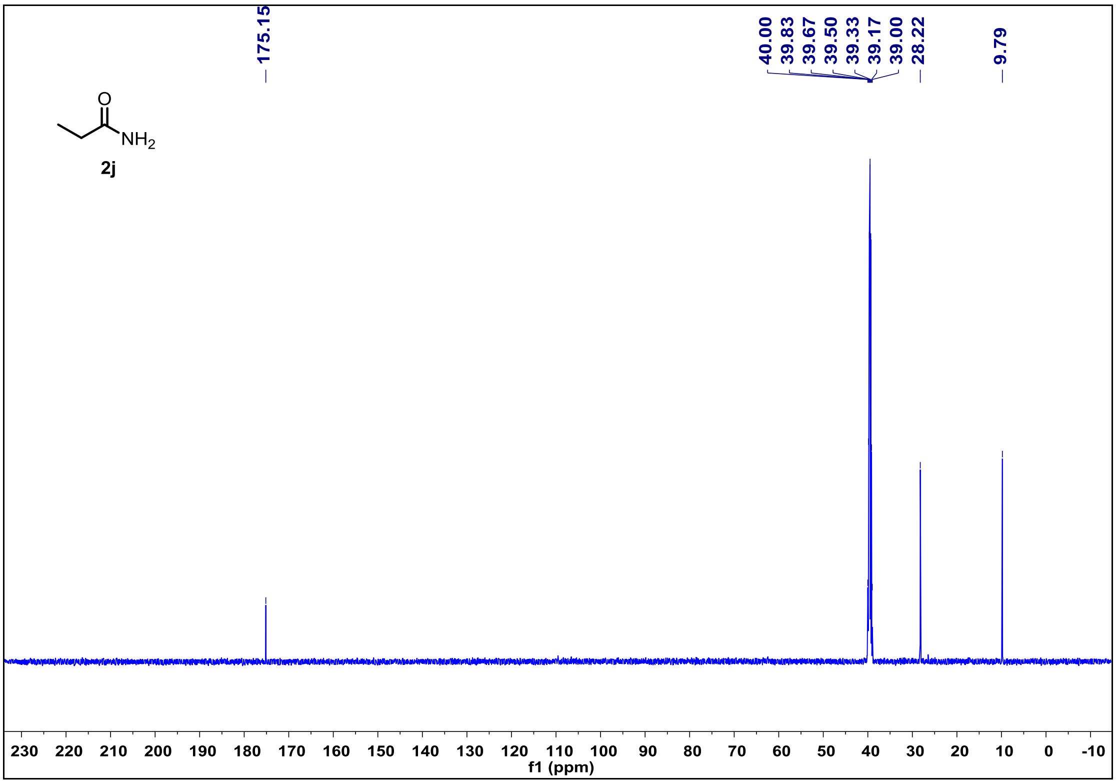

Figure S30. $^{13}C\{^{1}H\}$ NMR spectrum of **2j** (125 MHz, DMSO-d6, 298 K).

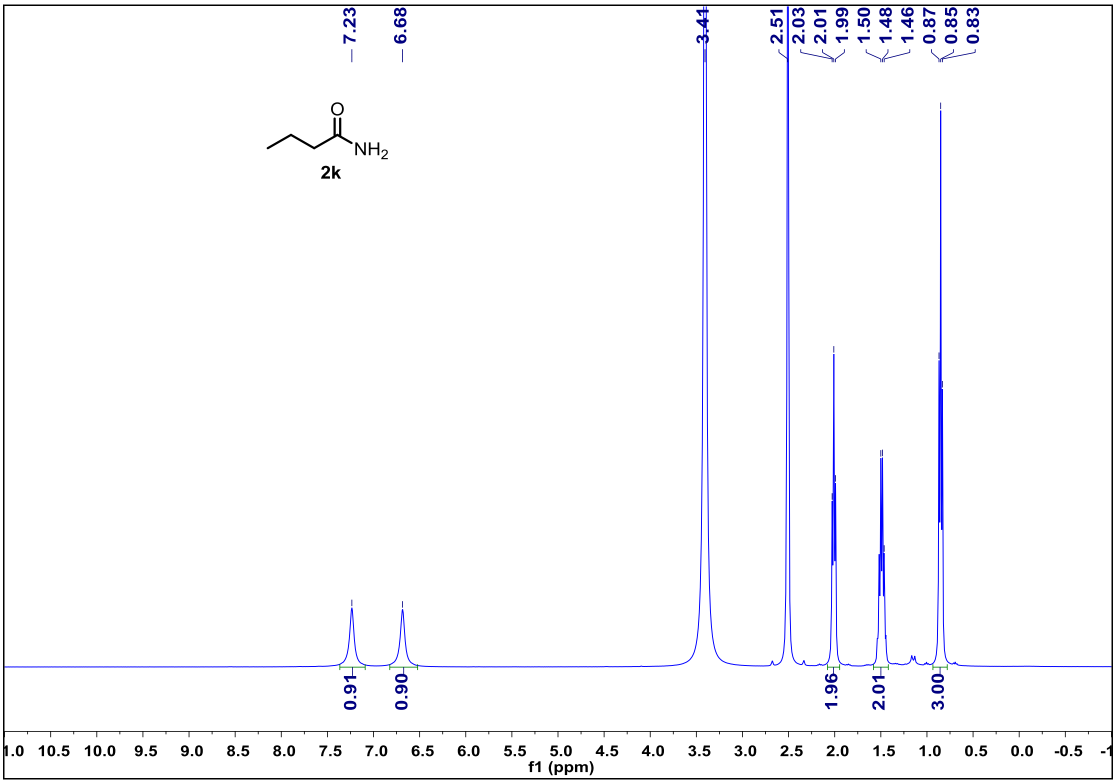

Figure S31. ^{1}H NMR spectrum of **2k** (400 MHz, DMSO-d6, 298 K).

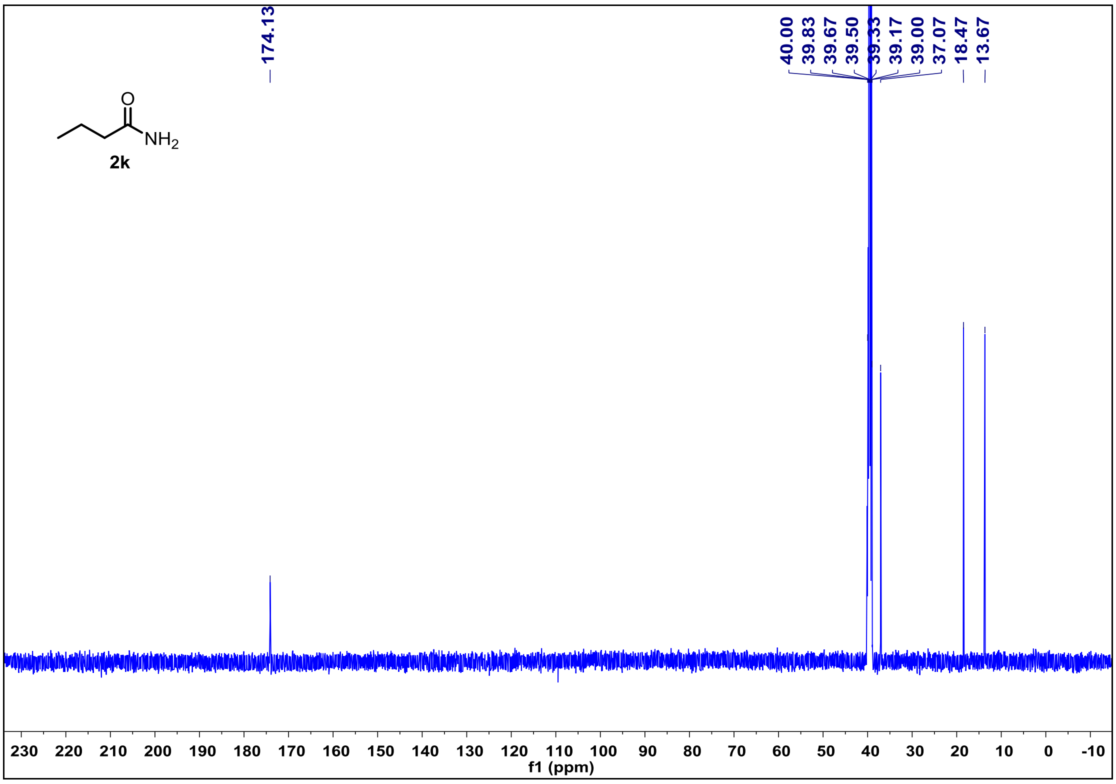

Figure S32. $^{13}C\{^{1}H\}$ NMR spectrum of **2k** (125 MHz, DMSO-d6, 298 K).

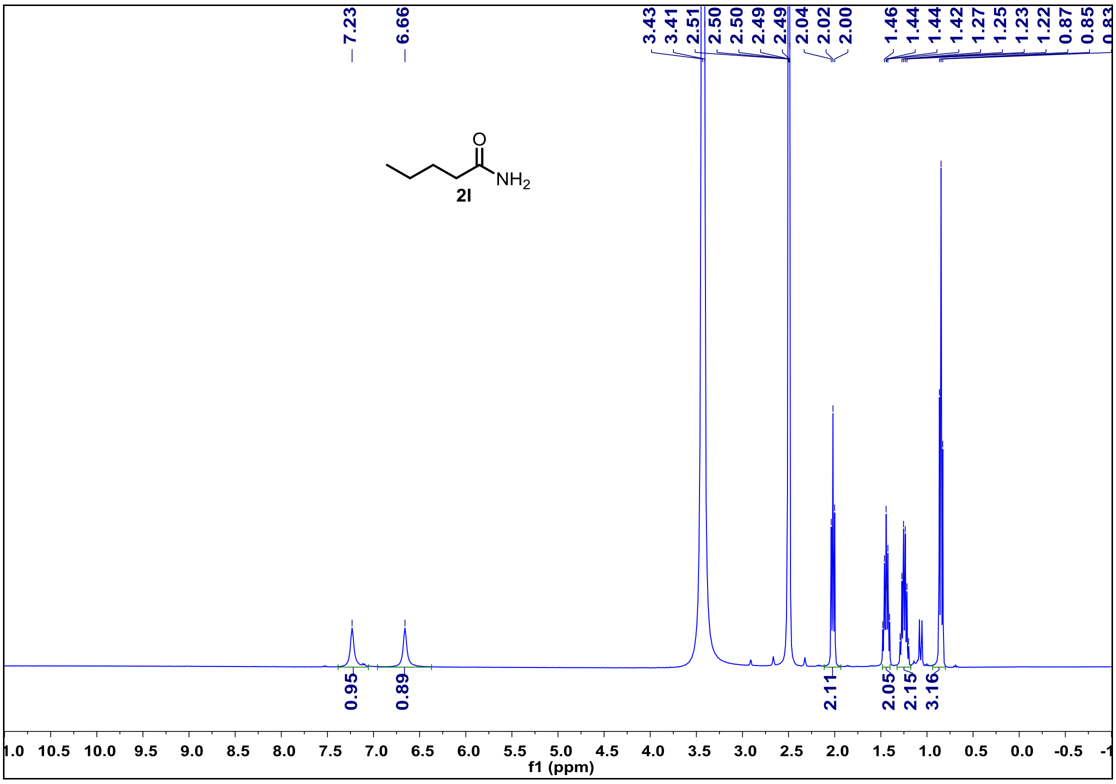

Figure S33. ^{1}H NMR spectrum of **2l** (400 MHz, DMSO-d6, 298 K).

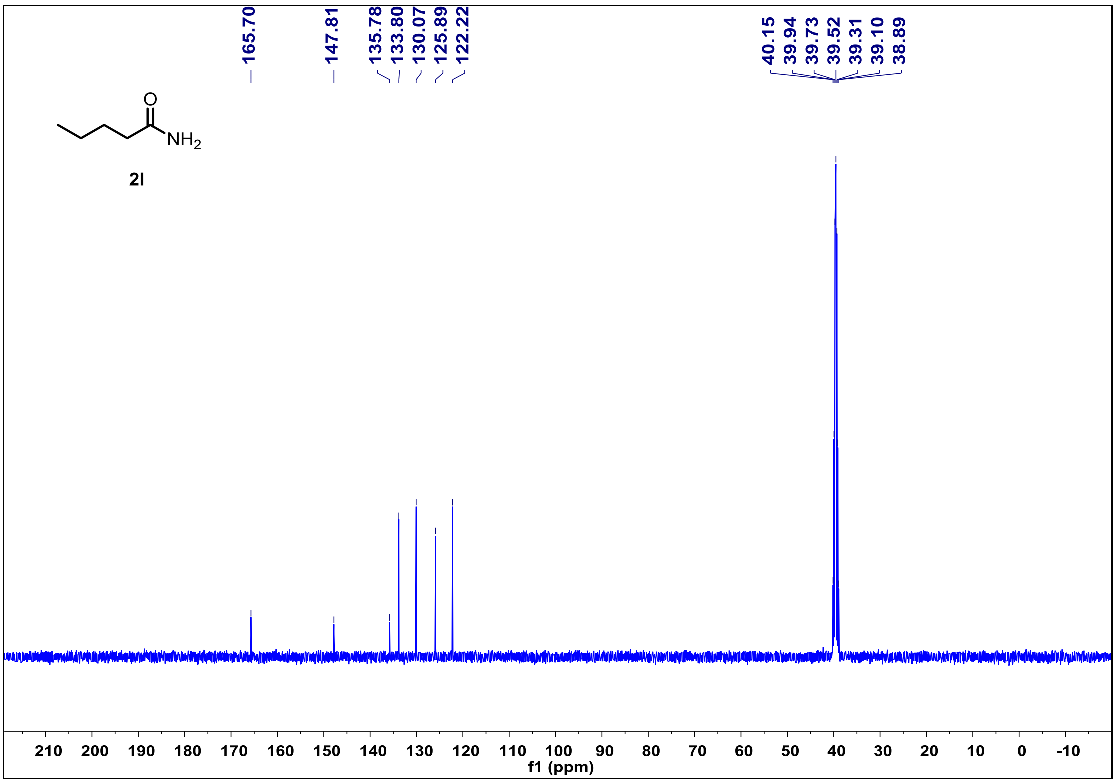

Figure S34. $^{13}C\{^{1}H\}$ NMR spectrum of **2l** (100 MHz, DMSO-d6, 298 K).

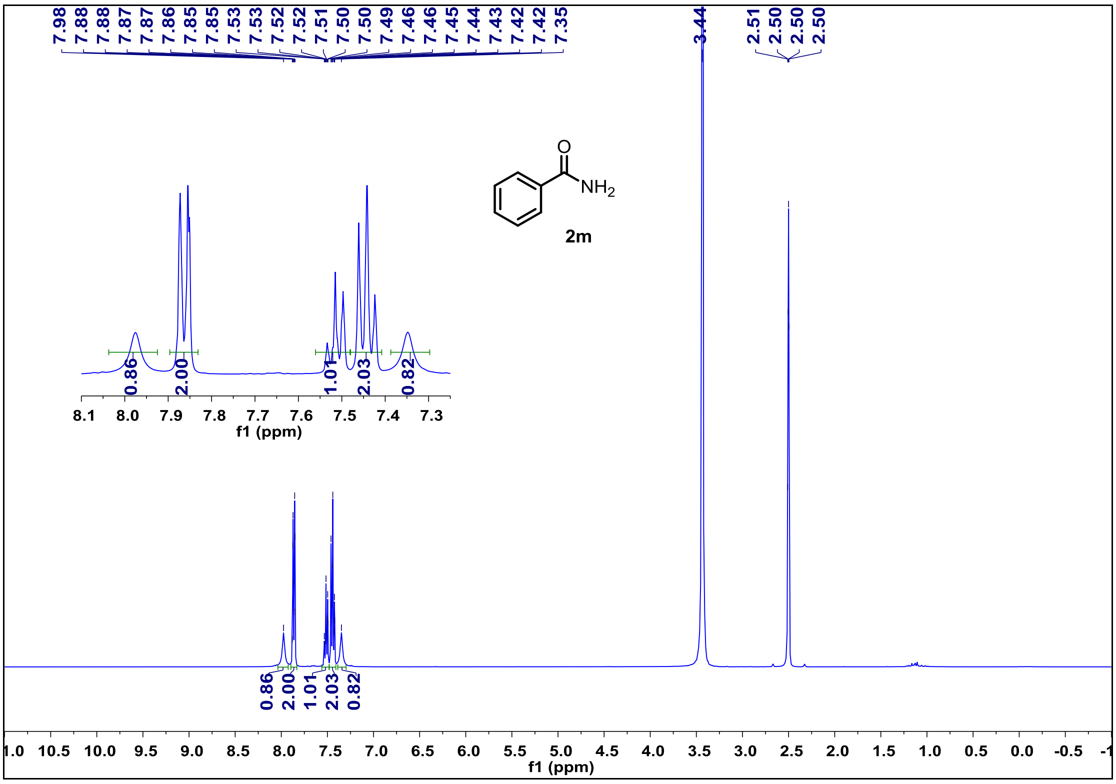

Figure S35. ^{1}H NMR spectrum of **2m** (400 MHz, DMSO-d6, 298 K).

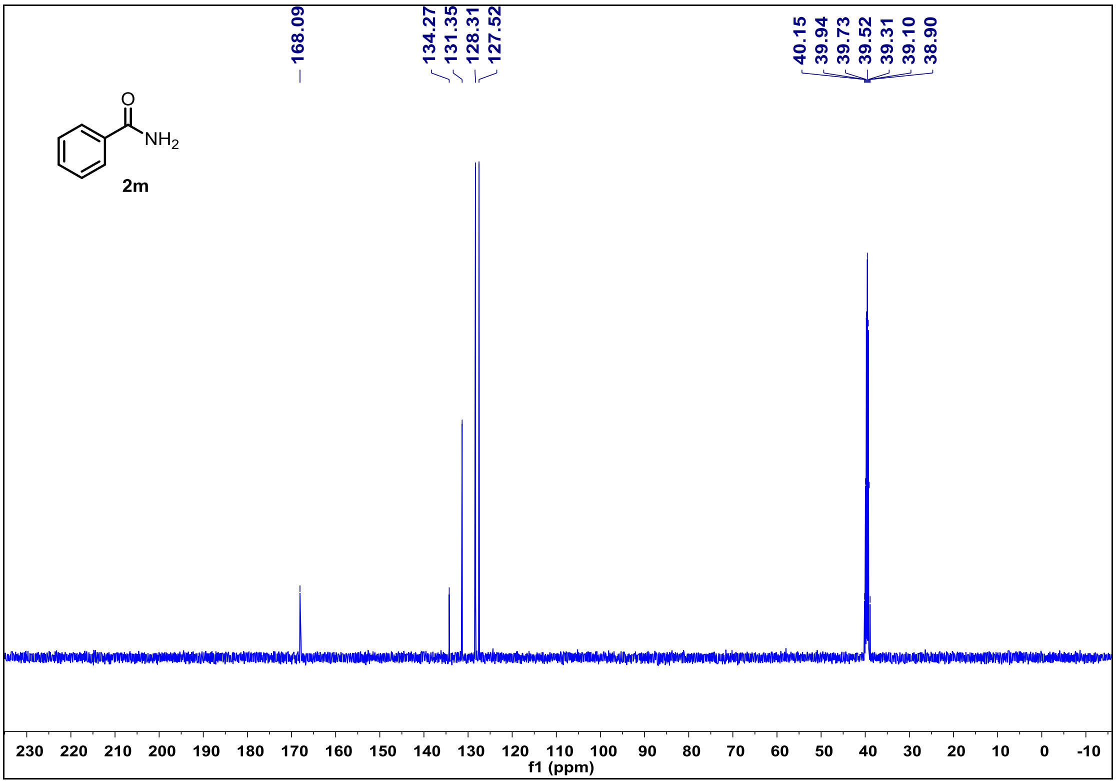

Figure S36. $^{13}C\{^{1}H\}$ NMR spectrum of **2m** (100 MHz, DMSO-d6, 298 K).

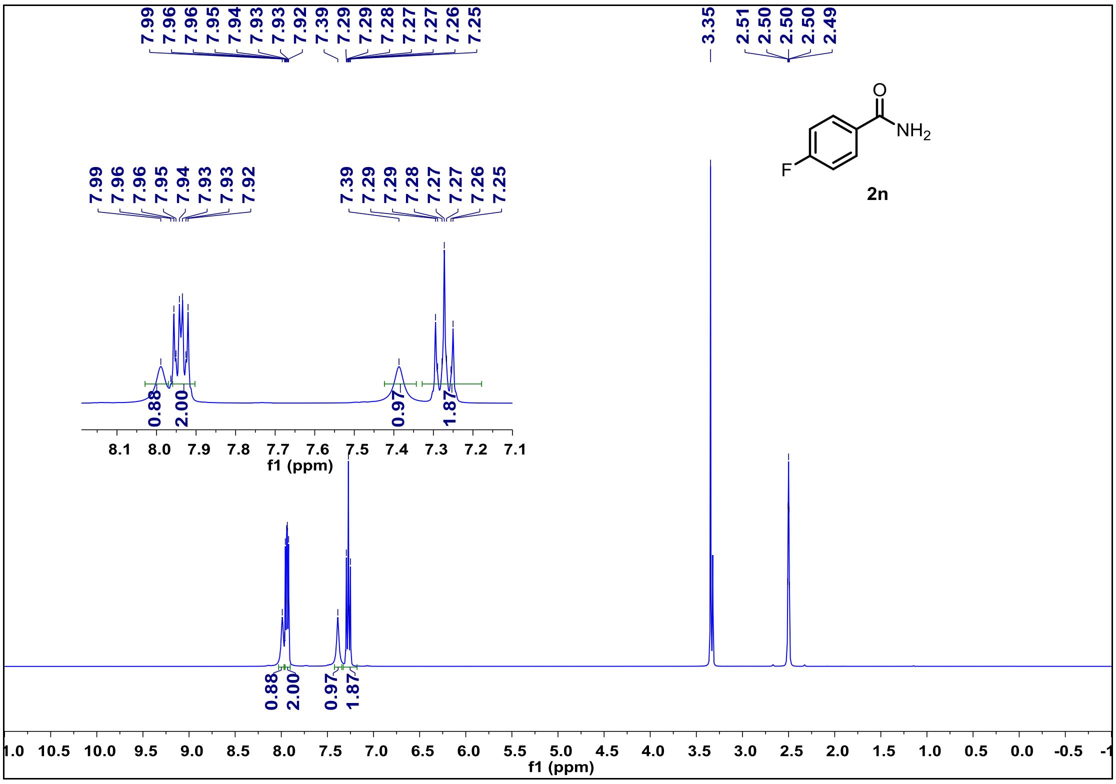

Figure S37. ^{1}H NMR spectrum of **2n** (400 MHz, DMSO-d6, 298 K).

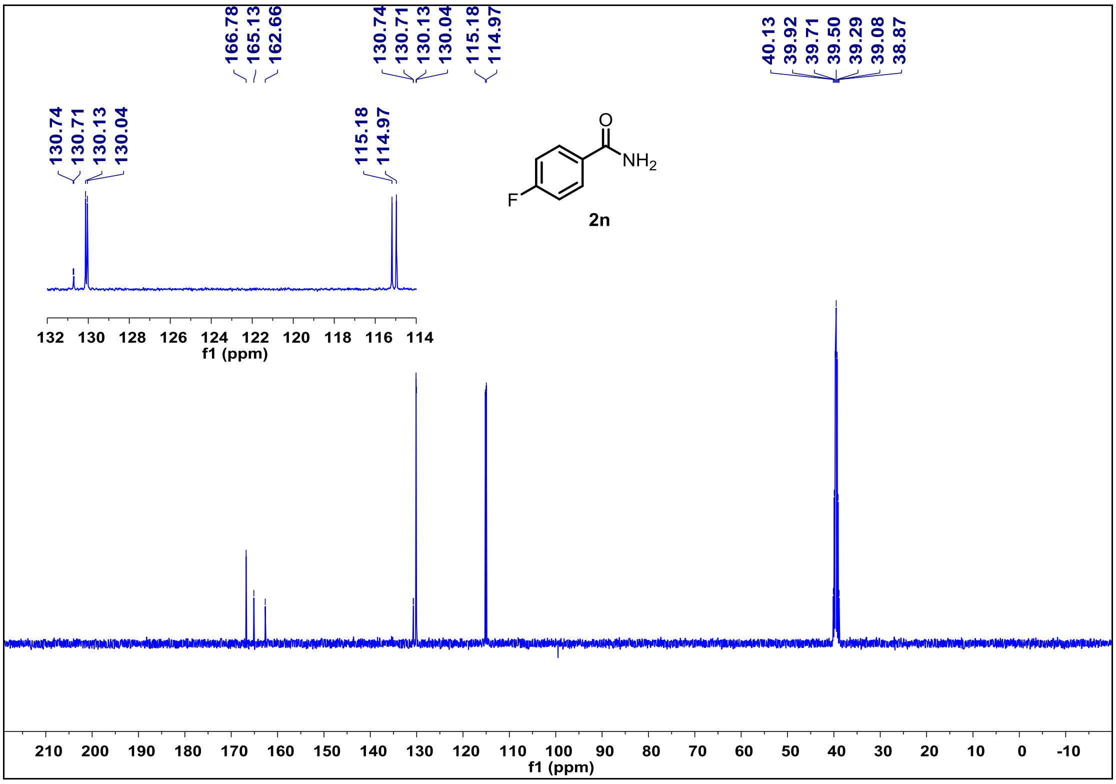

Figure S38. $^{13}C\{^{1}H\}$ NMR spectrum of **2n** (100 MHz, DMSO-d6, 298 K).

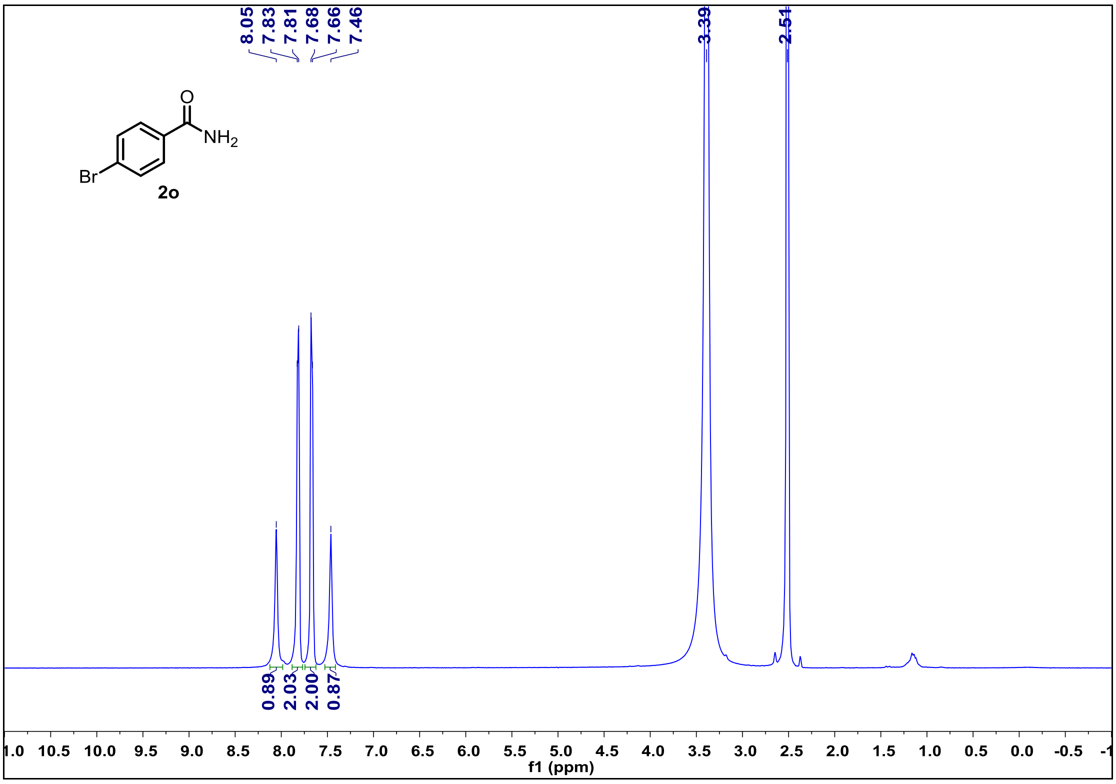

Figure S39. ^{1}H NMR spectrum of **2o** (500 MHz, DMSO-d6, 298 K).

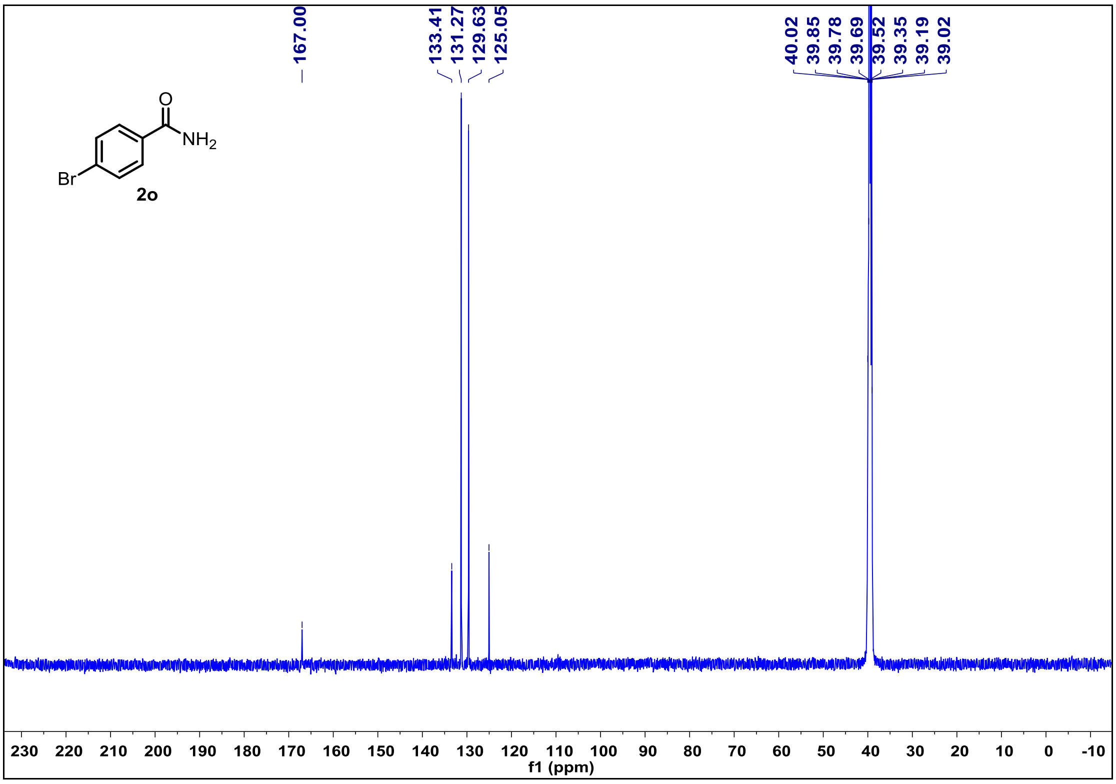

Figure S40. $^{13}C\{^{1}H\}$ NMR spectrum of **2o** (125 MHz, DMSO-d6, 298 K).

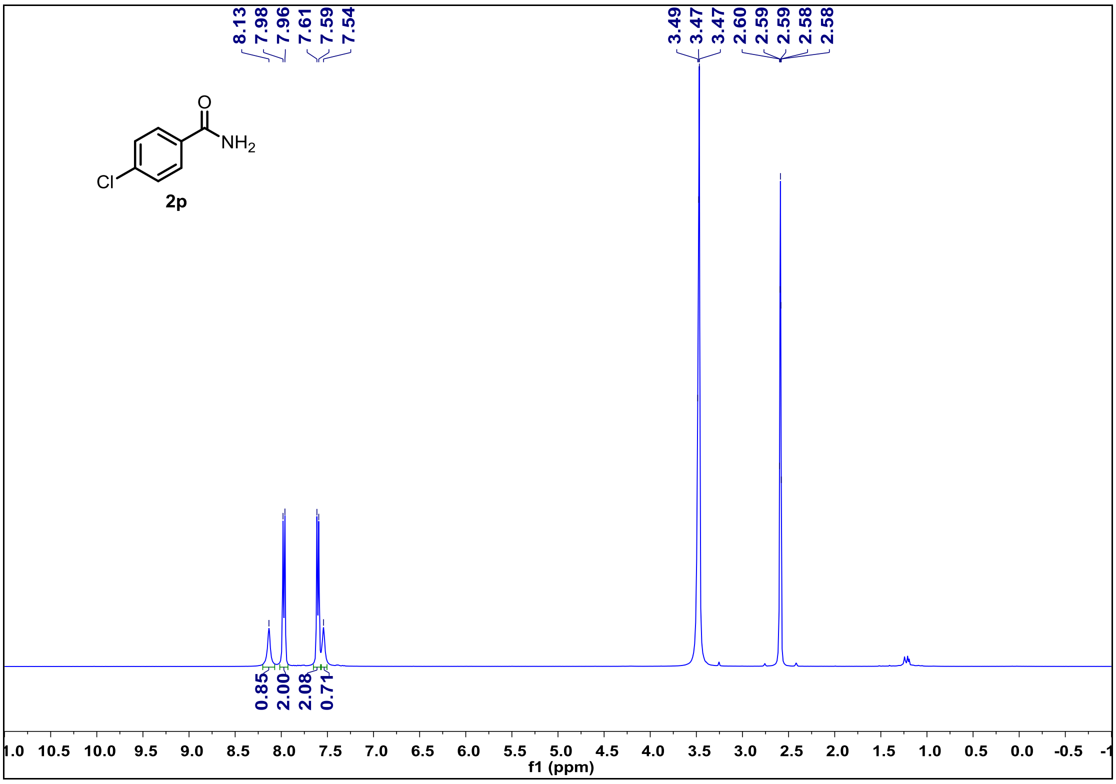

Figure S41. ^{1}H NMR spectrum of **2p** (400 MHz, DMSO-d6, 298 K).

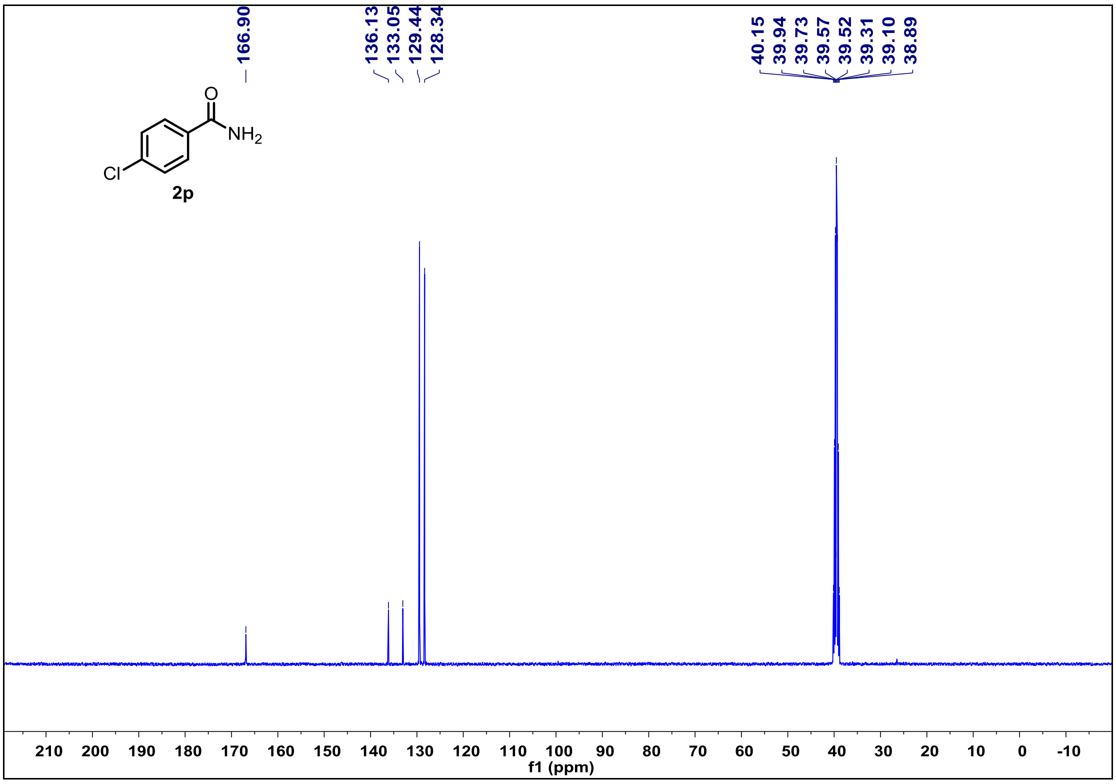

Figure S42. ^{13}C{^{1}H} NMR spectrum of **2p** (100 MHz, DMSO-d6, 298 K).

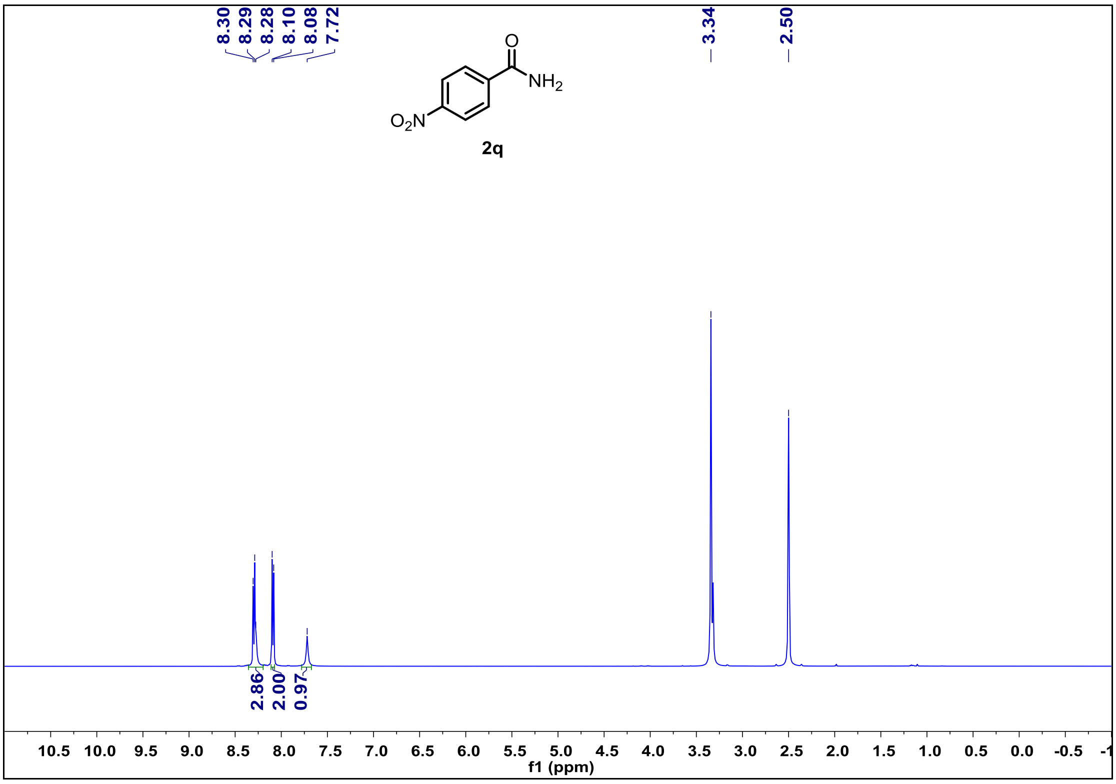

Figure S43. ^{1}H NMR spectrum of **2q** (500 MHz, DMSO-d6, 298 K).

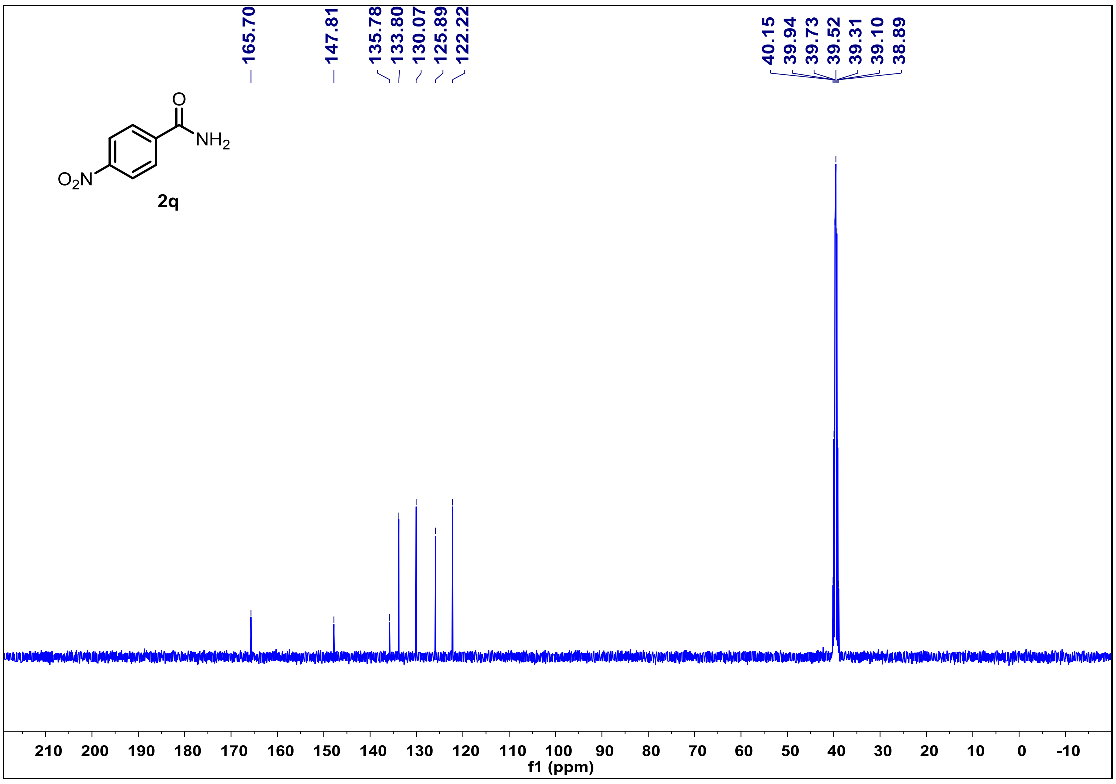

Figure S44. ^{13}C{^{1}H} NMR spectrum of **2q** (125 MHz, DMSO-d6, 298 K).

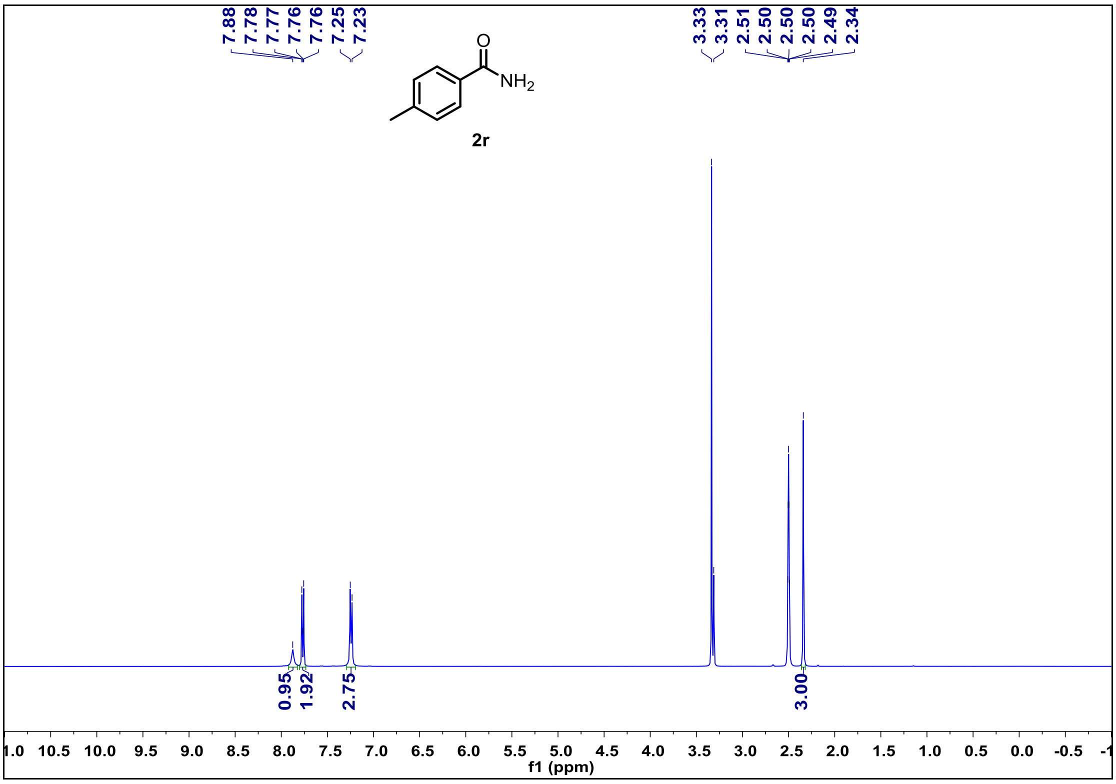

Figure S45. ^{1}H NMR spectrum of **2r** (400 MHz, DMSO-d6, 298 K).

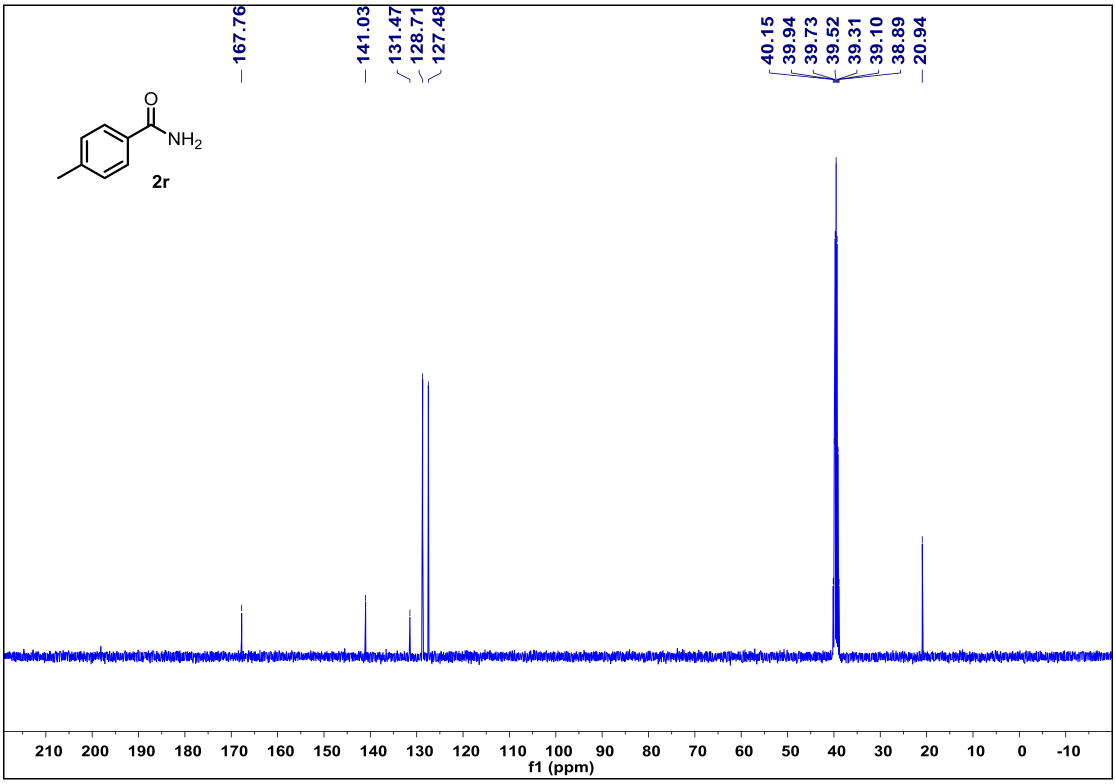

Figure S46. $^{13}C\{^{1}H\}$ NMR spectrum of **2r** (100 MHz, DMSO-d6, 298 K).

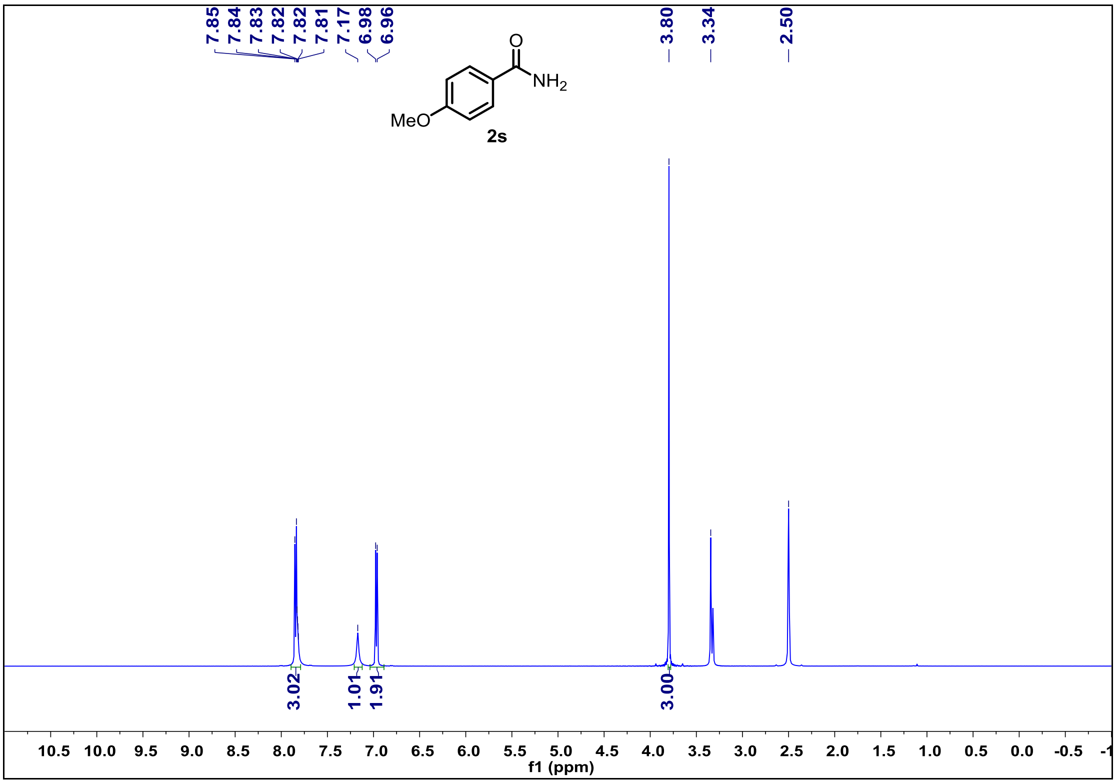

Figure S47. ^{1}H NMR spectrum of **2s** (500 MHz, DMSO-d6, 298 K).

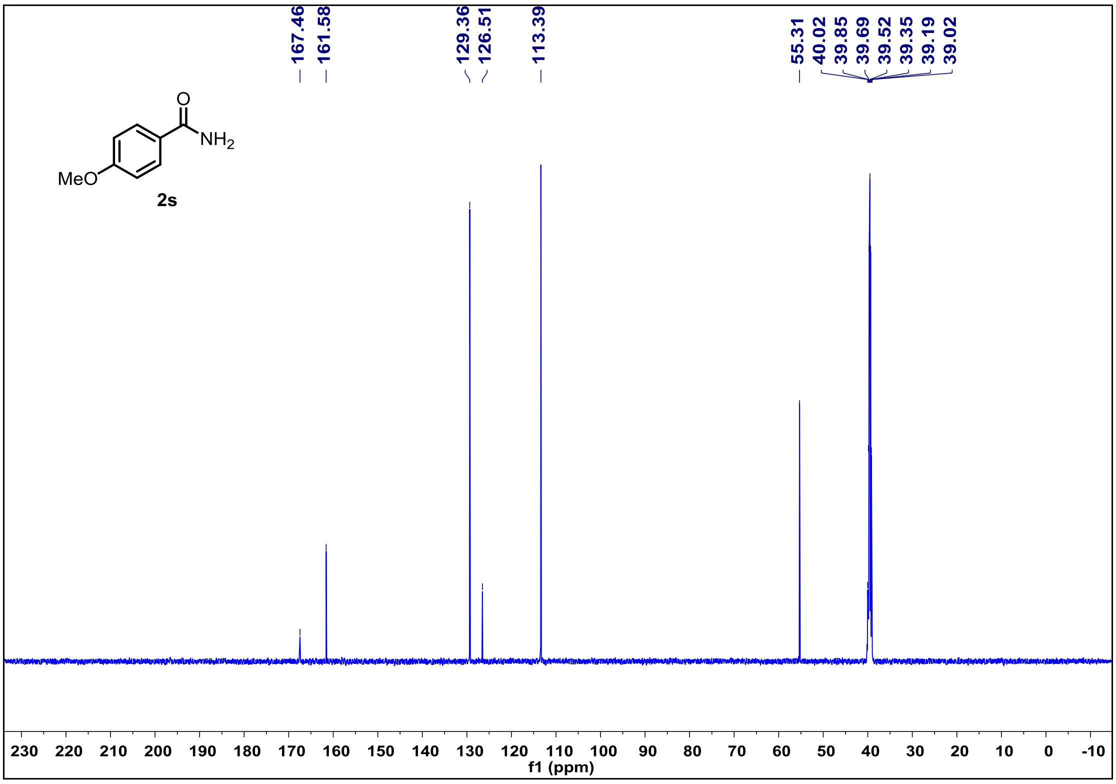

Figure S48. $^{13}C\{^{1}H\}$ NMR spectrum of **2s** (125 MHz, DMSO-d6, 298 K).

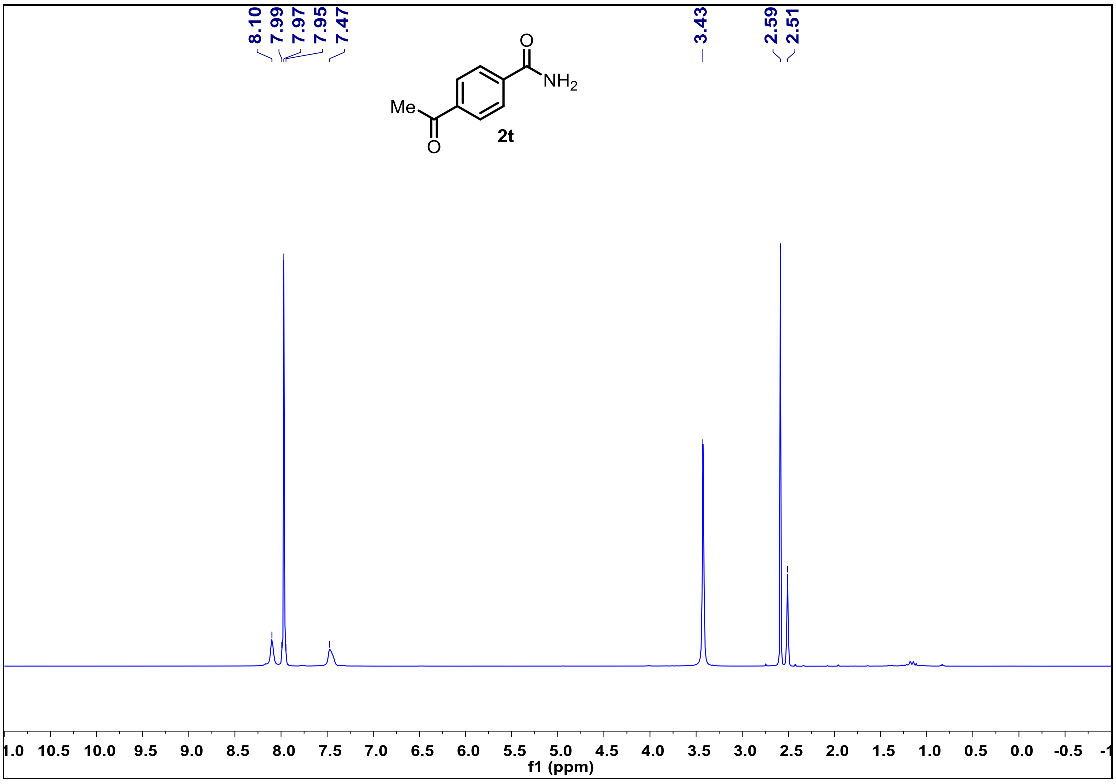

Figure S49. ^{1}H NMR spectrum of **2t** (400 MHz, DMSO-d6, 298 K).

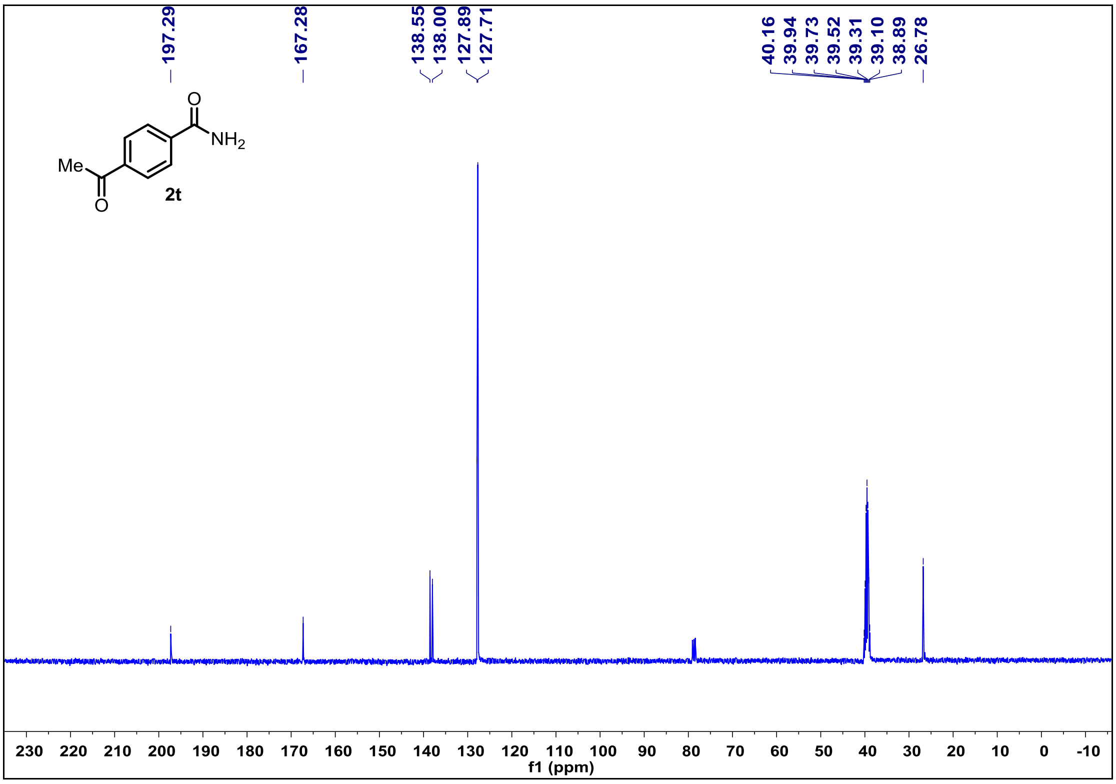

Figure S50. $^{13}C\{^{1}H\}$ NMR spectrum of **2t** (100 MHz, DMSO-d6, 298 K).

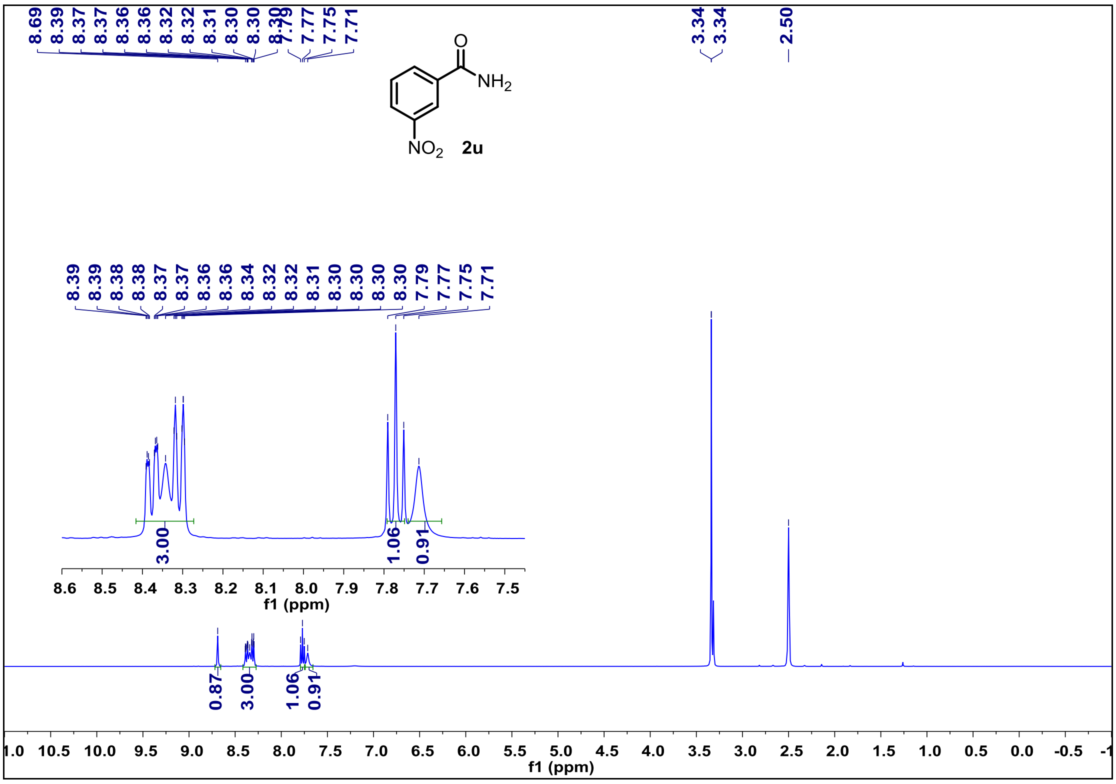

Figure S51. ^{1}H NMR spectrum of **2u** (400 MHz, DMSO-d6, 298 K).

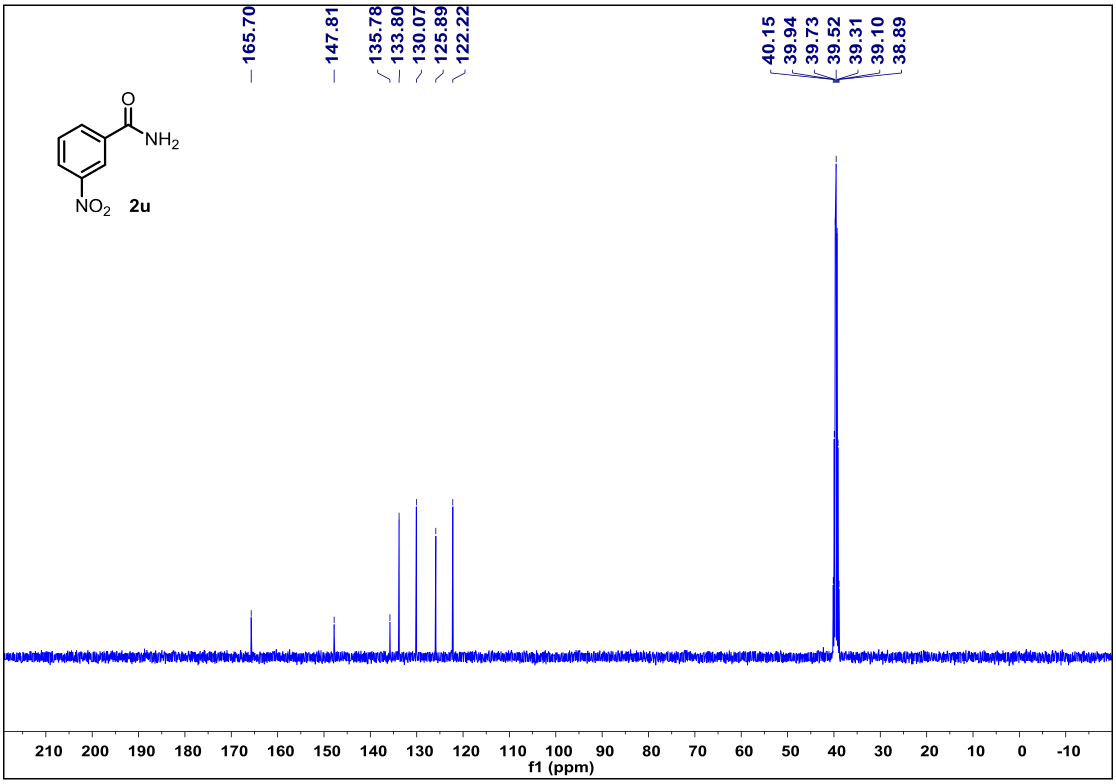

Figure S52. $^{13}C\{^{1}H\}$ NMR spectrum of **2u** (100 MHz, DMSO-d6, 298 K).

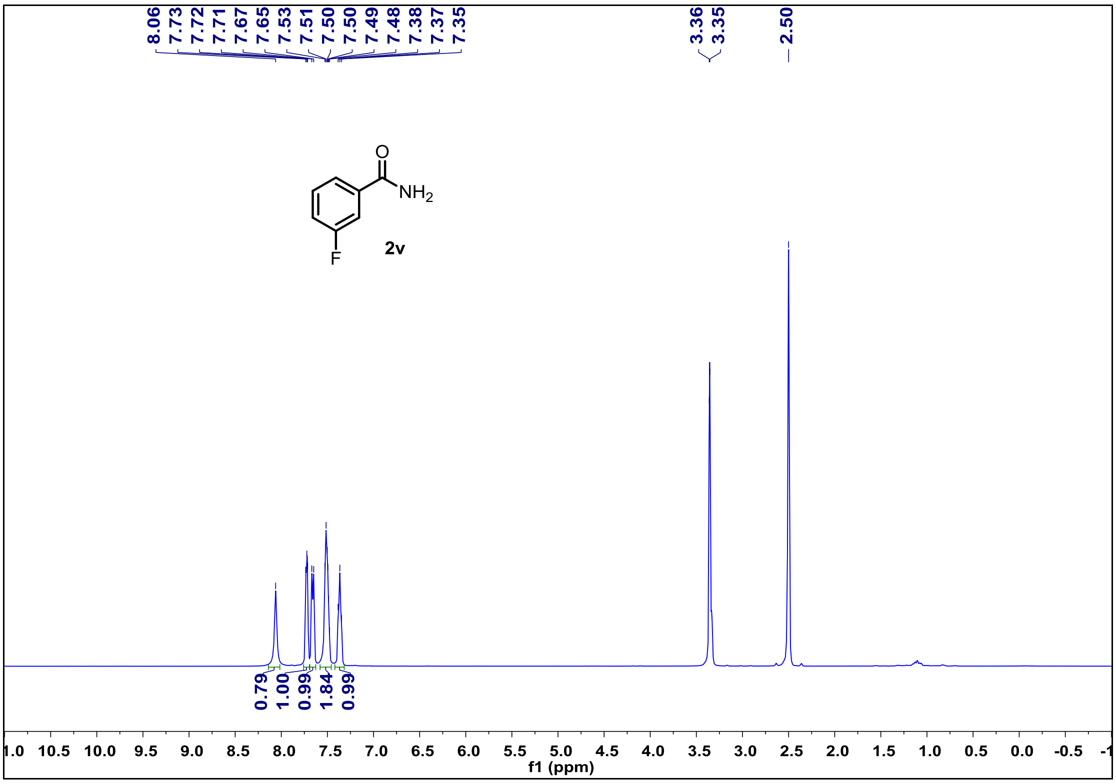

Figure S53. ^{1}H NMR spectrum of **2v** (500 MHz, DMSO-d6, 298 K).

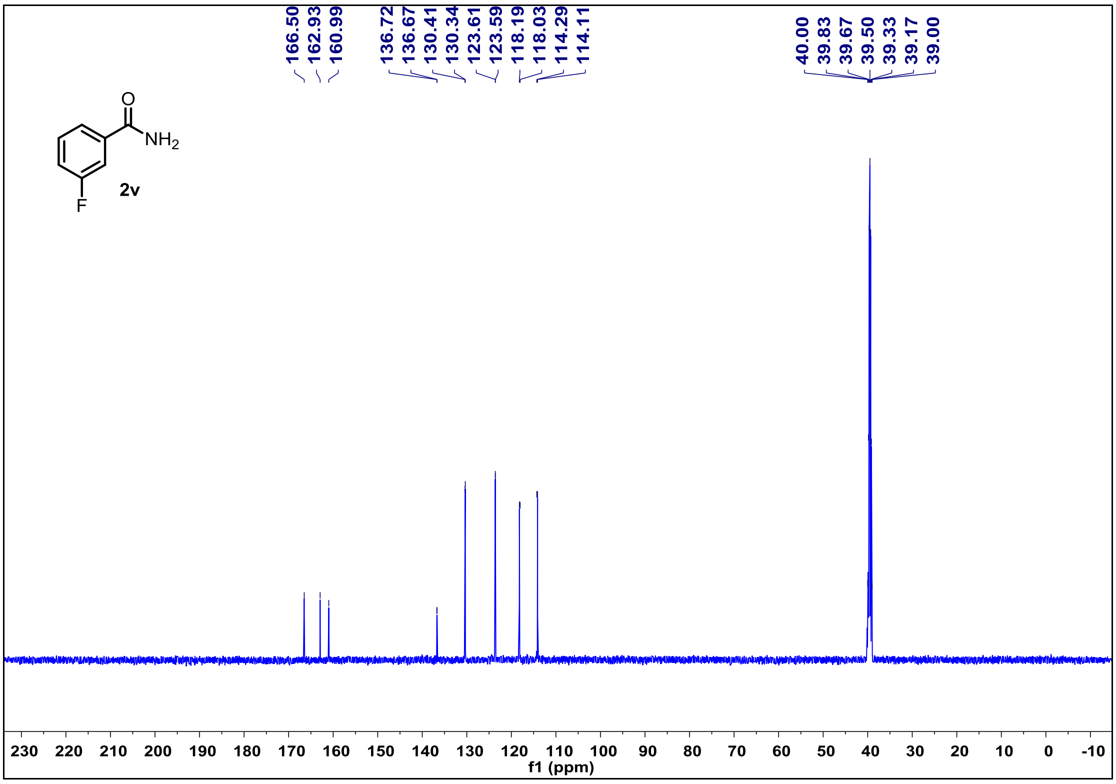

Figure S54. ^{13}C{^{1}H} NMR spectrum of **2v** (125 MHz, DMSO-d6, 298 K).

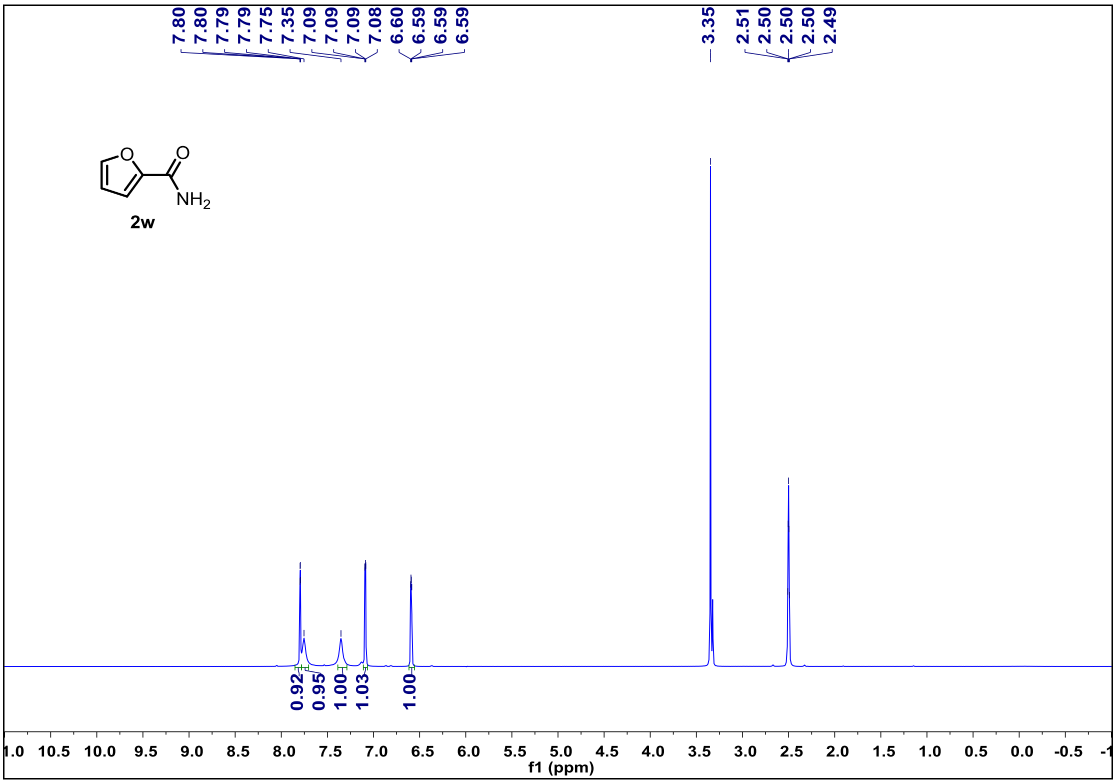

Figure S55. ^{1}H NMR spectrum of **2w** (400 MHz, DMSO-d6, 298 K).

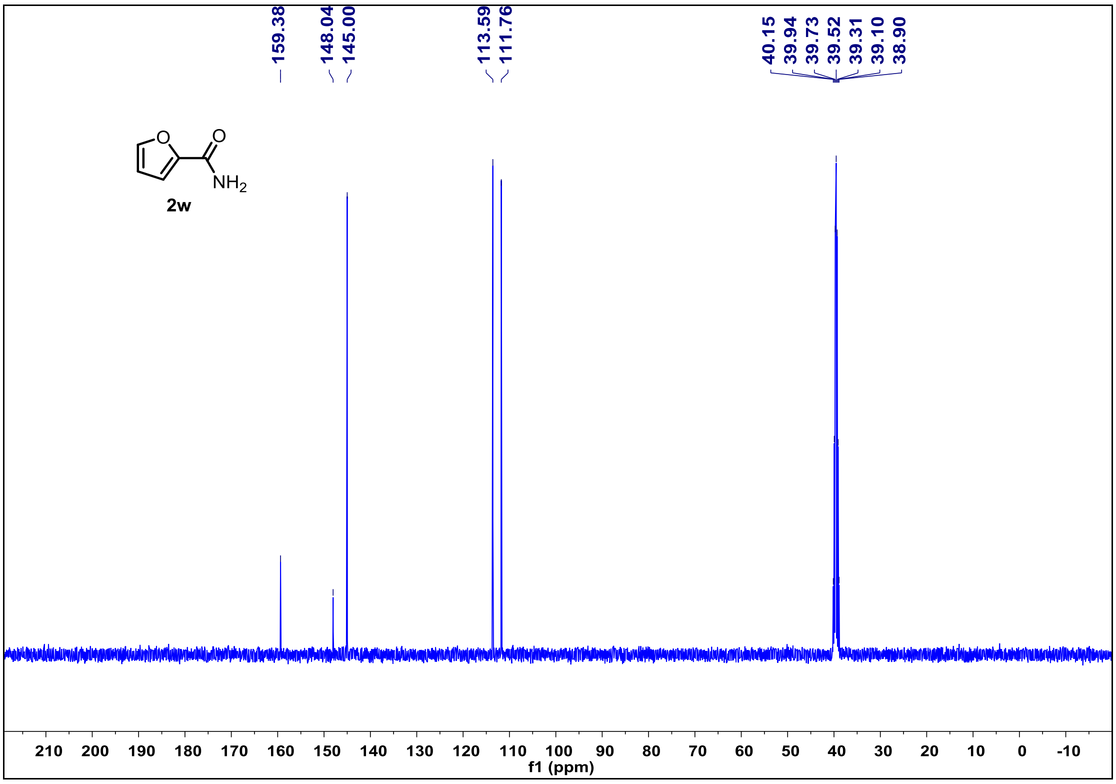

Figure S56. $^{13}C\{^{1}H\}$ NMR spectrum of **2w** (100 MHz, DMSO-d6, 298 K).

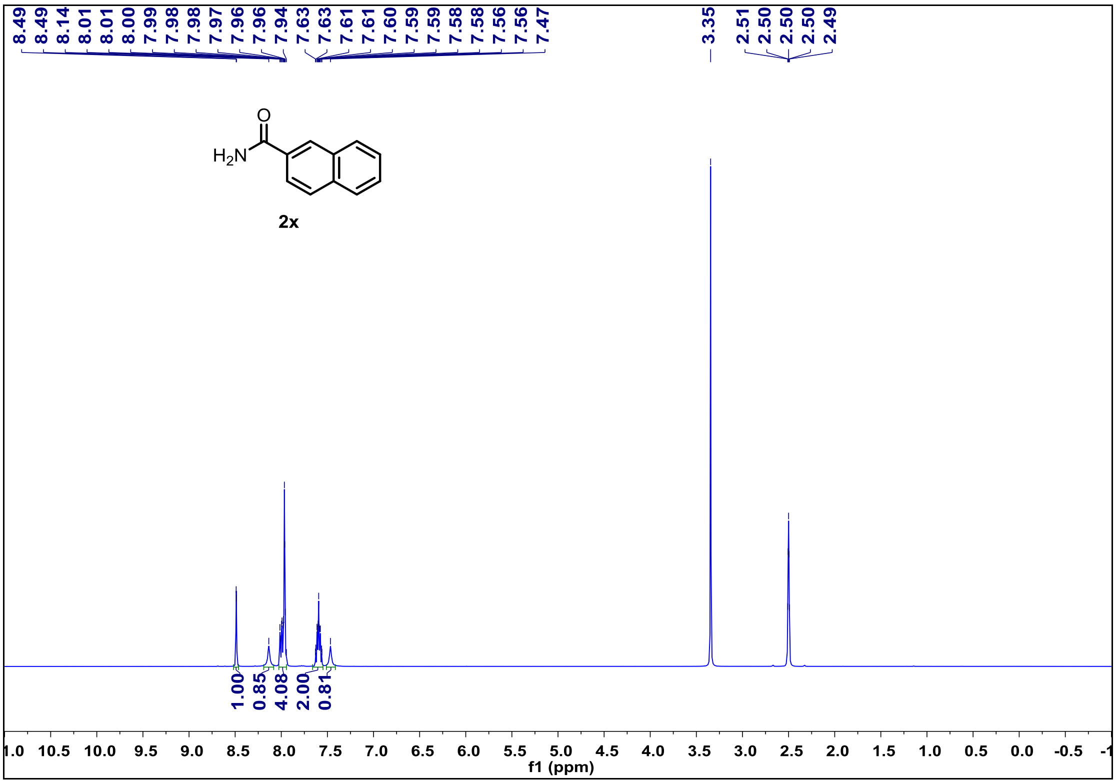

Figure S57. ^{1}H NMR spectrum of **2x** (400 MHz, DMSO-d6, 298 K).

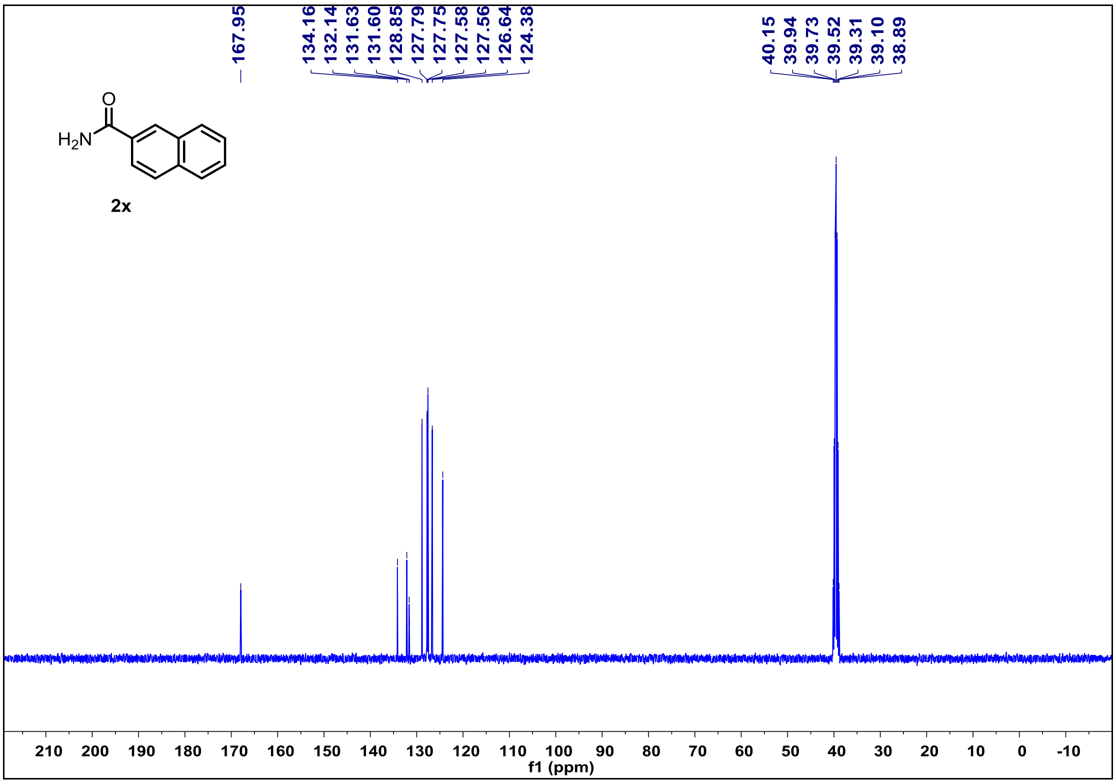

Figure S58. ^{13}C{^{1}H} NMR spectrum of **2x** (100 MHz, DMSO-d6, 298 K).

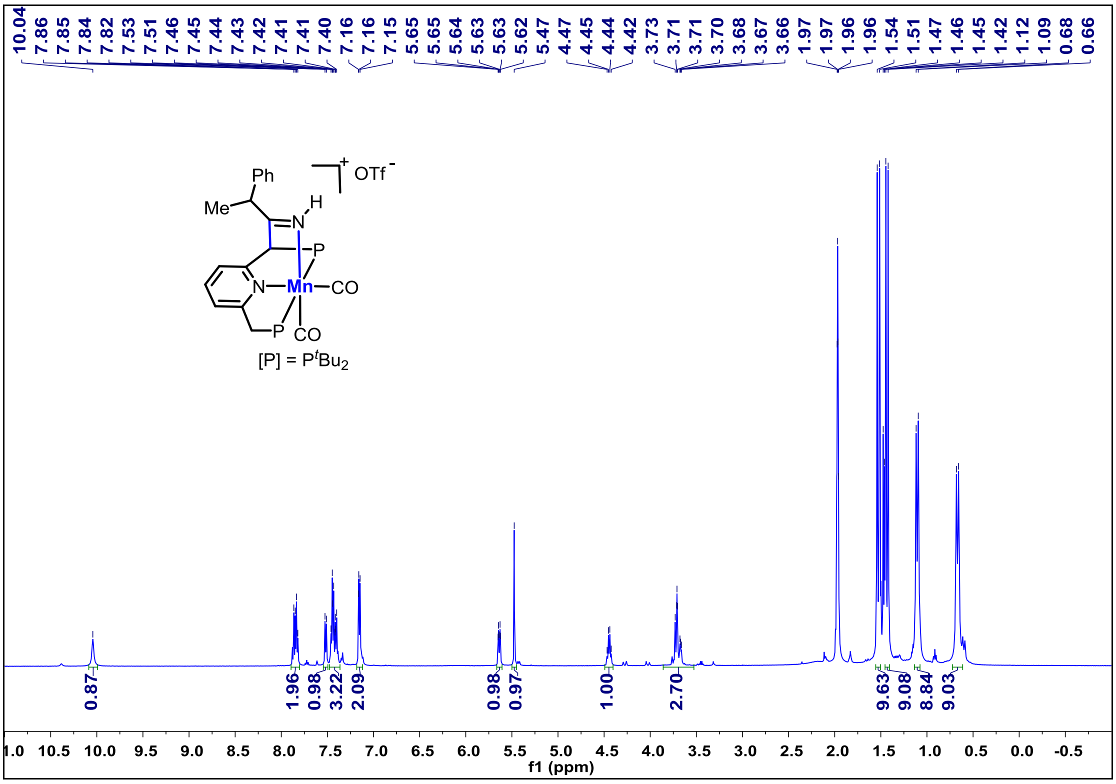

Figure S59. ^{1}H NMR spectrum of **Mn-7** (500 MHz, CD_3CN, 298 K).

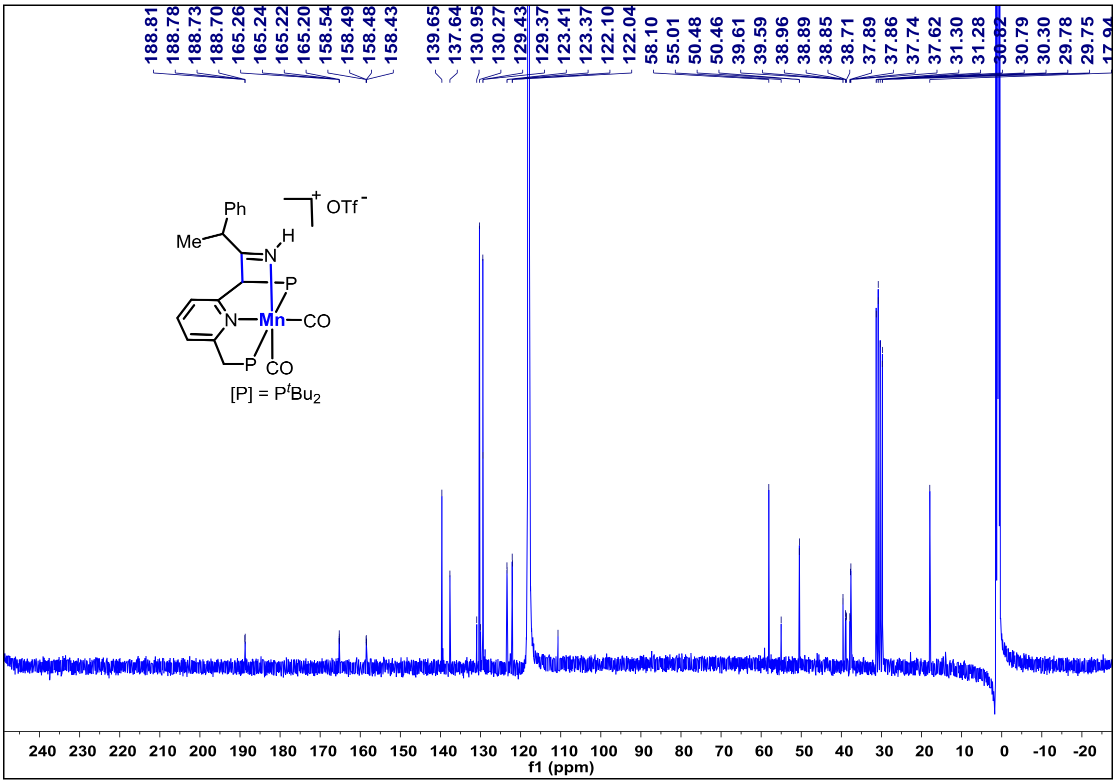

Figure S60. ^{1}H NMR spectrum of α- deuterated nitrile **Mn-7** (500 MHz, CD_3CN, 298 K).

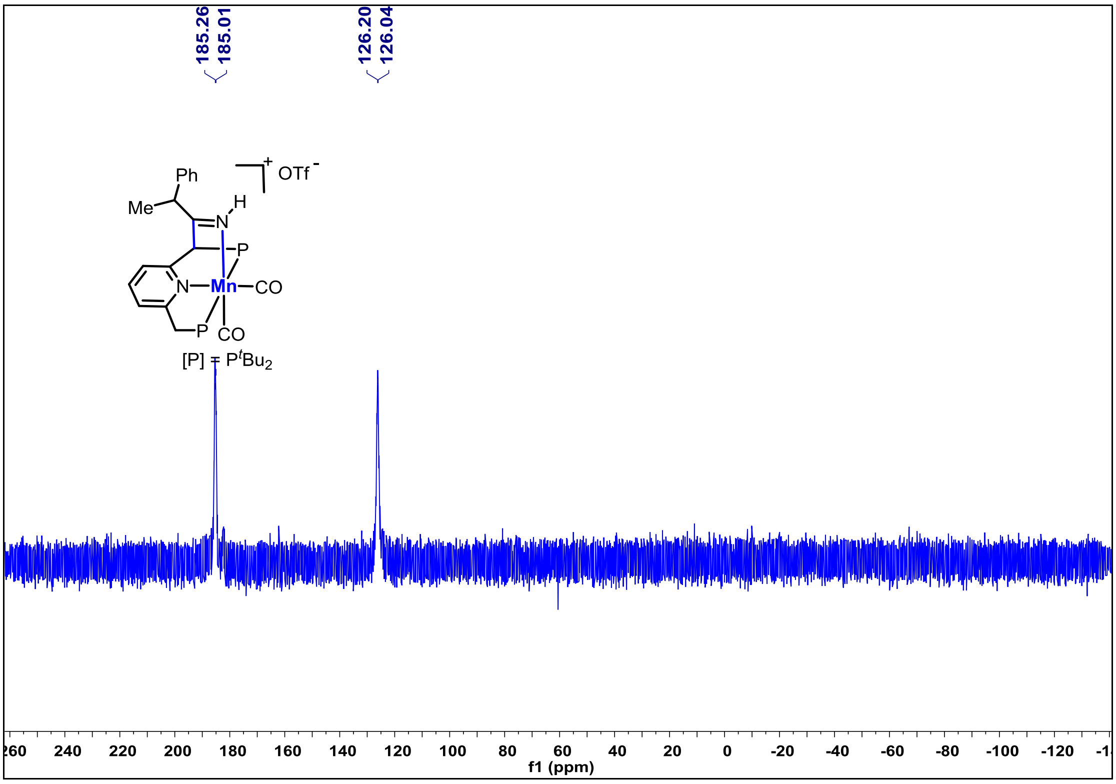

Figure S61. ^{31}P NMR spectrum of **Mn-7** (202 MHz, CD_3CN, 298 K).

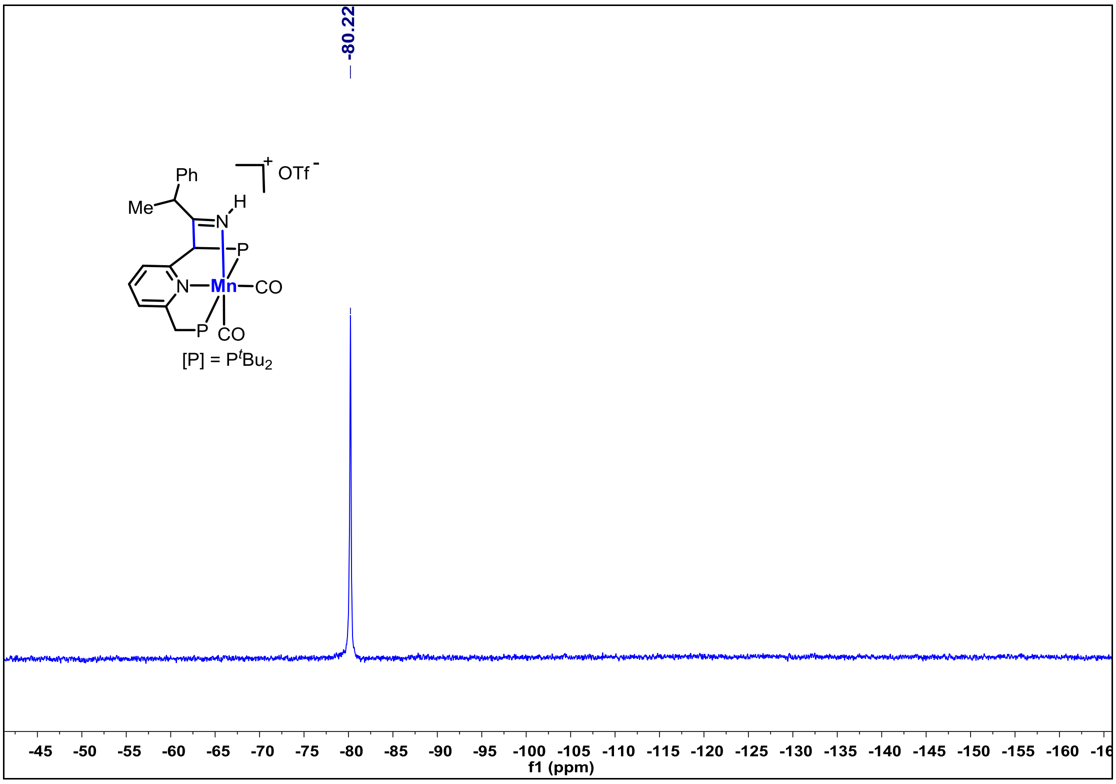

Figure S62. ^{19}F NMR spectrum of **Mn-7** (470 MHz, CD_3CN, 298 K).

7.2 ^{1}H NMR, ^{2}H NMR and ^{13}C NMR Spectra of the nitriles and α- deuterated nitriles

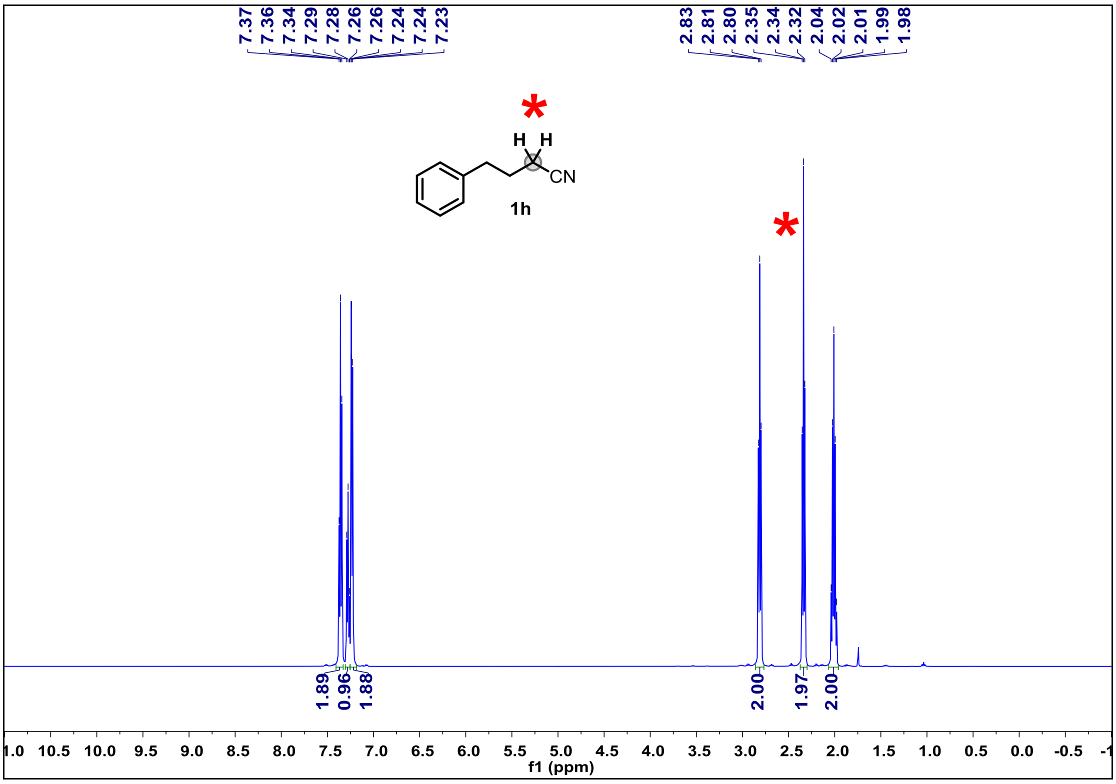

Figure S63. ^{1}H NMR spectrum of nitrile **1h** (500 MHz, $CDCl_3$, 298 K, red asterisk, α-CH_2 of nitrile).

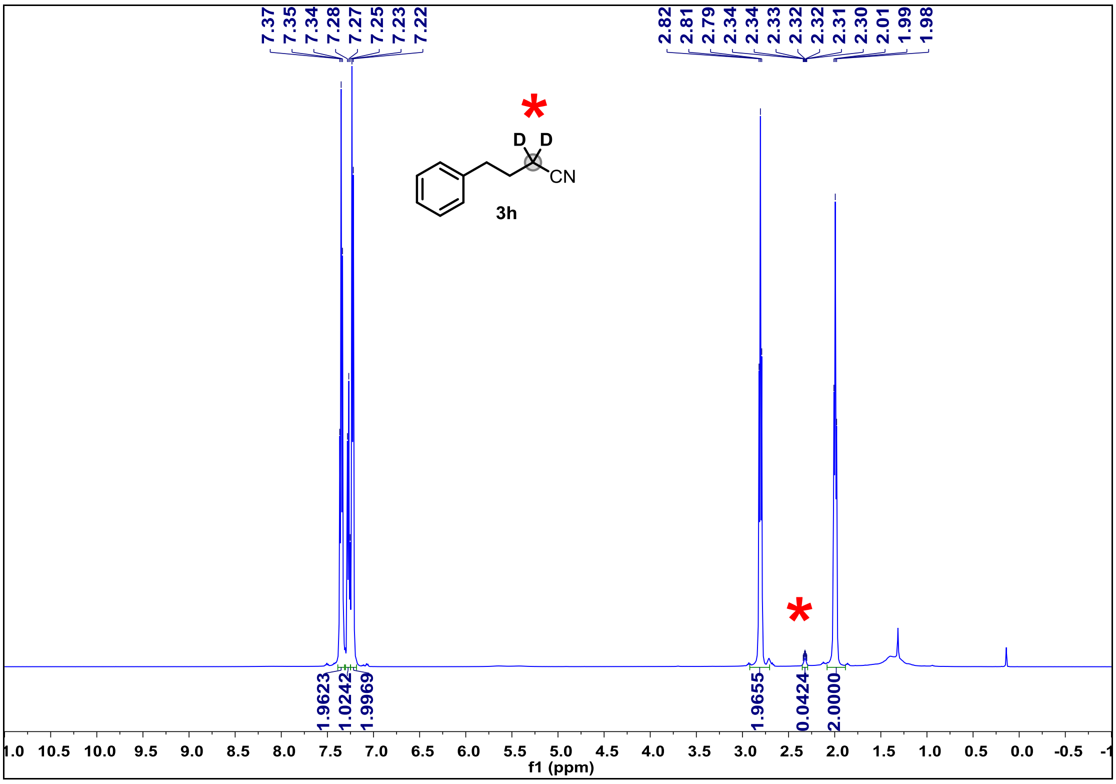

Figure S64. ^{1}H NMR spectrum of α- deuterated nitrile **3h** (500 MHz, $CDCl_3$, 298 K).

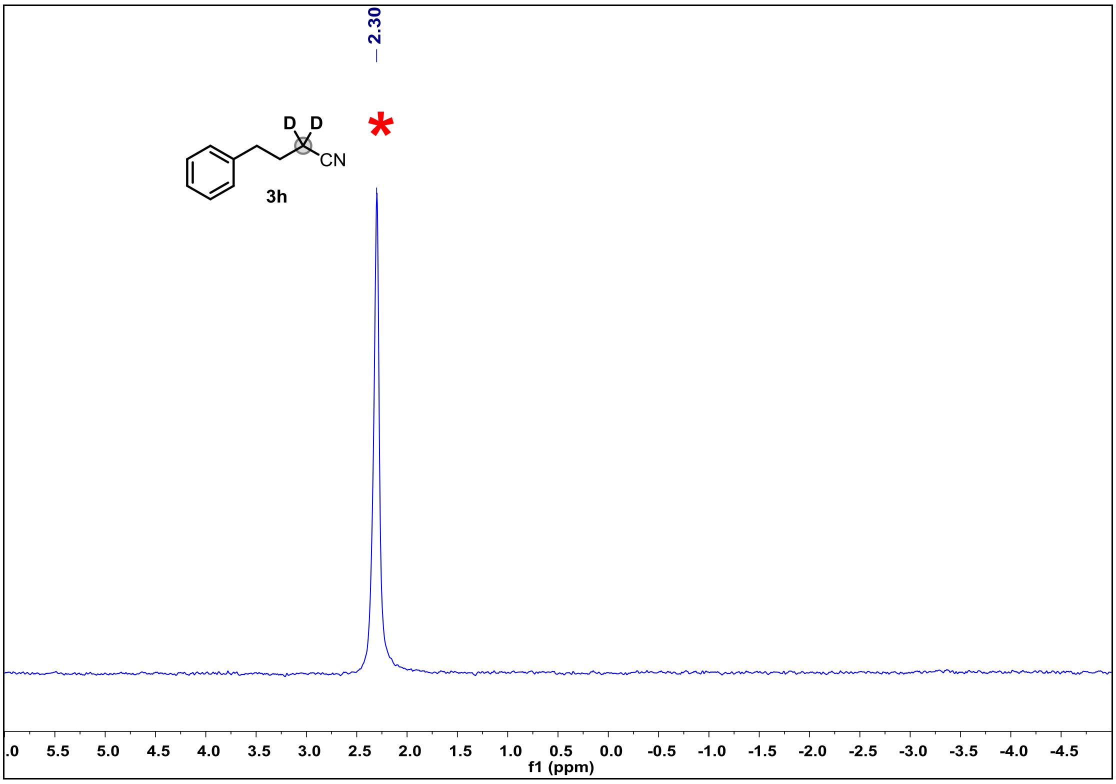

Figure S65. ^{2}H NMR spectrum of α- deuterated nitrile **3h** (77 MHz, $CDCl_3$, 298 K).

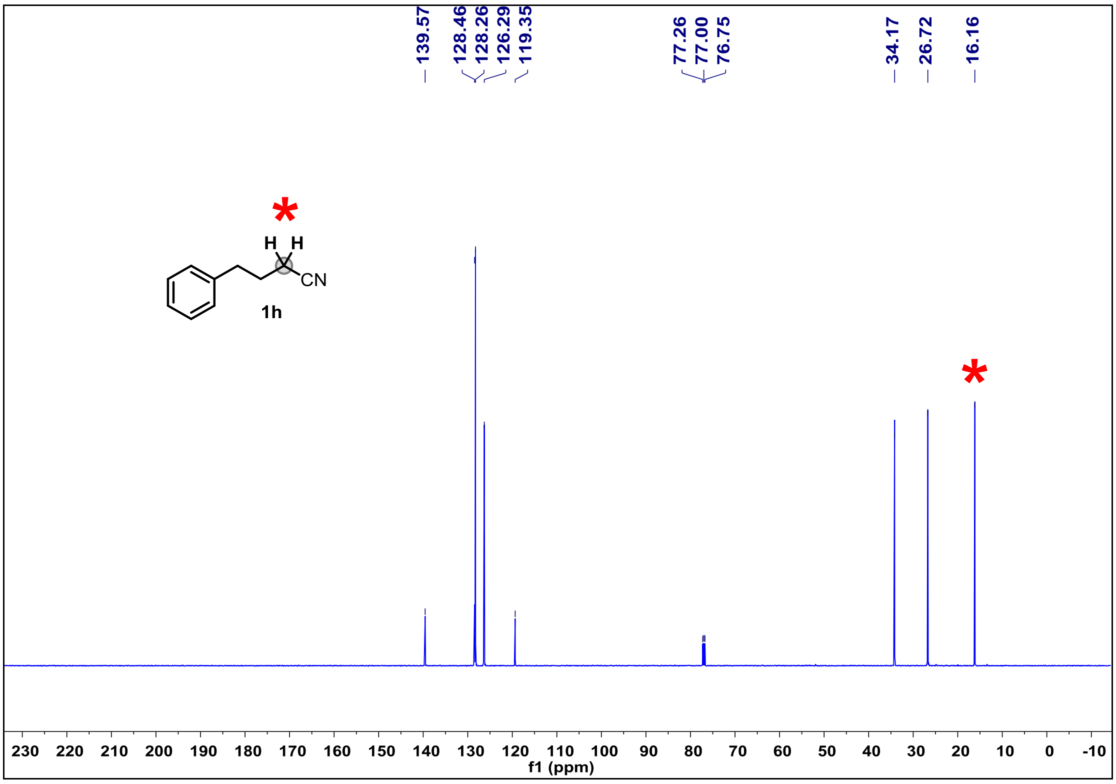

Figure S66. $^{13}C\{^{1}H\}$ NMR spectrum of nitrile **1h** (125 MHz, $CDCl_3$, 298 K).

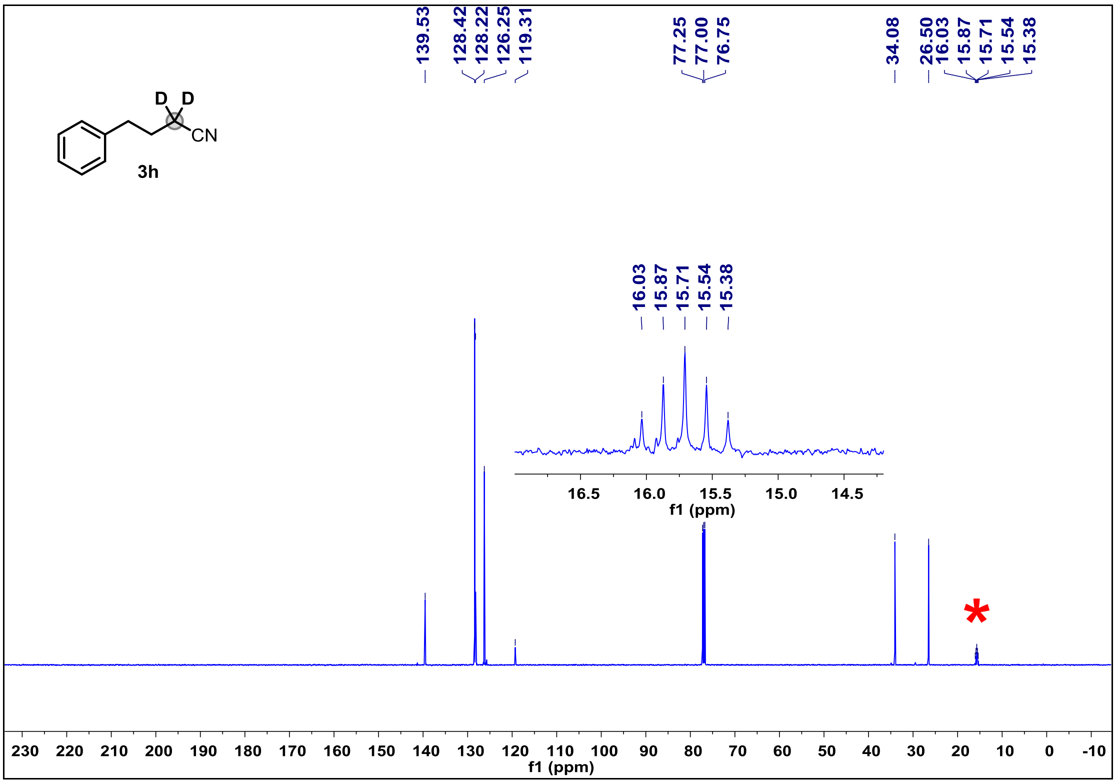

Figure S67. $^{13}C\{^1H\}$ NMR spectrum of α- deuterated nitrile **3h** (125 MHz, $CDCl_3$, 298 K).

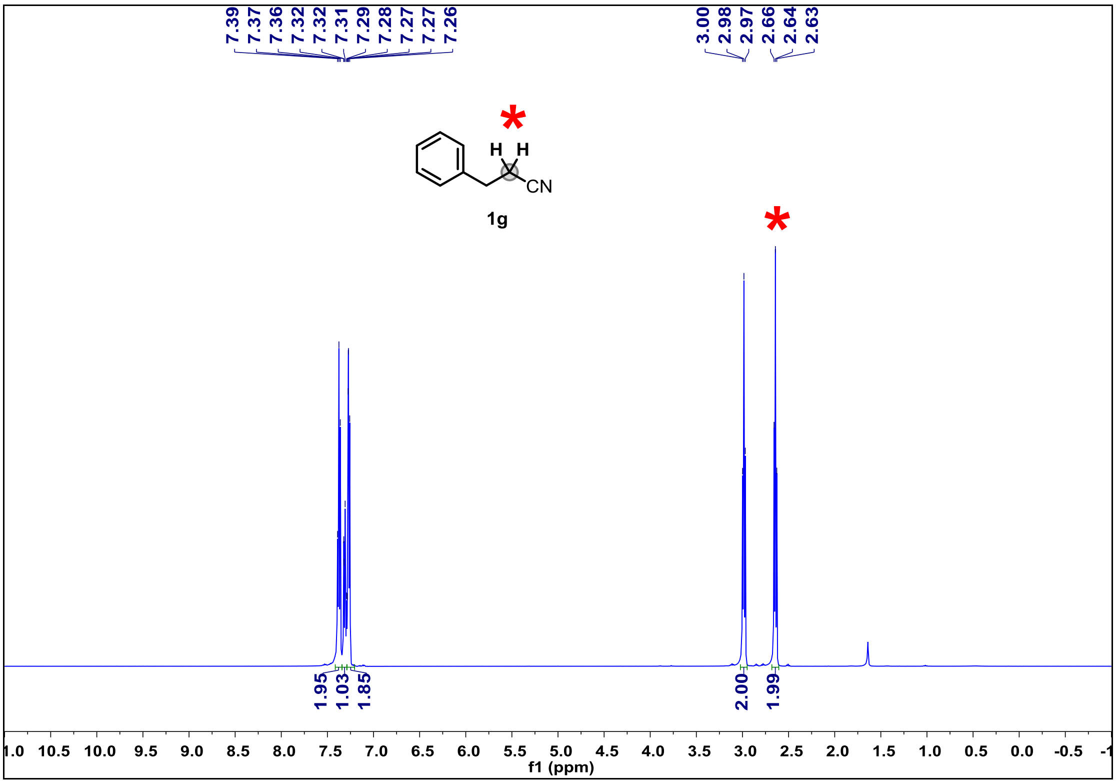

Figure S68. ^1H NMR spectrum of nitrile **1g** (500 MHz, $CDCl_3$, 298 K).

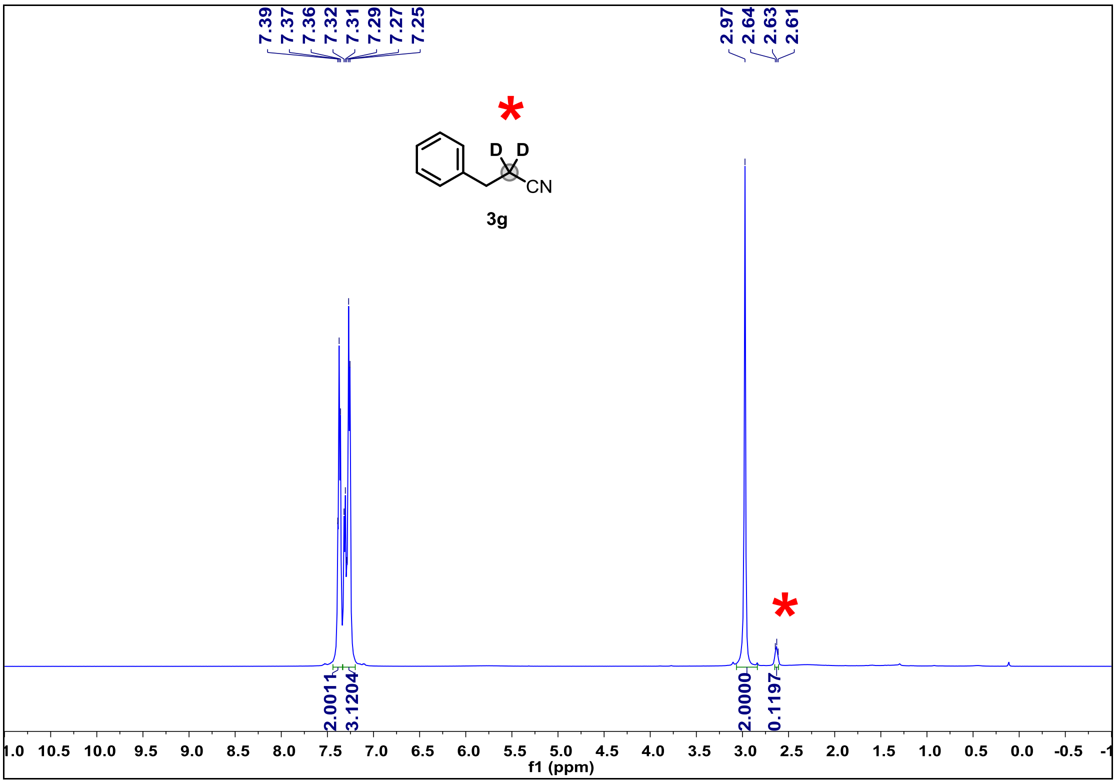

Figure S69. ^{1}H NMR spectrum of α- deuterated nitrile **3g** (125 MHz, $CDCl_3$, 298 K).

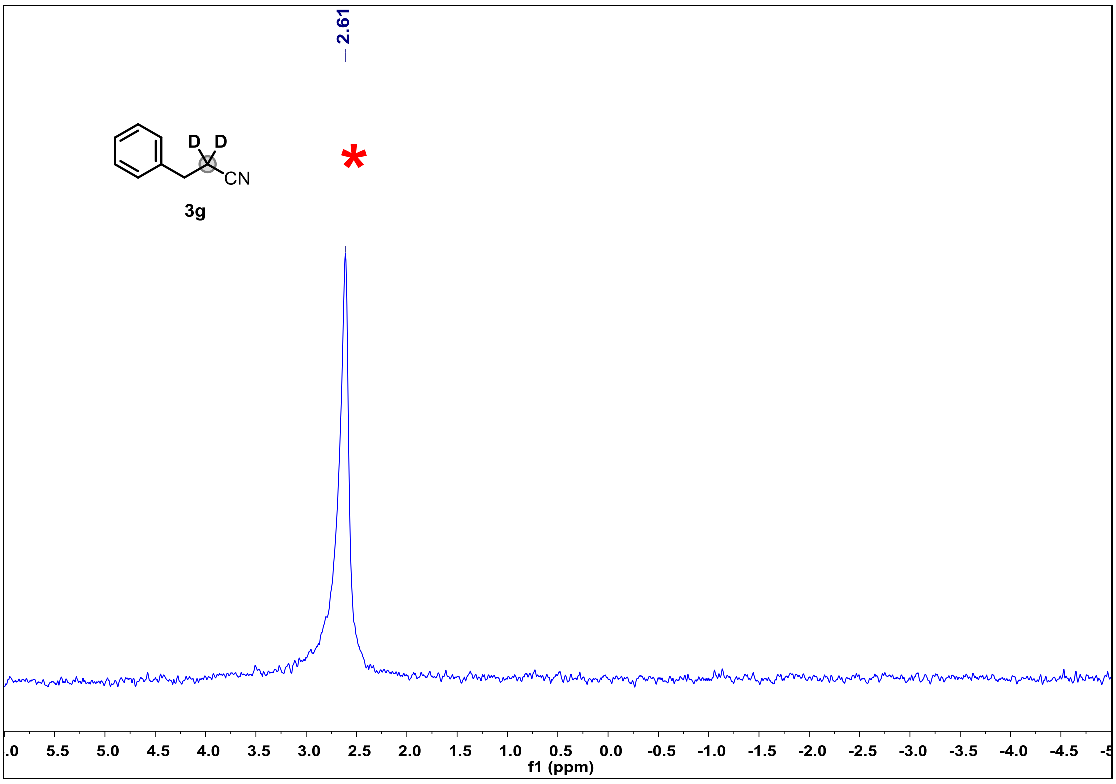

Figure S70. ^{2}H NMR spectrum of α- deuterated nitrile **3g** (77 MHz, $CDCl_3$, 298 K).

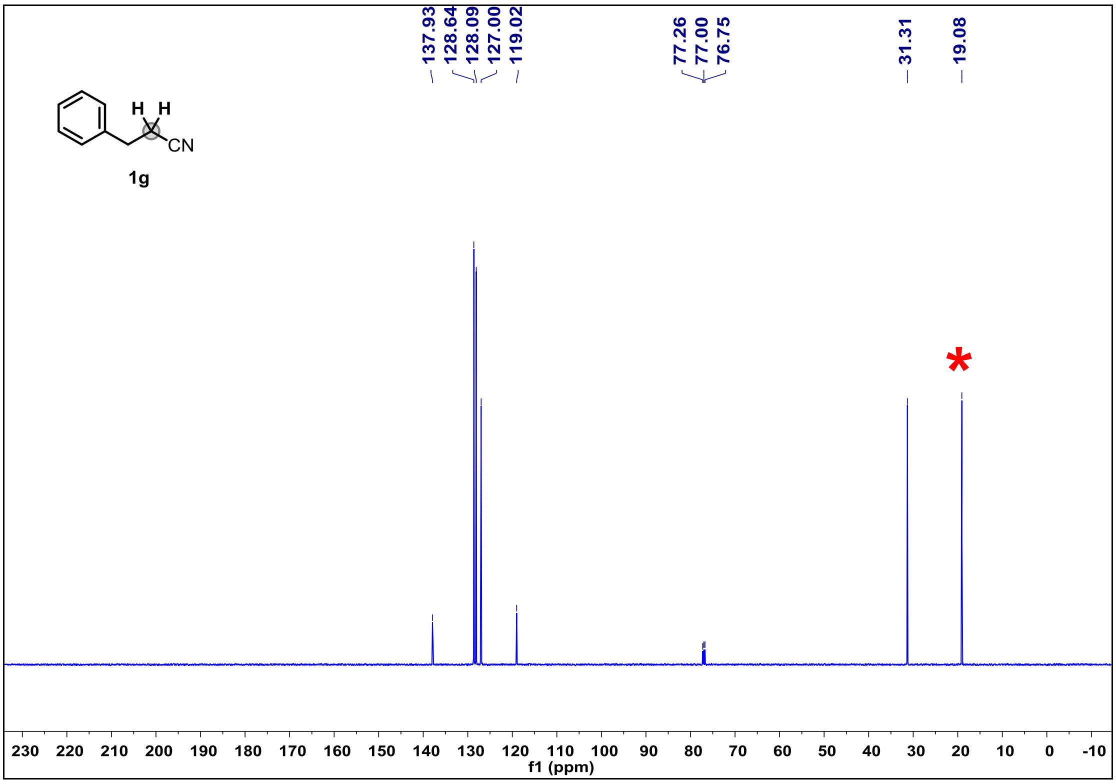

Figure S71. $^{13}C\{^1H\}$ NMR spectrum of nitrile **1g** (125 MHz, $CDCl_3$, 298 K).

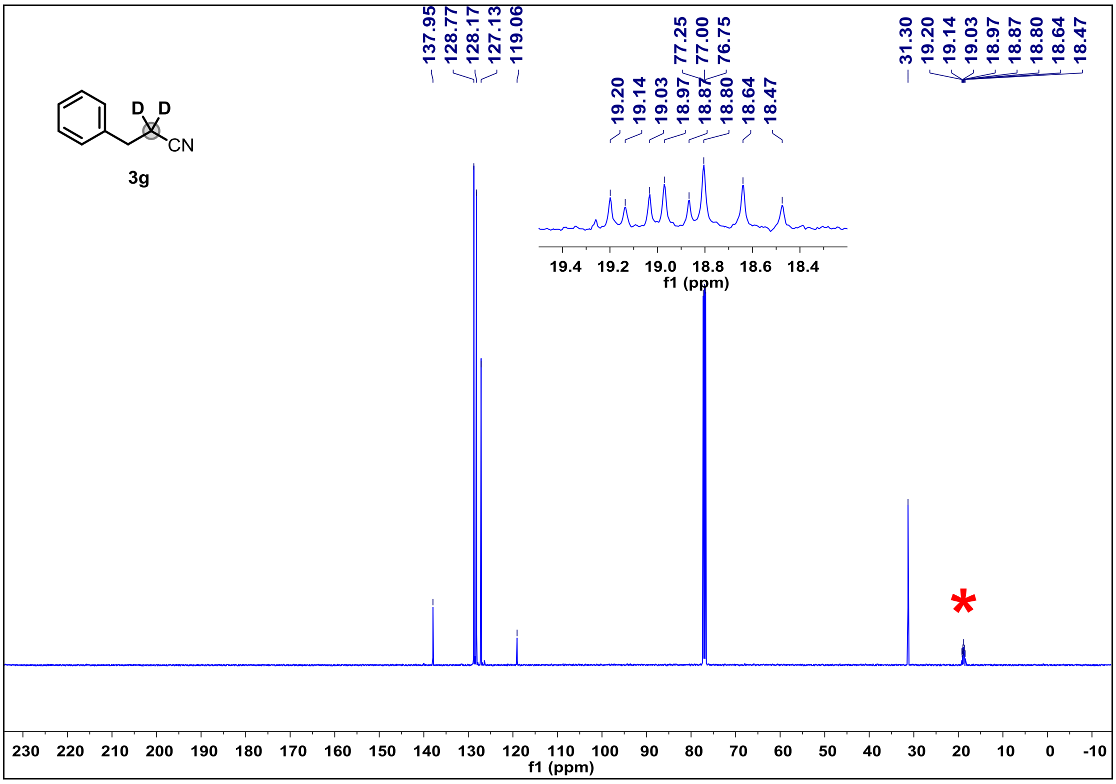

Figure S72. $^{13}C\{^1H\}$ NMR spectrum of α- deuterated nitrile **3g** (125 MHz, $CDCl_3$, 298 K).

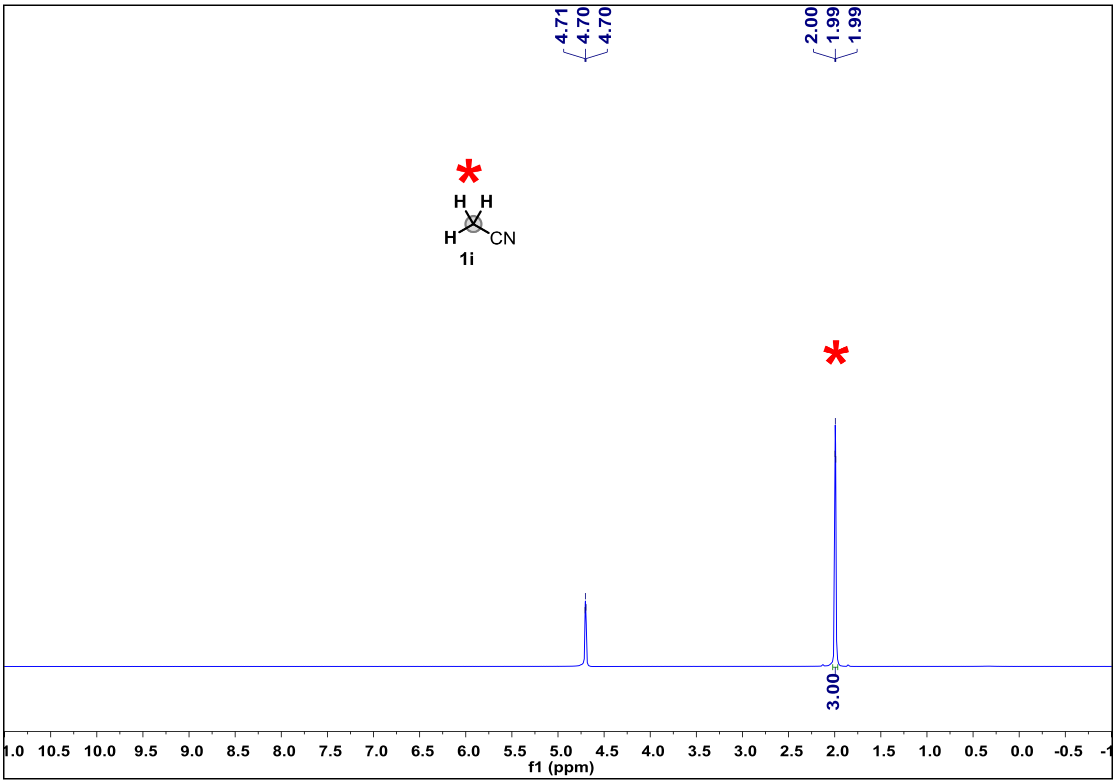

Figure S73. ^{1}H NMR spectrum of nitrile **1i** (500 MHz, $CDCl_3$, 298 K).

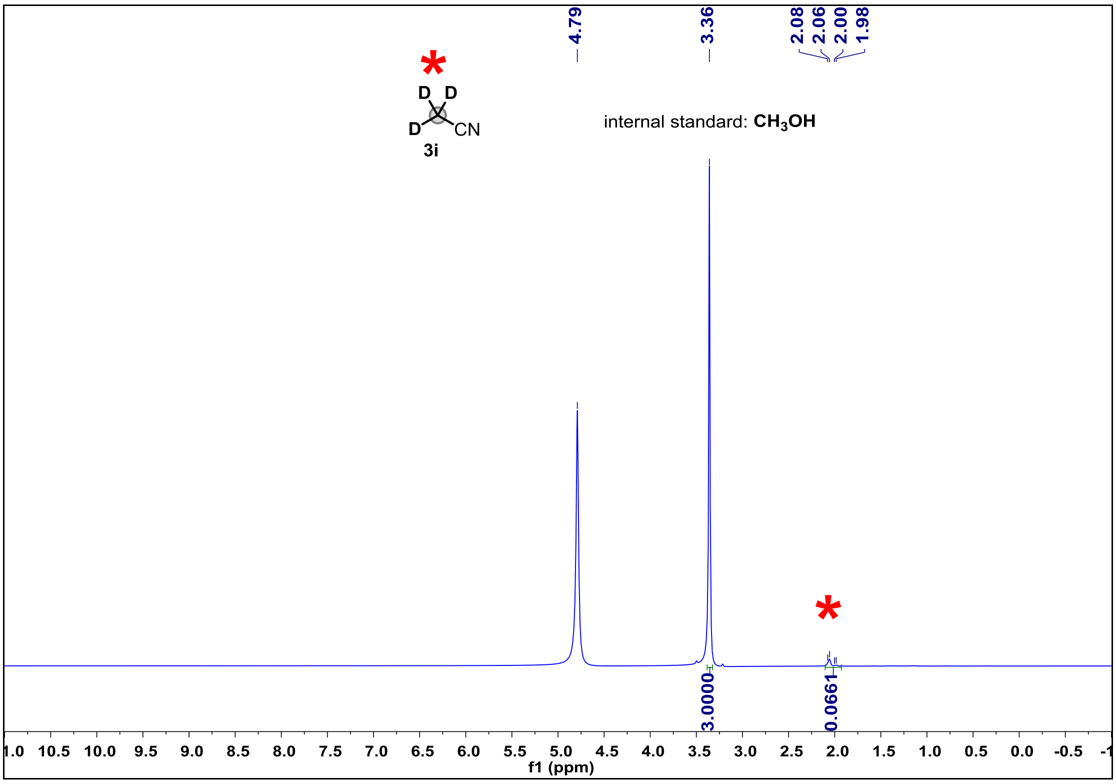

Figure S74. ^{1}H NMR spectrum of α- deuterated nitrile **3i** (125 MHz, $CDCl_3$, 298 K).

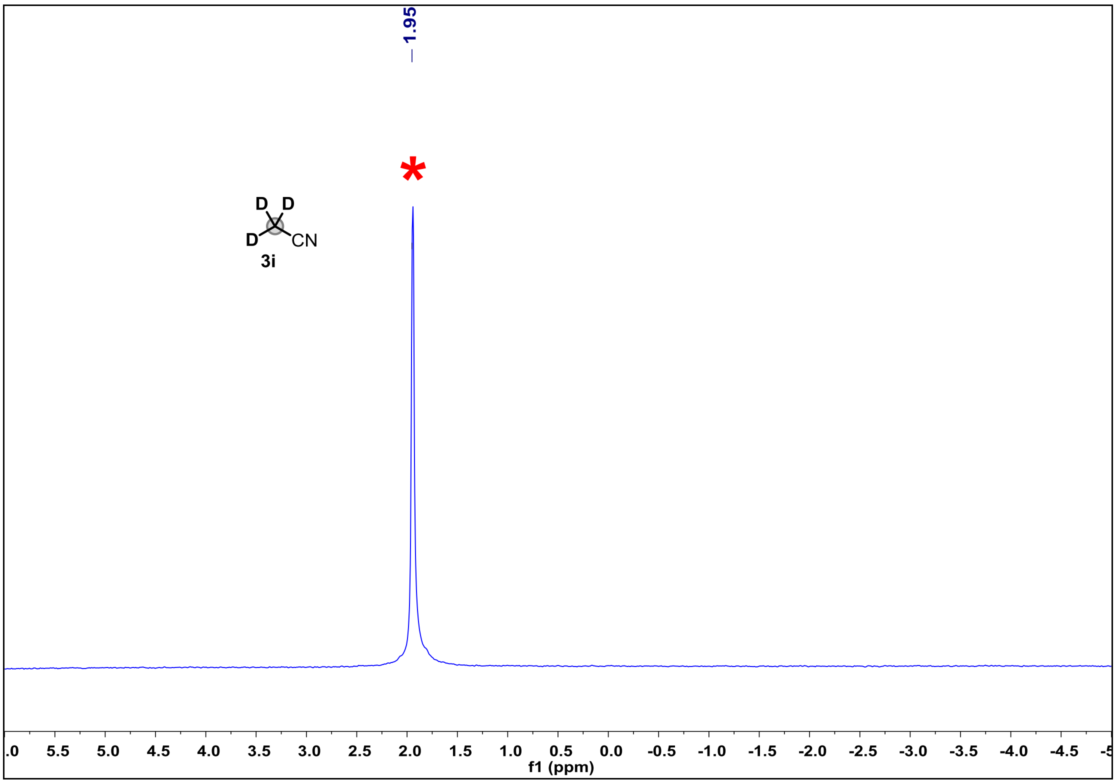

Figure S75. ^{2}H NMR spectrum of α- deuterated nitrile **3i** (77 MHz, $CDCl_3$, 298 K).

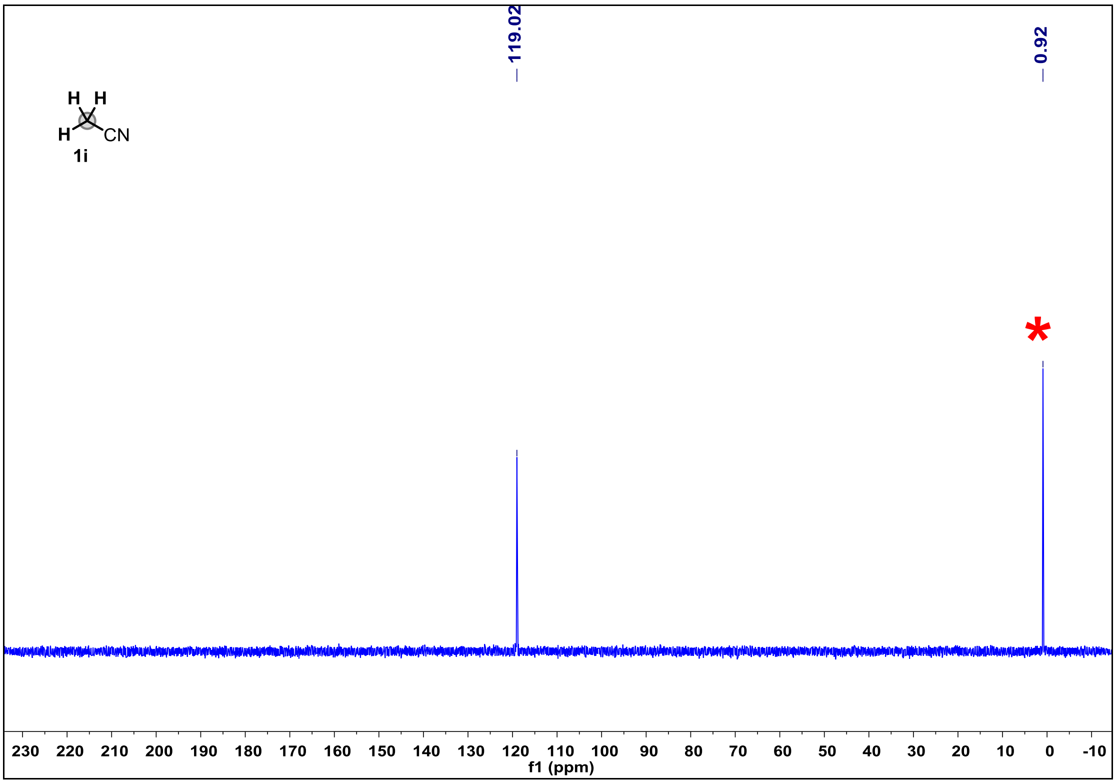

Figure S76. $^{13}C\{^{1}H\}$ NMR spectrum of nitrile **1i** (500 MHz, $CDCl_3$, 298 K).

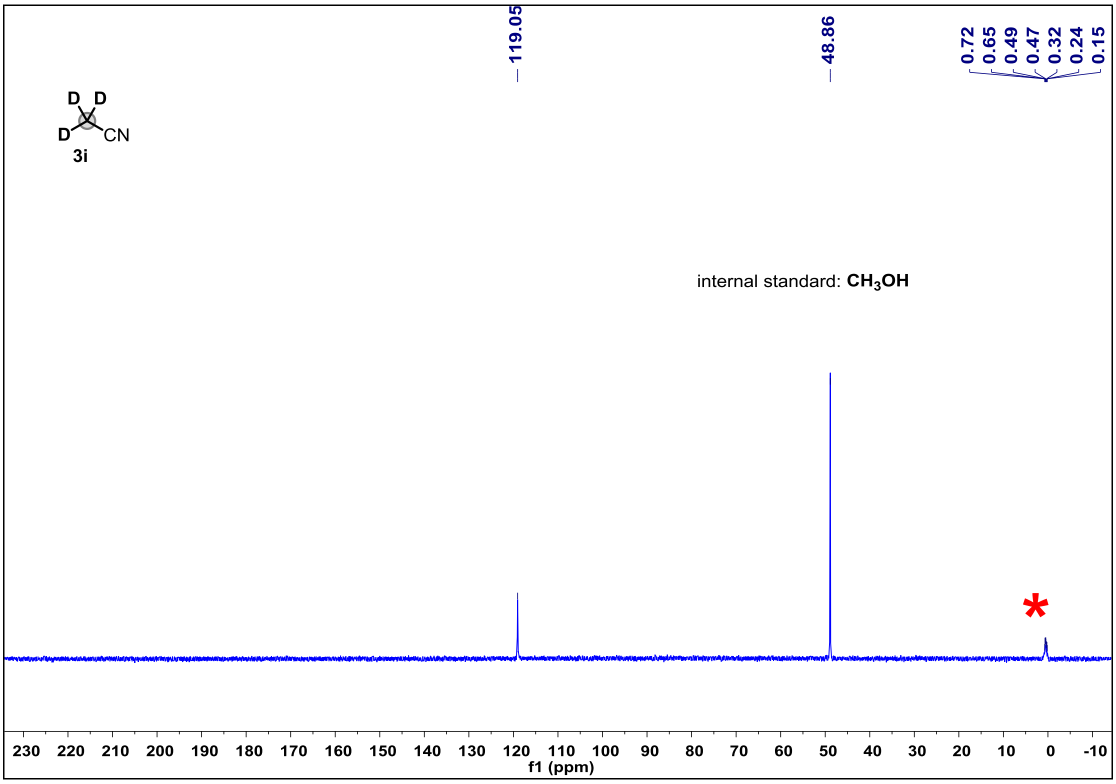

Figure S77. $^{13}C\{^{1}H\}$ NMR spectrum of α- deuterated nitrile **3i** (125 MHz, $CDCl_3$, 298 K).

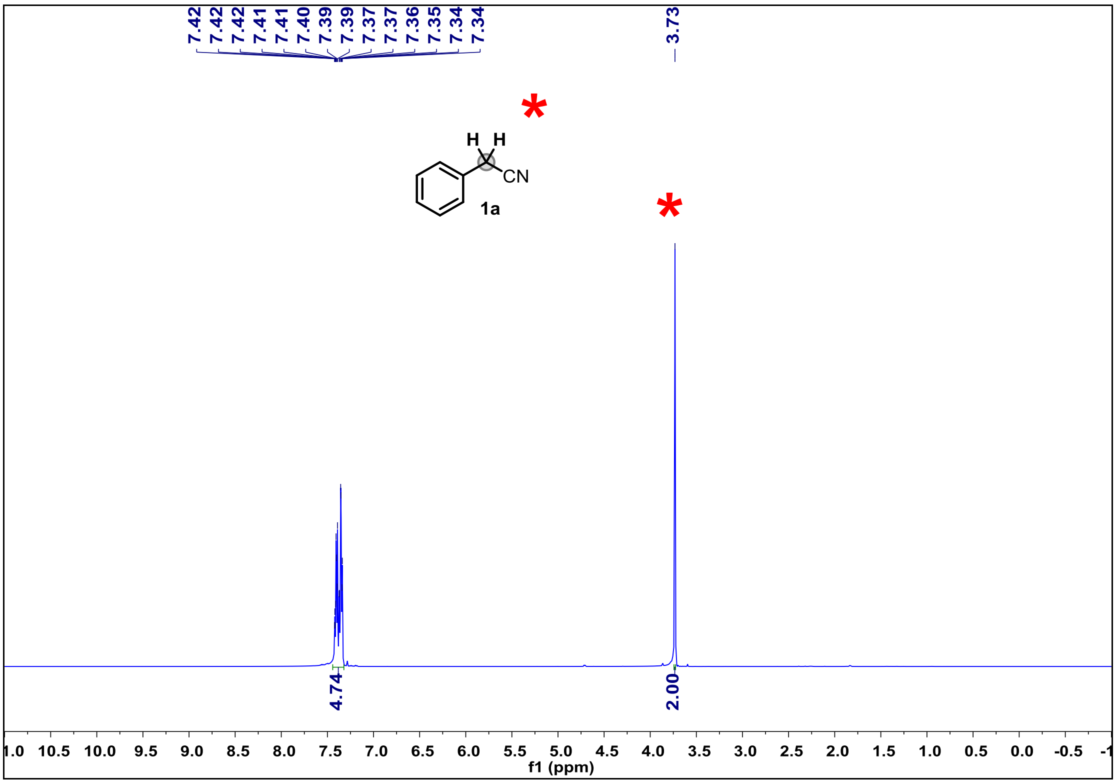

Figure S78. ^{1}H NMR spectrum of nitrile **1a** (500 MHz, $CDCl_3$, 298 K).

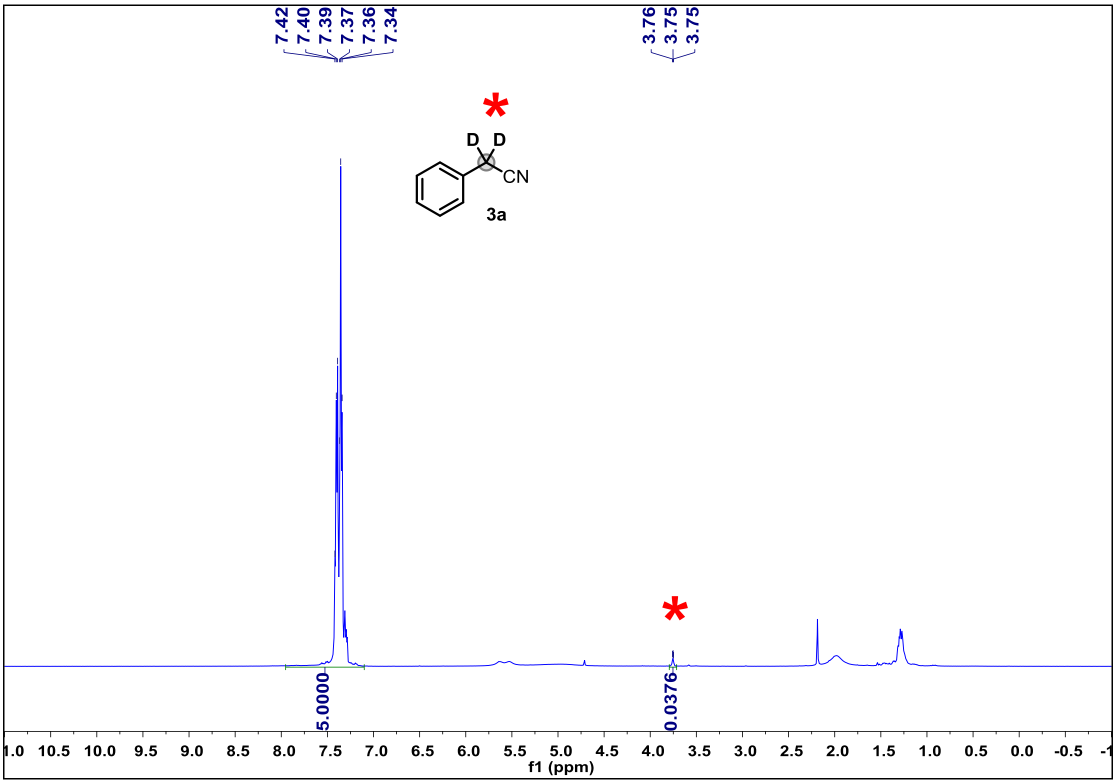

Figure S79. ¹H NMR spectrum of α- deuterated nitrile **3a** (125 MHz, $CDCl_3$, 298 K).

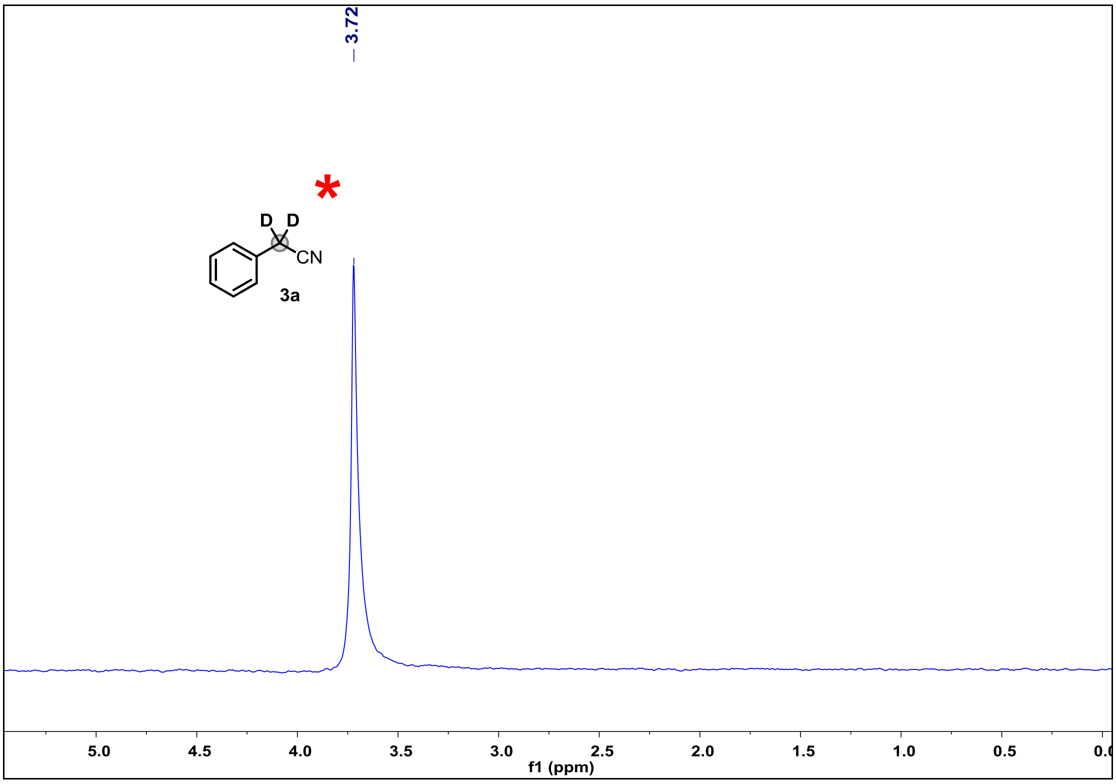

Figure S80. ²H NMR spectrum of α- deuterated nitrile **3a** (77 MHz, $CDCl_3$, 298 K).

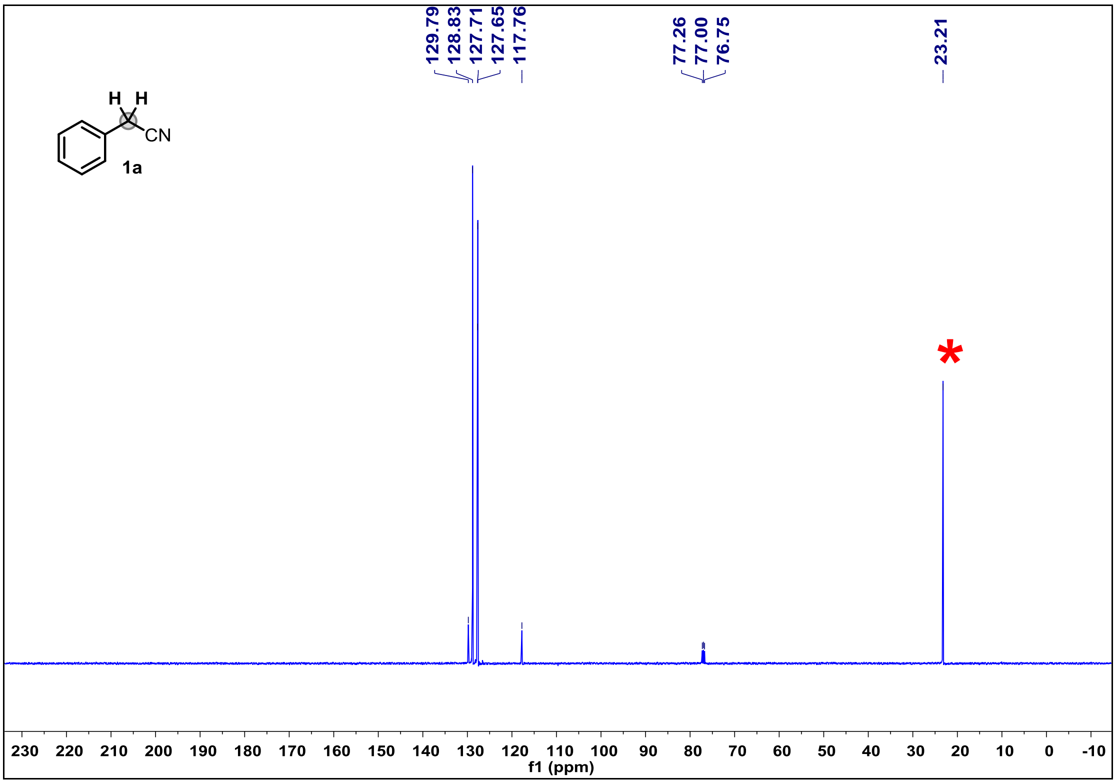

Figure S81. $^{13}C\{^{1}H\}$ NMR spectrum of nitrile **1a** (500 MHz, $CDCl_3$, 298 K).

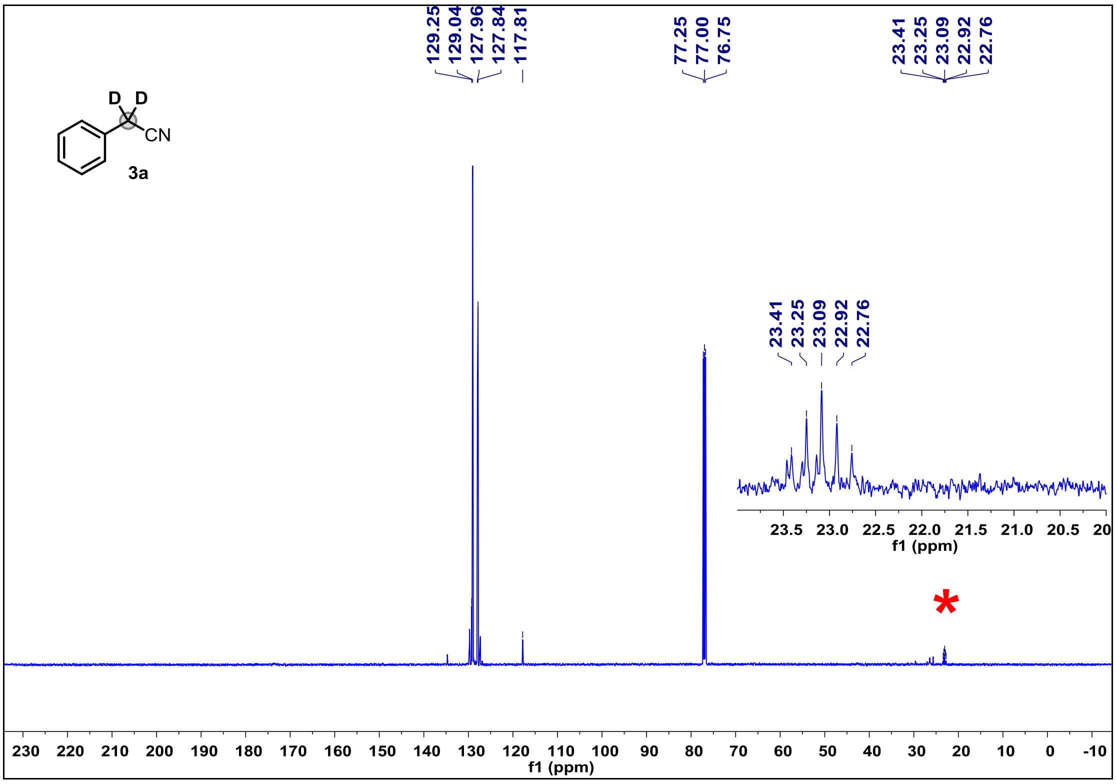

Figure S82. $^{13}C\{^{1}H\}$ NMR spectrum of α- deuterated nitrile **3a** (125 MHz, $CDCl_3$, 298 K).

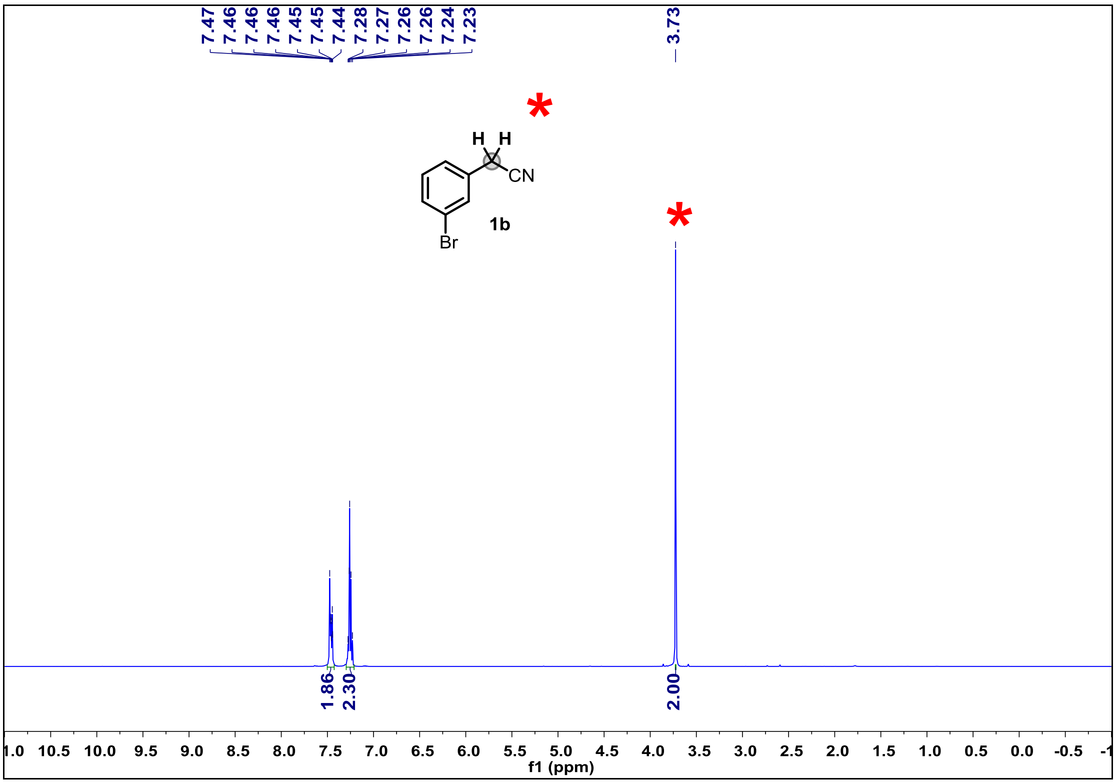

Figure S83. ^{1}H NMR spectrum of nitrile **1b** (500 MHz, $CDCl_3$, 298 K).

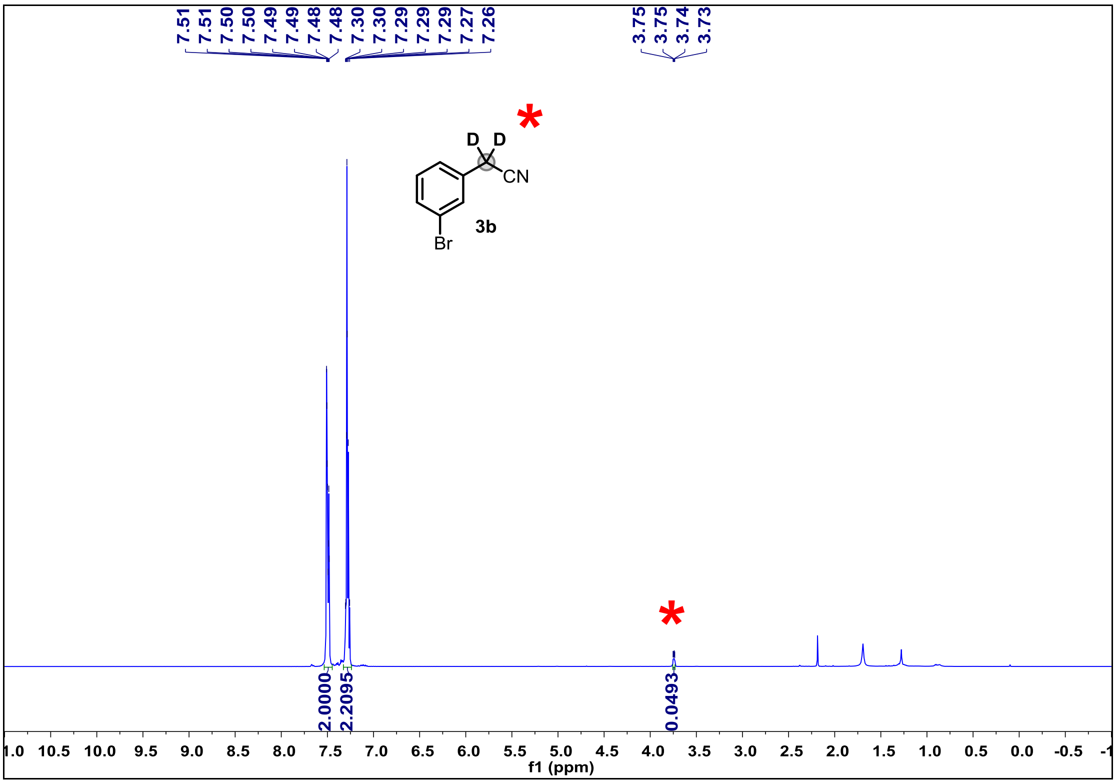

Figure S84. ^{1}H NMR spectrum of α- deuterated nitrile **3b** (125 MHz, $CDCl_3$, 298 K).

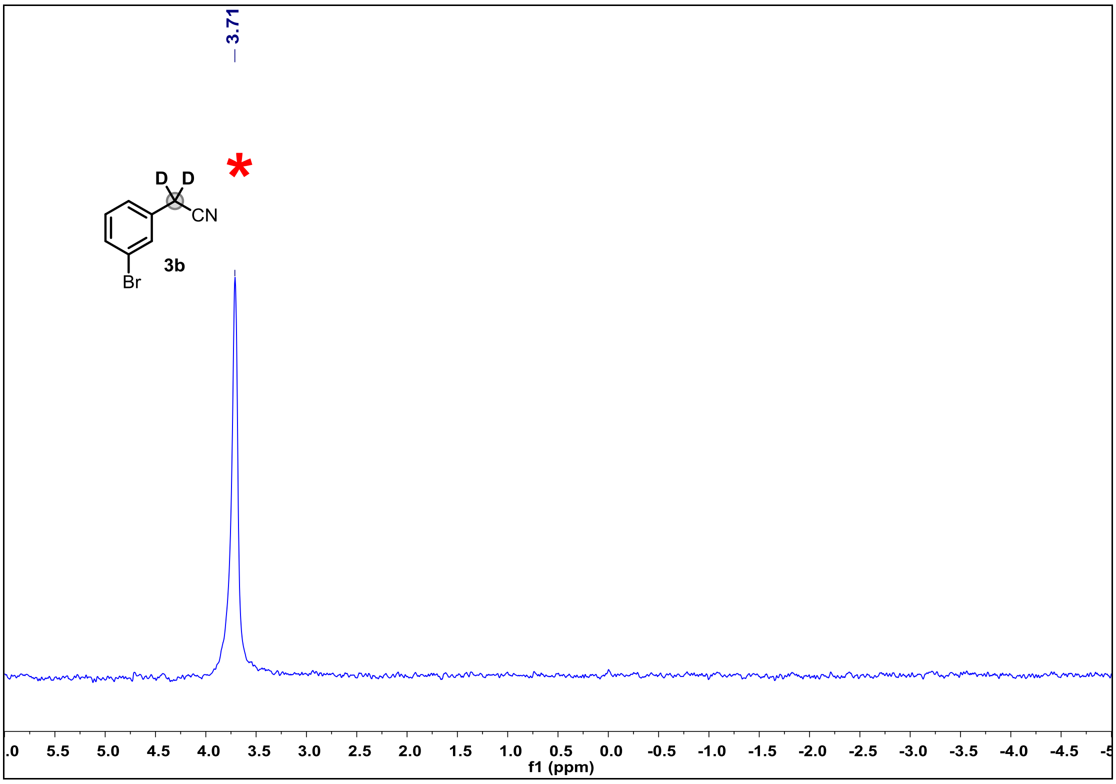

Figure S85. ^{2}H NMR spectrum of α- deuterated nitrile **3b** (77 MHz, $CDCl_3$, 298 K).

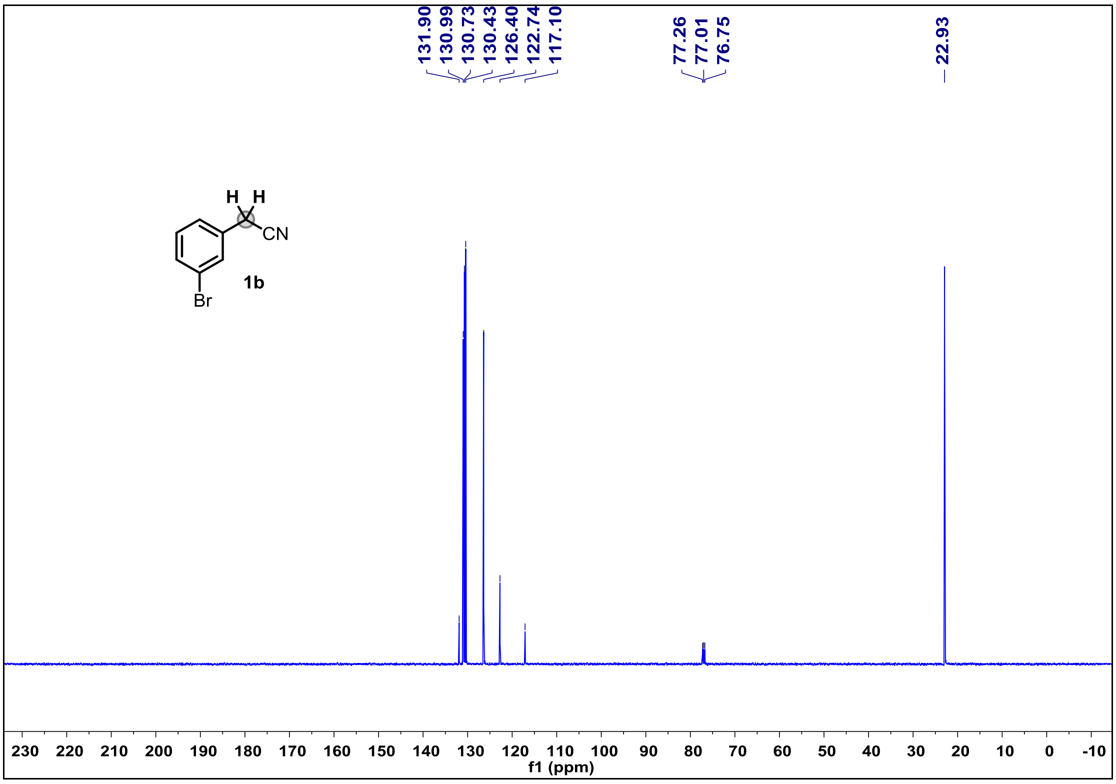

Figure S86. $^{13}C\{^{1}H\}$ NMR spectrum of nitrile **1b** (500 MHz, $CDCl_3$, 298 K).

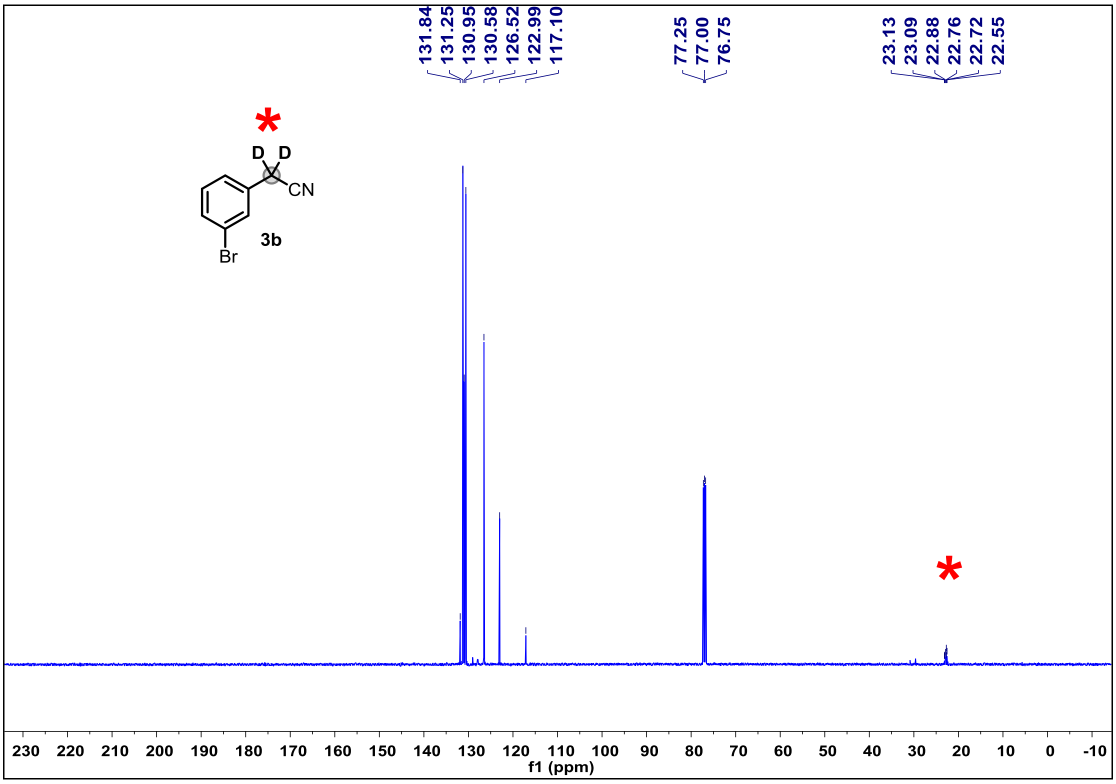

Figure S87. $^{13}C\{^{1}H\}$ NMR spectrum of α- deuterated nitrile **3b** (125 MHz, $CDCl_3$, 298 K).

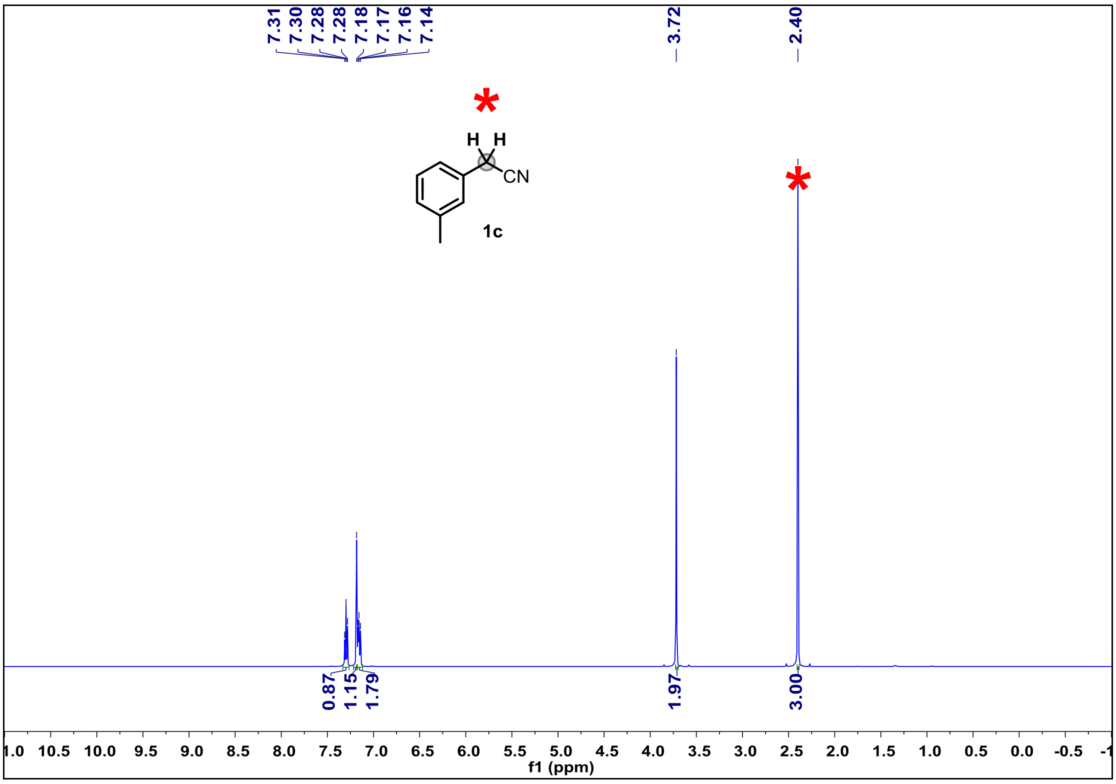

Figure S88. ^{1}H NMR spectrum of nitrile **1c** (500 MHz, $CDCl_3$, 298 K).

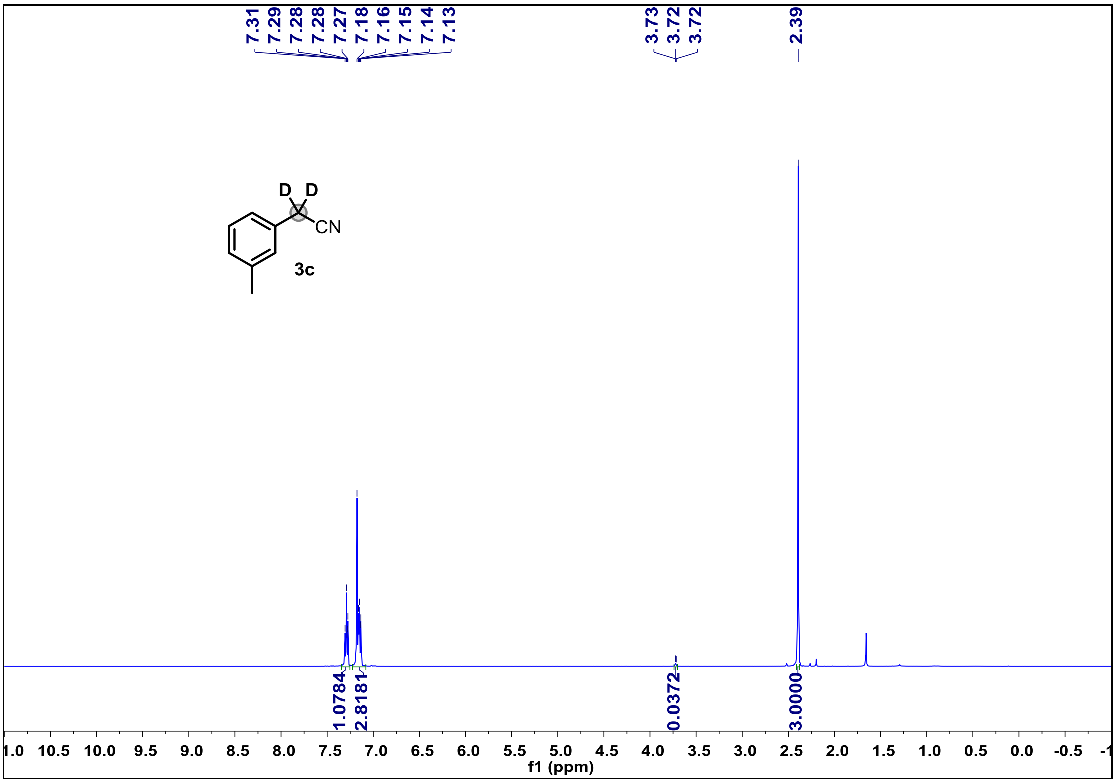

Figure S89. ^{1}H NMR spectrum of α- deuterated nitrile **3c** (125 MHz, $CDCl_3$, 298 K).

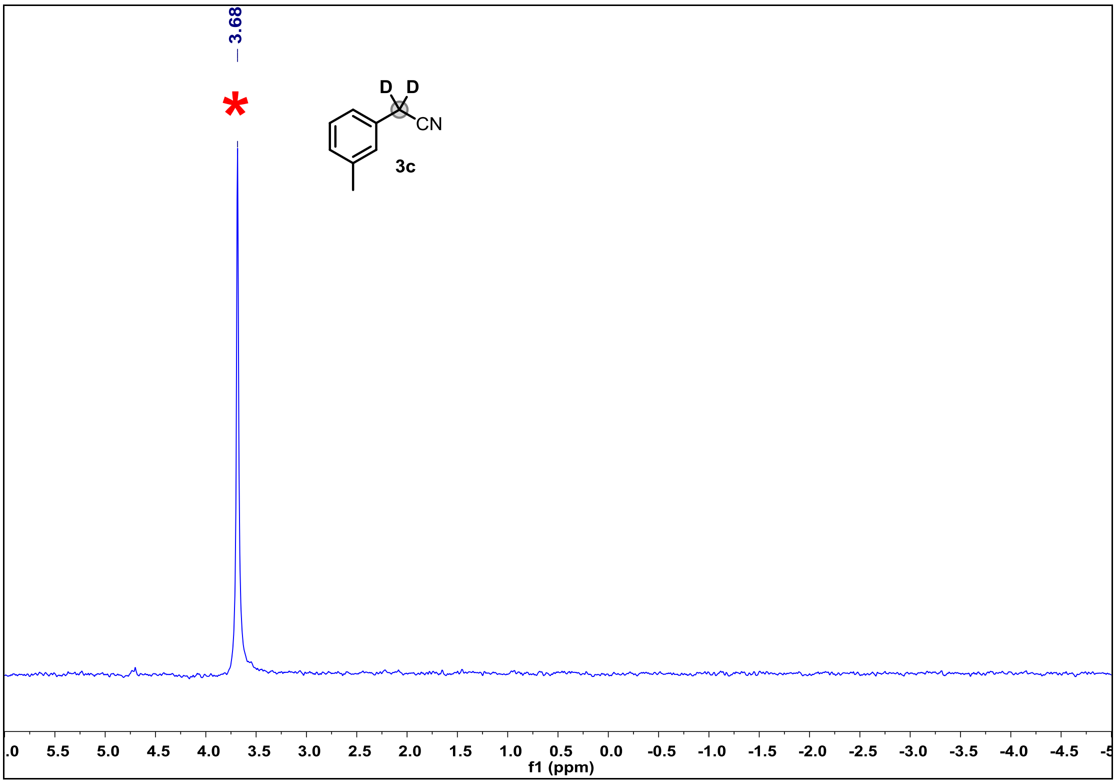

Figure S90. ^{2}H NMR spectrum of α- deuterated nitrile **3c** (77 MHz, $CDCl_3$, 298 K).

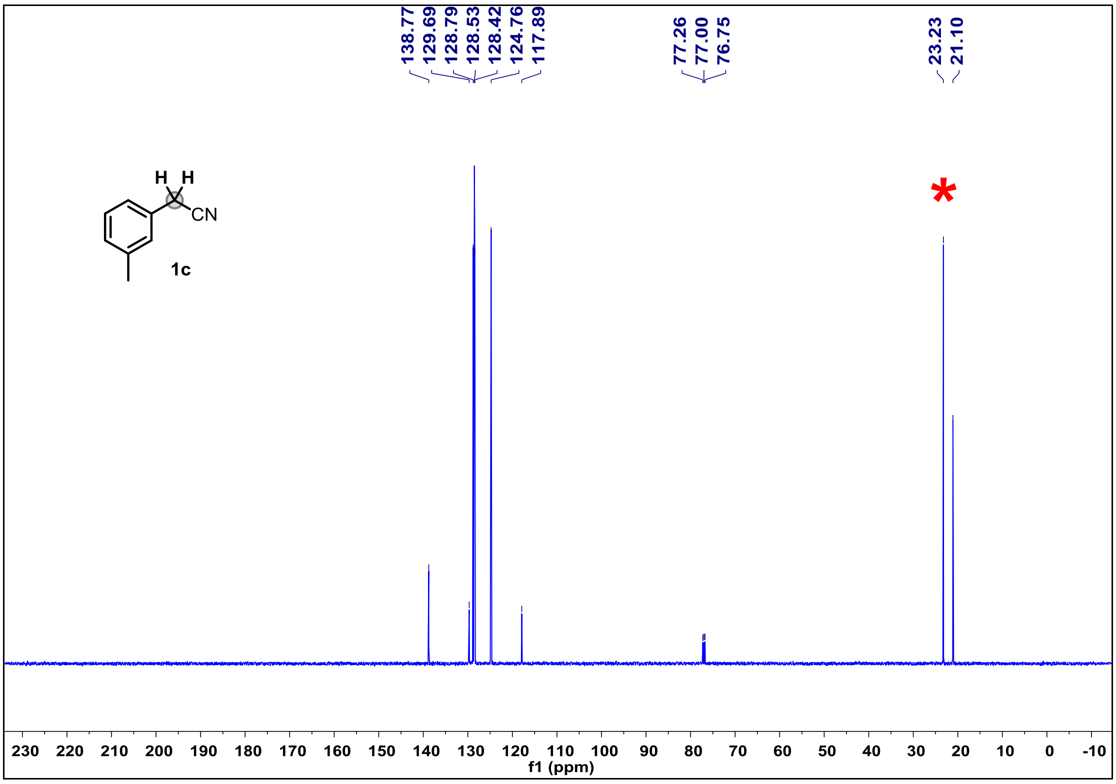

Figure S91. $^{13}C\{^1H\}$ NMR spectrum of nitrile **1c** (125 MHz, $CDCl_3$, 298 K).

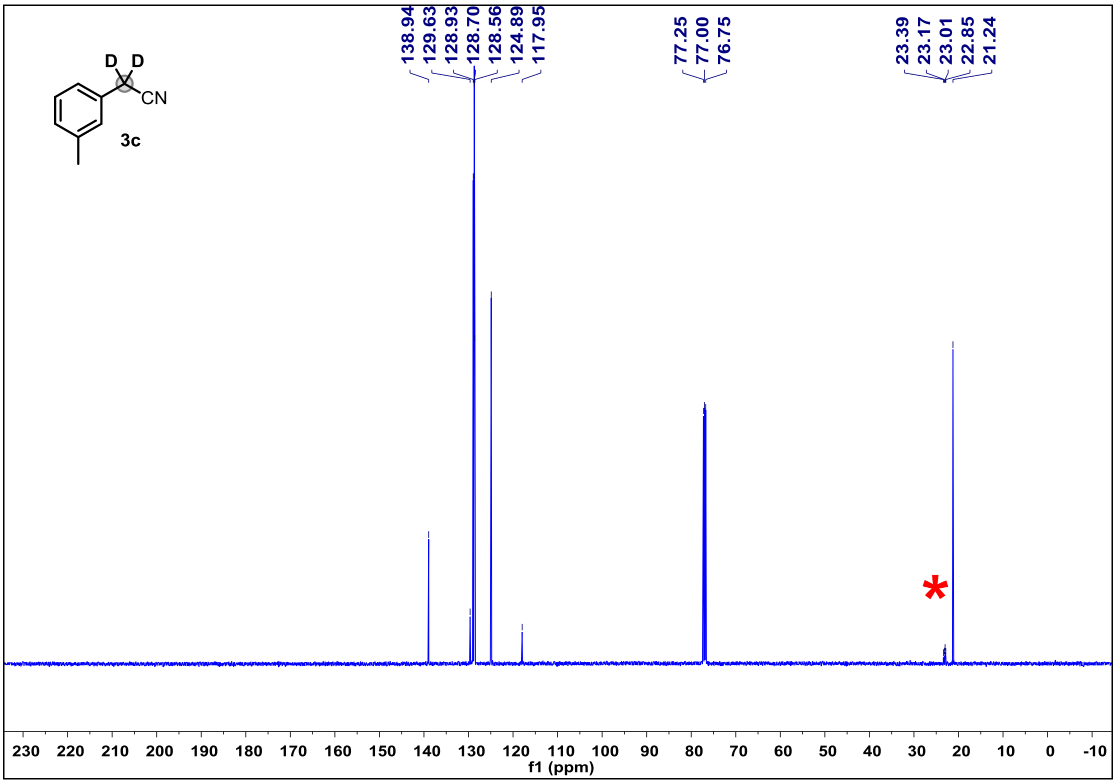

Figure S92. $^{13}C\{^1H\}$ NMR spectrum of α- deuterated nitrile **3c** (125 MHz, $CDCl_3$, 298 K).

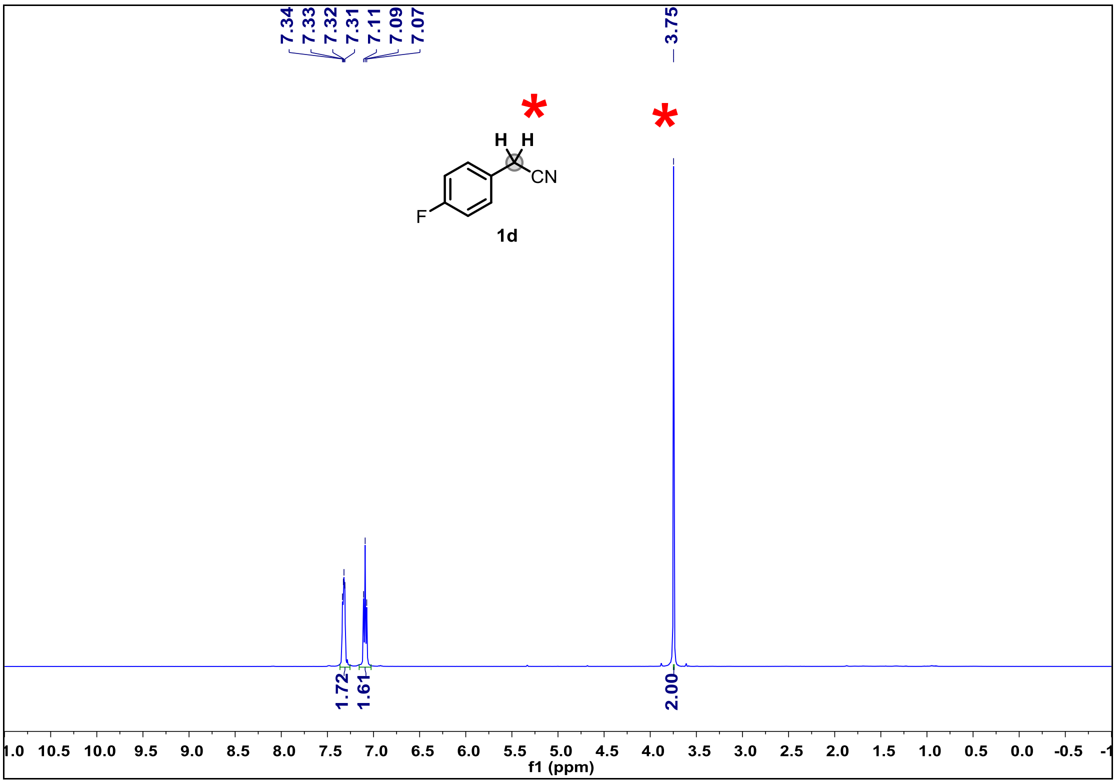

Figure S93. ^{1}H NMR spectrum of nitrile **1d** (500 MHz, $CDCl_3$, 298 K).

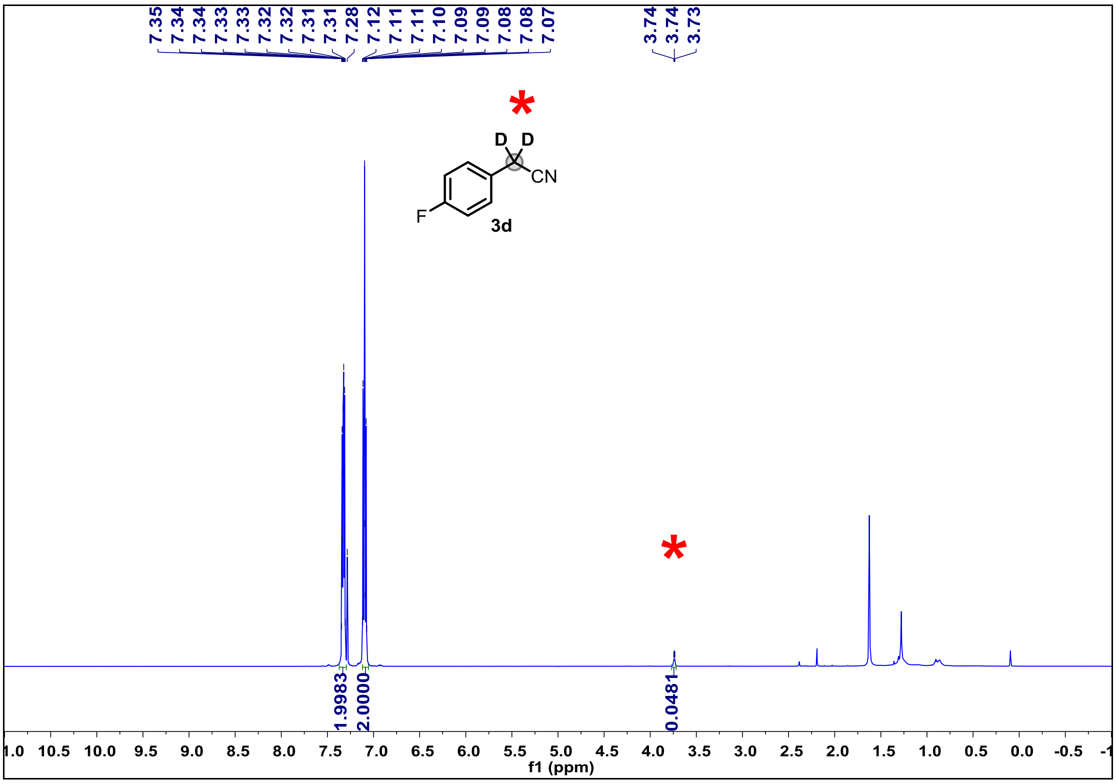

Figure S94. ^{1}H NMR spectrum of α- deuterated nitrile **3d** (500 MHz, $CDCl_3$, 298 K).

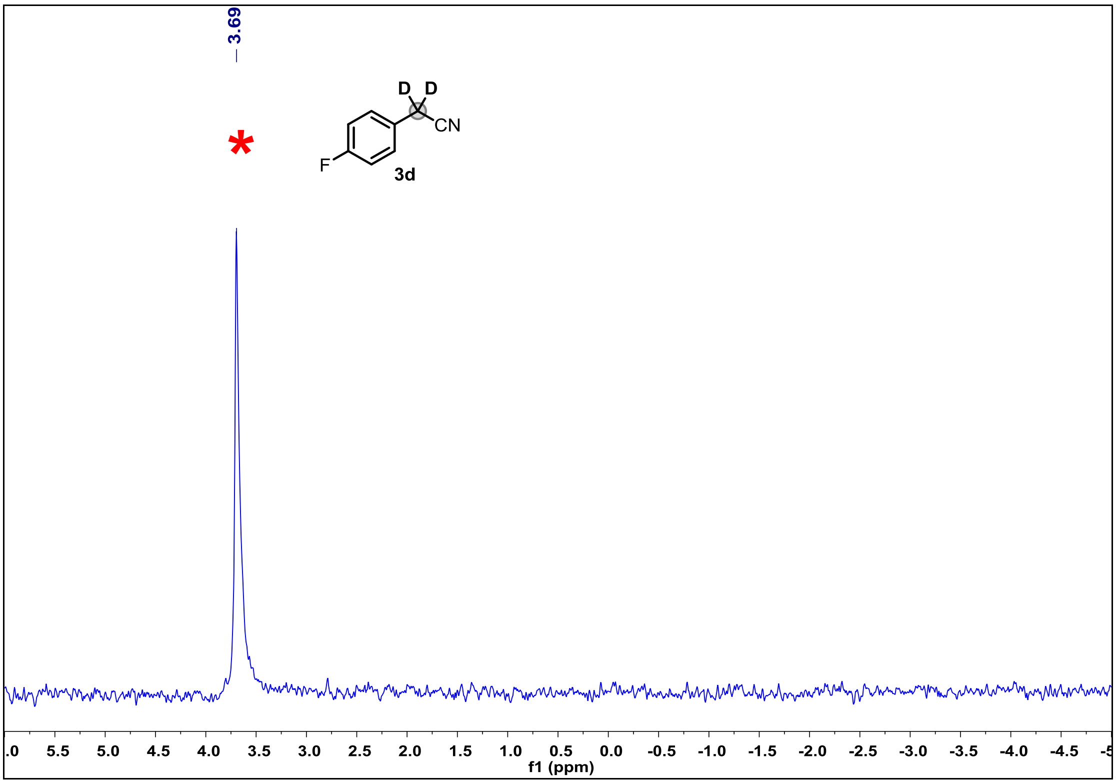

Figure S95. ^{2}H NMR spectrum of α- deuterated nitrile **3d** (77 MHz, $CDCl_3$, 298 K).

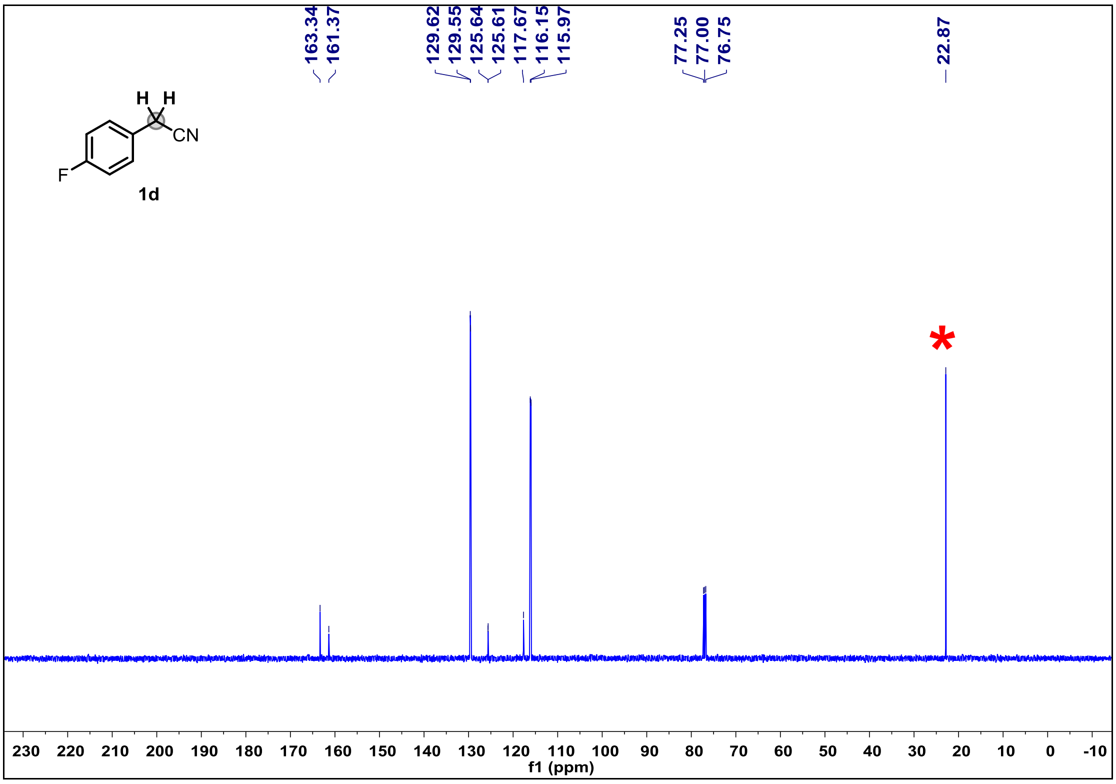

Figure S96. $^{13}C\{^{1}H\}$ NMR spectrum of nitrile **1d** (125 MHz, $CDCl_3$, 298 K).

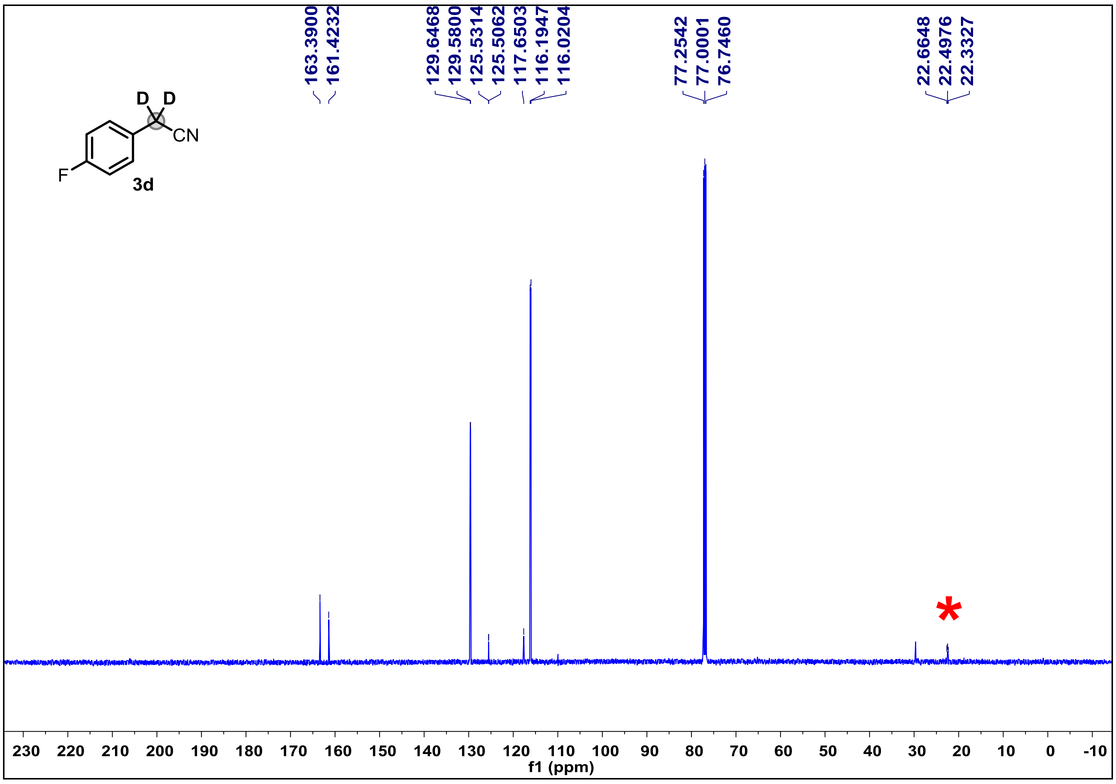

Figure S97. $^{13}C\{^1H\}$ NMR spectrum of α- deuterated nitrile **3d** (125 MHz, $CDCl_3$, 298 K).

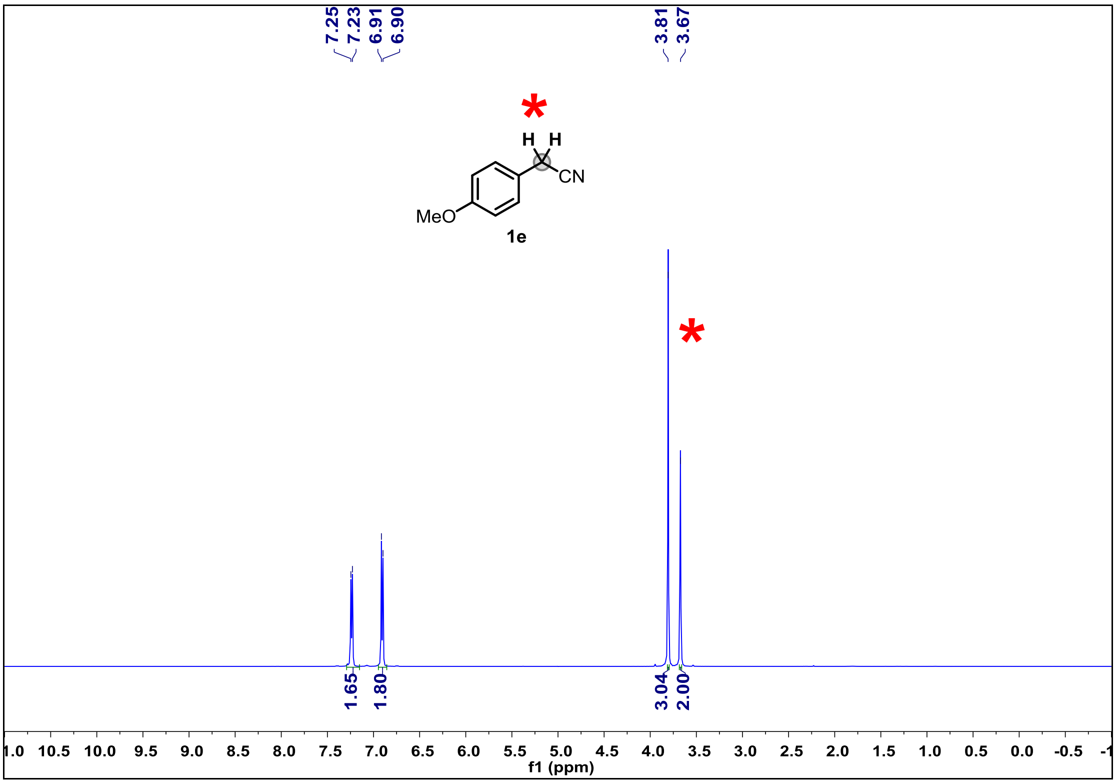

Figure S98. ^1H NMR spectrum of nitrile **1e** (500 MHz, $CDCl_3$, 298 K).

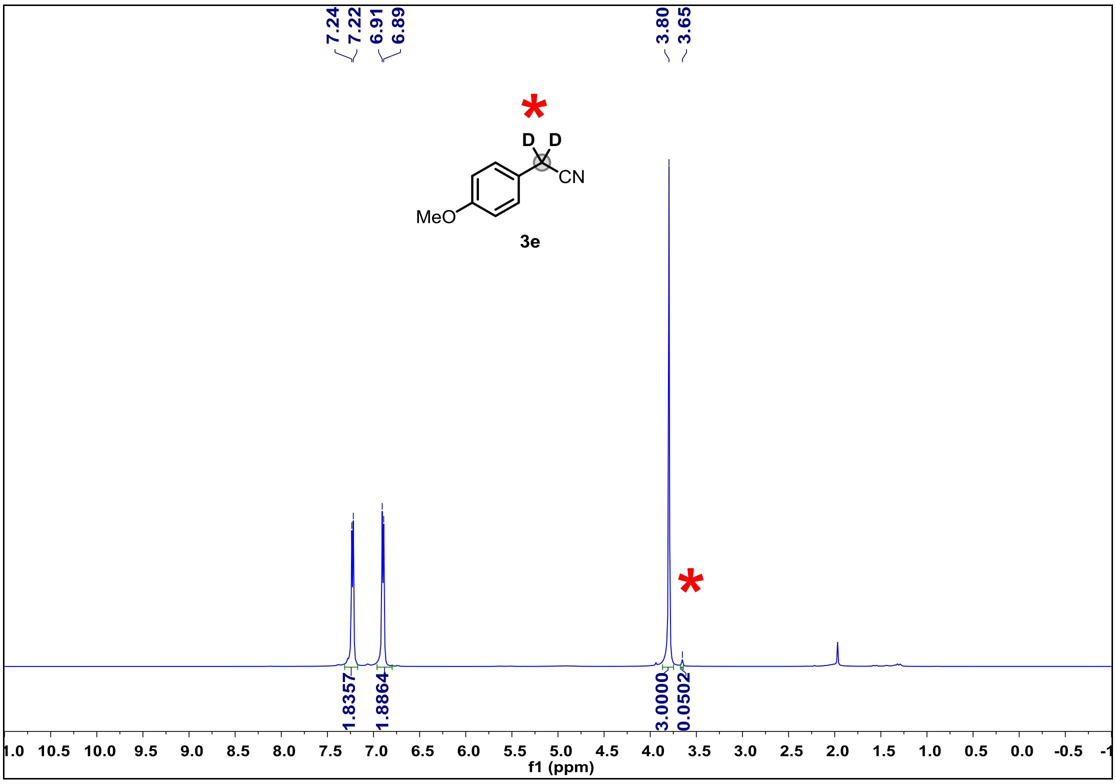

Figure S99. ^{1}H NMR spectrum of α- deuterated nitrile **3e** (125 MHz, $CDCl_3$, 298 K).

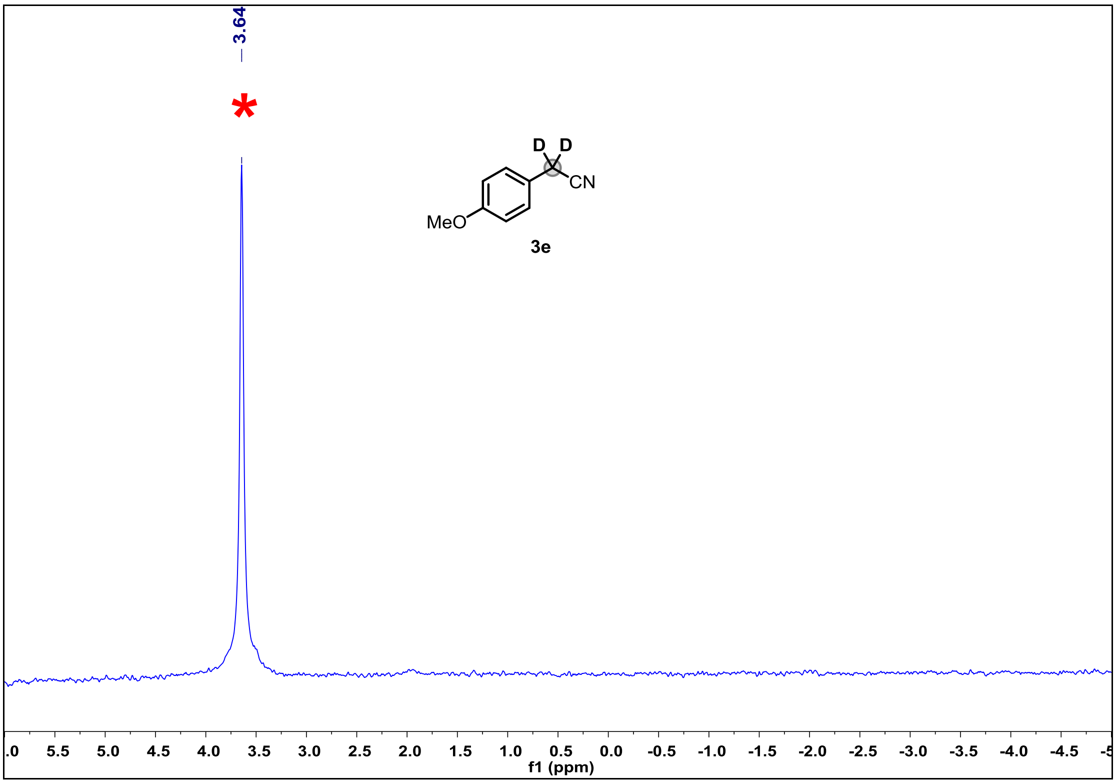

Figure S100. ^{2}H NMR spectrum of α- deuterated nitrile **3e** (77 MHz, $CDCl_3$, 298 K).

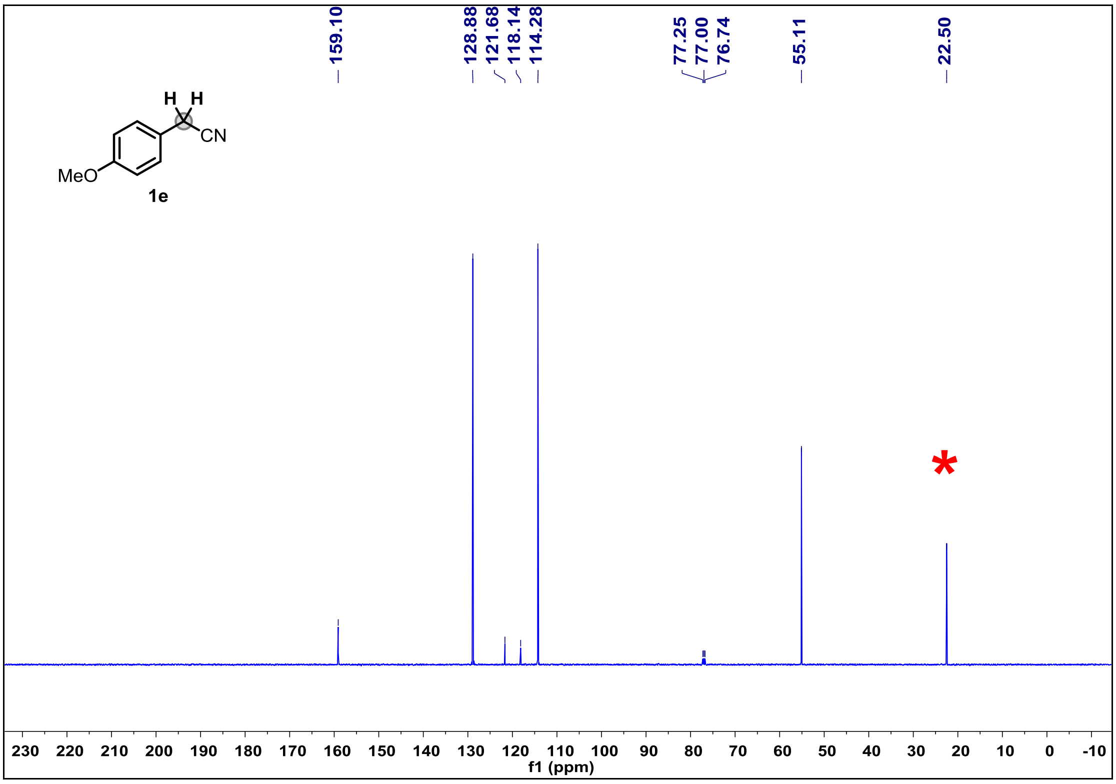

Figure S101. $^{13}C\{^{1}H\}$ NMR spectrum of nitrile **1e** (125 MHz, $CDCl_3$, 298 K).

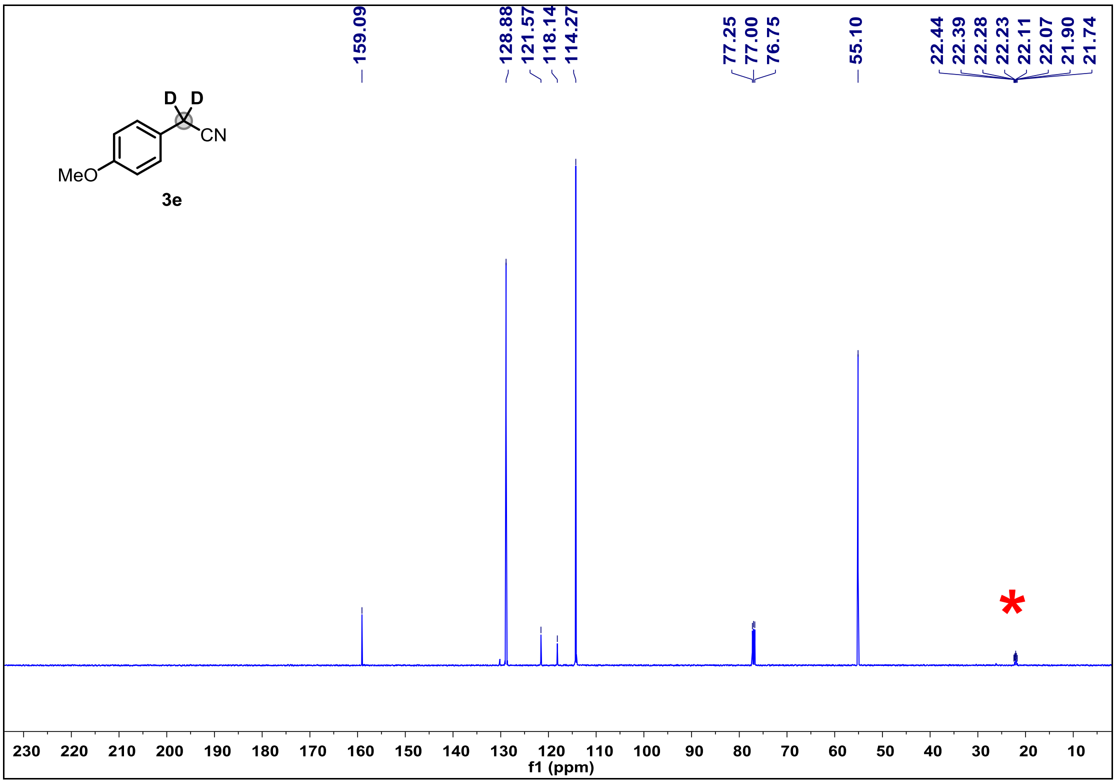

Figure S102. $^{13}C\{^{1}H\}$ NMR spectrum of α- deuterated nitrile **3e** (125 MHz, $CDCl_3$, 298 K).

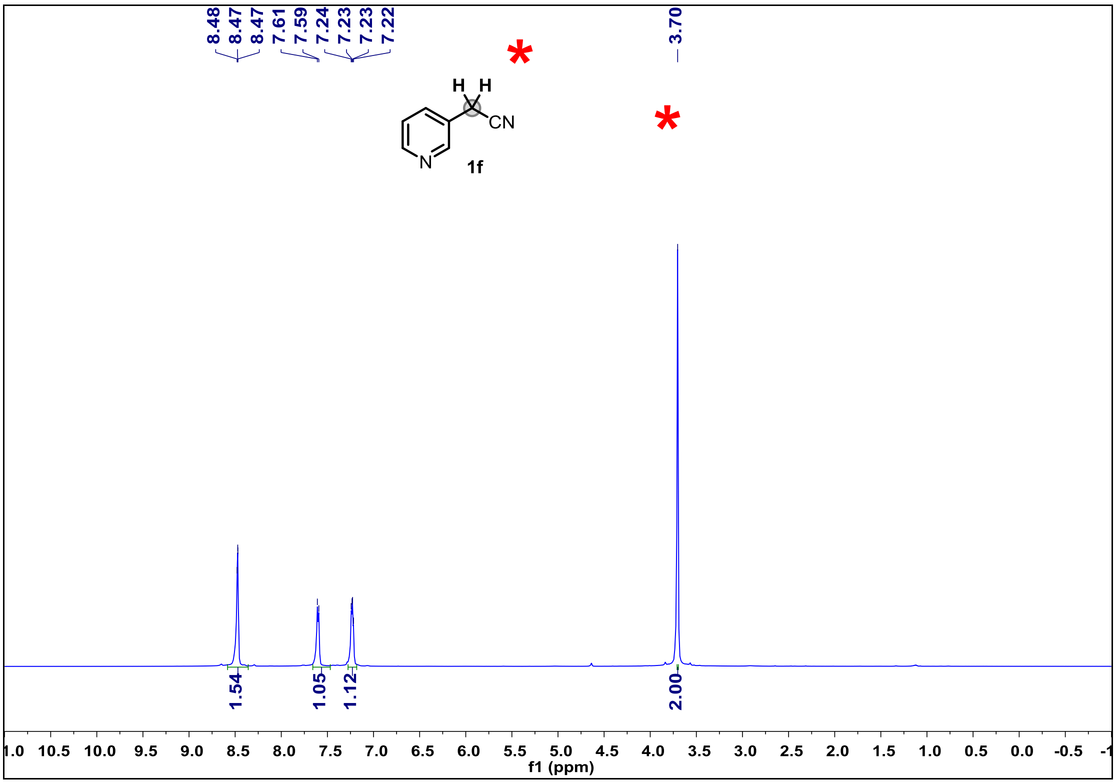

Figure S103. ^{1}H NMR spectrum of nitrile **1f** (500 MHz, $CDCl_3$, 298 K).

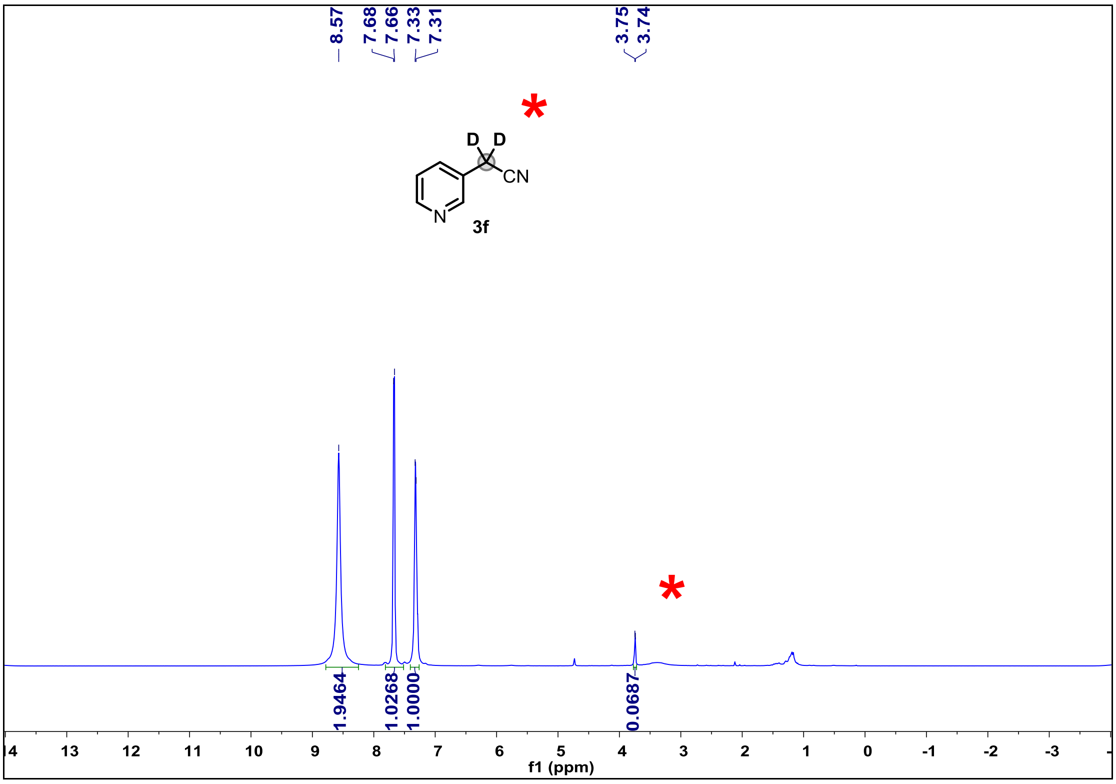

Figure S104. ^{1}H NMR spectrum of α- deuterated nitrile **3e** (125 MHz, $CDCl_3$, 298 K).

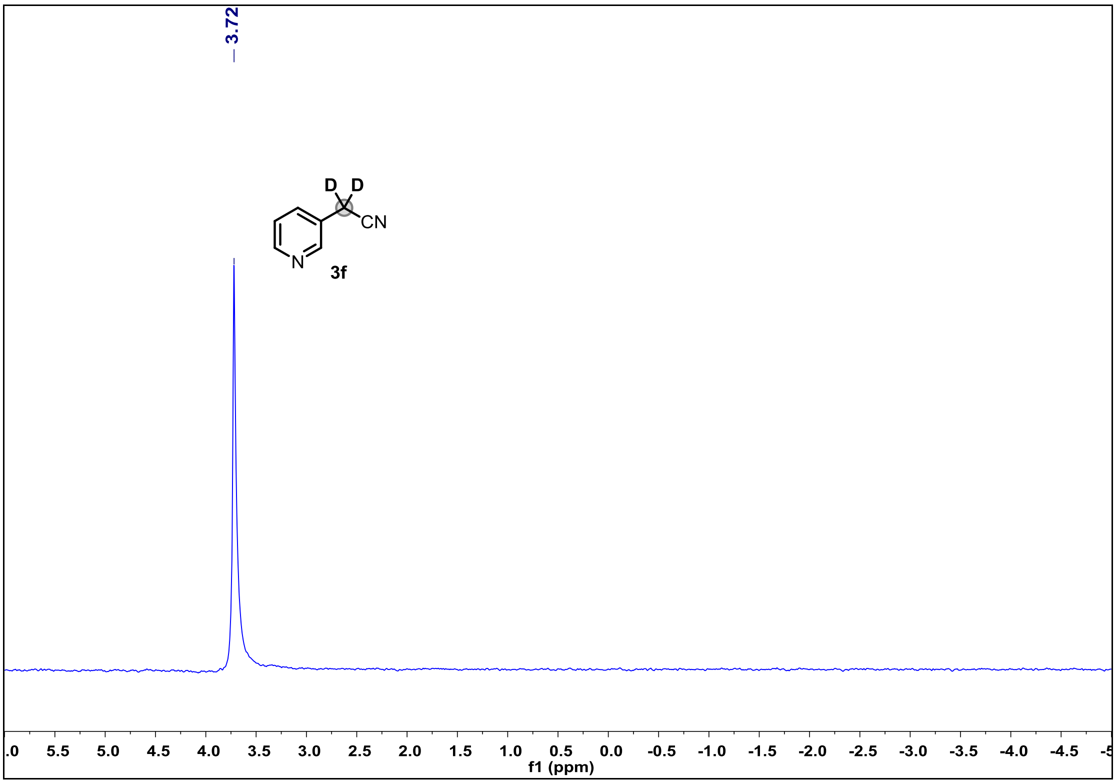

Figure S105. ^{2}H NMR spectrum of α- deuterated nitrile **3f** (77 MHz, $CDCl_3$, 298 K).

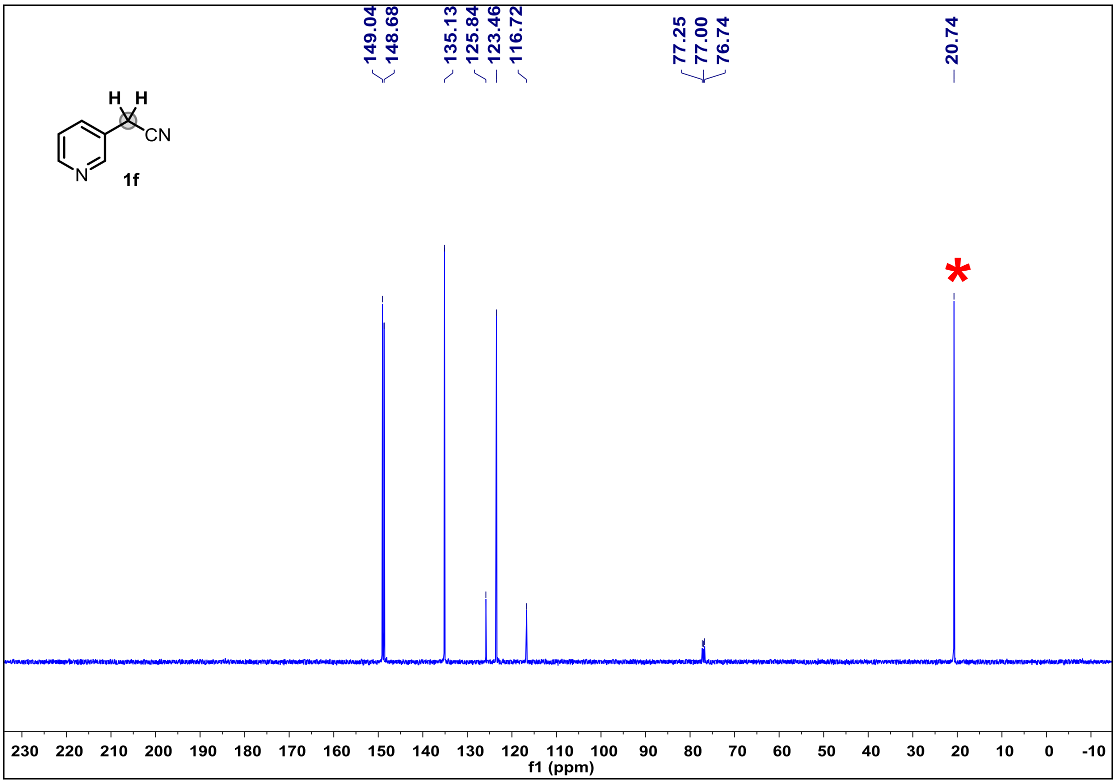

Figure S106. $^{13}C\{^{1}H\}$ NMR spectrum of nitrile **1f** (125 MHz, $CDCl_3$, 298 K).

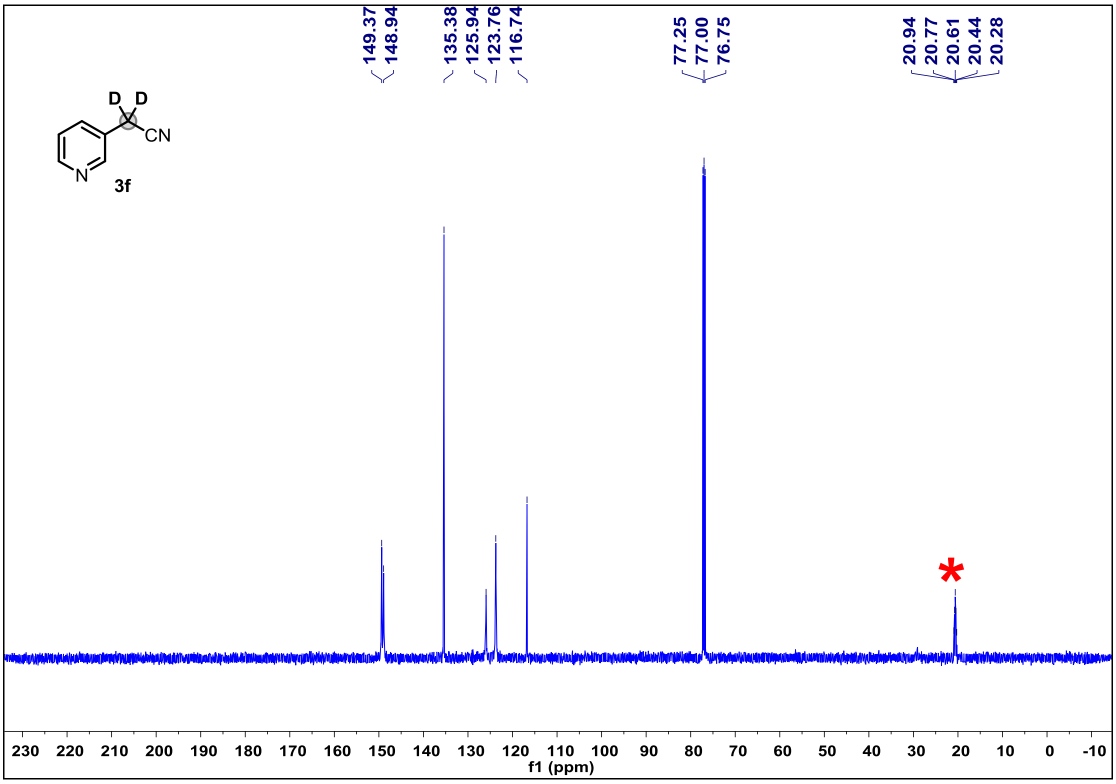

Figure S107. $^{13}C\{^1H\}$ NMR spectrum of α- deuterated nitrile **3f** (125 MHz, $CDCl_3$, 298 K).

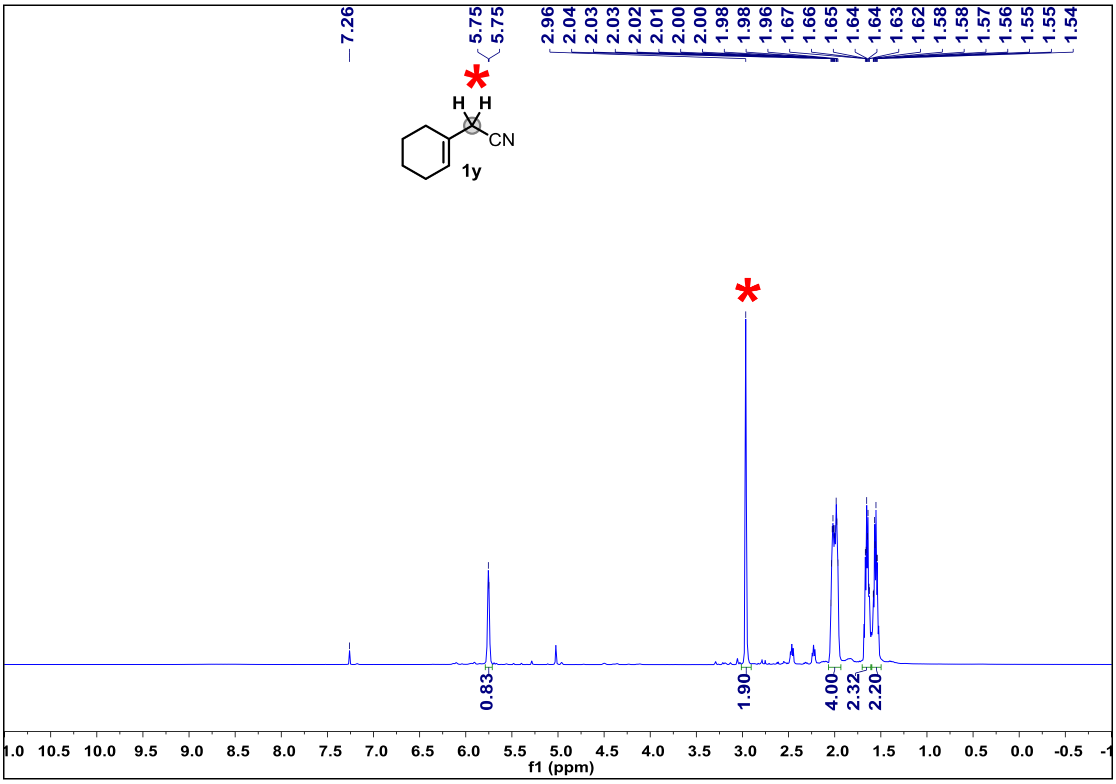

Figure S108. ^1H NMR spectrum of nitrile **1y** (400 MHz, $CDCl_3$, 298 K).

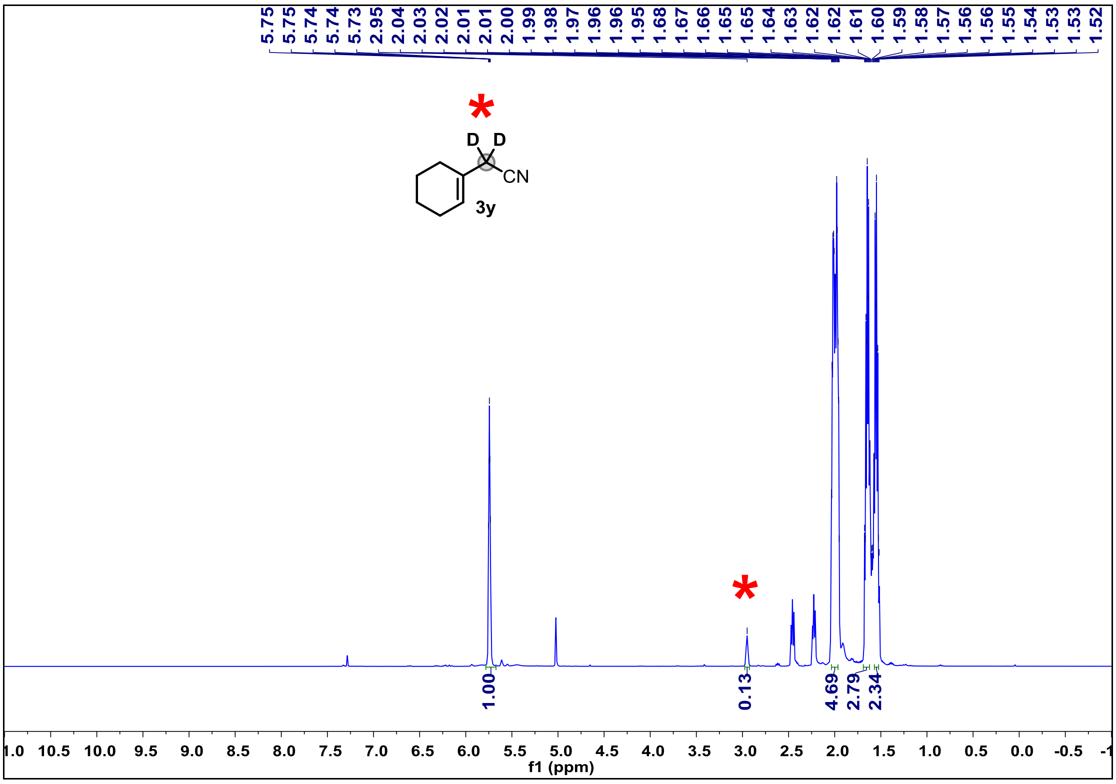

Figure S109. ^{1}H NMR spectrum of α- deuterated nitrile **3y** (125 MHz, $CDCl_3$, 298 K).

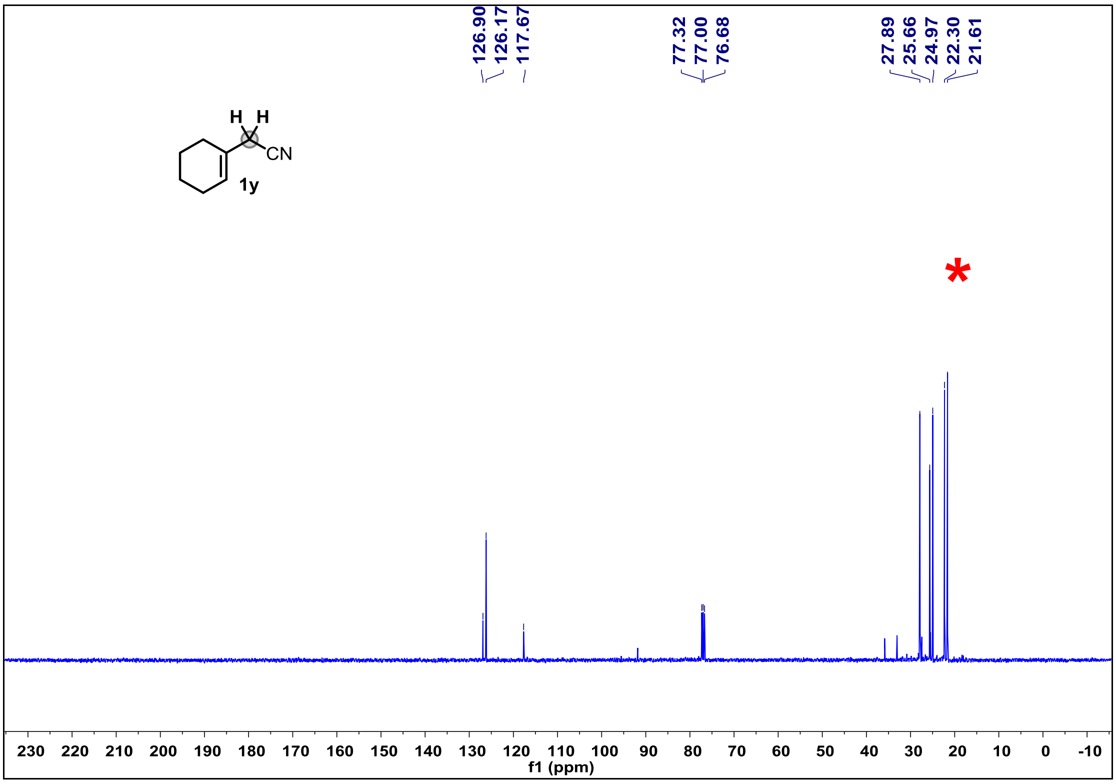

Figure S110. $^{13}C\{^{1}H\}$ NMR spectrum of nitrile **1y** (125 MHz, $CDCl_3$, 298 K).

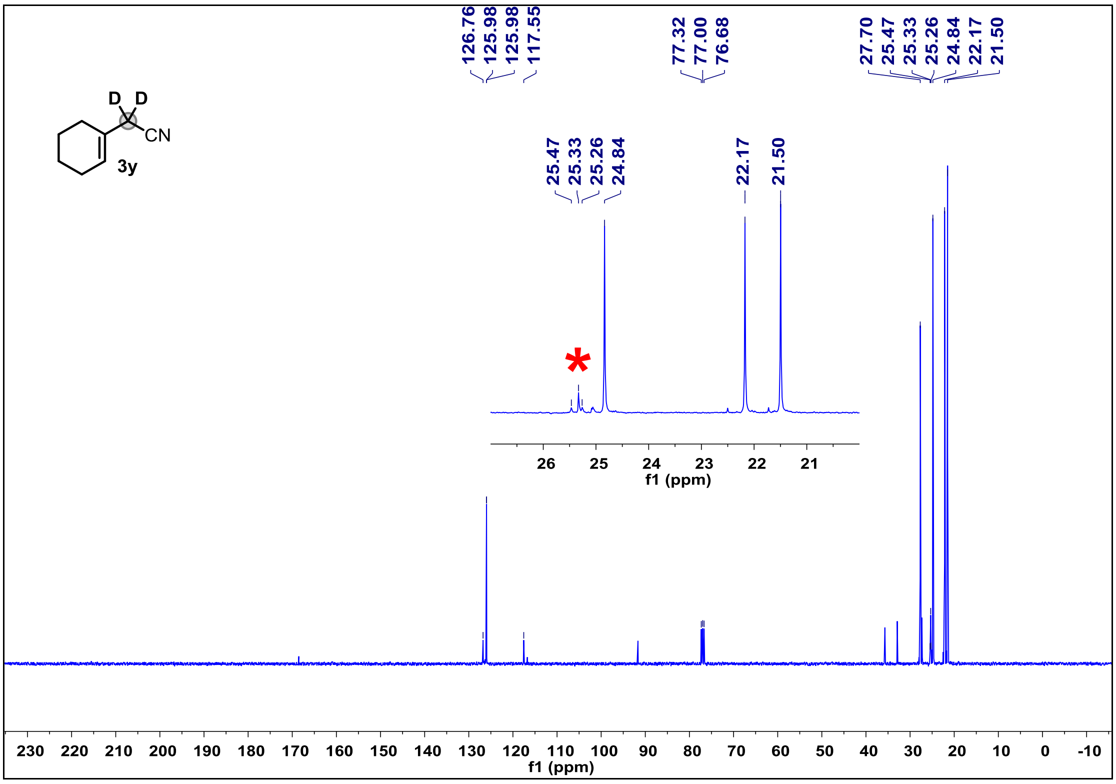

Figure S111. $^{13}C\{^1H\}$ NMR spectrum of α- deuterated nitrile **3y** (125 MHz, $CDCl_3$, 298 K).

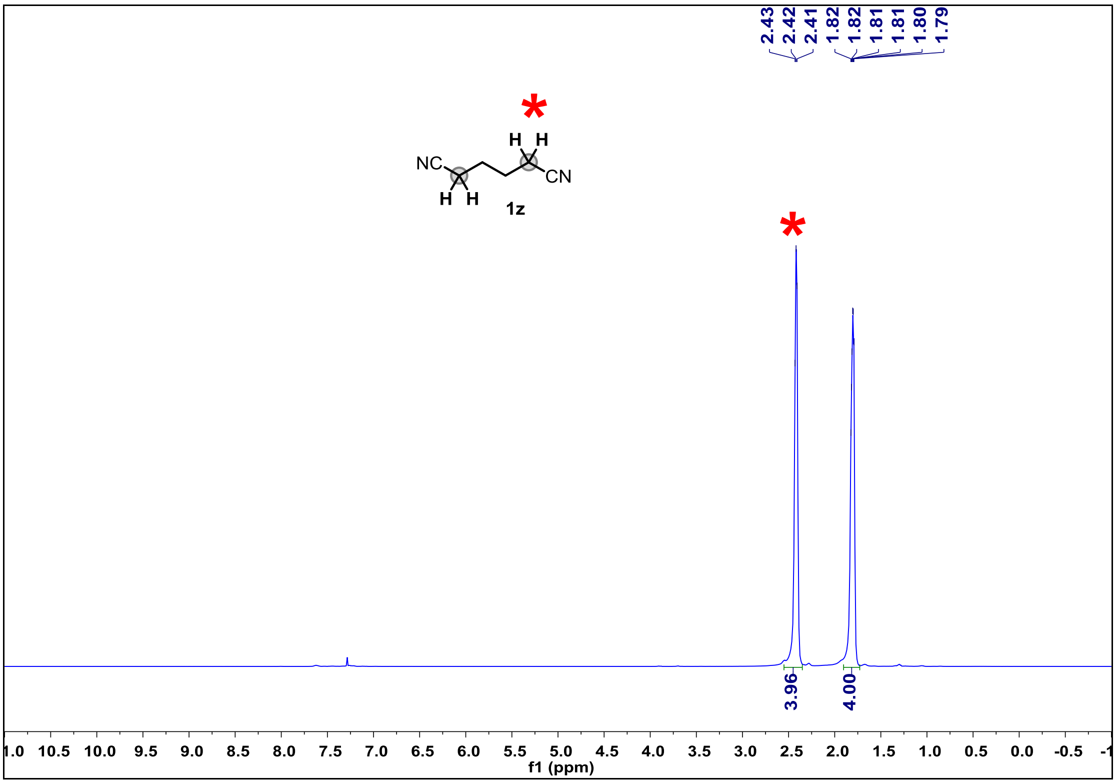

Figure S112. ^1H NMR spectrum of nitrile **1z** (500 MHz, $CDCl_3$, 298 K).

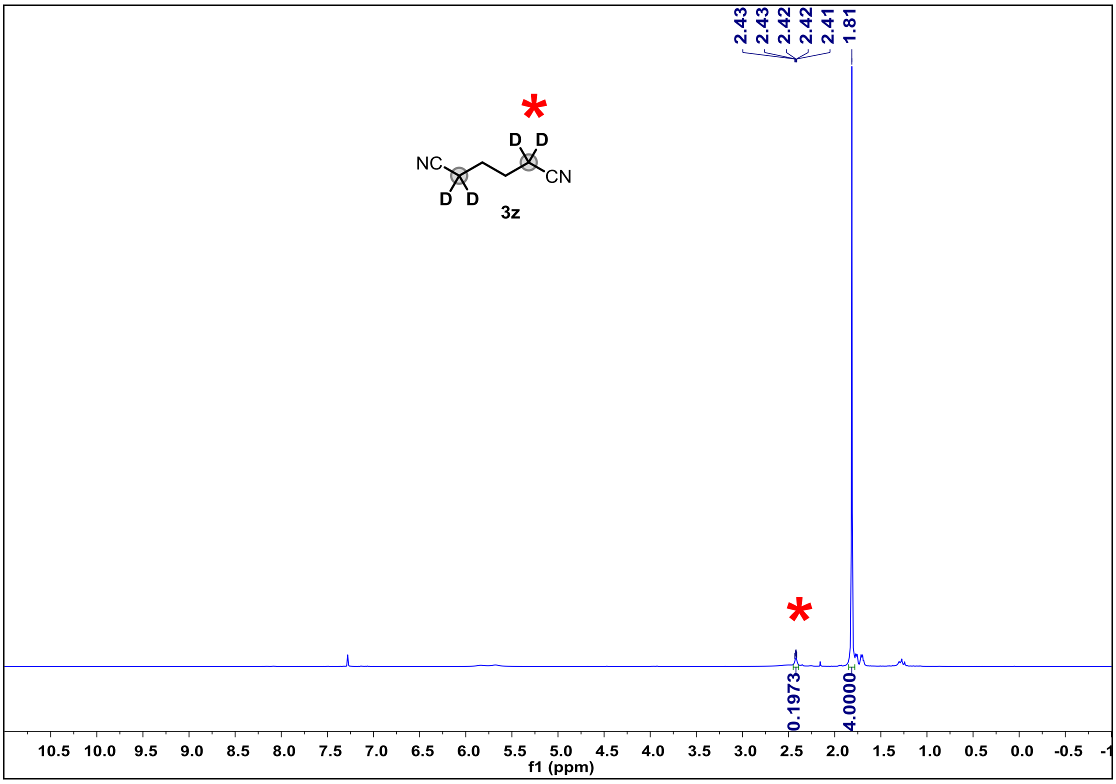

Figure S113. ^{1}H NMR spectrum of α- deuterated nitrile **3z** (125 MHz, $CDCl_3$, 298 K).

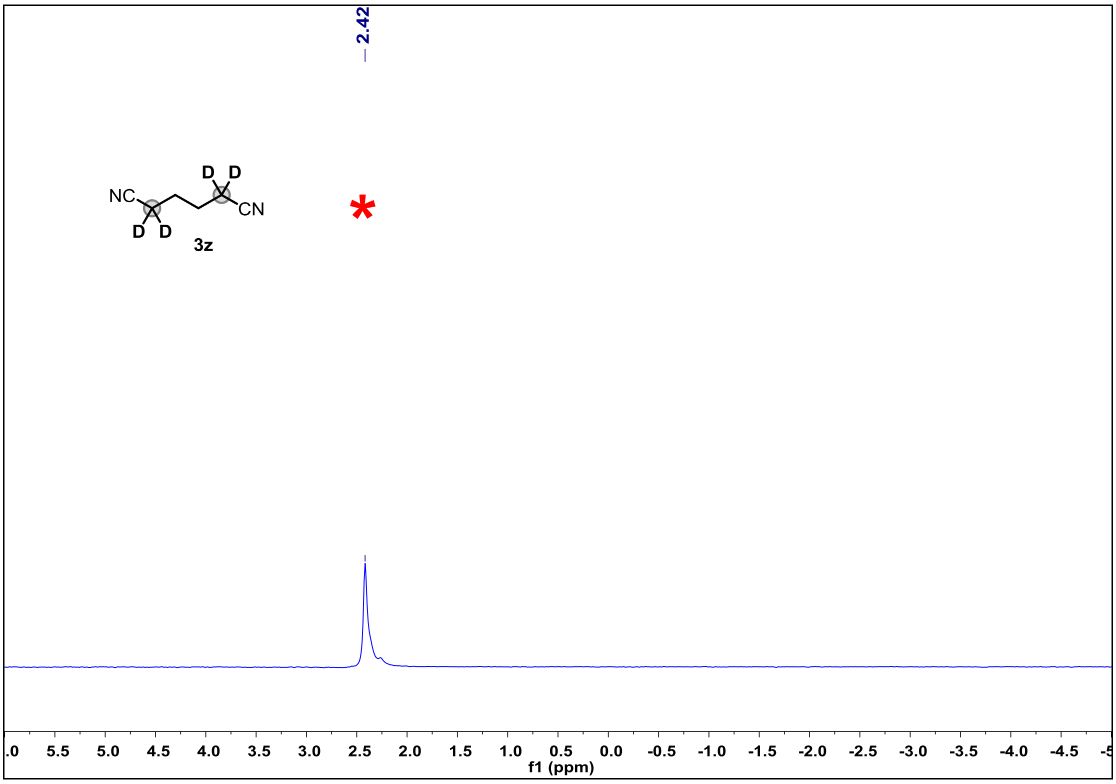

Figure S114. ^{2}H NMR spectrum of α- deuterated nitrile **3z** (77 MHz, $CDCl_3$, 298 K).

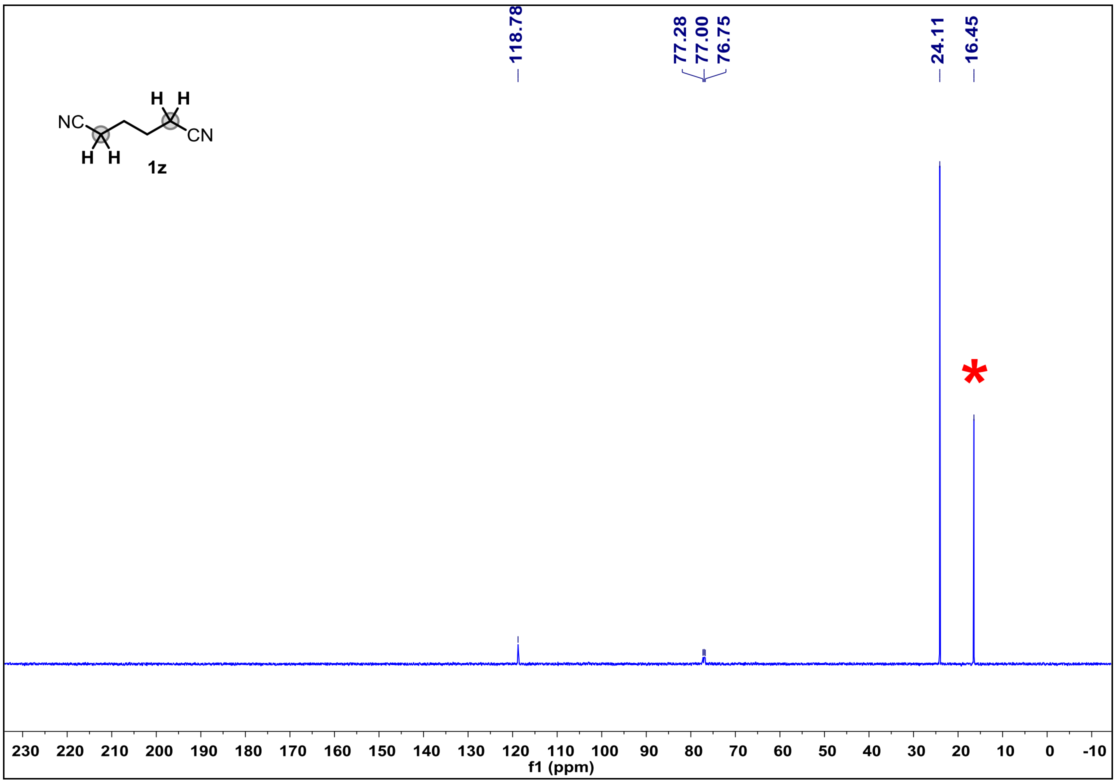

Figure S115. $^{13}C\{^{1}H\}$ NMR spectrum of nitrile **1z** (125 MHz, $CDCl_3$, 298 K).

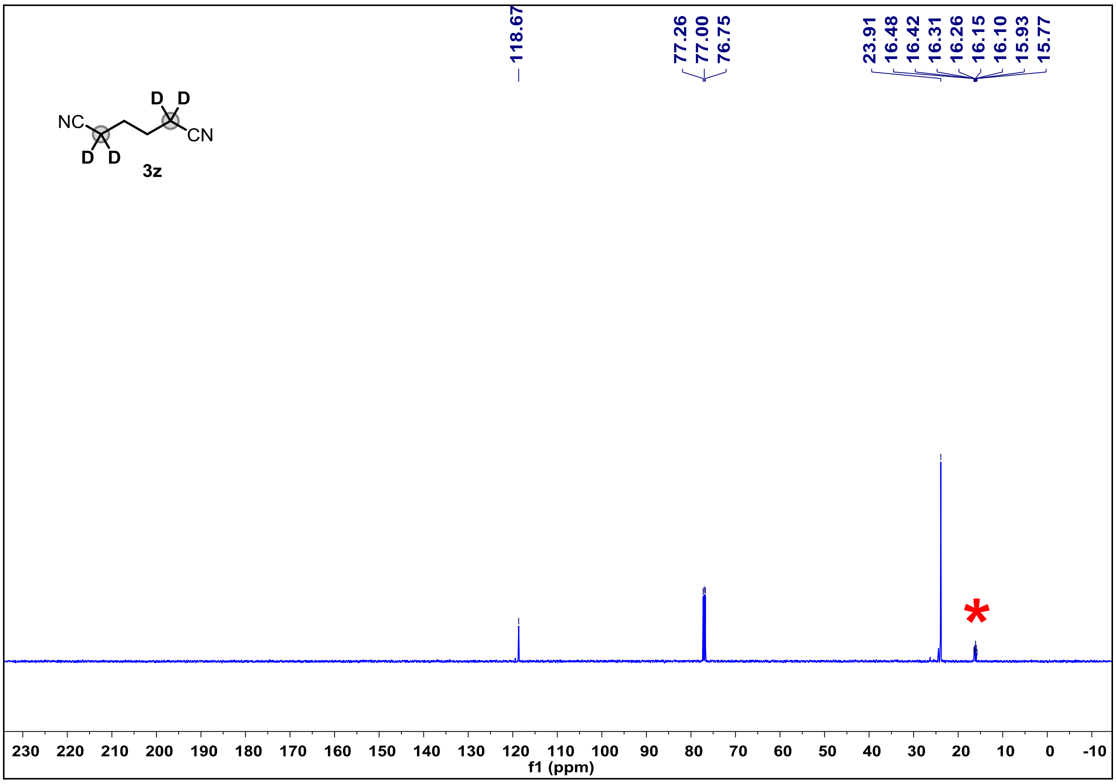

Figure S116. $^{13}C\{^{1}H\}$ NMR spectrum of α- deuterated nitrile **3z** (125 MHz, $CDCl_3$, 298 K).

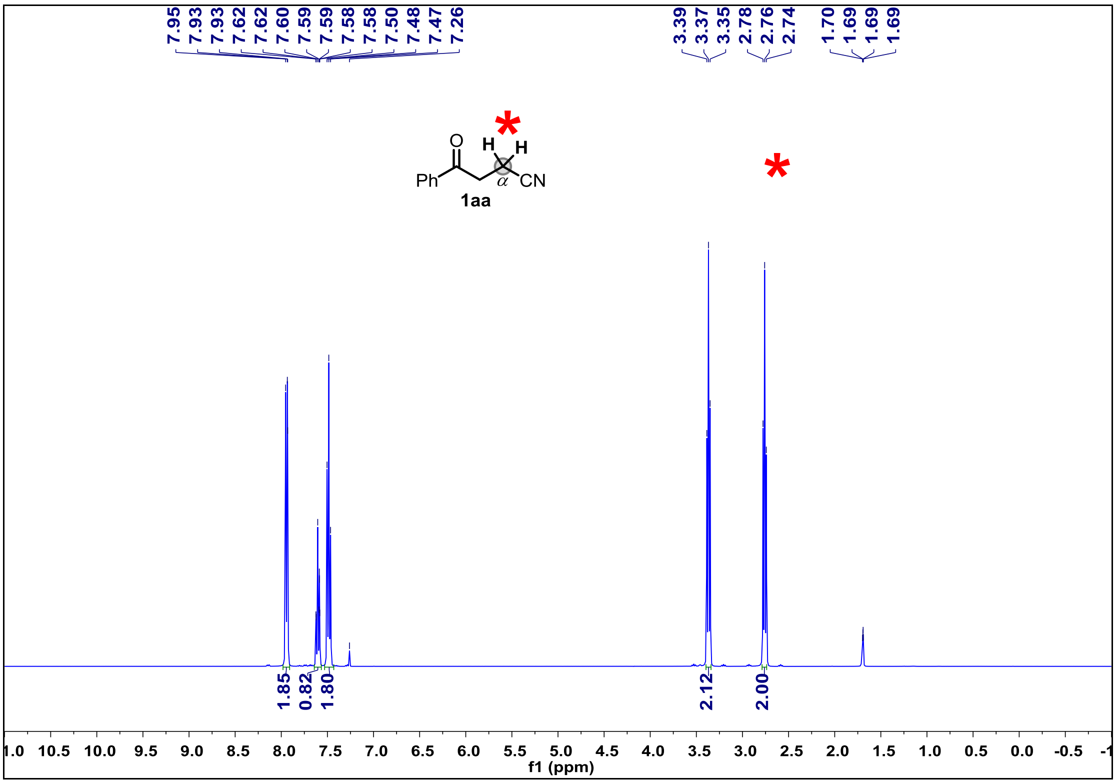

Figure S117. ^{1}H NMR spectrum of nitrile **1aa** (400 MHz, $CDCl_3$, 298 K).

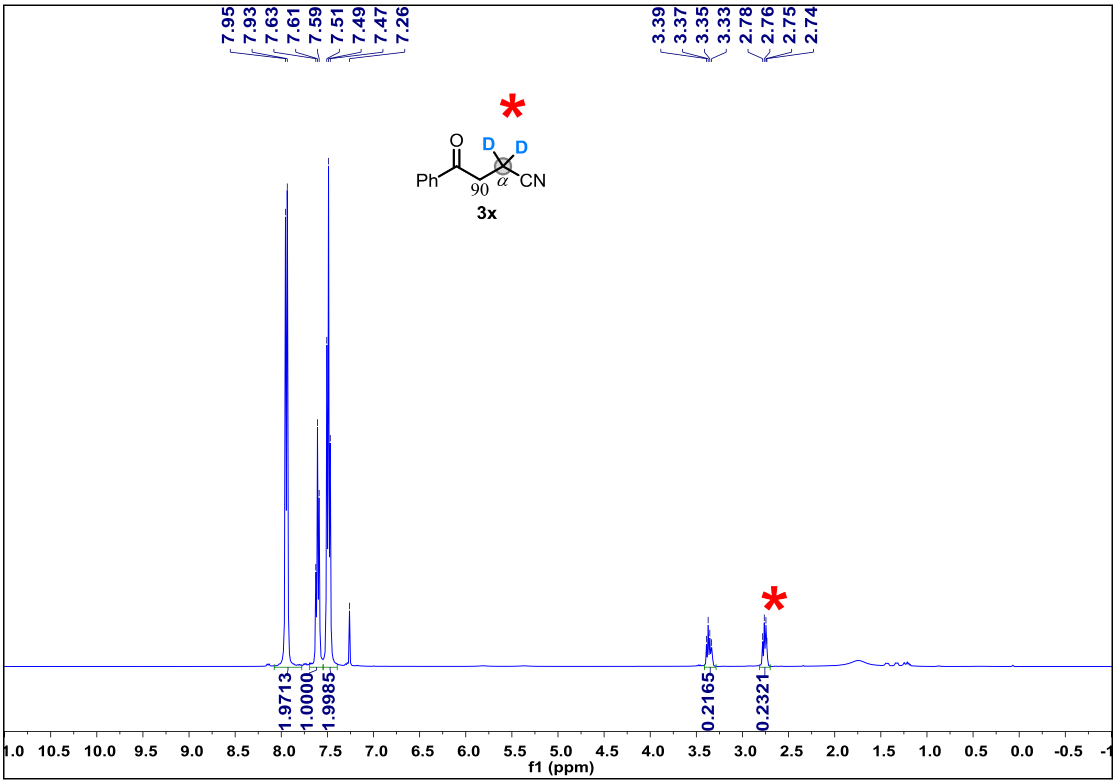

Figure S118. ^{1}H NMR spectrum of α- deuterated nitrile **3aa** (100 MHz, $CDCl_3$, 298 K).

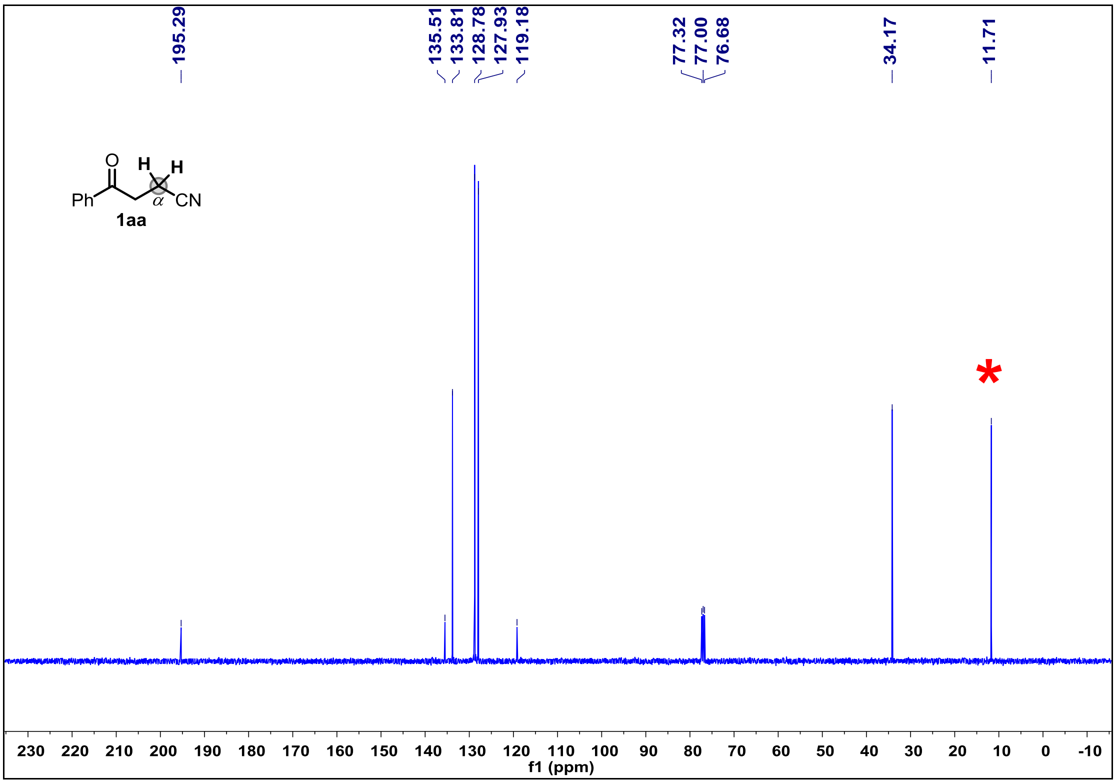

Figure S119. $^{13}C\{^1H\}$ NMR spectrum of nitrile **1aa** (100 MHz, $CDCl_3$, 298 K).

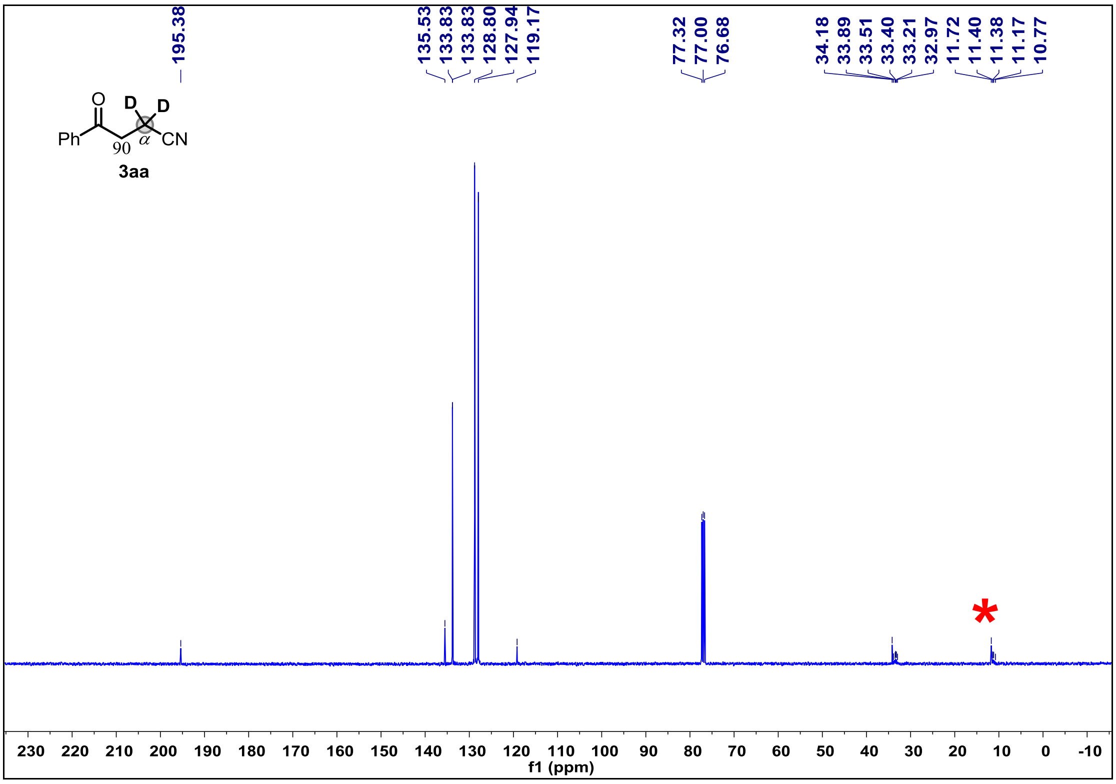

Figure S120. $^{13}C\{^1H\}$ NMR spectrum of α- deuterated nitrile **3aa** (100 MHz, $CDCl_3$, 298 K).

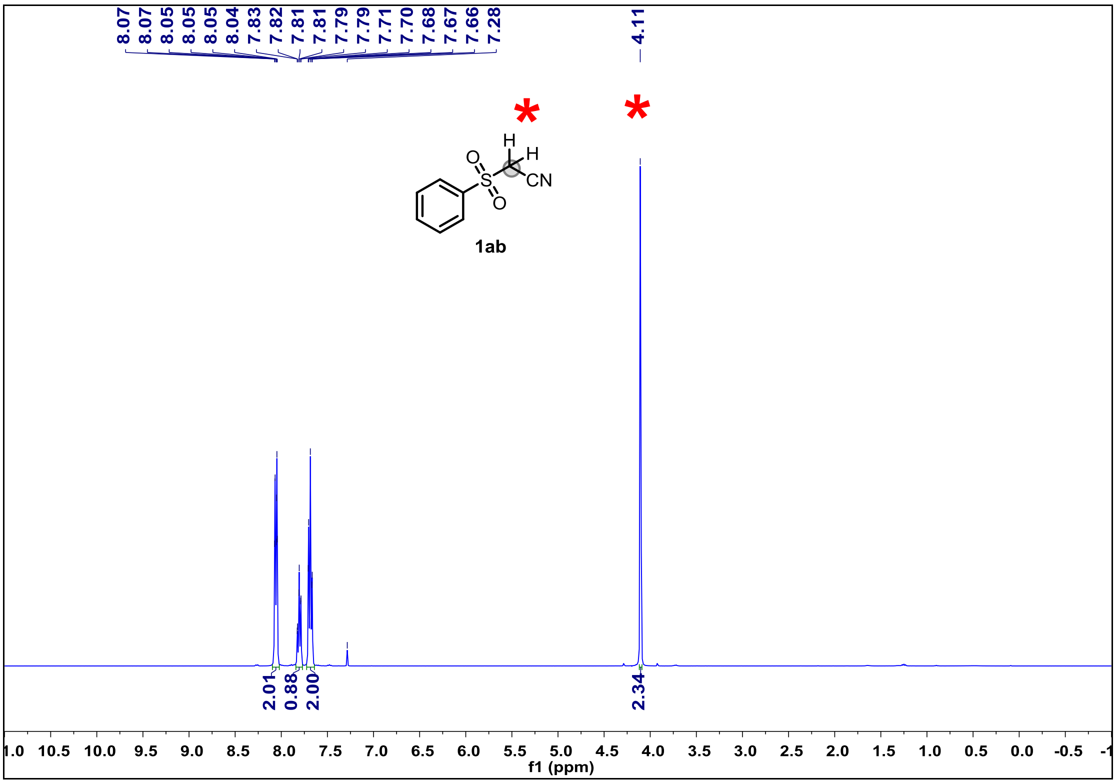

Figure S121. ^{1}H NMR spectrum of nitrile **1ab** (400 MHz, $CDCl_3$, 298 K).

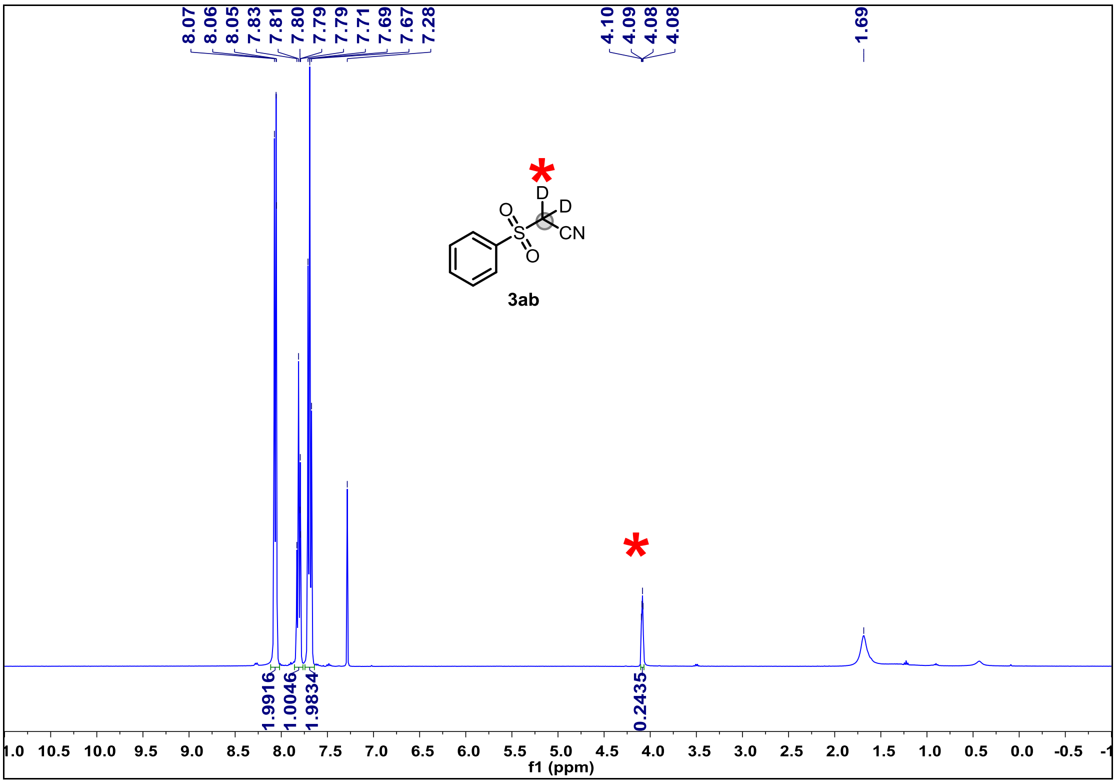

Figure S122. ^{1}H NMR spectrum of α- deuterated nitrile **3ab** (100 MHz, $CDCl_3$, 298 K).

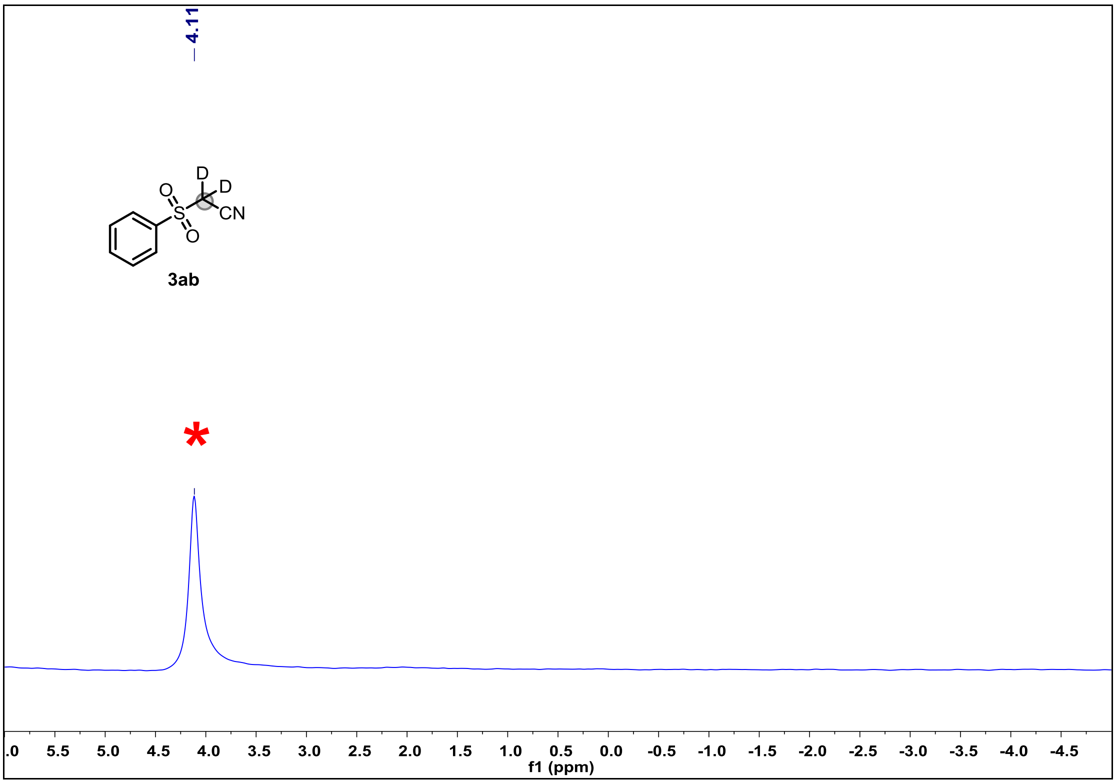

Figure S123. ^{2}H NMR spectrum of α- deuterated nitrile **3ab** (61 MHz, $CDCl_3$, 298 K).

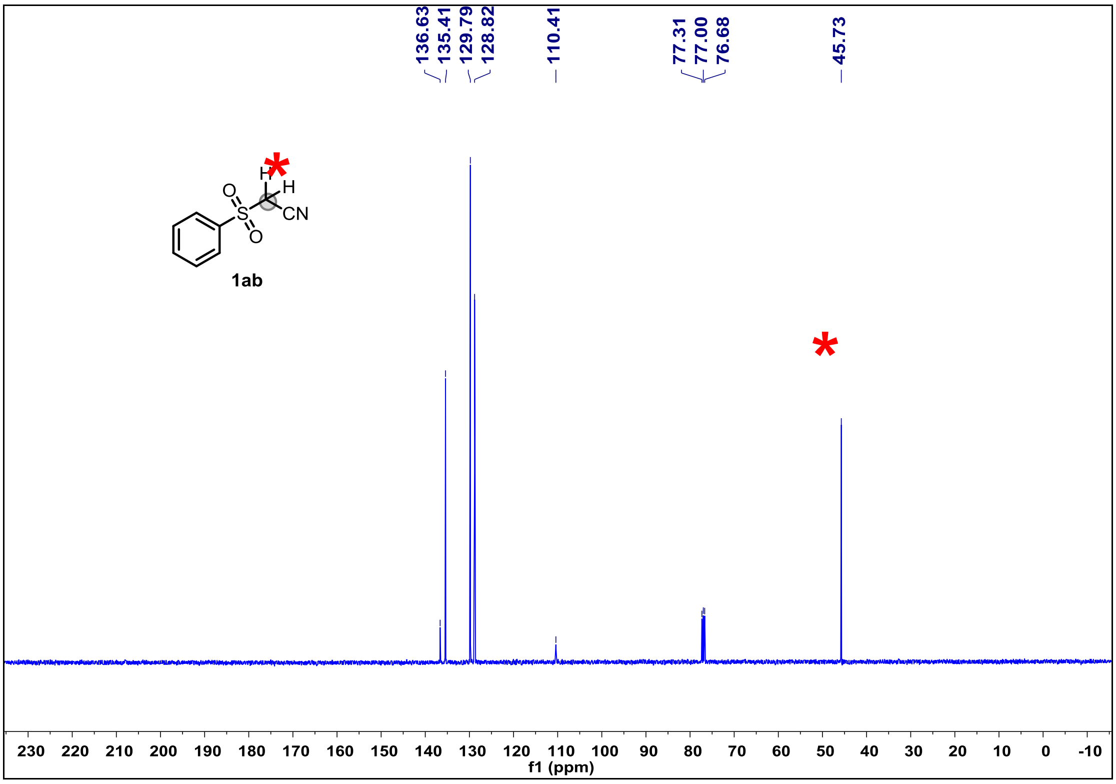

Figure S124. $^{13}C\{^{1}H\}$ NMR spectrum of nitrile **1ab** (125 MHz, $CDCl_3$, 298 K).

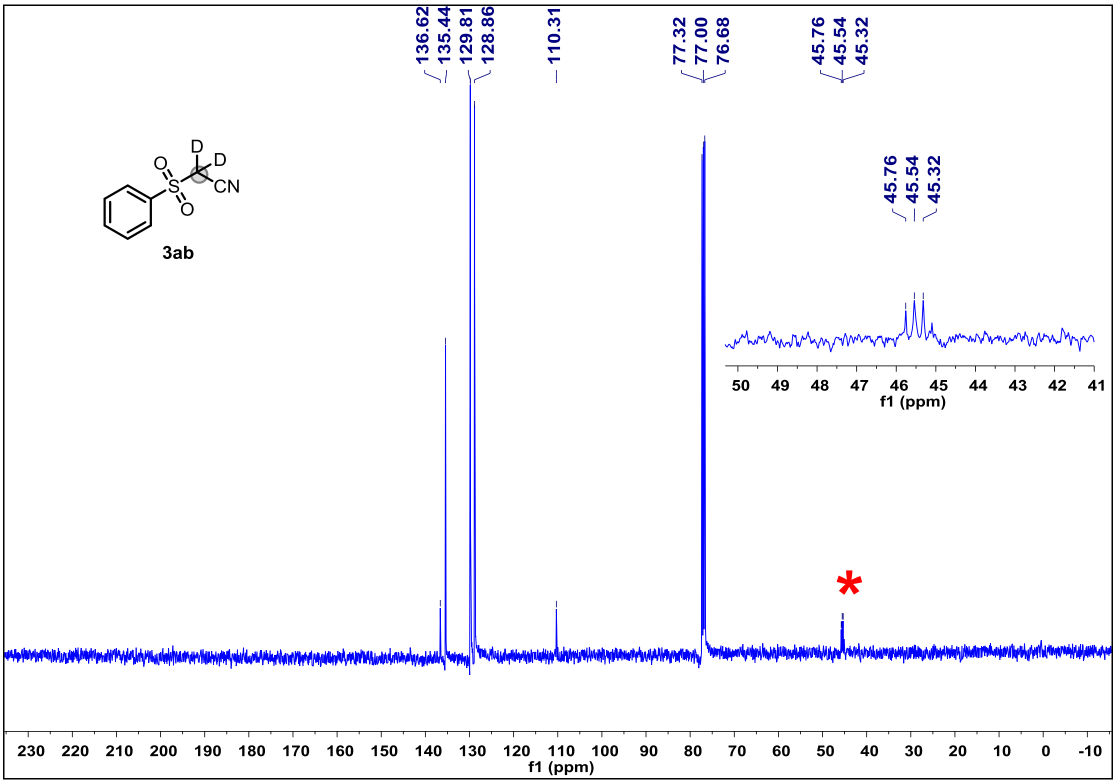

Figure S125. $^{13}C\{^{1}H\}$ NMR spectrum of α- deuterated nitrile **3ab** (100 MHz, $CDCl_3$, 298 K).

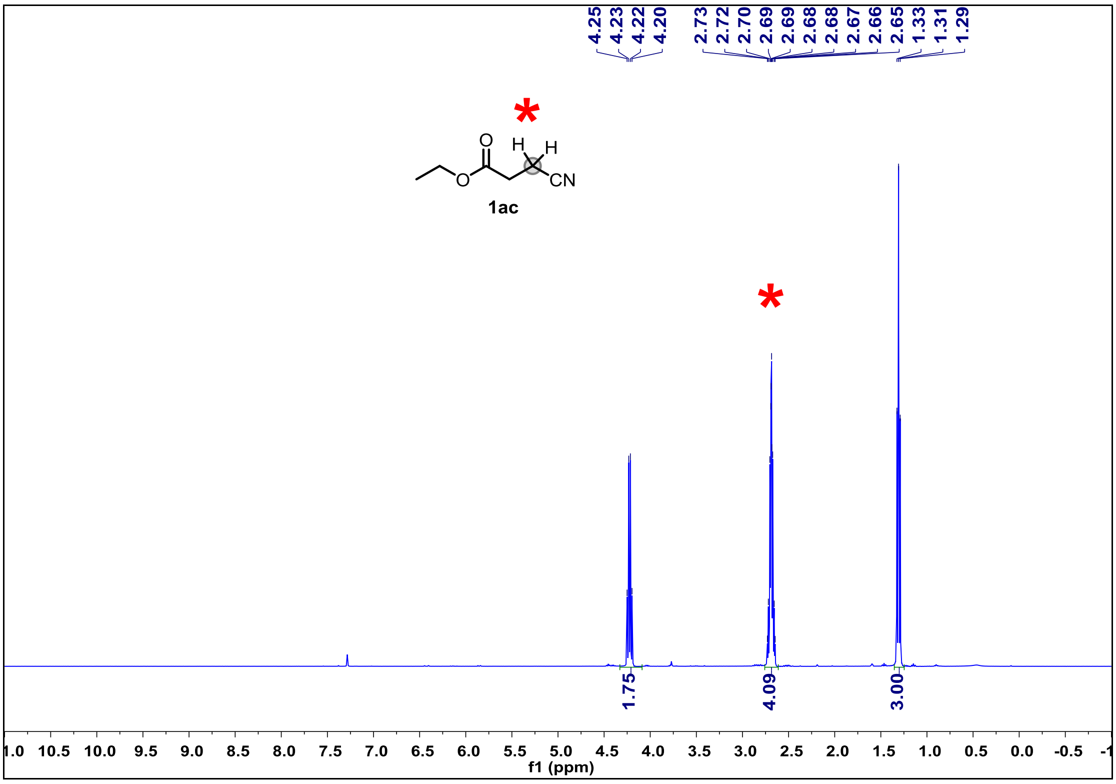

Figure S126. ^{1}H NMR spectrum of nitrile **1ac** (400 MHz, $CDCl_3$, 298 K).

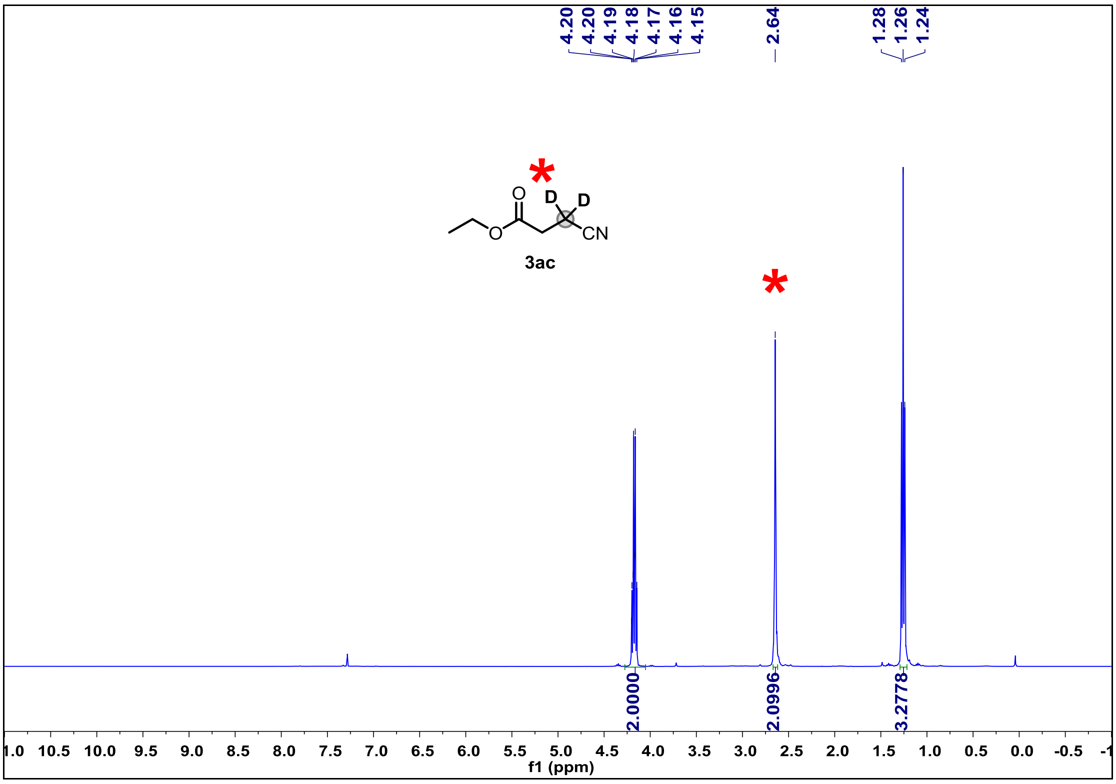

Figure S127. ^{1}H NMR spectrum of α- deuterated nitrile **3ac** (100 MHz, $CDCl_3$, 298 K).

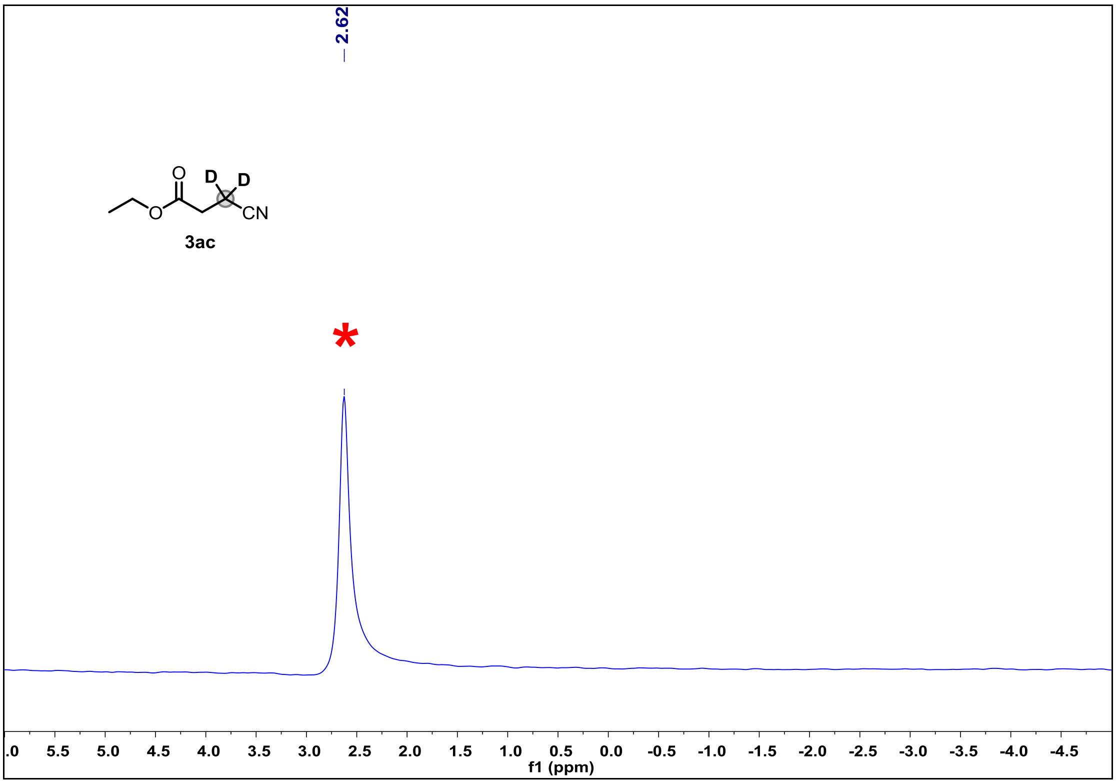

Figure S128. ^{2}H NMR spectrum of α- deuterated nitrile **3ac** (61 MHz, $CDCl_3$, 298 K).

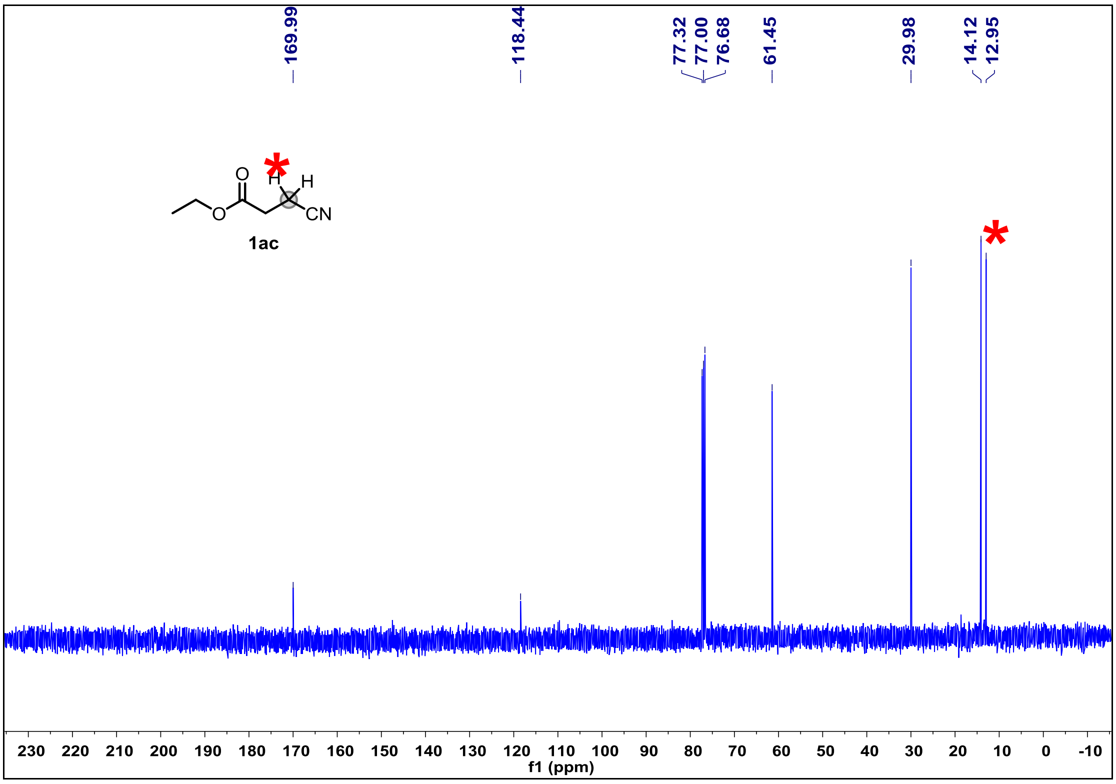

Figure S129. $^{13}C\{^{1}H\}$ NMR spectrum of nitrile **1ac** (125 MHz, $CDCl_3$, 298 K).

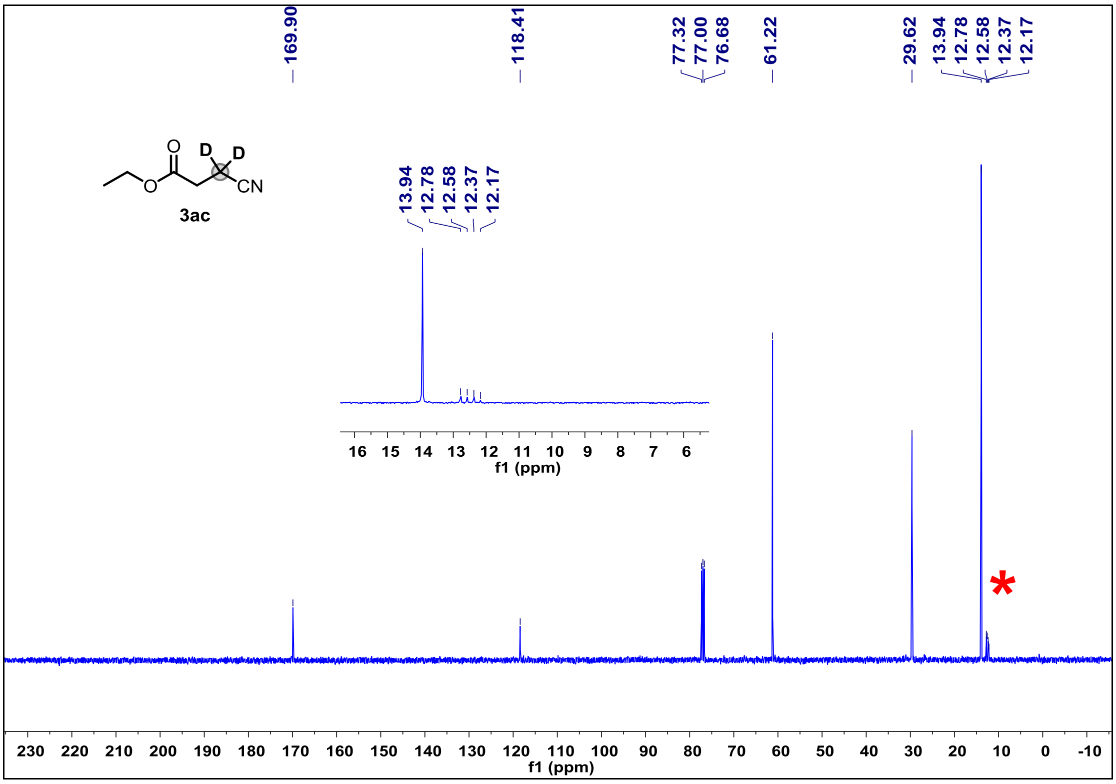

Figure S130. $^{13}C\{^{1}H\}$ NMR spectrum of α- deuterated nitrile **3ac** (100 MHz, $CDCl_3$, 298 K).

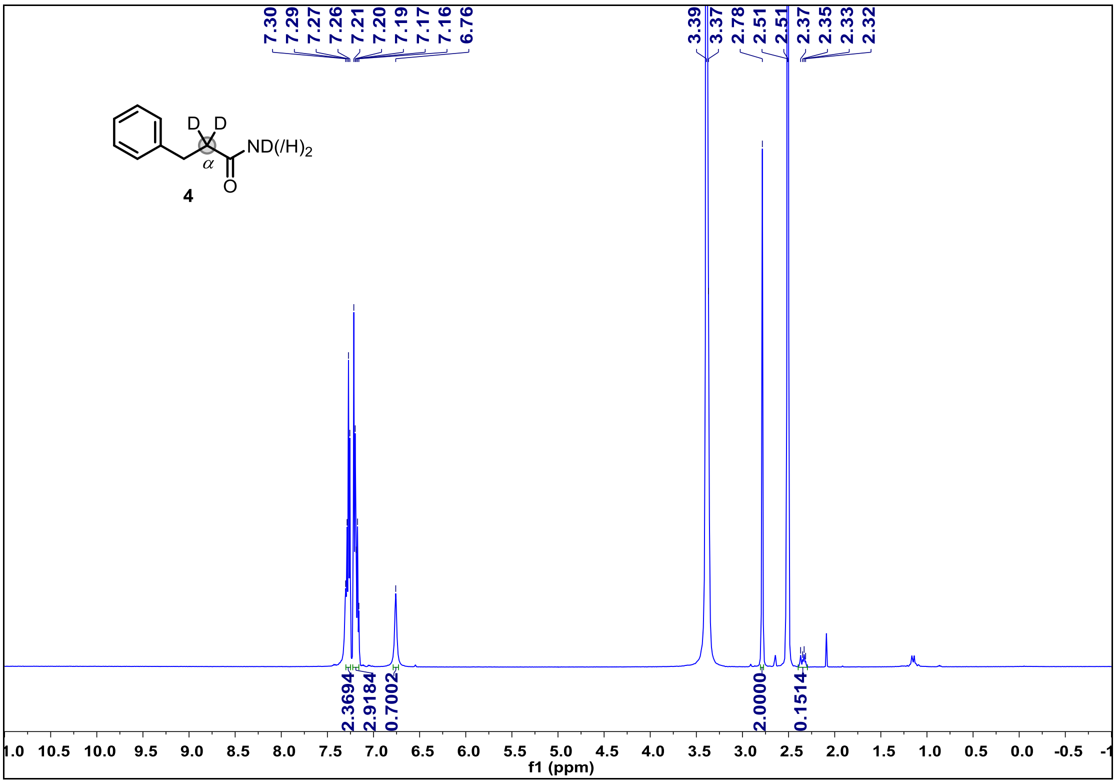

Figure S131. ^{1}H NMR spectrum of α- deuterated amide **4** (500 MHz, $CDCl_3$, 298 K).

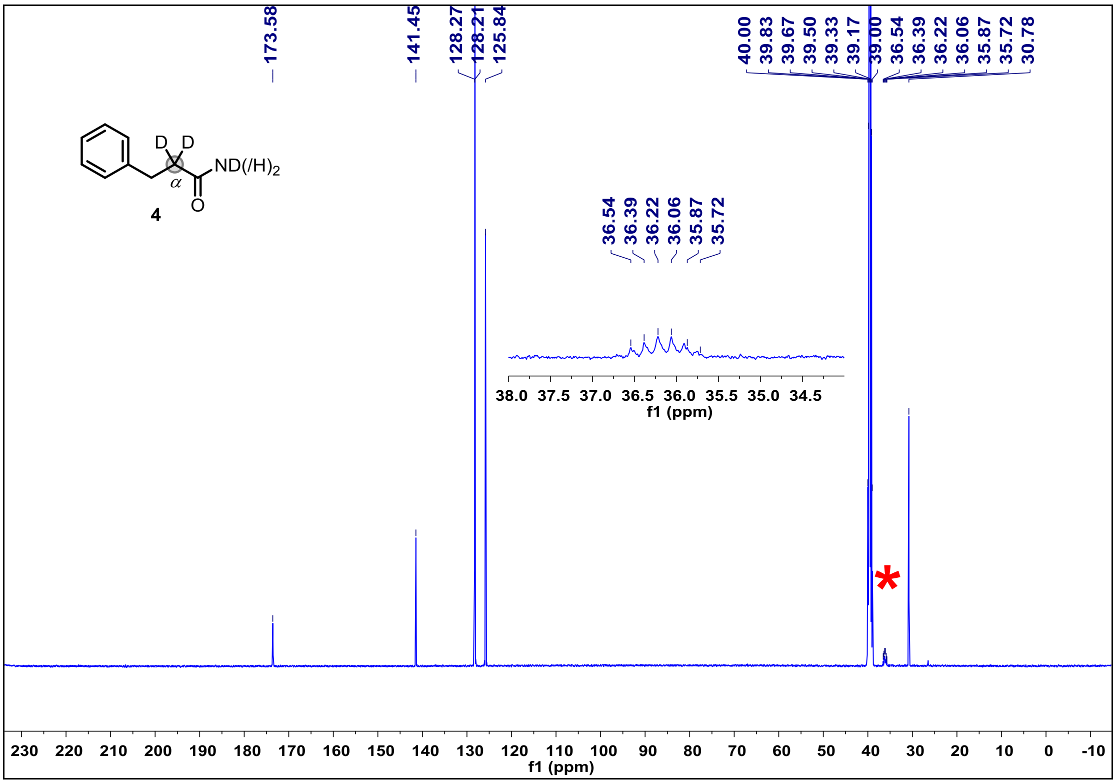

Figure S132. $^{13}C\{^{1}H\}$ NMR spectrum of α- deuterated amide **4** (125 MHz, $CDCl_3$, 298 K).

7. References

[1] Armarego, W. L. F.; Perrin, D. D. *Purification of Laboratory Chemicals;* 4th Edition; Butterworth-Heineman: Oxford, **2000**.

[2] Still, W.C.; Kahn, M.; Mitra, A. Rapid Chromatographic Technique for Preparative Separations with Moderate Resolution. *J. Org. Chem*. **1987**, *43*, 2923-2925.

[3] Gottlieb, H. E.; Kotlyar, V.; Nudelman, A. NMR Chemical Shifts of Common Laboratory Solvents as Trace Impurities. *J. Org. Chhem.* **1997**, *62*, 7512-7515.

[4] Nerush, A.; Vogt, M.; Gellrich, U.; Leitus, G.; Ben-David, Y. D.; Milstein, Template Catalysis by Metal–Ligand Cooperation. C–C Bond Formation via Conjugate Addition of Non-activated Nitriles under Mild, Base-free Conditions Catalyzed by a Manganese Pincer Complex. *J. Am. Chem. Soc*. **2016**, *138*, 6985-6997.

[5] Espinosa-Jalapa, N. A.; Kumar, A.; Leitus, G.; Diskin-Posner, Y.; Milstein, D. Synthesis of Cyclic Imides by Acceptorless Dehydrogenative Coupling of Diols and Amines Catalyzed by a Manganese Pincer Complex. *J. Am, Chem. Soc.* **2017**, *139*, 11722-11725.

[6] Das, U. K.; Ben-David, Y.; Leitus, G.; Diskin-Posner, Y.; Milstein, D. Dehydrogenative Cross-Coupling of Primary Alcohols to form Cross-Esters Catalyzed by a Manganese Pincer Complex. *ACS Catal.* **2019**, *9*, 479-484.

[7] Daw, P.; Kumar, A.; Espinosa-Jalapa, N. A.; Ben-David, Y.; Milstein, D. Direct Synthesis of Amides by Acceptorless Dehydrogenative Coupling of Benzyl Alcohols and Ammonia Catalyzed by a Manganese Pincer Complex: Unexpected Crucial Role of Base. *J. Am. Chem. Soc.* **2019**, *141*, 12202-12206.

[8] Sheldrick, G. M. *SHELXT* -Integrated Space-Group and Crystal-Structure Determination. *Acta Cryst.* **2015**, *A71*, 3–8.

[9] Sheldrick, G. M. Crystal Structure Refinement with SHELXL. *Acta Cryst.* **2015**, *C71*, 3–8.

[10] Zhao, Y.; Truhlar, D. G., A new local density functional for main-group thermochemistry, transition metal bonding, thermochemical kinetics, and noncovalent interactions. *J. Chem. Phys.* **2006,** *125*, 194101.

[11] Weigend, F.; Ahlrichs, R., Balanced basis sets of split valence, triple zeta valence and quadruple zeta valence quality for H to Rn: Design and assessment of accuracy. *Phys. Chem. Chem. Phys.* **2005,** *7*, 3297-3305.

[12] Weigend, F., Accurate Coulomb-fitting basis sets for H to Rn. *Phys. Chem. Chem. Phys.* **2006,** *8*, 1057-1065.

[13] Grimme, S.; Antony, J.; Ehrlich, S.; Krieg, H., A consistent and accurate ab initio parametrization of density functional dispersion correction (DFT-D) for the 94 elements H-Pu. *J. Chem. Phys.* **2010,** *132*, 154104.

[14] Mardirossian, N.; Head-Gordon, M., ωB97M-V: A combinatorially optimized, range-separated hybrid, meta-GGA density functional with VV10 nonlocal correlation. *J. Chem. Phys.* **2016,** *144*, 214110.

[15] (a) Vydrov, O. A.; Van Voorhis, T., Nonlocal van der Waals density functional: The simpler the better. *J. Chem. Phys.* **2010,** *133*, 244103; (b) Hujo, W.; Grimme, S., Performance of the van der Waals Density Functional VV10 and (hybrid)GGA Variants for Thermochemistry and Noncovalent Interactions. *J. CHem. Theory Comput.* **2011,** *7*, 3866-3871.

[16] Hellweg, A.; Hättig, C.; Höfener, S.; Klopper, W., Optimized accurate auxiliary basis sets for RI-MP2 and RI-CC2 calculations for the atoms Rb to Rn. *Theor. Chem. Acc.* **2007,** *117*, 587-597.

[17] Frisch, M. J.; Trucks, G. W.; Schlegel, H. B.; Scuseria, G. E.; Robb, M. A.; Cheeseman, J. R.; Scalmani, G.; Barone, V.; Petersson, G. A.; Nakatsuji, H.; Li, X.; Caricato, M.; Marenich, A. V.; Bloino, J.; Janesko, B. G.; Gomperts, R.; Mennucci, B.; Hratchian, H. P.; Ortiz, J. V.; Izmaylov, A. F.; Sonnenberg, J. L.; Williams; Ding, F.; Lipparini, F.; Egidi, F.; Goings, J.; Peng, B.; Petrone, A.; Henderson, T.; Ranasinghe, D.; Zakrzewski, V. G.; Gao, J.; Rega, N.; Zheng, G.; Liang, W.; Hada, M.; Ehara, M.; Toyota, K.; Fukuda, R.; Hasegawa, J.; Ishida, M.; Nakajima, T.; Honda, Y.; Kitao, O.; Nakai, H.; Vreven, T.; Throssell, K.; Montgomery Jr., J. A.; Peralta, J. E.; Ogliaro, F.; Bearpark, M. J.; Heyd, J. J.; Brothers, E. N.; Kudin, K. N.; Staroverov, V. N.; Keith, T. A.; Kobayashi, R.; Normand, J.; Raghavachari, K.; Rendell, A. P.; Burant, J. C.; Iyengar, S. S.; Tomasi, J.; Cossi, M.; Millam, J. M.; Klene, M.; Adamo, C.; Cammi, R.; Ochterski, J. W.; Martin, R. L.; Morokuma, K.; Farkas, O.; Foresman, J. B.; Fox, D. J. *Gaussian 16 Rev. C.01*, Wallingford, CT, 2016.

[18] Neese, F., Software update: the ORCA program system, version 4.0. *WIREs Comput. Mol. Sci.* **2018,** *8*, e1327.
